# Regional variability in the Acheulian to Middle Stone Age transition in southern Africa

**DOI:** 10.1038/s41598-026-40075-8

**Published:** 2026-03-19

**Authors:** A. F. Blackwood, J. Wilkins, L. J. Arnold, M. Demuro, G. Boschian, M. V. Caruana, E. F. Lalunio, M. Spate, A. Hatton, R. A. Muir, C. G. Wilson, L. J. Quick, M. Meredith-Williams, A. I. R. Herries

**Affiliations:** 1 Human Palaeosystems Group, Max Planck Institute of Geoanthropology, Jena, Germany; 2 ARC Centre of Excellence for Transforming Human Origins Research, Department of Archaeology and History, La Trobe University, Bundoora, Naarm, VIC 3086 Australia; 3 Department of Geological Sciences, Human Evolution Research Institute (HERI), University of Cape Town, Rondebosch, South Africa; 4 School of Environment and Science, Griffith University, Brisbane, Australia; 5 School of Physics, Chemistry and Earth Sciences, Environment Institute and Institute for Photonics and Advanced Sensing (IPAS), Adelaide University, Adelaide, Australia; 6 Department of Biology, University of Pisa, Pisa, Italy; 7 The Palaeo-Research Institute, University of Johannesburg, Johannesburg, South Africa; 8 Department of Earth Sciences, University of the Western Cape, Cape Town, South Africa; 9 Evolutionary Studies Institute, University of Witwatersrand, Johannesburg, South Africa; 10 African Centre for Coastal Palaeoscience, Nelson Mandela University, Gqeberha, South Africa

**Keywords:** Middle Stone Age, Earlier Stone Age, Acheulian, Southern Africa, Human evolution, Ecology, Ecology, Evolution, Solid Earth sciences

## Abstract

*Homo sapiens* emerged in Africa around 300 − 200 thousand years ago (ka). Although the earliest *H. sapiens* fossils are associated with the Middle Stone Age (MSA), lithic technologies considered diagnostic of the MSA have been found alongside Acheulian technology in eastern Africa and the interior of southern Africa by ~ 500 − 400 ka, suggesting a deep evolutionary history of our species in these regions. The southern coastal plain of South Africa, geographically separated from the interior by the Cape Fold Belt and Great Escarpment, has one of the best documented records of the MSA in Africa; however, only a single site is older than 125 ka and little is known about the origins of the MSA in this region. Here, we report a stratified sequence of Acheulian to MSA lithic assemblages from the open-air site of Amanzi Springs covering the period between ~ 379 to 95 ka. We show that the MSA emerged around 230 ± 18 ka, significantly earlier than previously documented along the southern coast. The pattern of technological change also differs to the interior, with no diagnostic MSA elements found in the late Acheulian, although persistent methods of flake production indicate a gradual transition and continuity into the MSA. The relatively late emergence of the MSA along the southern coast highlights the variable and complex nature of demographic and behavioural change during this period, with regionally distinct technological trajectories extending into the Middle Pleistocene in southern Africa.

**Supplementary Information:**

The online version contains supplementary material available at 10.1038/s41598-026-40075-8.

## Introduction

The evolutionary history of *Homo sapiens* is increasingly understood as a continent-wide process, with interactions between structured populations across Africa contributing to the mosaic-like appearance of our species^1–6^. This pan-African model of human evolution emphasises the role of sporadic gene flow between semi-isolated populations living in diverse environments, promoting adaptation to local ecological conditions^1,4^. Archaeological and fossil archives dating to the late Middle Pleistocene document several major biogeographic and cultural transitions involving a diverse range of environments across Africa, including the earliest fossils attributed to the *H. sapiens* lineage, directly dated to 286 ± 32 ka in northern Africa^2,7^, 259 ± 35 ka in southern Africa^8,9^, and possibly as old as 233 ± 22 ka in eastern Africa^10^. The most significant cultural change during this period is the shift from the Acheulian, a technocomplex of the Earlier Stone Age (ESA), to the MSA, a division of the archaeological record that encompasses the earliest material traces associated with our species. This process is characterized by a decline in Acheulian modes of technology that had persisted for more than a million years prior, particularly the production of large cutting tools (LCTs, e.g., handaxes and cleavers), alongside the appearance and elaboration of prepared core technologies such as the Levallois method used to produce flake and blade-based toolkits^5,11–15^. These elements of MSA lithic technology first occur within Acheulian assemblages in eastern Africa^14,16–19^, and in assemblages with a mosaic of Acheulian and MSA characteristics in the interior of South Africa^20,21^, well before the onset of the MSA. Moreover, the manufacture of LCTs persisted in some regions long after the first appearance of the MSA^22–24^, highlighting the variable and complex nature of the archaeological record of this period and the difficulties involved in identifying the origins of the MSA. Developing robust spatio-temporal frameworks that account for these complexities is necessary to test the pan-African model and requires well dated and regionally contextualised archaeological archives that document this period of transition.

**Fig. 1:**
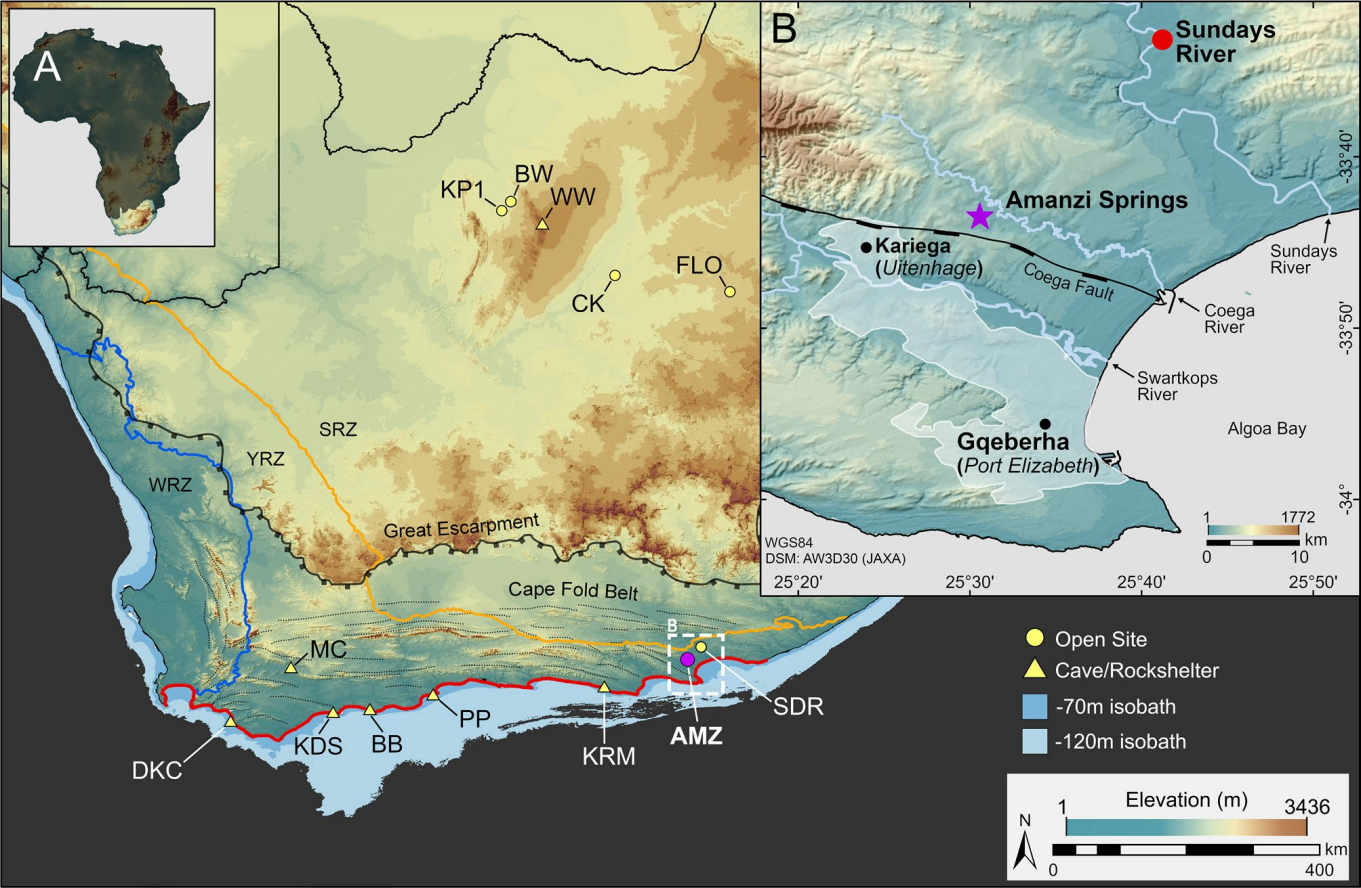
Location of Amanzi Springs Area 7 (**A**) Map of South Africa, showing sites along the southern coastal plain (highlighted in red), and early MSA sites in the interior: Die Kelders 1 (DKC), Montagu Cave (MC), Klipdrift Shelter (KDS), Blombos Cave (BB), Pinnacle Point (PP), Klasies River Mouth (KRM), Amanzi Springs (AMZ), Sundays River (SDR), Kathu Pan 1 (KP1), Bestwood 1 (BW), Wonderwerk Cave (WW), Canteen Koppie (CK), and Florisbad (FLO). Bathymetry from^51^, winter (WRZ), year-round (YRZ), and summer (SRZ) rainfall zones from^42^. (**B**) Location of Amanzi Springs within the Algoa Bay region. Figure produced using ArcGIS Pro 3.4 (esri.com) and the ALOS Global Digital Surface Model (JAXA).

In southern Africa, the earliest assemblages that lack LCTs first appear on the Southern African Plateau by ~ 291 − 279 ka^8,20,25^, signalling the onset of the MSA in the Savanna and Grassland biomes in the interior of South Africa. In contrast, much less is known about when and where this transition first appeared in the Thicket and Fynbos biomes on the southern coastal plain, a region that is well-known for some of the earliest examples of symbolic and complex behaviour in the archaeological record^26–32^. Most of these sites are found in caves and rock-shelters younger than ~ 125 ka, with only a single site dated to Marine Isotope Stage 6^26,33–36^ (MIS; age ranges from^37^. Our understanding of early modern human inhabitation in this region is therefore skewed towards residential sites from MIS 5 onwards, providing only a narrow window into past human behaviour and lifeways within the wider coastal landscape^38^. The rarity of detailed Middle Pleistocene archaeological archives from this region, and the limited number of open-air sites, restricts our ability to evaluate the behavioural, cultural, and ecological dynamics associated with the emergence of the MSA in coastal South Africa. To address these issues and investigate the stratigraphic and chronological context of this transition along the southern coastal plain, we conducted excavations at Amanzi Springs Area 7 (AMZ7), a stratified open-air site ~ 20 km inland from Algoa Bay in the Eastern Cape Provence, South Africa (Fig. 1).

Located at the eastern end of the Cape Fold Belt mountains and the broad, shallow continental shelf of the Agulhas Bank that formed the Palaeo-Agulhas Plain (PAP)^39–41^, Amanzi Springs falls within the modern-day Albany Thicket Biome in the year-round rainfall zone^42,43^ (Fig. 1). Two of the springs, Areas 1 and 2, were first excavated in the 1960s^44,45^, and are known for their Acheulian deposits and preservation of wood. Recent work has established that the Acheulian layers at Areas 1 and 2 date to between 534 − 390 ka^46–48^, making them one of the few dated ESA sites along Africa’s southern coast^49,50^. Here we report new findings from excavations at Area 7 that have revealed a sequence of Acheulian to MSA assemblages, documenting the shift from the Acheulian to the MSA in a well-contextualized, non-cave setting. Multiple luminescence methods place the onset of the MSA at Amanzi Springs by 230 ± 18 ka (1σ), extending the origins of the MSA in this region and providing our first insights into the emergence of the MSA along Africa’s southern coastal plain.

## Results

### Site formation and chronology

**Fig. 2:**
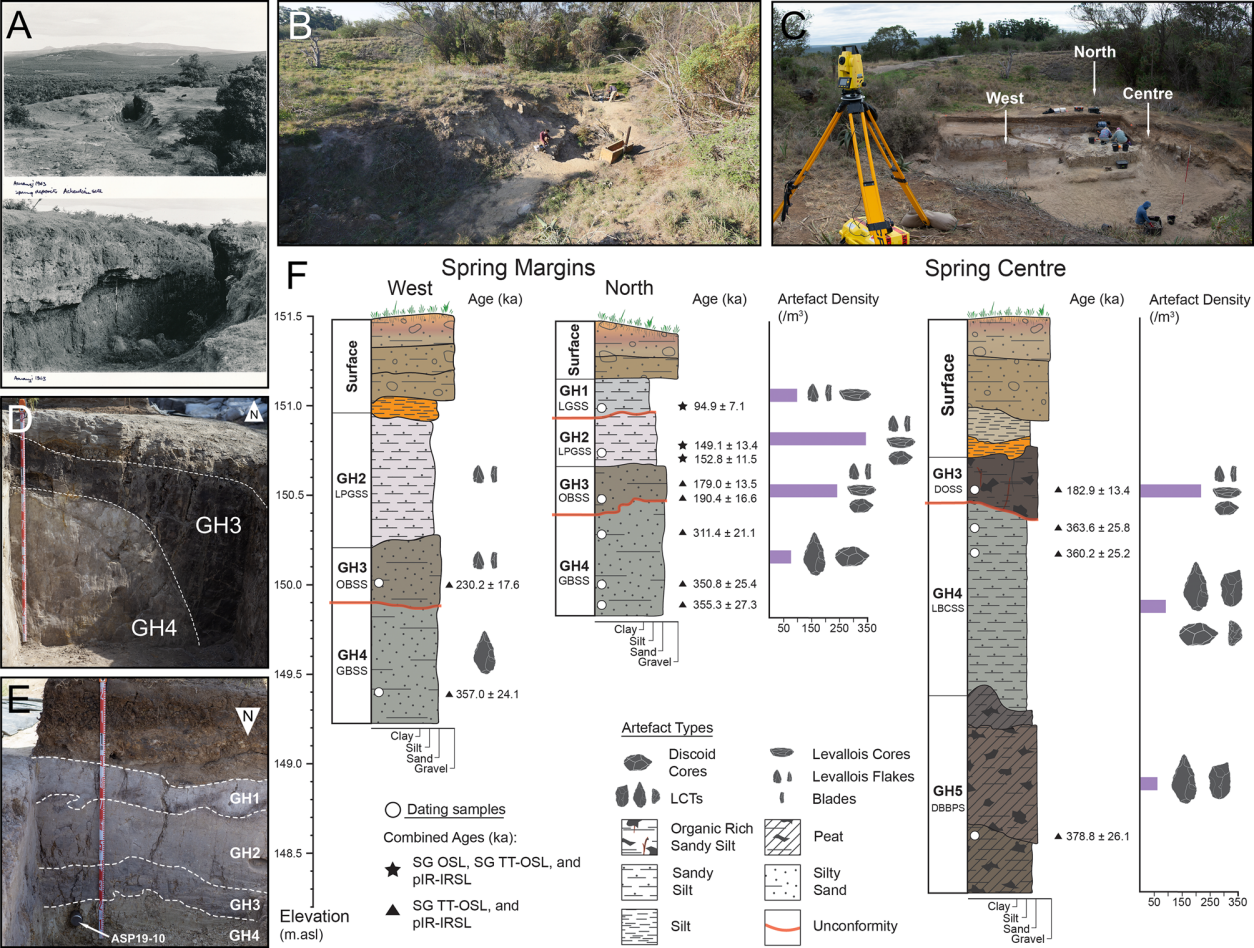
The Area 7 Spring. (**A**) Photos of Area 7 taken by Hilary Deacon in 1963, and (**B**,**C**) the spring before and during excavation in 2017. (**D**) North section showing the steep bank of the LBCSS that separates GH3 (DOSS) from GH4, (**E**) south section showing the location of OSL sample ASP19-10 (311.4 ± 21.1 ka) and the GH1-4 sequence towards the north of the spring. (**F**) Stratigraphic logs of the sedimentary sequence at the margins of the spring to the west and north, and the spring centre.

Five phases of spring formation were identified at AMZ7, referred to here as geological horizons (GH) 1–5, numbered from the top to the base of the sequence and defined by their unifying sedimentological characteristics and chronological age (Fig. 2; Fig. S10; Supplementary Text 2–3). Each horizon represents a continuous, long-term period of sediment deposition across the site, incorporating contemporaneous but distinct facies at the margins and towards the centre of the spring basin. Single-grain thermally transferred (SG TT-OSL), single-grain optically stimulated luminescence (SG OSL), and multiple-grain post-infrared infrared stimulated luminescence (MG pIR-IRSL) dating methods provide stratigraphically consistent ages for the sequence, ranging from 379 ± 26 to 95 ± 7 ka (Fig. 2; Table 1; age ranges presented herein are the weighted mean luminescence ages for each sample and their associated 1σ uncertainties).

**Table 1:** Dose rate data, equivalent doses (D_e_), and luminescence ages for Amanzi Springs Area 7.

Sample	Unit	Grain size (µm)	Water content (%) ^a^	Environmental dose rate (Gy/ka)					Equivalent dose (D_e_) data					Age (ka) ^g, l^		Combined age (ka) ^g, m^	
				Beta dose rate ^b^	Gamma dose rate ^c^	Cosmic dose rate ^d^	Internal dose rate ^e, f^	Total dose rate ^g^	D_e_ type ^h^	No. of grains or aliquots ^i^	Over- dispersion (%) ^j^	Age model ^k^	D_e_ (Gy) ^g^				
ASP19-6	LGSS	212–250	8 / 15	0.88 ± 0.04	0.74 ± 0.03	0.12 ± 0.01	0.03 ± 0.01	1.77 ± 0.11	SG OSL	158 / 800	29 ± 2	CAM	169 ± 5	95.5 ± 6.9		94.9 ± 7.1	
	(GH1)	212–250		0.88 ± 0.04	0.74 ± 0.03	0.12 ± 0.01	0.03 ± 0.01	1.77 ± 0.11	SG TT-OSL	75 / 1000	26 ± 6	CAM	168 ± 9	94.8 ± 8.1			
		90–125		0.94 ± 0.05	0.74 ± 0.03	0.12 ± 0.01	0.49 ± 0.04	2.29 ± 0.12	MG pIR-IRSL	12 / 12	11 ± 2	CAM	216 ± 7	94.5 ± 6.3			
ASP18-7	LPGSS	212–250	11 / 24	0.74 ± 0.04	0.61 ± 0.02	0.11 ± 0.01	0.03 ± 0.01	1.49 ± 0.09	SG OSL	63 / 800	31 ± 4	CAM	214 ± 10	143.8 ± 11.3		149.1 ± 13.4	
	(GH2)	212–250		0.74 ± 0.04	0.61 ± 0.02	0.11 ± 0.01	0.03 ± 0.01	1.49 ± 0.09	SG TT-OSL	103 / 1000	35 ± 4	MAM-3	242 ± 21	162.5 ± 17.7			
		90–125		0.80 ± 0.04	0.61 ± 0.02	0.11 ± 0.01	0.49 ± 0.04	2.00 ± 0.10	MG pIR-IRSL	10 / 10	19 ± 4	CAM	298 ± 18	148.8 ± 12.2			
ASP19-14	LPGSS	212–250	6 / 25	0.71 ± 0.04	0.58 ± 0.02	0.11 ± 0.01	0.03 ± 0.01	1.43 ± 0.09	SG OSL	94 / 800	30 ± 3	CAM	213 ± 8	148.9 ± 11.0		152.8 ± 11.5	
	(GH2)	212–250		0.71 ± 0.04	0.58 ± 0.02	0.11 ± 0.01	0.03 ± 0.01	1.43 ± 0.09	SG TT-OSL	72 / 1000	28 ± 5	CAM	230 ± 12	160.8 ± 13.2			
		90–125		0.76 ± 0.04	0.58 ± 0.02	0.11 ± 0.01	0.49 ± 0.04	1.94 ± 0.10	MG pIR-IRSL	12 / 12	15 ± 3	CAM	293 ± 13	151.2 ± 10.5			
ASP18-9	OBSS	212–250	18 / 29	0.82 ± 0.04	0.63 ± 0.02	0.10 ± 0.01	0.03 ± 0.01	1.59 ± 0.09	SG TT-OSL	84 / 1000	48 ± 5	MAM-3	309 ± 30	194.2 ± 22.6		190.4 ± 16.6	
	(GH3)	90–125		0.88 ± 0.04	0.63 ± 0.02	0.10 ± 0.01	0.49 ± 0.04	2.10 ± 0.10	MG pIR-IRSL	12 / 12	7 ± 1	CAM	399 ± 8	189.5 ± 10.9			
ASP18-11	OBSS	212–250	19 / 36	0.77 ± 0.04	0.60 ± 0.02	0.09 ± 0.01	0.03 ± 0.01	1.50 ± 0.09	SG TT-OSL	84 / 1000	29 ± 4	CAM	354 ± 16	236.8 ± 18.0		230.2 ± 17.6	
	(GH3)	90–125		0.83 ± 0.04	0.60 ± 0.02	0.09 ± 0.01	0.49 ± 0.04	2.01 ± 0.10	MG pIR-IRSL	12 / 12	19 ± 4	CAM	450 ± 25	224.1 ± 17.2			
ASP19-11	DOSS	212–250	12 / 22	0.79 ± 0.04	0.61 ± 0.02	0.11 ± 0.01	0.03 ± 0.01	1.54 ± 0.10	SG TT-OSL	56 / 2000	30 ± 6	CAM	304 ± 18	197.8 ± 17.3		179.0 ± 13.5	
	(GH3)	90–125		0.85 ± 0.04	0.61 ± 0.02	0.11 ± 0.01	0.49 ± 0.04	2.05 ± 0.11	MG pIR-IRSL	10 / 10	10 ± 2	CAM	352 ± 11	171.5 ± 10.9			
ASP18-4	DOSS	212–250	10 / 21	0.79 ± 0.04	0.63 ± 0.02	0.11 ± 0.01	0.03 ± 0.01	1.55 ± 0.10	SG TT-OSL	101 / 1000	34 ± 4	CAM	294 ± 14	189.6 ± 15.2		182.9 ± 13.4	
	(GH3)	90–125		0.85 ± 0.04	0.63 ± 0.02	0.11 ± 0.01	0.49 ± 0.04	2.06 ± 0.11	MG pIR-IRSL	11 / 12	12 ± 3	CAM	369 ± 13	178.9 ± 11.8			
ASP19-10	LBCSS	212–250	17 / 38	0.78 ± 0.04	0.58 ± 0.02	0.09 ± 0.01	0.03 ± 0.01	1.48 ± 0.09	SG TT-OSL	54 / 1000	25 ± 5	CAM	477 ± 24	323.3 ± 25.8		311.4 ± 21.1	
	(GH4)	90–125		0.83 ± 0.04	0.58 ± 0.02	0.09 ± 0.01	0.49 ± 0.04	1.99 ± 0.10	MG pIR-IRSL	10 / 10	6 ± 1	CAM	608 ± 12	306.2 ± 17.1			
ASP19-12	LBCSS	212–250	11 / 21	0.67 ± 0.03	0.54 ± 0.02	0.10 ± 0.01	0.03 ± 0.01	1.34 ± 0.08	SG TT-OSL	59 / 1000	25 ± 5	CAM	468 ± 25	348.1 ± 29.4		350.8 ± 25.4	
	(GH4)	90–125		0.72 ± 0.04	0.54 ± 0.02	0.10 ± 0.01	0.49 ± 0.04	1.85 ± 0.09	MG pIR-IRSL	10 / 10	8 ± 2	CAM	651 ± 17	352.2 ± 21.3			
ASP19-13	LBCSS	212–250	24 / 47	0.82 ± 0.04	0.54 ± 0.02	0.08 ± 0.01	0.03 ± 0.01	1.48 ± 0.08	SG TT-OSL	82 / 1000	23 ± 4	CAM	525 ± 21	355.7 ± 25.7		355.3 ± 27.3	
	(GH4)	90–125		0.88 ± 0.04	0.54 ± 0.02	0.08 ± 0.01	0.49 ± 0.04	1.99 ± 0.09	MG pIR-IRSL	11 / 12	21 ± 4	CAM	706 ± 44	354.9 ± 28.8			
ASP18-10	GBSS	212–250	15 / 33	0.89 ± 0.05	0.63 ± 0.02	0.08 ± 0.01	0.03 ± 0.01	1.63 ± 0.10	SG TT-OSL	66 / 1000	20 ± 5	CAM	576 ± 24	352.4 ± 26.6		357.0 ± 24.1	
	(GH4)	90–125		0.96 ± 0.05	0.63 ± 0.02	0.08 ± 0.01	0.49 ± 0.04	2.15 ± 0.11	MG pIR-IRSL	12 / 12	8 / 2	CAM	775 ± 20	360.0 ± 21.4			
ASP18-6	LBCSS	212–250	10 / 23	0.72 ± 0.04	0.60 ± 0.02	0.10 ± 0.01	0.03 ± 0.01	1.45 ± 0.09	SG TT-OSL	108 / 1000	28 ± 3	CAM	506 ± 19	349.7 ± 26.3		360.2 ± 25.2	
	(GH4)	90–125		0.77 ± 0.04	0.60 ± 0.02	0.10 ± 0.01	0.49 ± 0.04	1.95 ± 0.10	MG pIR-IRSL	11 / 11	11 ± 3	CAM	721 ± 25	368.9 ± 23.9			
ASP18-5	LBCSS	212–250	13 / 27	0.82 ± 0.04	0.60 ± 0.02	0.10 ± 0.01	0.03 ± 0.01	1.55 ± 0.09	SG TT-OSL	89 / 1000	36 ± 4	CAM	546 ± 26	351.9 ± 28.2		363.6 ± 25.8	
	(GH4)	90–125		0.88 ± 0.04	0.60 ± 0.02	0.10 ± 0.01	0.49 ± 0.04	2.07 ± 0.10	MG pIR-IRSL	12 / 12	10 ± 2	CAM	767 ± 23	371.4 ± 23.0			
ASP18-12	DBBPS	212–250	30 / 38	0.62 ± 0.03	0.50 ± 0.02	0.07 ± 0.01	0.03 ± 0.01	1.23 ± 0.07	SG TT-OSL	65 / 1000	28 ± 4	CAM	486 ± 23	396.6 ± 30.8		378.8 ± 26.1	
	(GH5)	90–125		0.67 ± 0.03	0.50 ± 0.02	0.07 ± 0.01	0.49 ± 0.04	1.73 ± 0.08	MG pIR-IRSL	11 / 12	10 ± 2	CAM	638 ± 20	369.5 ± 22.2			

^a^Present-day water content / long-term estimated water content, expressed as % of dry mass of mineral fraction, with an assigned 1σ uncertainty of ± 5%. The long-term water content of samples collected from within or adjacent to the spring eye (ASP18-12) is taken as 100% of the saturated water content. The long-term water content of samples collected further away from the spring eye (all other samples considered here) is taken as 70% of the saturated water content^47,48^.

^b^Beta dose rates were calculated using a Risø GM-25-5 low-level beta counter^52^, after making allowance for beta dose attenuation due to grain-size effects and HF etching^53,54^. Radionuclide concentrations and specific activities of beta counting standards have been converted to dose rates using the conversion factors given in Guérin et al.^55^.

^c^Gamma dose rates were calculated from in situ measurements made at each sample position with a NaI: Tl detector using the ‘energy windows’ method detailed in Arnold et al.^56^ and Duval and Arnold^57^. Radionuclide concentrations and specific activities of gamma spectrometry calibration materials, and K, U, Th concentrations determined from the field gamma-ray spectra have been converted to dose rates using the conversion factors given in Guérin et al.^55^.

^d^Cosmic-ray dose rates were calculated according to Prescott and Huntley^58^and assigned a relative 1σ uncertainty of ± 10%.

^e^The assumed internal alpha + beta dose rate for quartz, with an assigned relative 1σ uncertainty of ± 30%, is based on intrinsic ^238^U and ^232^Th contents published by Mejdahl^59^, Bowler et al.^60^, Jacobs et al.^61^, Pawley et al.^62^, and Lewis et al.^63^, and an a-value of 0.04 ± 0.01^64,65^. Intrinsic radionuclide concentrations and specific activities have been converted to dose rates using the conversion factors given in Guérin et al.^55^, making allowance for beta dose attenuation due to grain-size effects^53^.

^f^The assumed internal feldspar dose rate is based on assumed internal ^40^K and ^87^Rb concentrations of 12.5 ± 0.5%^66^ and 400 ± 100 ppm^67^, respectively, yielding an internal beta dose rate of 0.43 ± 0.03 Gy / ka for the 90–125 μm K-feldspar grains measured in this study. An additional internal alpha + beta dose rate of 0.06 ± 0.03 Gy / ka has been calculated for the K-feldspar fractions using assumed intrinsic ^238^U and ^232^Th concentrations of 0.15 ± 0.03 ppm and 0.35 ± 0.07 ppm, respectively^59,68–70^, and an a-value of 0.09 ± 0.03^64,71–75^. Intrinsic radionuclide concentrations and specific activities have been converted to dose rates using the conversion factors given in Guérin et al.^55^ and Readhead^76^, making allowance for beta dose attenuation due to grain-size effects^53,76^.

^g^Mean ± total uncertainty (68% confidence interval), calculated as the quadratic sum of the random and systematic uncertainties.

^h^SG OSL = quartz single-grain optically stimulated luminescence; SG TT-OSL = quartz single-grain thermally transferred OSL; MG pIR-IRSL = k-feldspar multi-grain aliquot post-IR IRSL performed at 250 °C.

^i^Number of D_e_ measurements that passed the SAR rejection criteria and were used for D_e_ determination / total number of D_e_ values analysed.

^j^The relative spread in the D_e_ dataset beyond that associated with the measurement uncertainties for individual D_e_ values.

^k^Age model used to calculate the sample-averaged D_e_ value for each sample. MAM-3 = 3-parameter minimum age model; CAM = central age model^77^. Single-grain TT-OSL MAM-3 D_e_ estimates were calculated after adding, in quadrature, a relative error of 20% to each individual D_e_ measurement error to approximate the underlying dose overdispersion observed for ‘ideal’ (well-bleached and unmixed) sedimentary samples from Amanzi Springs sites (e.g. ASP18-10, ASP19-13; plus ASP18-18 and ASP18-2 from^47,48^).

^l^Total uncertainty includes a systematic component of ± 2% associated with laboratory beta-source calibration.

^m^The comparative luminescence ages available for each sample have been combined to produce a single, weighted average age estimate for the purpose of chronological interpretations. The associated 1σ uncertainty on this combined age has been calculated by multiplying the average relative uncertainties of the replicate age estimates by the combined weighted average age estimate.

From the base of the sequence to the top, the lowermost phase of spring formation exposed by excavation is GH5, with a weighted mean age of 379 ± 26 ka, centred on late MIS 11. It contains the dark brown to black peaty sands (DBBPS), a stratigraphic aggregate with abundant preserved wood, organics, and macrofossils. The high frequency of vegetal residues in life position indicates that DBBPS formed during a phase of still water in an environment that favoured riparian vegetation and the formation of turf. Pollen from this unit is dominated by *Cliffortia*, a taxon associated with fynbos vegetation of the Cape Floristic Region (CFR)^43^, as well as wetland taxa that includes sedges (Cyperaceae), and monolete and trilete spores from mosses and ferns (Supplementary Text 4).

A sharp stratigraphic boundary separates GH5 from the overlying GH4, which consists of two units; the light brown compact sandy silt (LBCSS), and the laterally equivalent green to brown silty sand (GBSS). The sedimentologic characteristics of GH4 and the absence of vegetal remains within it suggest that water levels were raised above the top of DBBPS, forming a well-oxygenated basin. TT-OSL and pIR-IRSL ages from LBCSS and GBSS bracket these layers to 360 ± 25 and 357 ± 24 ka (centred on MIS 10). At the spring centre, the upper boundary of the LBCSS layer is truncated by an abrupt erosional interface that separates it from the overlying GH3, forming a steep bank (Fig. 2d). In the north of the site this erosional contact is less pronounced, varying from clear to diffuse with distance from the spring centre. Here, a sample from the top of the LBCSS (Fig. 2e) produced an age of 311 ± 21 ka (MIS 9), indicating that GH4 is better preserved at the margins of the spring. Both GH5 and GH4 are hereafter referred to as the lower deposits (379 ± 26 to 311 ± 21 ka) and contain an assemblage of Acheulian artefacts (*n* = 465).

The upper deposit (GH1-3) contains an assemblage of MSA artefacts (*n* = 3018). At the base of the upper deposit is GH3, which consists of the orange to brown silty sand (OBSS) at the spring margin, dating to between 230 ± 18 ka at its base and 190 ± 17 ka at its top, indicating it most likely formed during MIS 7. GH3 also includes the dark organic silty sand (DOSS) layer towards the spring centre, with two samples yielding ages of 183 ± 13 ka and 179 ± 14 ka, indicating that deposition of GH3 continued through the MIS 7 − 6 transition. The DOSS layer contains abundant organic matter composed of highly fragmented and variably humified vegetal remains, however environmental conditions were evidently different to the DBBPS, with transport into the pan of partly humified and likely charred material that originated in areas subject to wildfires. Like DBBPS, pollen from this unit is mostly composed of wetland taxa, with an increase in monolete fern spores and Typhaceae pollen, as well as *Gunnera*, a large-leafed herb that occurs in marshy and aquatic environments. The presence of the conifer *Podocarpus*, found in the coastal Afrotemperate forests and montane areas of the Eastern Cape, as well as Ebenaceae, suggests a possible shift towards a more forested environment at this time.

At the centre of the spring, the top of the DOSS unit and the overlying GH2 and GH1 layers have been truncated by modern alteration of the landscape. They are preserved at the spring margins, however, where their sedimentary characteristics indicate limpid and oxygenated water had again filled the pan. Here, GH2 overlies both OBSS and DOSS layers (GH3) with an abrupt to clear contact. GH2 consists of the light pink to grey sandy silt (LPGSS) layer, with two samples providing ages of 153 ± 12 and 149 ± 13 ka (centred on MIS 6). A clear boundary separates this from GH1, the uppermost unit, which is made up of the light grey sandy silt (LGSS) layer and is dated to 95 ± 7 ka, during MIS 5. Thus, the upper deposits (GH1-3) formed over three main phases of spring activity between MIS 7 − 5 (230 ± 18 to 95 ± 7 ka).

### Archaeology

**Fig. 3:**
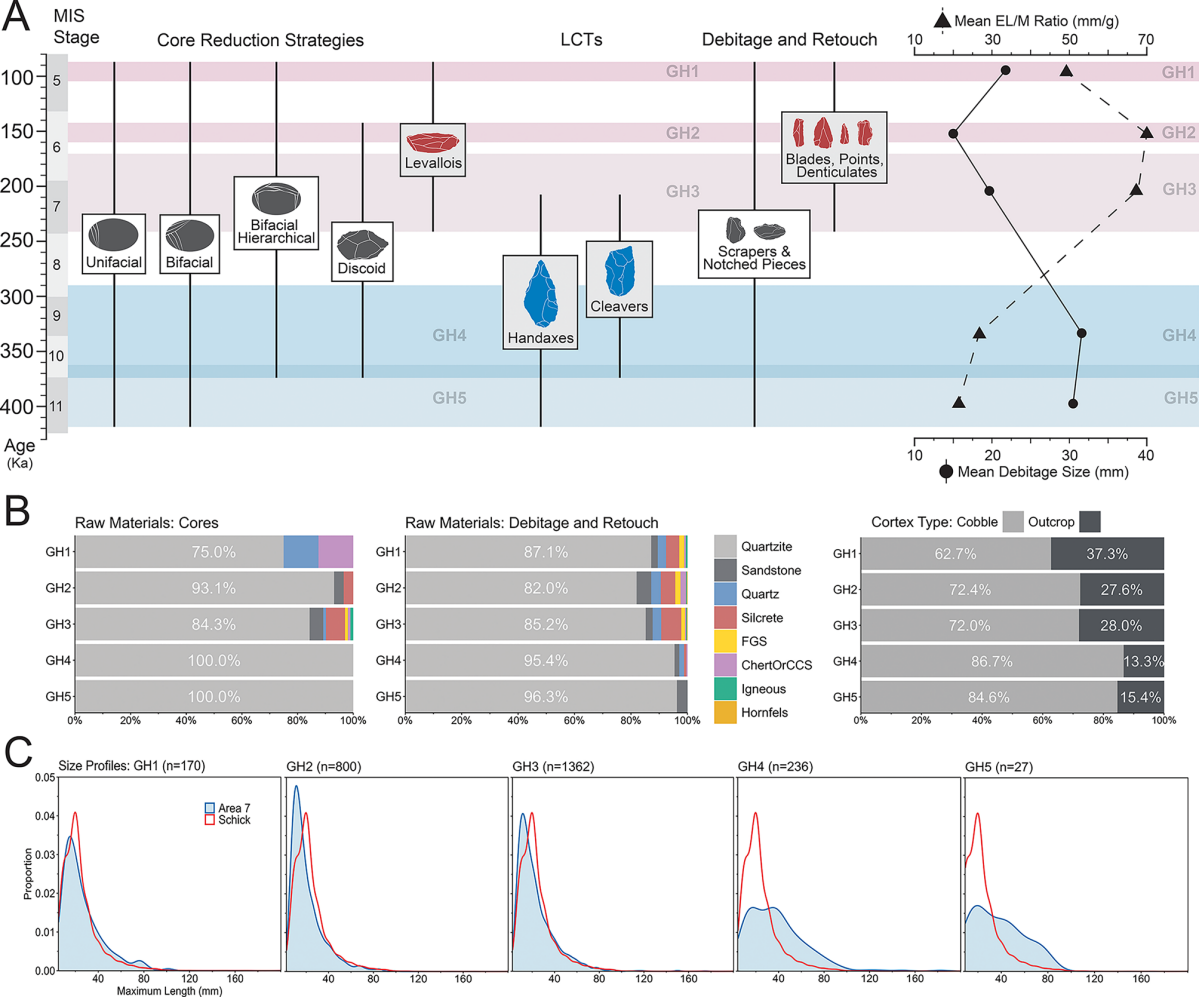
Technological continuity and change through time at Area 7. (**A**) Elements of technological continuity (grey) and change from the Acheulian (blue) to the MSA (red), with mean debitage size and edge length to mass (EL/M) ratios on the right. (**B**) Changes in raw material use and cortex types through time, and (C) density plots showing artefact size distributions compared with an experimental dataset^78^.

Multiple lines of evidence support the overall integrity of the site. Artefact size profiles show that the full range of size classes produced during stone tool manufacture are present in the upper deposit (GH1-3), closely matching density curves for experimental knapping assemblages^78^(Fig. 3). In contrast, small flaking debris is underrepresented in the lower deposit (GH4-5), similar to the pattern observed in the Acheulian layers at Areas 1 and 2^47,48^. Most artefacts are unweathered (64.5%), or only slightly (22.5%) to moderately (9.7%) weathered. Overall, the stratigraphic integrity of the deposit, confirmed by the luminescence chronology, size profiles, and artefact weathering patterns, indicates that the artefact assemblages are in a near-primary context.

In total, excavations at AMZ7 have produced 3483 stone tools (Table 2). Quartzite accounts for 86% of all artefacts at AMZ7, most of which were produced through on-site reduction of cobbles obtained from nearby secondary sources (Fig. 3, Supplementary Text 1.4). Changes in raw material use from the Acheulian to the MSA show that quartzite decreases from 96.4% to 84.4%, with greater use of silcrete and fine-grained siliceous lithologies in the MSA layers, while the use of quartzite sourced from outcrops increases from 13.4% to 28.5% (Fig. 3b). All cores from the Acheulian layers were made on quartzite, whereas cores were made on a broader range of raw materials in the MSA layers (Fig. 3b). These non-quartzite cores were transported to the site after initial reduction elsewhere, demonstrating that changes in patterns of landscape mobility and raw material provisioning had appeared in this region by ~ 230 ka.

**Table 2:** Stone tools from AMZ7, organised by technological group and category.

Group	Category	Layer					Number
		GH1	GH2	GH3	GH4	GH5	
Cores	Unifacial core	1	9	29	42	1	82
	Bifacial core	3	6	10	21	1	41
	Discoid		6	23	26		55
	Bifacial hierarchical core	1	1	9	5		16
	Levallois core	2	2	8			12
	Blade core		1	1			2
	Core on flake		2	5		1	8
	Core fragment	1	2	17	12	1	33
Complete flakes	Initial cortical flake	11	52	87	24		174
	Residual cortical flake	12	32	79	15	1	139
	Non-cortical flake	48	293	476	70	8	895
	Core maintenance flake	1	10	27	14	3	55
	Levallois flake	2	8	14			24
	Bipolar flake	1	3	7	3		14
Blades	Complete	7	10	9	1		27
	Proximal	1		2			3
	Medial	1	1	2			4
	Distal	1					1
	Split			1			1
Flake fragments	Fragment	47	199	431	38	3	718
	Split flake	10	87	117	23	3	240
	Core maintenance frag.		2	5			7
	Levallois flake frag.	4	16	21			41
Retouched pieces	Minimally retouched	2	1	5	2		10
	Laterally retouched	3	1	13	9	3	29
	End scraper		6	5	3		14
	Side scraper		2	2	2		6
	Other scraper types			7	2		9
	Simple notch	1	8	18	4	2	33
	Complex notch	1	3	12			16
	Notched denticulate	1	4	1			6
	Serrated denticulate		1				1
	Single awl	1	1				2
LCTs	Cleaver				4		4
	Handaxe			7	19	1	27
	Preform				11		11
	Fragment			2	9	1	12
Shatter	Cortical	3	47	95	5	2	152
	Non cortical	13	140	290	25	2	470
Hammerstones/cobbles	Hammerstone		6	13	14	1	34
	Hammerstone fragment				1		1
	Cobble fragment		1	2	2		5
	Unmodified cobble		4	20	24	1	49
**Total**		**179**	**967**	**1872**	**430**	**35**	**3483**

The lithic assemblages from the lower deposit (GH4-5) include cores (*n* = 110), LCTs (*n* = 45), debitage and debris, and retouched flakes (*n* = 27) (Fig. 3). The LCTs comprise both handaxes (*n* = 20; 87.5%) and cleavers (*n* = 4; 12.5%), and the presence of preforms and fragments suggests that production of these tools took place at or near the site. The unretouched debitage and high proportion of cores in the lower deposit also provide evidence for on-site reduction of quartzite cobbles (Figs. S32-33; Supplementary Text 5.4). Cores in the Acheulian layers are typologically diverse and generally exhibit short reduction chains, with unifacial and bifacial reduction strategies featuring the fewest flake scars per core (average flake scars: unifacial = 3, bifacial = 7). Discoidal cores (*n* = 26) are the second most common core type, and together with several hierarchically organised bifacial cores (*n* = 5) feature the longest reduction chains with an average of 11 flake scars. Platform preparation in the form of faceting is rare in the Acheulian layers (*n* = 2), although there is evidence for management of core detachment surfaces via the removal of core maintenance flakes (*n* = 14).

**Fig. 4:**
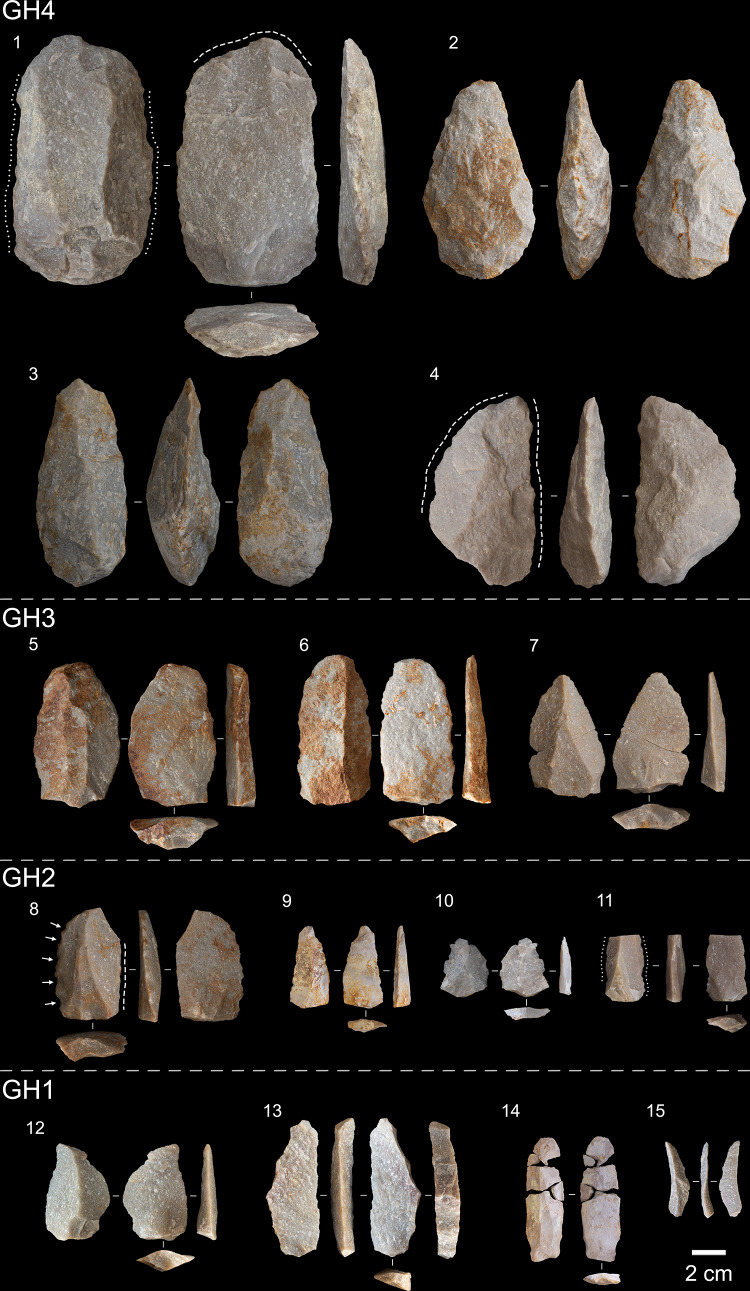
Acheulian to MSA artefacts from Area 7. 1) A large, retouched flake, 2–3) bifacial handaxes, and 4) bifacial knife from GH4. 5–7) Early MSA technology from GH3, showing unretouched convergent flakes with prepared platforms. 8) Denticulate, 9–10) blade and convergent flake, and 11) proximal blade with lateral retouch from GH2. 12) Convergent flake, and 13–15) blades from GH1. (Arrows: notches; dashed lines: feathered, parallel, or scalar retouch; dotted lines: mixed/minimal retouch).

The introduction of recurrent unidirectional, centripetal, and bidirectional methods of Levallois core reduction in GH3 signal the beginning of the MSA at AMZ7. Blades and elongated debitage are rare but increase in the upper deposits (*n* = 35), along with the production and use of parallel and convergent flakes. There are also changes in the way retouch was applied to flakes, with the introduction of denticulates (*n* = 7) and complex retouched notches (*n* = 16)^79^. Mean debitage size decreases in the MSA layers and there is a corresponding increase in edge length to mass ratios on complete flakes (Fig. 3a; Figs. S34-35; Supplementary Text 5.7). Prepared cores (*n* = 23), core maintenance flakes (*n* = 51), and faceted platforms on flakes (*n* = 118) all increase, indicating greater investment in the management and shaping of core detachment and platform surfaces.

Together, these elements of technological change reflect a broader shift in the composition of toolkits towards smaller, predetermined end-products (Fig. 4). However, they also represent a relatively small component of the early MSA assemblages at AMZ7, and examination of the cores reveals technological continuity with the preceding Acheulian, with most of the debitage produced by the same core reduction systems. The unifacial, bifacial, and discoidal cores, the main methods of flake production in the Acheulian layers, are the most common cores in GH2 and GH3, with no significant changes in their morphology or length of reduction chains detected (average flake scars: unifacial = 3, bifacial = 8, discoidal = 12) (Supplementary Text 5.6). In addition, several LCTs (*n* = 7) and two cleaver fragments were found in GH3, although unlike the lower deposit there is no other evidence for their manufacture in these layers, such as preforms.

The GH1 lithic assemblage (95 ± 7 ka), although small, shows several differences to the early MSA layers. Discoidal core reduction is absent and unifacial cores are less common, while the proportion of Levallois cores increases. Unlike the GH2-3 layers, the morphology of cores in GH1 differs to those in the Acheulian layers. These changes in assemblage composition are accompanied by proportionally more blades and Levallois end-products compared with the GH2-3 layers (Supplementary Text 5.3).

**Fig. 5:**
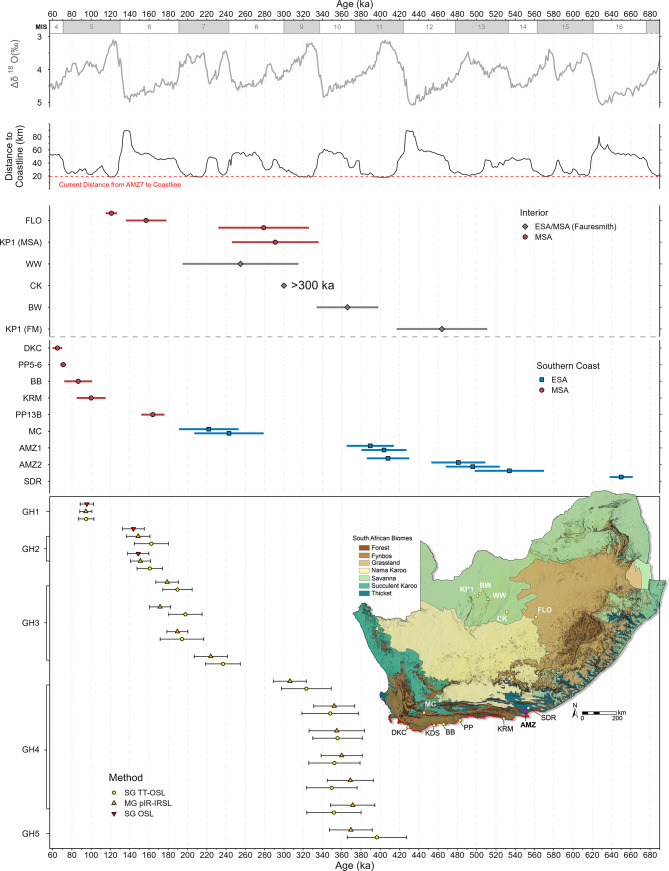
Area 7 chronology and key southern African ESA-MSA sites. From top: MIS and benthic δ18O (LR04 stack)^37^, and distance from AMZ7 to coastline modelled using relative sea levels^80,81^and bathymetry^82^. Published ages for key sites in the interior and southern coast of South Africa, and Area 7 luminescence chronology, showing pIR-IRSL, TT-OSL, and OSL ages, along with their 1σ uncertainty ranges. Map showing location of sites and vegetation biomes of South Africa^43^, produced using ArcGIS Pro 3.4 (esri.com) and the ALOS Global Digital Surface Model (JAXA).

## Discussion

The archaeological record of Africa’s southern coastal plain has featured prominently in discussions of complex social, symbolic, and technological behaviours that first appeared during the MSA^26–32^. However, little is known about the origins of the MSA in this region, and until recently there has been no chrono-cultural framework for the preceding local Acheulian^47,48,50^. Amanzi Springs, with its unique preservation conditions and site context represents the first stratified, open-air ESA-MSA archive to be documented in coastal South Africa. The MSA bearing 230 ± 18 ka layers at AMZ7 contain the oldest known MSA assemblage on the southern coast, extending the origins of the MSA in this region by ~ 95 − 36 ka.

The rarity of MSA sites dating to MIS 6 or earlier along this coastline may be partly due to erosion of low-lying deposits by sea-level high stands during MIS 11 and 5e, with large areas of once habitable land along the PAP now submerged^41,83^. Amanzi Springs, which sits at an elevation of 151 m, was not directly impacted by sea-level changes during the Middle to Late Pleistocene. Modelling indicates that sea-level high stands would have resulted in the expansion of estuarine habitats in Algoa Bay, while the distance to the coastline was largely unaffected owing to the local topography^47^. As sea levels retreated during glacial maxima and the PAP emerged, the coastline extended up to 90 km from the site (Fig. 5), expanding access to terrestrial resources within the diverse habitats that the PAP supported^41,84,85^, and potentially facilitating population connectivity along this coastal corridor with the interior of the continent^86^. The closing of the PAP during interglacial periods and the subsequent loss of habitat is likely to have disconnected populations, disrupting social structures and intensifying selection pressures on the groups that remained^41^, and may have promoted the development of adaptative subsistence and social behaviours^86^. The repeated occupation of Amanzi Springs during both interglacial and glacial periods, regardless of their distance to the coastline, suggests they represented a predictable, low-risk ecological setting with access to resources during prolonged periods of climatic flux.

Results from AMZ7 show that the Acheulian continued in this region until as late as 311 ± 21 ka, with the MSA appearing ~ 84 − 49 ka later along the southern coastal plain than in the interior. Together with ages of ~ 243 − 222 ka for the terminal Acheulian at Montagu Cave^50^, located at the western end of this Cape Fold Belt zone, our results support the late persistence of the Acheulian in both the Thicket and Fynbos biomes below the Great Escarpment. Despite overlapping chronologically with some of the earliest ESA-MSA transitional sites in the interior of South Africa (Fig. 5), there are no early examples of MSA-like technologies (i.e., blade and point production, or the Levallois method) in the Acheulian layers at Amanzi Springs Areas 1–2 (535 − 390 ka)^47,48^, Area 7 (379 − 311 ka), or Montagu Cave (~ 243 − 222 ka)^50^. In contrast, Levallois technology and blades appear by 464 ± 47 ka at Kathu Pan 1^20,21,87^, while in eastern Africa blades appear by ~ 543 − 509 ka and Levallois flake blanks were used for LCT production by ~ 400 ka at sites in the Kapthurin Formation in Kenya^13,18,19^. The presence of hierarchically organised cores in GH4, although not Levallois cores *sensu stricto*, does suggest a degree of technological innovation towards the end of the Acheulian in this region, however they do not appear to be linked with the production of LCTs, points, or blades. Moreover, the LCTs at both Montagu Cave and Amanzi Springs could be described as ‘classically’ Acheulian and are generally unstandardised compared with the late Acheulian in the interior^48,50,88^. The lack of transitional elements in the Acheulian in this region, despite being a feature of assemblages in the Savanna and Grassland Biomes of the Southern African Plateau at this time, suggests that populations living along the southern coast may have been isolated from those in the interior for at least the previous two glacial-interglacial cycles (MIS 12 − 9) and potentially longer.

It is not until after MIS 7 that the lithic assemblages at Amanzi Springs exhibit patterns that are consistent with the onset of the MSA, such as the introduction of Levallois reduction strategies, expanded raw material use, and increased investment in core preparation^5,11,15,89^. Published MSA sites that predate or are roughly contemporaneous with the GH3 layers include Jebel Irhoud in Morocco (315 ± 34 ka)^7^, Koimilot in the Kapthurin Formation, Kenya (~ 250 − 200 ka)^18^, KHS in Ethiopia (198 ± 14 ka)^90^, and Florisbad in central South Africa (~ 294 − 225 ka)^8,25^. These assemblages all have several elements in common, including a prepared core component, usually with both preferential and recurrent Levallois methods. Retouched and formal tools are relatively rare at early MSA sites, compared with the later MSA, but usually include both scrapers and retouched points^91^. Although the latter are absent from the MSA at AMZ7, Levallois cores were used to produce convergent flakes, and scrapers are among the most common retouched implements, along with notched pieces and denticulates. Another notable difference is that blades are rare at AMZ7 but are generally found in greater numbers at other early MSA sites. Nevertheless, the trend towards more efficient production of cutting edge per unit of mass, smaller elongated and convergent flakes, and more frequent platform faceting appear to be gradual but variable processes that continue throughout the MSA^92,93^. The lack of Levallois technology in the Acheulian layers at Amanzi Springs, and it’s appearance by ~ 230 ka, suggests that it was introduced, or independently invented, much later along the southern coast, after which it became a recurrent component of MSA assemblages from MIS 6 onwards^26,36^. In this manner, the Amanzi Springs sequence anticipates later MSA assemblages in this region at sites such as Pinnacle Point^26,27,36,93,94^, Klasies River Mouth^95–97^, and Blombos Cave^98,99^.

Although these technological changes may signal increased population connectivity with other regions of southern Africa, the evidence from AMZ7 does not reflect abrupt population replacement but rather a more gradual accumulation of innovative elements of lithic technology and a degree of continuity with the preceding local Acheulian. All core reduction strategies found in the Acheulian layers remained in use at the site until at least MIS 6, with the shape, size, and reduction intensity of these cores remaining relatively stable through time. Although this could be interpreted to suggest that methods of flake production developed directly from the Acheulian, it may also reflect continuity in site function and raw material constraints. Aside of the production and replacement of LCTs in the Acheulian layers, site-specific activities at Amanzi Springs were dominated by on-site reduction of locally sourced quartzite. Indeed, the low frequency of Levallois cores and debitage in the upper deposit may be due to the large quantities of debitage from the early stages of reduction of quartzite cobbles somewhat masking the Levallois component. This appears to be consistent with other sites in this region, where bifacial and discoidal cores are more common, raw materials are dominated by locally available rocks that were often reduced on-site to produce large amounts of morphologically variable debitage, and curated formal tools are relatively rare^50^. However, the presence of notched pieces, scrapers, and laterally retouched flakes throughout the sequence highlights the range of behaviours represented at Amanzi Springs and suggests that other subsistence tasks were likely undertaken at or near the site^48^. Site function is therefore unlikely to be sole explanation for this pattern.

The presence of LCTs in the upper deposit at AMZ7 may also indicate the continuity of technological systems during the early MSA. However, these LCTs fall within the size variation observed in the Acheulian layers and comprise only 0.3–0.7% of the GH3 assemblage, with no evidence they were being manufactured on site, such as preforms, or changes in production strategy. From a behavioural perspective, their presence may reflect a persistent functional need for heavy-duty tools at the site, even as the broader technological system appears to have shifted towards lighter, more mobile toolkits. Alternatively, they may represent the re-use of LCTs from surface contexts or exposed spring sediments. These artefacts are both durable and highly visible on the landscape due to their size and appearance, and the already established bifacial edge represents a potentially expedient source of flakes. In this scenario, the LCTs may represent an older technological signal incorporated into the GH3 layers during the earliest phases of formation. Given the continued and frequent nature of occupation at the spring, with each phase of spring formation having taken place over prolonged periods, we cannot at present rule this out. Taking the above into consideration, there is currently no direct behavioural or technological association between the LCTs and the more diagnostic MSA components of the GH3 assemblage (i.e., Levallois cores and debitage).

The co-occurrence of LCTs and Levallois cores, points, and blades is often attributed to ‘transitional’ industries, such as the Fauresmith, that are said to precede the onset of the MSA in the interior of South Africa^22,100,101,102^. However, robustly dated sites of this age are rare, and there are few detailed technological descriptions of these assemblages^22,102^. Consequently, the degree to which site function, site formation, and our frameworks for interpreting lithic variability have structured our understanding of these Middle Pleistocene contexts has received little attention. Retouched points and blades, innovative hunting technologies that first appear in southern Africa at Kathu Pan 1^20,21^, are considered diagnostic of the Fauresmith^100^. They are also a consistent component of other early MSA assemblages across Africa by MIS 6^91^. Despite the adaptative advantages they likely conveyed^11,87^, neither retouched points nor blade production are features of the Acheulian at Amanzi Springs. The lack of unifacial or bifacially retouched points, even after the introduction of Levallois cores and convergent flakes to the site, may again reflect site-specific activities. However, given the evidence for other subsistence tasks being undertaken at the site and as well as increasing landscape mobility, we would expect to see at least some evidence for these behaviours if they were a component of local technological systems. For these reasons, we refer to the period after ~ 230 ka at Amanzi Springs as the ‘early MSA’. Assigning the AMZ7 assemblages to a named stone tool industry such as the Fauresmith implies not only a high degree of cultural and technological connectivity with sites in the interior such as Kathu Pan 1^20^, Canteen Kopjie^100,103^, and Bestwood 1^104^, but also a behavioural association between the LCTs and more characteristically MSA elements of the assemblage that has so far not been demonstrated. Rather, the evidence from AMZ7 suggests a regionally distinct trajectory along the southern coast, and supports the argument that the more marginal environments found in the interior may have required novel technological adaptations to enable successful occupation of those regions^105^.

The mechanisms driving these patterns remain uncertain, however. One possible explanation is that the enduring behavioural stability of the Acheulian and the relatively late emergence of the MSA along this coastal corridor may be due to the Great Escarpment and Cape Fold Belt mountains acting as a biogeographic barrier, separating it from the interior during the earlier part of the Middle Pleistocene^86^. Together with the expansion and contraction of the Palaeo-Agulhas Plain in response to global eustatic changes, this may have contributed to the generation of population sub-structure and diversity between these regions, a scenario that has been inferred more broadly across Africa^1,4,106^. Although the role that environmental and climatic variation played in shaping hominin adaptative behavioural strategies remains poorly resolved, there is increasing evidence that emergence of the MSA was a long and complex process that involved many regions across the continent, with rates of technological and demographic change and cultural transmission being contingent on local conditions^5,11,105–108^. Recent fossil and genetic evidence supports a similar scenario with a complex evolutionary history for *H. sapiens* involving interactions between structured populations across the whole African continent, with deep ancestral structure shared by all modern humans prior to 1 Ma followed by the divergence of populations that coalesced again sometime after 300 ka^1, 3, 109–114^. In this model the more deep-rooted ancestral line only contributes about 20% to this later divergence event. Such a model could explain situations like we are suggesting for the southern coastal plain whereby interior and coastal populations evolved separately before coalescing again, resulting in the observed differences in the early MSA of this area. Although at present is not possible to confirm this, our results indicate there was degree of regional structure in the timing and nature of technological change between the Acheulian and early MSA in southern Africa. Determining the underlying mechanisms for this pattern, and the role of that local ecological parameters played in the emergence and spread of the MSA remain key challenges in the study of our evolutionary past.

## Materials and methods

### Excavation

Total Stations and photogrammetry were employed to document excavations at Area 7, using software and excavation protocols developed by Harold Dibble^115^and others^116–120^, and modified for recording an open-air site. Permanent control points were installed around the outer margins of the spring to establish a site grid using a differential GPS system (Leica GPS-1200), with corrected coordinates obtained in the WGS 1984 UTM zone 35S geographic coordinate system. Spatial data were recorded on total stations connected to Trimble Nomad hand-held computers using EDM-Mobile (oldstoneage.com) to store three-dimensional coordinates and contextual information. All excavated finds, stratigraphic boundaries, features, excavation outlines, specialist samples, and targets for geo-rectifying section photos and photogrammetry models were recorded to within the accuracy thresholds of the total station initialisations (< 5 mm residual error on each axis).

Excavations proceeded following identifiable natural stratigraphy. Stratigraphic layers were defined based on their sedimentary characteristics, including unifying sedimentary features such as colour, texture, and sedimentary components, and excavated separately. Excavated sediments were collected in buckets and dry sieved on site through 3 mm mesh screens, or collected in sample bags for sieving off site. An outline of the area excavated for each bucket was recorded so that any artefacts found in the sieves could be associated back to their original context with a reasonable degree of accuracy. Artefacts identified in the sieves were incorporated into the spatial database as aggregate ‘bucket’ samples and assigned to their corresponding stratigraphic layer. Bulk sediment samples were collected from each stratigraphic layer and stored for sedimentological analysis. Erosional/disturbance features were excavated and recorded separately and are not included in the artefact samples for each layer. All artefacts found during excavations were piece plotted with no size cutoff. All artefacts and other excavated finds were collected in sample bags and assigned a unique identifying number from a running sequence using barcode labels^117^. Curation of the Area 7 material will be undertaken at the Albany Museum, Makhanda in the Eastern Cape Province, South Africa.

### Geochronology

Luminescence dating was used to provide direct estimates of when the Acheulian- and MSA-bearing deposits of Area 7 were last exposed to light prior to burial. Fourteen luminescence dating samples were collected from GH1–GH5 during the 2018 and 2019 excavations and subsequently analysed at the University of Adelaide. The luminescence dating samples were taken from cleaned sedimentary exposures using metal or PVC tubes, with additional bulk sediment collected from the surrounding few cm of each tube for beta dose rate determination and water content analysis. Field gamma spectrometry measurements were performed at each luminescence dating sample position immediately after removal of the tubes. The luminescence dating approach adopted at Area 7 is based on that employed previously at Area 1 and Area 2, which has been described in detail^47,48^. Two semi-independent, extended-range luminescence dating techniques have been routinely applied to all samples, namely single-grain quartz thermally transferred optically stimulated luminescence (TT-OSL) and multi-grain K-feldspar post-infrared infrared stimulated luminescence (pIR-IRSL) dating^121–123^. These luminescence signals exhibit considerably higher dose saturation properties than conventional quartz OSL dating and offer the potential to establish finite and reliable depositional chronologies over the Middle Pleistocene timescales of interest for Area 7^124–132^. Traditional single-grain quartz OSL dating has additionally been applied to samples ASP19-6, ASP18-7 and ASP19-14 from the uppermost layers of Area 7 (LGSS and LPGSS) as initial TT-OSL D_e_ measurements indicated that these deposits were notably younger than the underlying Acheulian-bearing layers. The combined TT-OSL, pIR-IRSL and OSL dating approach employed in this study enables us to cross-check the reliability of the luminescence chronologies for Area 7 using multiple mineral fractions (quartz versus K-feldspar) and at different scales of equivalent dose (D_e_) analyses (multiple-grain versus single-grain).

Full discussions of the TT-OSL, OSL and pIR-IRSL D_e_ distributions and statistical age models used to derive representative burial dose estimates for each sample are provided in the Supplementary Text 3. In general, the single-grain OSL, single-grain TT-OSL and pIR-IRSL D_e_ distributions exhibit limited scatter and are characteristic of well-bleached, unmixed samples^133–137^, hence we have used the weighted mean D_e_ values (calculated using the central age model of Galbraith et al.^77^) to derive final burial dose estimates (Table 1). The only exceptions are the single-grain TT-OSL D_e_ datasets of samples ASP18-7 and ASP18-9, which exhibit more pronounced scatter and enhanced tails of high D_e_ values. These D_e_ characteristics are interpreted as reflecting the presence of heterogeneously bleached grain populations, which is consistent with the relatively slow bleaching characteristics of TT-OSL signals^138,139^, and the potential for insufficient bleaching of residual doses in spring-margin or subaqueous pond settings that may have experienced limited, indirect or filtered daylight conditions. For these two samples, the final TT-OSL ages have been derived using the minimum age model of Galbraith et al.^77^ (Table 1) in order to isolate burial dose estimates from the well-bleached portion of grains in each D_e_ dataset^124,133,140^.

### Soil micromorphology

Samples were taken in 2017 and 2018 for thin-sectioning and micromorphological study. Before collecting the samples, the stratigraphic succession exposed on excavation profiles was described following^141^and documented by high-resolution photographs. Undisturbed sediment monoliths were collected from the Sector 1 north profile (seven samples), and Sector 4 north profile (three samples). A sample was collected from each lithostratigraphic unit and across all major stratigraphic interfaces and sedimentary features of interest, with the aim of investigating the microstratigraphic context, formation processes, post-depositional alteration, and stratigraphic boundaries.

The monoliths were carved by hand and trowel out of excavation profiles, wrapped in cellulose paper for protection and sealed by paper adhesive tape. In the laboratory, the samples were air-dried in ventilated oven at 35 °C for one week and subsequently impregnated with low-concentration acetone-dissolved polyester resin under moderate vacuum. Polymerisation was fostered by heating at 40 °C for about 30 days, then the resin was left to harden for 3 months. The impregnated monoliths were cut by diamond saw, glued on 90 × 60 mm microscope slides, reduced to about 0.5 mm again by diamond saw, and finally ground to 30 μm by corundum and aluminium oxide powder using petroleum as coolant. The slides were finally covered by standard microscopy protection glass. The thin sections were examined using a Zeiss Axio Scope.A1 standard petrographic microscope under 2.5x, 10x, 20x and 50x magnification. Thin section descriptions follow the standard criteria proposed by^142^. The results of thin-section analysis are presented in the Supplementary Text 2.2.

### Pollen and palaeoecology

Pollen and microcharcoal analyses were conducted on sediment samples collected from the excavation profile walls as columns. Columns were taken from the Sector 1, 2, and 4 north profile walls from the base to the top of the sections, with two duplicate side by side columns sampled in 2 cm increments for high resolution analyses and one bulk column sampled in 20 cm increments. Extraction of palynomorphs from these samples followed dense-media separation procedures adapted from Campbell et al.^143^. *Lycopodium*—exotic marker spore—tablets from Lund University (batch #010922211 and #140119321) were added to the samples to allow estimation of absolute pollen and microcharcoal concentration^144^. Samples were dispersed with sodium pyrophosphate before being sieved through 212 μm and 106 μm sieve meshes to remove coarse mineral sediment, plant debris, and macrofossils. The remaining sediment was treated with 7% HCl to remove carbonates and 10% NaOH to remove humic acids. Heavy liquid mineral separation using sodium polytungstate (SPT) prepared to a specific gravity of 1.95 g cm − 3 was done to separate the pollen and microcharcoal grains from the siliceous mineral fraction. Samples were then acetolysed with a 9:1 ratio of (CH_3_CO)_2_O and H_2_SO_4_. Samples were mounted in glycerin and pollen was counted to 400–700 grains per sample using a Zeiss Axio Lab.A1 microscope at 400x and 1000x magnification for identification. Palynomorph identifications were made using The University of Cape Town pollen reference collection, the references of van Zinderen Bakker^145–148^, and Scott^149^. Spores (monoletes and triletes) were plotted along with pollen but are not included in the total pollen sum/pollen absolute abundance used to generate the percentage relative abundances.

### Mineral sediment particle size

Approximately 1 cc of sediment was subsampled from the column samples and treated with 10% HCl to remove carbonates and 30% H_2_O_2_ to oxidise any organic fraction. Samples were agitated in 10% sodium hexametaphosphate for a minimum of two hours to disperse and deflocculate particles. Grain size was measured on a Malvern Mastersizer 2000 with at a maximum obscuration tolerance of 20%. Output data was processed in Gradistat v9.1^150^.

### Lithic analysis

A technological attribute approach was used to analyse the Area 7 lithic assemblage, incorporating definitions and attributes from Wilkins et al.^93^, Shea^151,152^, Sánchez-Yustos et al.^153^, Soriano et al.^154^, Braun et al.^155^, Holdaway and Stern^156^, Andrefski^157^, Debénath and Dibble^158^, Dibble^159^, Boëda^160^, and de la Torre^161^. All lithics that were piece-plotted from secure stratigraphic contexts were measured, weighed, and relevant technological attributes recorded for each specific lithic type. Artefacts were categorised into debitage and tools (complete flakes, flake fragments, blades, and retouched pieces), cores, LCTs, and other pieces (hammerstones, shatter, and cobble fragments). Artefact attributes and measurements were recorded using E4 (oldstoneage.com) for data-entry, and Microsoft Access to store the lithic database. Edge-length / mass (EL/M) ratios of complete flakes were calculated using the technological length, maximum dimension, and maximum width measurements following^92,162,163^.

### Statistical analysis

Statistical analyses were conducted using R statistical software^164^to facilitate the reproducibility of this study. The code used for the statistical procedures and to produce the plots is provided with the supplementary data. Metric attributes were first examined using contingency tables, with Shapiro-Wilk tests and histograms used to test the relevant variables for normality of distribution. Continuous variables were compared using independent-samples t-tests for normally distributed data, and non-parametric Mann-Whitney U tests for non-normally distributed data. MANOVA tests were used to examine categorical variables against multiple independent variables. Confidence intervals (at the 95% level) of means were plotted using error bars. Categorical attributes were compared using chi-square tests of independence. Rather than report ‘statistical significance’, we follow the approach advocated by^165^ for reporting results where a p value is used; p values are reported for each test, with those between 0.05 − 0.01 considered as moderate evidence, 0.01–0.001 as strong evidence, and < 0.001 as very strong evidence.

## Supplementary Information

Below is the link to the electronic supplementary material.

Supplementary Material 1

## Data Availability

Raw data and R code are available in the supplementary data.
