# Supplementary material for: Regional variability in the Acheulian to Middle Stone Age transition in southern Africa
Source: Sci Rep. 2026 Mar 19;16:9529. doi: 10.1038/s41598-026-40075-8 (PMC13004965; doi:10.1038/s41598-026-40075-8)
Supplement: Supplementary file 1 — Supplementary Material 1 [file 41598_2026_40075_MOESM1_ESM.docx]

**Supplementary Information**

Regional Variability in the Acheulian to Middle Stone Age Transition in Southern Africa

Blackwood, A.F.*, Wilkins, J., Arnold, L.J., Demuro, M., Boschian, G., Caruana, M.V., Lalunio, E.F., Spate, M., Hatton, A., Muir, R.A., Wilson, C.G., Quick, L.J., Meredith-Williams, M., Herries, A.I.R.

**This PDF file includes:**

Supplementary Text

Figs. S1 to S36

Tables S1 to S15

Supplementary References

**Supplementary Text**

[1. Background Information 4](#_Toc221197639)

[1.1 Amanzi Springs 4](#_Toc221197640)

[1.2 Geological background 4](#_Toc221197641)

[1.3. Regional Hydrology 8](#_Toc221197642)

[1.4. Local and Regional Raw Material Sources 11](#_Toc221197643)

[1.5 Amanzi Springs Area 7 14](#_Toc221197644)

[2. Geoarchaeology and Site Formation 18](#_Toc221197645)

[2.1 Sedimentology and Site Formation 18](#_Toc221197646)

[2.2 Micromorphology and Particle Size Analysis Summary 26](#_Toc221197647)

[2.3 Site Formation Summary 35](#_Toc221197648)

[3. Luminescence Dating 36](#_Toc221197649)

[3.1 Sample information and Detailed Luminescence Methods 36](#_Toc221197650)

[3.2 Equivalent dose (D_e_) determination 40](#_Toc221197651)

[3.3 Single aliquot regenerative dose (SAR) D_e_ validation tests 43](#_Toc221197652)

[3.4 pIR-IRSL anomalous fading tests 45](#_Toc221197653)

[3.5 D_e_ results and ages 46](#_Toc221197654)

[4. Palaeoecology 62](#_Toc221197655)

[4.1 Previous Palaeoenvironmental Research at Amanzi Springs 62](#_Toc221197656)

[4.2 Area 7 Pollen Analysis 65](#_Toc221197657)

[5. Archaeological Data 69](#_Toc221197658)

[5.1 Introduction 69](#_Toc221197659)

[5.2 Raw Materials 71](#_Toc221197660)

[5.3 The Area 7 Lithic Assemblages 75](#_Toc221197661)

[5.4 Platforms 87](#_Toc221197662)

[5.5 Retouch 87](#_Toc221197663)

[5.6 Cores 89](#_Toc221197664)

[5.7 Evidence for On-site Reduction 92](#_Toc221197665)

[5.8 Edge length / mass (EL/M) Ratios 95](#_Toc221197666)

[5.9 Artefact Weathering Patterns 97](#_Toc221197667)

[5.10 Artefact Size Distribution 99](#_Toc221197668)

[5.11 Site Function 103](#_Toc221197669)

[Supplementary References 103](#_Toc221197670)

**Supplementary Figures**

[Fig. S1. Geology of the Algoa Bay region. 6](#_Toc221197671)

[Fig. S2. Geology and topography of the study area 8](#_Toc221197672)

[Fig. S3. Hydrogeological map of the study area 10](#_Toc221197673)

[Fig. S4. Primary and secondary raw material sources 12](#_Toc221197674)

[Fig. S5. Primary raw material sources close to Amanzi Springs 13](#_Toc221197675)

[Fig. S6. Cobble bedload in the Coega River 14](#_Toc221197676)

[Fig. S7. Map of the Amanzi Springs complex 16](#_Toc221197677)

[Fig. S8. Aerial view of Amanzi Springs Area 7 17](#_Toc221197678)

[Fig. S9. Excavations at the Area 7 spring 19](#_Toc221197679)

[Fig. S10. Stratigraphy of the Area 7 spring 25](#_Toc221197680)

[Fig. S11. Photomicrographs of Area 7 thin sections 28](#_Toc221197681)

[Fig. S12. Particle size distribution for layers GBSS, OBSS, LPGSS, and LGSS 29](#_Toc221197682)

[Fig. S13. Photomicrographs of Area 7 thin sections 32](#_Toc221197683)

[Fig. S14. Particle size analysis of samples from the DBBPS 33](#_Toc221197684)

[Fig. S15. Photomicrographs of Area 7 thin sections 34](#_Toc221197685)

[Fig. S16. Particle size analysis for samples from LBCSS and DOSS 35](#_Toc221197686)

[Fig. S17. Representative K-feldspar pIR-IRSL250, single-grain quartz OSL and single-grain quartz TT-OSL decay / dose-response curves 50](#_Toc221197687)

[Fig. S18. Dose-recovery test results for samples ASP18-9, ASP18-12 and ASP19-6. 51](#_Toc221197688)

[Fig. S19. Single-grain TT-OSL De distributions (left-hand plots) and multi-grain pIR-IRSL De distributions (right-hand plots) 56](#_Toc221197689)

[Fig. S20. Single-grain OSL De distributions 57](#_Toc221197690)

[Fig. S21. Relative percentage pollen and non-pollen palynomorphs diagram 67](#_Toc221197691)

[Fig. S22. Pollen and non-pollen palynomorph grains 67](#_Toc221197692)

[Fig. S23. Microscopic charcoal fragments 68](#_Toc221197693)

[Fig. S24. Summary of the Area 7 assemblages 78](#_Toc221197694)

[Fig. S25. Area 7 northern and western profile views 80](#_Toc221197695)

[Fig. S26. Area 7 northern and western profile views 81](#_Toc221197696)

[Fig. S27. Area 7 northern and western profile views 82](#_Toc221197697)

[Fig. S28. Prepared cores from the upper deposit at Area 7. 83](#_Toc221197698)

[Fig. S29. Bifacial hierarchical cores from the lower deposit at Area 7 84](#_Toc221197699)

[Fig. S30. Debitage and retouch from the Area 7 sequence 85](#_Toc221197700)

[Fig. S31. Large cutting tools from Area 7 86](#_Toc221197701)

[Fig. S32. Evidence for on-site reduction 92](#_Toc221197702)

[Fig. S33. Cortical vs non cortical complete flakes, blades, and retouched pieces 94](#_Toc221197703)

[Fig. S34. Edge length to mass (EL/M) ratio of complete unretouched flakes 96](#_Toc221197704)

[Fig. S35. Confidence intervals for EL/M ratios of complete flakes, blades, and retouched pieces >20 mm 97](#_Toc221197705)

[Fig. S36. Density plots showing artefact size distributions 102](#_Toc221197706)

**Supplementary Tables**

[Table S1. 7](#_Toc221197707)

[Table S2. 58](#_Toc221197708)

[Table S3. 59](#_Toc221197709)

[Table S3. Continued. 60](#_Toc221197710)

[Table S4. 61](#_Toc221197711)

[Table S5. 61](#_Toc221197712)

[Table S6. 64](#_Toc221197713)

[Table S7. 69](#_Toc221197714)

[Table S8. 70](#_Toc221197715)

[Table S9. 73](#_Toc221197716)

[Table S10. 74](#_Toc221197717)

[Table S11. 78](#_Toc221197718)

[Table S12. 79](#_Toc221197719)

[Table S13 88](#_Toc221197720)

[Table S14 89](#_Toc221197721)

[Table S15. 90](#_Toc221197722)

1. Background Information

1.1 Amanzi Springs

Amanzi Springs is a complex of 12 separate springs clustered around the northern slope of a low-rising hill above the terraced coastal plains of Algoa Bay (Fig. S1; Table S1). Two of the springs, Areas 1 and 2, were first excavated in the 1960s by Inskeep ^1^ and Deacon ^2,3^, who documented stratified Acheulian assemblages along with preserved wood and botanical remains within the spring deposits. Such preservation conditions are exceptionally rare in Acheulian contexts, and the only other site in Africa where similar conditions are reported is Kalambo Falls in Zambia (499-453 ka) ^4–7^. At the time of the original excavations at Amanzi Springs, radiometric dating techniques were in their infancy and the only ages obtained were radiocarbon dates on fragments of wood that suggested a Late Pleistocene age ^2^. Despite the apparently young age of the springs, Deacon attributed the assemblage, which included a large collection of LCTs, to the late Acheulian on typological grounds. Recent research at Amanzi Springs has established a robust chronology for Areas 1 and 2 using multiple luminescence methods, with results demonstrating that the springs were repeatedly occupied between ~534-390 ka ^8,9^. The age of the occupations at Areas 1 and 2 thus confirms Deacon’s ^2^ original hypothesis, and reanalysis of the artefact collections indicate that the springs likely functioned as a workshop locality, with high frequencies of LCTs and cores all made from locally available quartzite raw materials.

The following sections provide additional information on the geological and hydrological background of Amanzi Springs, the Area 7 stratigraphic sequence, site formation, palaeoecology, and the artefact assemblage collected during excavations at the site.

1.2 Geological background

Erosion-resilient quartzitic sandstones of the Ordivician to Silurian Table Mountain Group (TMG) dominate the elevated regions of the southeastern Cape including the Groot Winterhoekberge and several isolated ‘koppies’ (low rising hills) northwest and south of Amanzi Springs (Fig. S1-S2; Table S1). This highly deformed, fractured rock, especially its Peninsula Formation ^10^, is an important unit controlling deep regional groundwater movement in the Cape and accounts for several springs in the area including those at Amanzi Springs ^11–14^. The TMG is also the primary source for the abundant quartzitic boulders, cobbles and pebbles found in the bedload of local rivers, on deflated land surfaces, and in variably consolidated Mesozoic and Cenozoic conglomerates/gravels that occur in the local vicinity.

The less resistant conglomerates, sandstones and mudstones of the Jurassic – Cretaceous Uitenhage Group occupy most of the low-lying areas north, south and east of Amanzi Springs and outcrop in the fault-bounded Algoa Basin, which formed during the Mesozoic breakup of Gondwana. The oldest unit in the Uitenhage Group is the Jurassic Enon Formation, which locally only outcrops in a small ravine ~10 km west of Amanzi Springs and is composed of massive to poorly stratified conglomerates deposited in alluvial fans derived from primarily the TMG and is commonly stained by iron-oxides giving it an orange-red appearance ^15^. Overlying the Enon Formation, and laterally grading into it, is the Jurassic – Cretaceous fluvio-lacustrine Kirkwood Formation, which consists of varicoloured red-mottled mudstones, beige sandstones and subordinate conglomerates ^16^ that can be found widely in regions north of the Coega Fault including the immediate vicinity of Amanzi Springs. The overlying Lower Cretaceous shallow marine Sundays River Formation is a mudstone-dominated unit with intermittent sandstones, albeit predominantly green-grey in colour ^17^. It outcrops south of the Coega Fault (Fig. S1-S2) ~2.8 km south of Amanzi Springs, and northeast of the Coega River.

Several isolated outcrops of Late Cretaceous – Cenozoic deposits are scattered around the Uitenhage District that would once have held a wide distribution but have since been eroded and dissected during intensive denudation driven by changes in relative sea level. One such unit is the erosion-resistant silcrete-rich Grahamstown Formation, which forms a number of isolated peneplains in the otherwise rugged topography of the Winterhoekberge west of Amanzi Springs ^17^. The calcareous Miocene – Pliocene Alexandria and Nanaga formations outcrop on wave-cut platforms northwest of Amanzi Springs with the former also capping several hills to the south ^18,19^. Moderately to poorly consolidated Cenozoic terrace gravel deposits occur in the Coega River valley and are related to periods of elevated discharge and energy of deposition and/or base-level change. Given that these deposits are related to existing drainage, modern day alluvial deposits closely resemble and are in places lithologically indistinguishable from previously deposited terrace gravels. Rare but important units in the region are the Cenozoic silcrete-ferricretes that are associated with small TMG ‘koppies’ that protrude through thin Uitenhage Group deposits. These mixed siliciclastic/chemical precipitates relate to groundwater movement in artesian TMG aquifers and probably resemble palaeo-spring activity. However, the spring eye deposits at Amanzi Springs, which include the same ferruginised precipitates along with sand, silt and clay, are classified as a separate stratigraphic unit named the Amanzi Formation ^2^ after recognition of their archaeological importance.


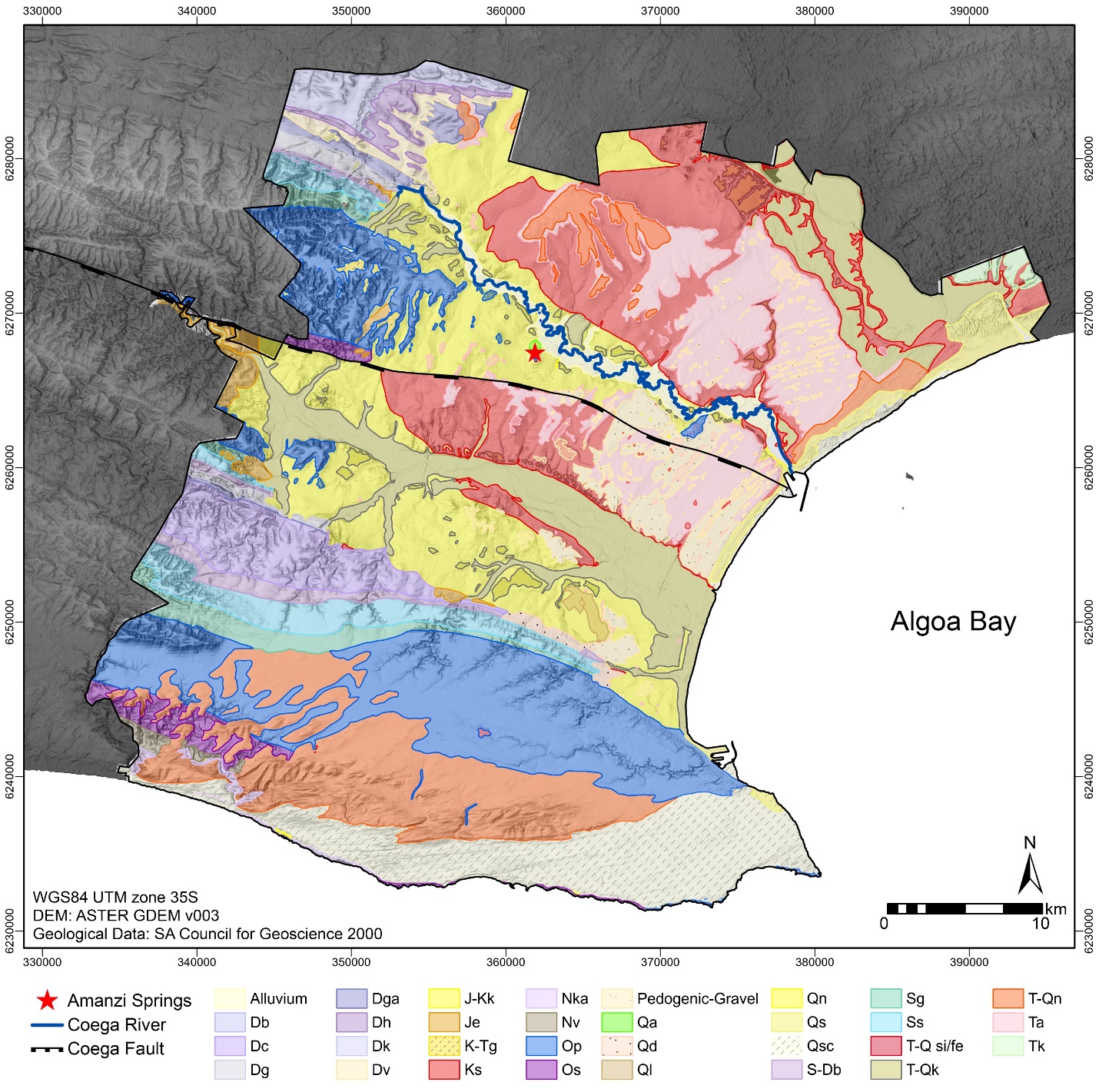


Fig. S1. Geology of the Algoa Bay region. Geological data from the South African Council for Geosciences ^20^ 1:50k series (maps 3325CB, 3325DA, 3325CD & 3425AB, 3325DC & 3425BA). See Table S1 for details on each formation. Figure produced using ASTER GDEM v003 ^134^ and ArcGIS Pro 3.4 (esri.com).

Table S1. Geological formations of the Algoa Bay region ^20^. TMG: Table Mountain Group

| **Lithology** | **Formation** | **Group** | **Age** | **Description** |
| --- | --- | --- | --- | --- |
| Tk | Kinklebos | - | Quaternary | Silt and fine-grained sand |
| Alluvium | - | - | Quaternary | Coega River Valley Alluvium |
| Qsc | Schelm Hoek | Algoa | Holocene | Aeolian sand, soil horizons, middens |
| Qa | Amanzi | Algoa | Quaternary | Amanzi Formation, sand, silt and clay |
| Ql | Nahoon | Algoa | Pleistocene | Lacustrine silt and mud |
| Qn | Nahoon | Algoa | Pleistocene | Aeolianite (calcareous sandstone), palaeosols and subordinate calcrete |
| Qs | Salnova | Algoa | Pleistocene | Marine/estuarine calcareous sand/sandstone, silt, siltstone, gravel/conglomerate, shelly limestone |
| T-Q si/fe | Salnova | Algoa | Pleistocene | Silcrete (Pedogenic Residue) |
| Ped. Gravel | Alexandria | Algoa | Quaternary | Weathered Alexandria Formation |
| Qd | Damascus | Algoa | Quaternary | Scree |
| T-Qk | Nanaga | Algoa | Quaternary | Fluvial terrace gravel, sand, silt |
| T-Qn | Nanaga | Algoa | Pliocene | Aeolianite/calcareous sandstone/sand |
| Ta | Alexandria | Algoa | Miocene | Calcareous marine-estuarine-lagoonal sandstone, conglomerate, conquinite |
| K-Tg | Grahamstown | - | Tertiary | Grahamstown Formation Silcrete on African Land Surface |
| J-Kk | Kirkwood | Uitenhage | Cretaceous | Kirkwood Formation, reddish and greenish mudstone, sandstone, conglomerate |
| Ks | Sundays River | Uitenhage | Cretaceous | Grey mudstone, siltstone, sandstone |
| Je | Enon | Uitenhage | Jurassic | Conglomerate, subordinate sandstone and mudstone |
| Db | Boplaas | Bokkeveld | Devonian | Sandstone and agrillaceous sandstone |
| Dc | Ceres | Bokkeveld | Devonian | Undifferentiated Shale, siltstone and sandstone |
| Dg | Gydo | Bokkeveld | Devonian | Black shale, subordinate siltstone, fossiliferous |
| Dga | Gamka | Bokkeveld | Devonian | Feldspathic sandstone, fossiliferous |
| Dh | Hex River | Bokkeveld | Devonian | Dark grey impure sandstone, fossiliferous |
| Dk | Karies | Bokkeveld | Devonian | Shale, silty at base, discontinuous impure sandstone |
| Dv | Voorstehoek | Bokkeveld | Devonian | Shale, siltstone and subordinate sandstone, fossiliferous |
| S-Db | Baviaanskloof | TMG | Silurian | Impure feldspathic sandstone, subordinate shale |
| Sg | Goudini | TMG | Silurian | Brown weathering quartzitic sandstone |
| Ss | Skurweberg | TMG | Silurian | Quartzitic sandstone, profusely cross-bedded |
| Op | Peninsula | TMG | Ordovician | Quartzitic sandstone |
| Os | Sardinia Bay | TMG | Ordovician | Cross-bedded white quartzite, ripple-bedded "dirty" sandstone, minor phylite, conglomerate lenses |
| Nka | Kleinrivier | Gamtoos | Namibian | Limestone, calcareous phyllite and sandstone, gritstone |
| Nv | Van Stadens | Gamtoos | Namibian | Quartzite, conglomerate, subordinate phyllite |


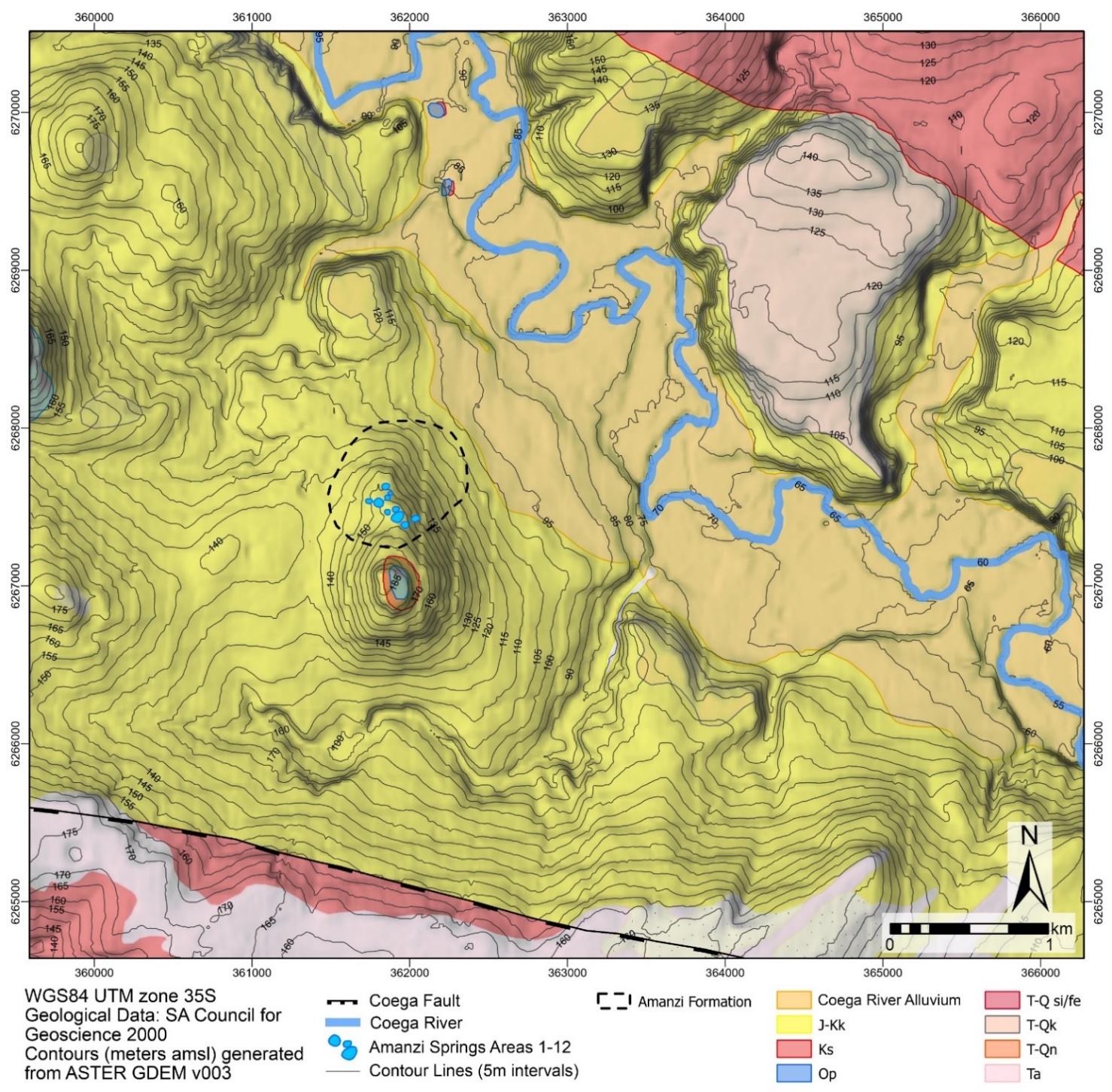


Fig. S2. Geology and topography of the study area showing location of Amanzi Springs in relation to the Coega River and Coega Fault. Figure produced using ASTER GDEM v003 ^134^ and ArcGIS Pro 3.4 (esri.com).

1.3. Regional Hydrology

The springs at Amanzi are fed by the Coega Ridge Aquifer (CRA), a unit of the Uitenhage Artesian Basin (UAB), which is the largest artesian groundwater basin in South Africa ^14^. One of the largest free flowing artesian aquifers in South Africa, it was an important source of irrigation water during the first half of the 20th century, until over-extraction greatly reduced the productivity of groundwater boreholes in the region ^14^. Covering an area of ~3700 km, the UAB is recharged by a large rainfall catchment in the Groot Winterhoek, Elands, and Zunga Berge mountain ranges to the west of Amanzi Springs (Fig. S3). The UAB is divided by the Coega Fault into two aquifer systems. To the north the CRA forms a confined artesian to sub-artesian aquifer, and to the south of the Coega Fault is the Swartkops Aquifer Unit (SAU), a semi-confined to unconfined alluvial aquifer ^14^. The flow of groundwater in the CRA travels in an east-southeast direction below the confining sediments of Uitenhage Group, where it discharges into Algoa Bay ^21^. The Uitenhage Group sediments are notable for their very low porosity and permeability, forming an aquiclude where they overlie the fractured TMG ^21^. The spring water at Amanzi is described as ‘deep-seated’ with a circulation depth estimated to be ~555m and a temperature of 33°C ^14^. The deep origin of spring water at Amanzi is likely to have resulted in a strong and uniform flow during periods of spring activity, and flow rates are thought to have been less seasonally dependent than other springs in the area. Cumulative departure from normal rainfall rates do exert influence on groundwater flow in the UAB system, however, and it is likely that long-term rainfall cycles, coupled with changes in relative sea level, played a major role in free-flowing spring activity in the CRA ^21^.

The springs at Amanzi follow a curving line around the side of Amanzi Hill, with a roughly NW-SE strike, in a broadly similar direction to the Coega Fault (Fig. S2). The shape, relatively shallow depth, and location of the artesian springs in this region are largely controlled by structural trends of the Cape Fold Belt imposed during the Cape Orogeny. In the southeastern Cape these take on a WNW – ESE orientation and while the folding produced cleavages and joints in the TMG that introduced secondary permeability, and planes of weakness introduced by thrusting were reactivated during Mesozoic inversion as the extensional Coega Fault. On the downthrown southern side of the Coega Fault, the TMG is buried beneath a thick overburden of the impermeable Uitenhage Group strata, whereas its northern section is relatively shallow and in places the TMG protrudes through what is only a thin veneer of the Uitenage Group to form isolated inliers. Several of such TMG exposures exist west and south of Amanzi Springs and are important points of artesian discharge after meteoric waters from the highlands infiltrate and flow down, eastward through the aquifer beneath the Uitenhage Group to come to surface where the TMG outcrops. Elsewhere however, away from the TMG exposures, groundwater is confined by the Uitenhage Group aquiclude and does not come to the surface. A groundwater borehole at Amanzi Springs (borehole 3325DA00002 in the National Groundwater Archives) extends 65 m yet passes through the entirety of the thin Uitenhage Group and into the TMG, confirming the near-surface expression of the TMG near the spring eyes. The significant exposures of silcrete/ferricrete at the TMG outcrop atop the hill south of Amanzi Springs indicate the existence of once-springing waters here. It is plausible that once the preferred site of springing had become clogged by chemical precipitates, groundwater forcibly penetrated the thin veneer of impermeable Uitenhage Group at Amanzi Springs aided by artesian pressure. Furthermore, the development of the Coega Fault may have enhanced the permeability of surrounding TMG by increasing the density and connectivity of inherited compressive fractures. For instance, the highly brecciated TMG outcrops observed at the Coega Kop quarry to the east of Amanzi Springs, a widening of the fault to at least 50 m at the Groendal Dam, and the presence of numerous smaller faults intersected in boreholes near the Coega Fault indicate that displacement generated fractures that extend well beyond the mapped fault plane ^21^. Highly fractured TMG is also present on a low rising hill approximately 2 km west-south-west of Amanzi Springs, and presumably beneath the spring eyes at Amanzi, facilitating elevated groundwater flow.


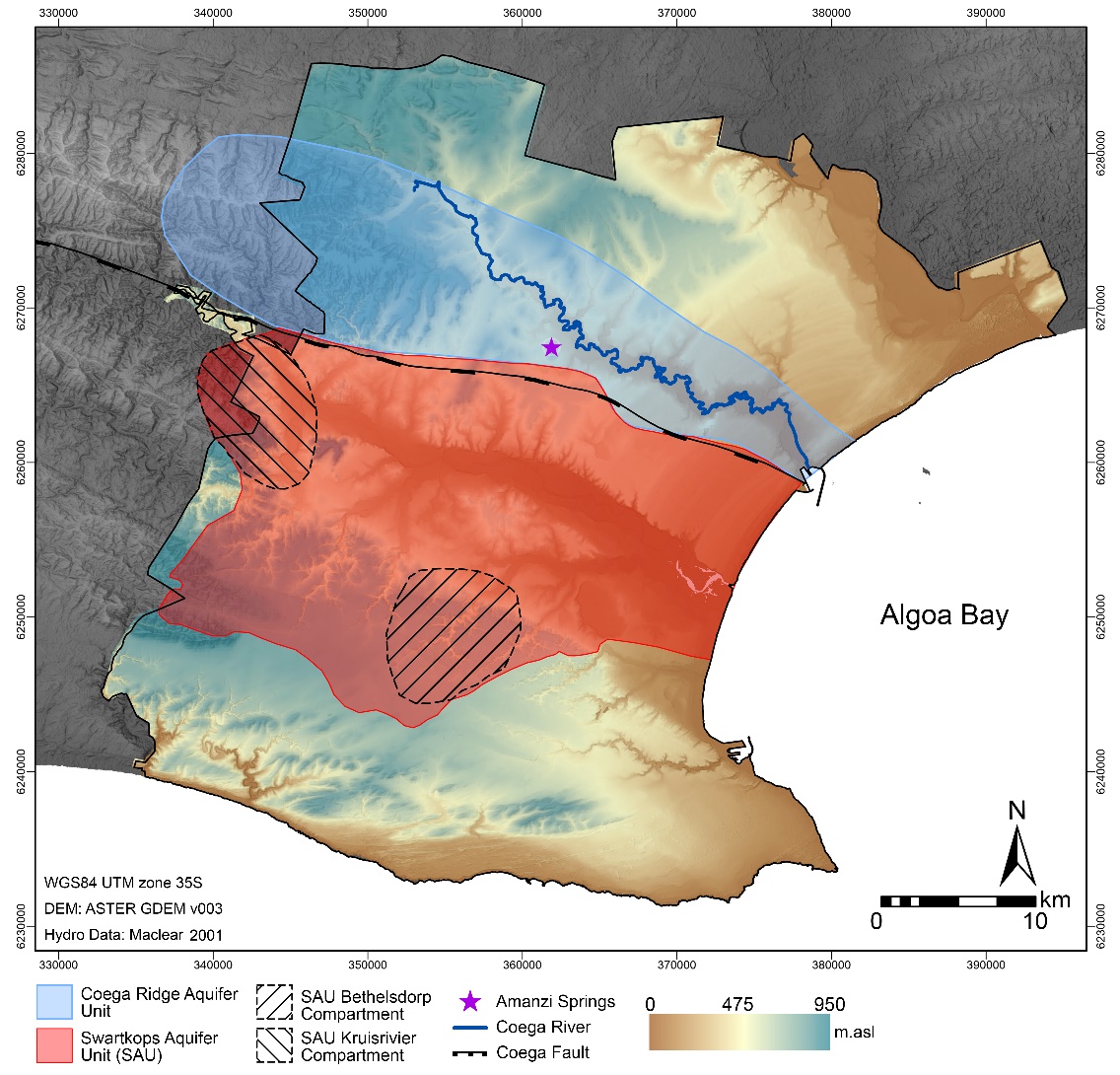


Fig. S3. Hydrogeological map of the study area**.** Showing delineation of the Uitenhage Artesian Basin with the Coega Ridge and Swartkops Aquifer Units. Hydrological data from Maclear ^14^. Figure produced using ASTER GDEM v003 ^134^ and ArcGIS Pro 3.4 (esri.com).

1.4. Local and Regional Raw Material Sources

Primary and secondary sources within a 20 km radius of Amanzi Springs were identified using 1:50k geological maps of the area (Fig. S1), and where accessible were surveyed on foot. Primary outcrops and secondary sources were inspected for archaeological material, photographed, and GPS coordinates recorded. Where possible, geological samples of raw materials were collected. An ongoing geochemical study aims to characterise the geological samples and develop a source probability map of the region.

Primary sources of both quartzite and silcrete occur near Amanzi Springs in various forms (Fig. S4). Outcrops of quartzite located within a 10 km radius of the site are mostly of the Peninsular Formation (Op), although other formations of TMG (Os, SS, Sg, and S-Db) are accessible within a 20 km radius. Quartzites of the TMG are hereafter referred to collectively as TMG quartzite. Primary outcrops of silcrete within the 10 km radius include pedogenic silcretes of the Salnova Formation (T-Q Si/Fe), and the Grahamstown Formation (K-Tg), with the former occurring closest to the site. Further from Amanzi Springs but still within a 20 km radius, silcretes are mostly of the Grahamstown Formation. No primary outcrops of chert, hornfels, igneous or fine-grained siliceous lithologies were identified within the 20 km survey radius.

The Peninsular Formation outcrops found within a 10 km radius of Amanzi Springs are the eroded remnants of the Cape Fold Belt that form low rising hills above the infilled sedimentary basin of Algoa Bay. Several of these are visible from the alluvial terraces of the Coega River Valley and from Amanzi Springs itself. The closest source is located at Amanzi Kop immediately south of the site. This deposit is associated with a remnant duricrust of Fe-rich silcrete, mapped as the Salnova Formation (Table S1), and is characterised by its distinctive dark reddish-brown matrix and smooth weathering surfaces that have the appearance of ironstone. It is distinct from the other silcretes in the area, particularly the Grahamstown Formation. Both quartzite and silcrete are obtainable at this source where erosion of the outcrop has detached tabular pieces with distinctive outcrop weathering surfaces (hereafter referred to as outcrop cortex). A large surface scatter with MSA-type artefacts made on silcrete was found at this source, including cores and refitting flakes (Fig. S5). Additional outcrops of TMG quartzite are located ~2.5 km to the north and north-west of Amanzi Springs, although no silcretes occur at these deposits. Numerous sources are available within 20 km of the site, the majority of which are found closer to the mountainous region of the Cape Fold Belt to the west. The TMG quartzites are of variable quality and fall along a spectrum from sandstone to quartzite, although higher quality quartzite can be readily found at most outcrops. Grahamstown Formation silcretes have been extensively mapped in the region, where they occur as hilltop terraces sitting above the TMG. The closest of these sources are located ~8.5-9.5 km to the west of Amanzi Springs, with larger and more numerous deposits found within a 20 km radius of the site. The petrographic characteristics of the Grahamstown silcretes, particularly the proportion of matrix to allogenic and detrital grains, varies widely in relation to the underlying lithology ^22^. Where they are found in association with quartzite deposits, such as the outcrops nearest to Amanzi Springs, silicification most often occurred via replacement of the interlocking grains of the quartzites with microcrystalline silica, resulting in the development of a remnant quartzitic fabric ^22^. Examples of these silcretes can also be found as cobbles with a water-worn cortex in secondary sources in the region.


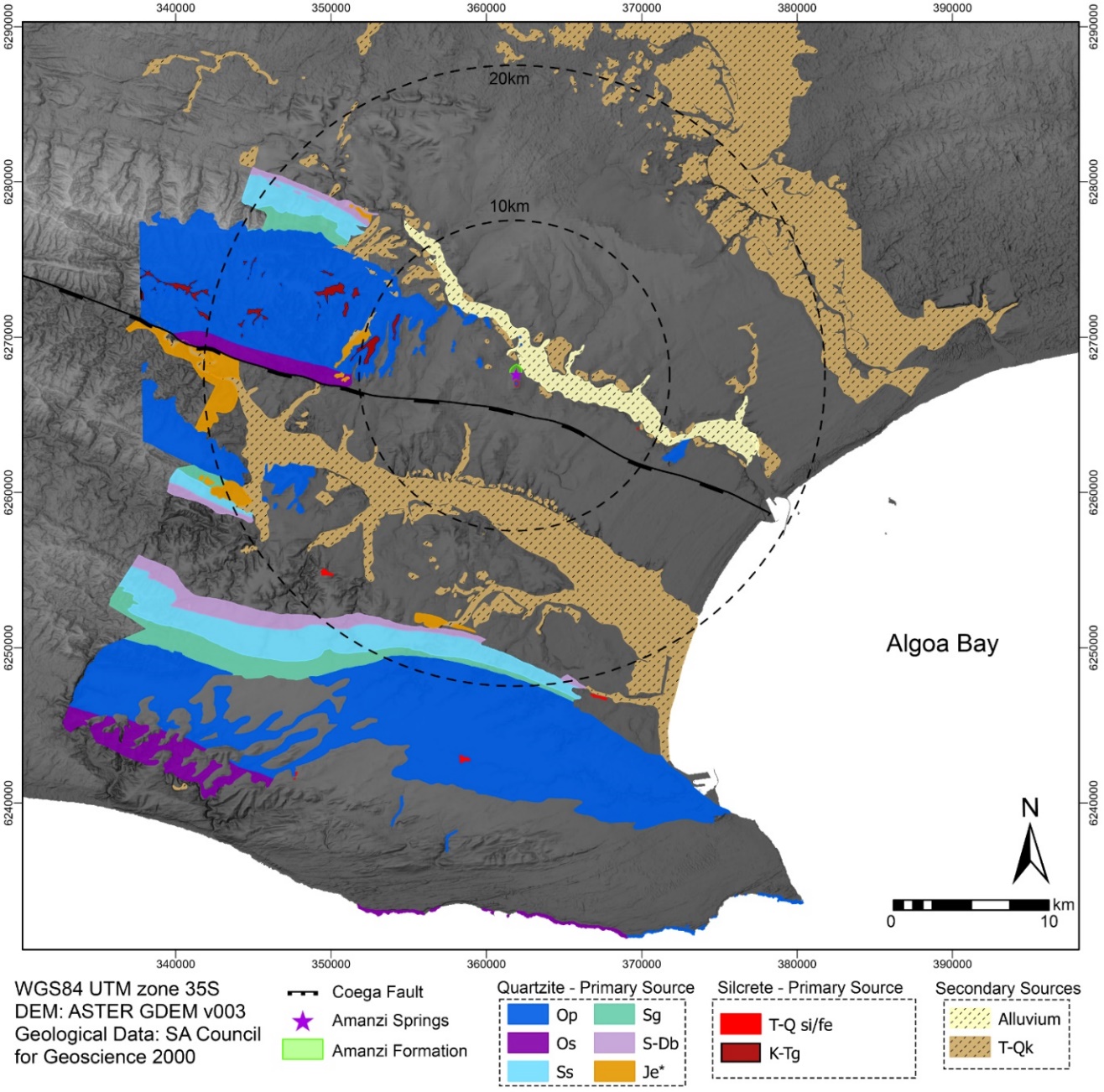


Fig. S4. Primary and secondary raw material sources identified within a 20 km radius of Amanzi Springs. *Je (Enon Formation) conglomerates are primary deposits that contain quartzite cobbles (see Table S1). Figure produced using ASTER GDEM v003 ^134^ and ArcGIS Pro 3.4 (esri.com).


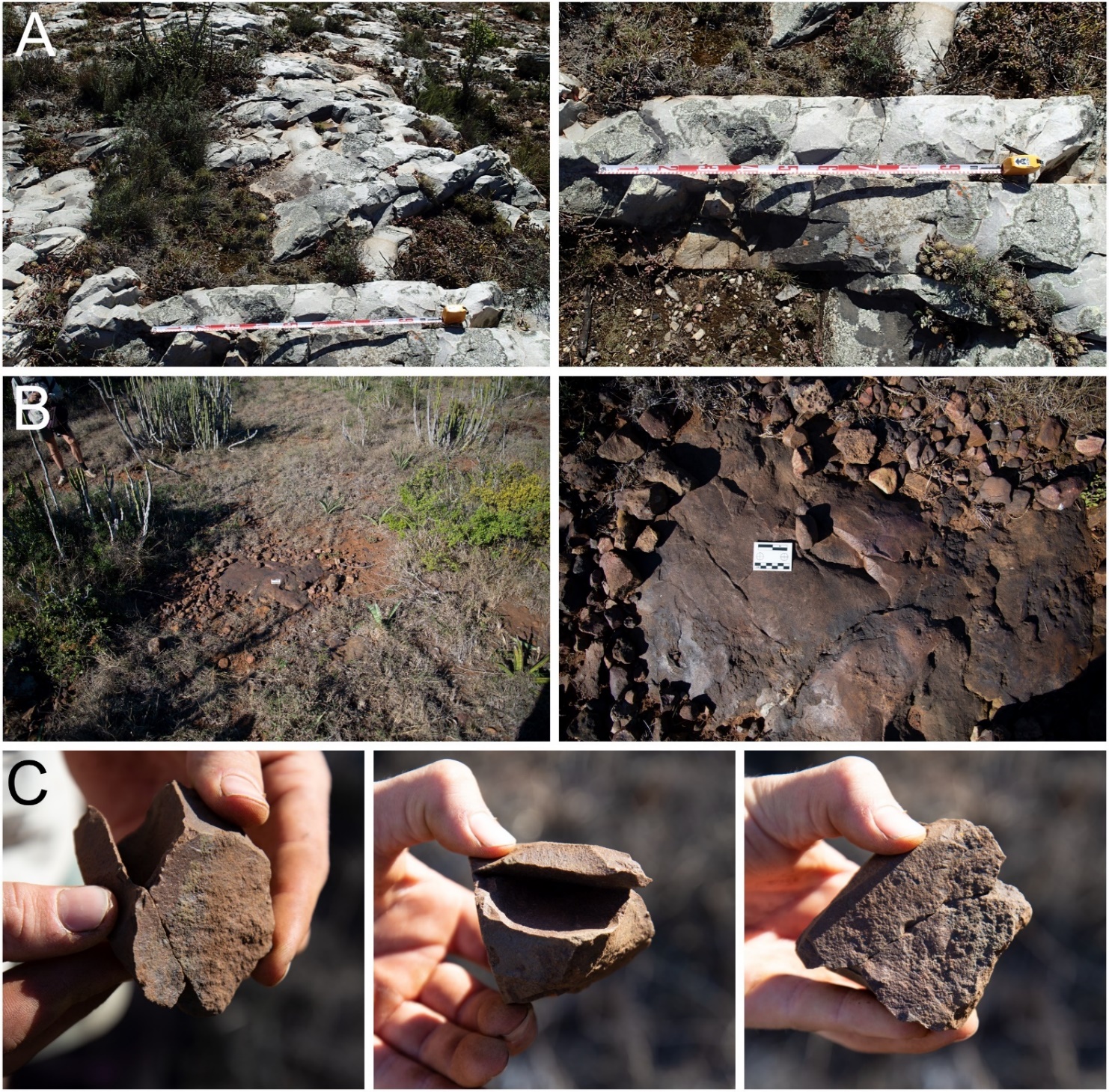


Fig. S5. Primary raw material sources close to Amanzi Springs including (A) Peninsula Formation (TMG) quartzite outcrop 3 km north of the site, (B) a silcrete outcrop at the summit of Amanzi Kop to the south of the site, showing smooth Fe-rich weathering surface and stone tool scatter, and (C) a flake and core refit found at this source.

Secondary sources of raw materials are also abundant in the area, mostly in the form of ex situ cobble deposits in stream and riverbeds, but also as isolated exposures of alluvial gravels and conglomerates in stratified geological formations. Nodules of quartzite, sandstone, silcrete, and to a lesser extent shale, mudstone, and siltstone, that have been transported in the drainage systems originating in the Cape Fold Belt can be found in the bedload of the rivers that dissect Algoa Basin. The closest secondary source to Amanzi Springs is the Coega River, located approximately 2 km north of the site (Fig. S6). The meandering path of the Coega River has incised through the alluvial terraces of the Algoa Basin, forming the Coega River Valley, and exposed sedimentary rock of the Uitenhage Group. The Enon Formation, the oldest and most proximal unit of the Uitenhage Group, is a conglomerate-dominated deposit that consists of sub- to well-rounded pebble to cobble sized clasts of sandstone, quartzite, and mudstone derived from erosion of the Cape Fold Belt during the breakup of Gondwana ^15^. It is distributed variably across the Algoa Basin, visible today in road-side cuttings and quarries, and where erosion and deflation have removed the Uitenhage Group sediments, disaggregated cobbles remain on the surface. Lithologies found within these Enon Formation deposits consist mainly of quartzite and sandstone of varying degrees of quality. The bedload of the Coega River forms the most abundant secondary source in this area. Cobbles found in the bedload of the Coega River vary in size from small pebbles to boulders and exhibit a water-worn cortex, often with abundant chatter-marks. Only quartzite and sandstone were identified at this source during this study. It is not presently possible to determine whether these cobbles represent redeposited Enon Formation conglomerates, or more recently transported clasts from the Cape Fold Belt, as both ultimately derive from the TMG deposits in this region.


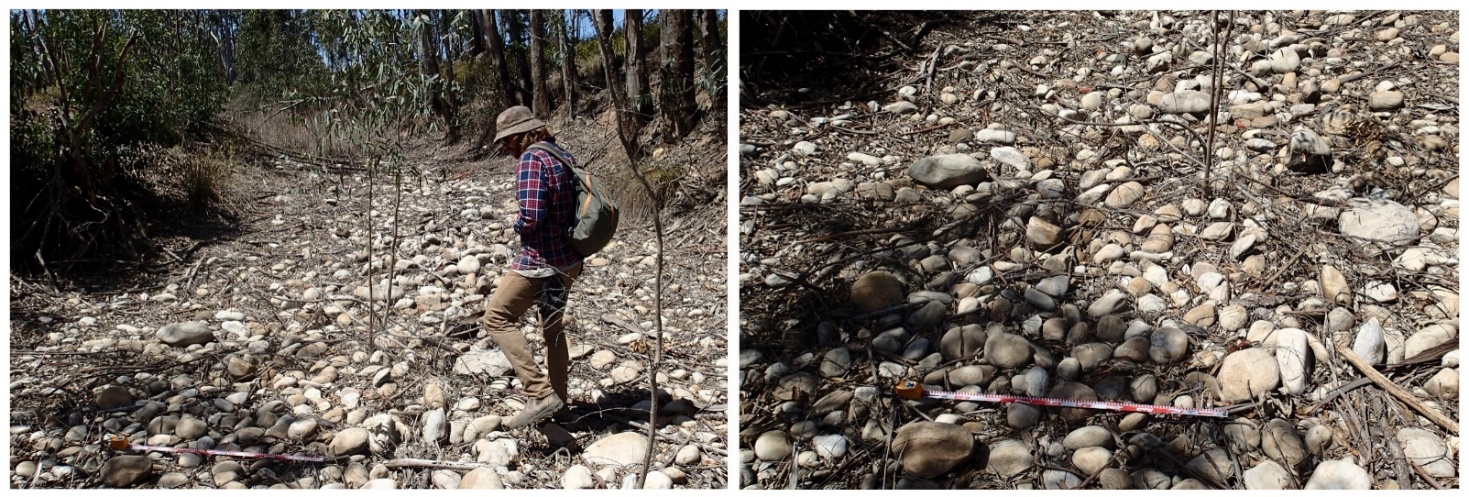


Fig. S6. Cobble bedload in the Coega River approximately 2 km north of Amanzi Springs.

1.5 Amanzi Springs Area 7

The Area 7 spring is located ~140m to the north-west of Areas 1 and 2 ^8,9^. It was first surveyed by Deacon ^3^, who identified stratified spring deposits exposed by a drainage furrow that had been dug through the southern end of the spring during the early 20^th^ century (Fig. S7). No further research was conducted at the site until 2015 when it was revisited during a survey as part of the newly formed Amanzi Springs Archaeological Project. Further erosion of the sections along the drainage furrow had exposed more of the spring deposits, including in situ artefacts, confirming the archaeological potential of the site. Despite the loss of a portion of the deposits caused by the drainage furrow and subsequent erosion (estimated to be <20% of the site), most of the spring on either side of the furrow remains undisturbed.

Excavations at Area 7 took place over five seasons between 2017-2019, initially targeting the erosional profile of the furrow (Sectors 1 & 2) and a 2x2 m test-pit to investigate the lateral extent of the intact spring deposits (Sector 4 test pit), after which excavations were expanded with a long trench linking the spring centre and margins (Sector 3) (Fig. S8-S10). In total, an area of ~42.75m^2^ was excavated, with deep soundings in Sector 1 and 2 reaching a depth of 2.6 m without encountering archaeologically sterile layers. Auger testing indicates that spring deposits continue to a depth of at least 6 m below the ground surface.


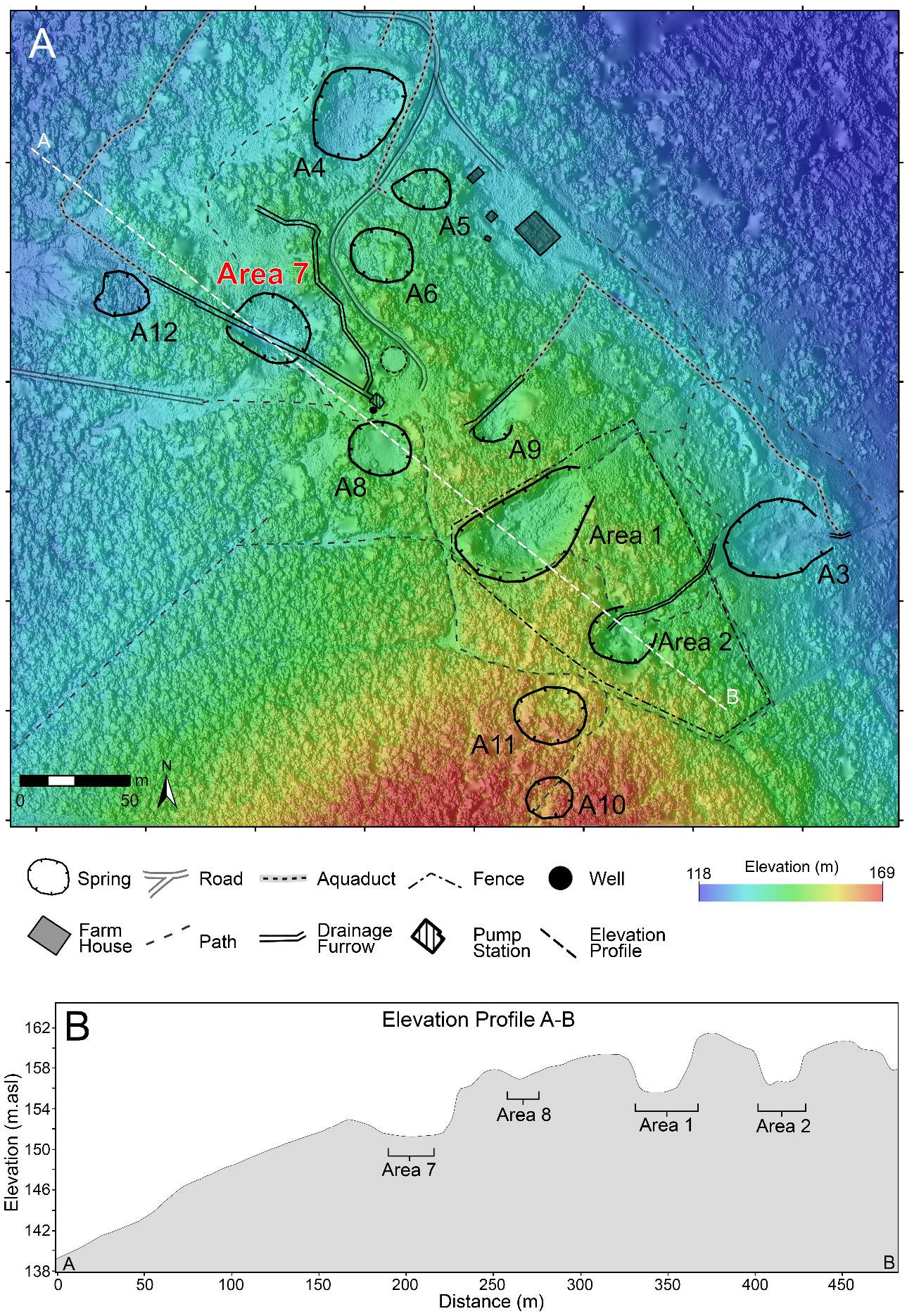


Fig. S7. Map of the Amanzi Springs complex. (A) Digital Elevation Model (DEM) showing the location of the springs, and (B) an elevation profile running northwest-southeast showing the location of Area 7 in relation to Areas 1, 2, and 8 (vertical axis scaled to highlight topography). Figure produced using ArcGIS Pro 3.4 (esri.com).


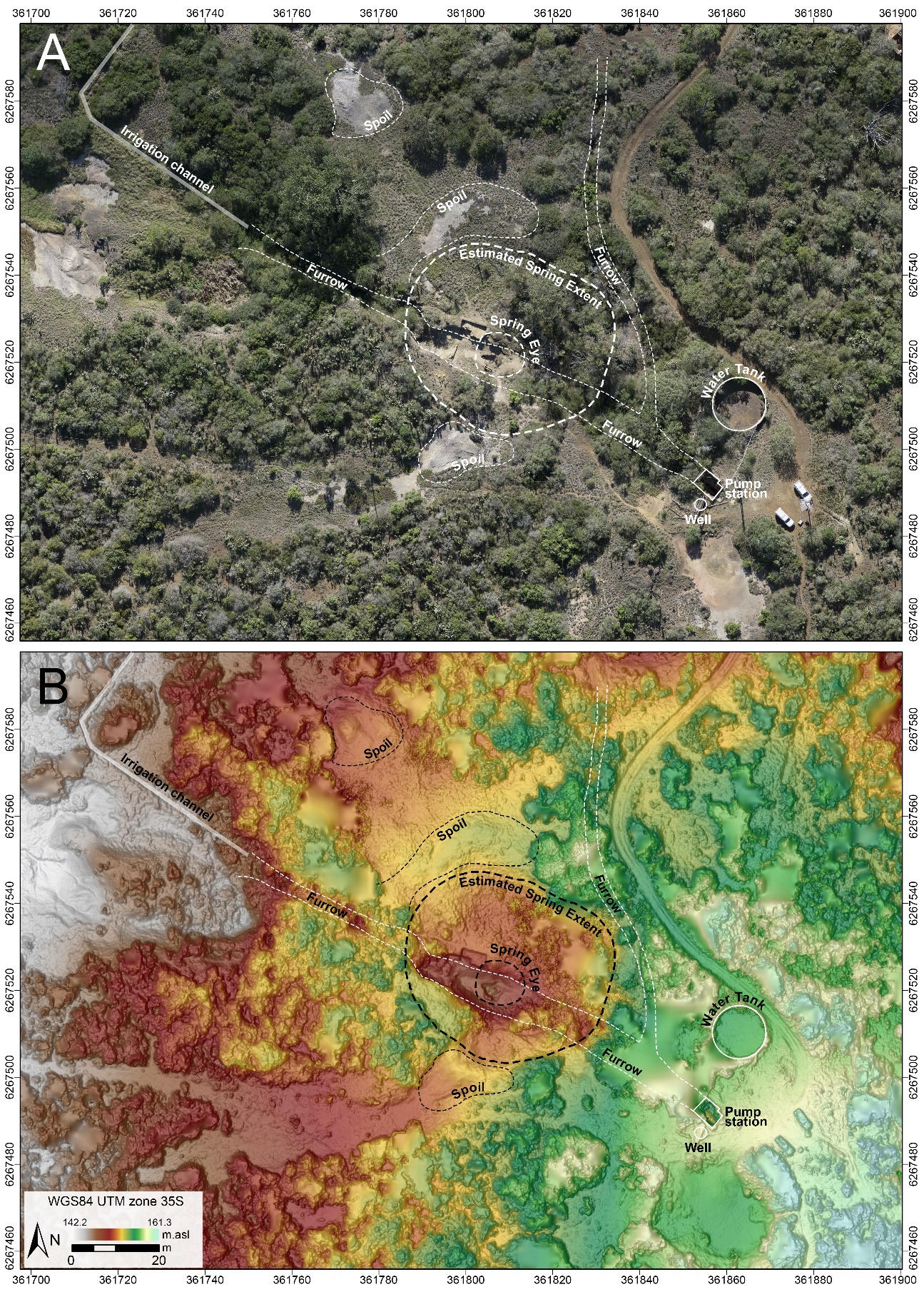


Fig. S8. Aerial view of Amanzi Springs Area 7 in 2017, showing features related to modification of the spring during the 20^th^ century. The spring eye and spring extent are estimated based on field observations. (A) an orthomosaic photo of the site during the first excavation season, and (B) the corresponding DEM (note that the DEM includes vegetation). Figure produced using ArcGIS Pro 3.4 (esri.com).

2. Geoarchaeology and Site Formation

The following section provides supplementary information on the Area 7 stratigraphic sequence, micromorphology and particle size analysis, and site formation summary.

2.1 Sedimentology and Site Formation

Five phases of spring formation were identified at AMZ7, referred to here as geological horizons (GH) 1-5, numbered from the top to the base of the sequence (Fig. 2; S9). Each horizon represents a continuous, long-term period of sediment deposition across the site, incorporating contemporaneous but distinct facies at the margins and towards the centre of the spring. Deposition was primarily via low to medium-energy groundwater flow from the centre of the spring basin, resulting in considerable lateral variation in sedimentary composition with distance from the spring outlets. Strata are mostly horizontally bedded, except at the centre of the basin where they dip sharply to the south and east, delineating the most geomorphologically active part of the spring.

Past spring activity was driven by long-term global, as well as local climatic conditions and changes in relative sea level, which influenced the rate of accumulation and composition of sediments. Periodic changes in groundwater flow rates at the spring also affected the structure and configuration of the plant communities that the springs supported. Cycles of reduced spring activity resulted in slower sediment accumulation, and the springs formed a marsh-like environment that supported semi-aquatic communities of plants ^23^. As groundwater flow increased during active phases of spring output, these layers were submerged and buried by silts and sands brought to the surface from the underlying Uitenhage Group and eventually formed peat, preserving organic materials including fragments of wood, charcoal, traces of the stems of aquatic plants, and other plant macrofossils.

The warm (~33℃) spring water at Amanzi Springs is slightly acidic (pH 5.5 - <4), with a low sodium (Na) and high iron (Fe) and manganese (Mn) mineral content. While not favourable to the preservation of osseous material, the geochemical conditions of the waterlogged deposits were beneficial for the preservation of botanical material ^2,8^. Organic preservation varies with distance from the central spring basin, with most botanical remains found closer to the spring centre where anoxic conditions in the water-logged sediments prevented oxidisation of organic matter. Except for small amounts of charcoal, very little organic material has been found at the spring margins.


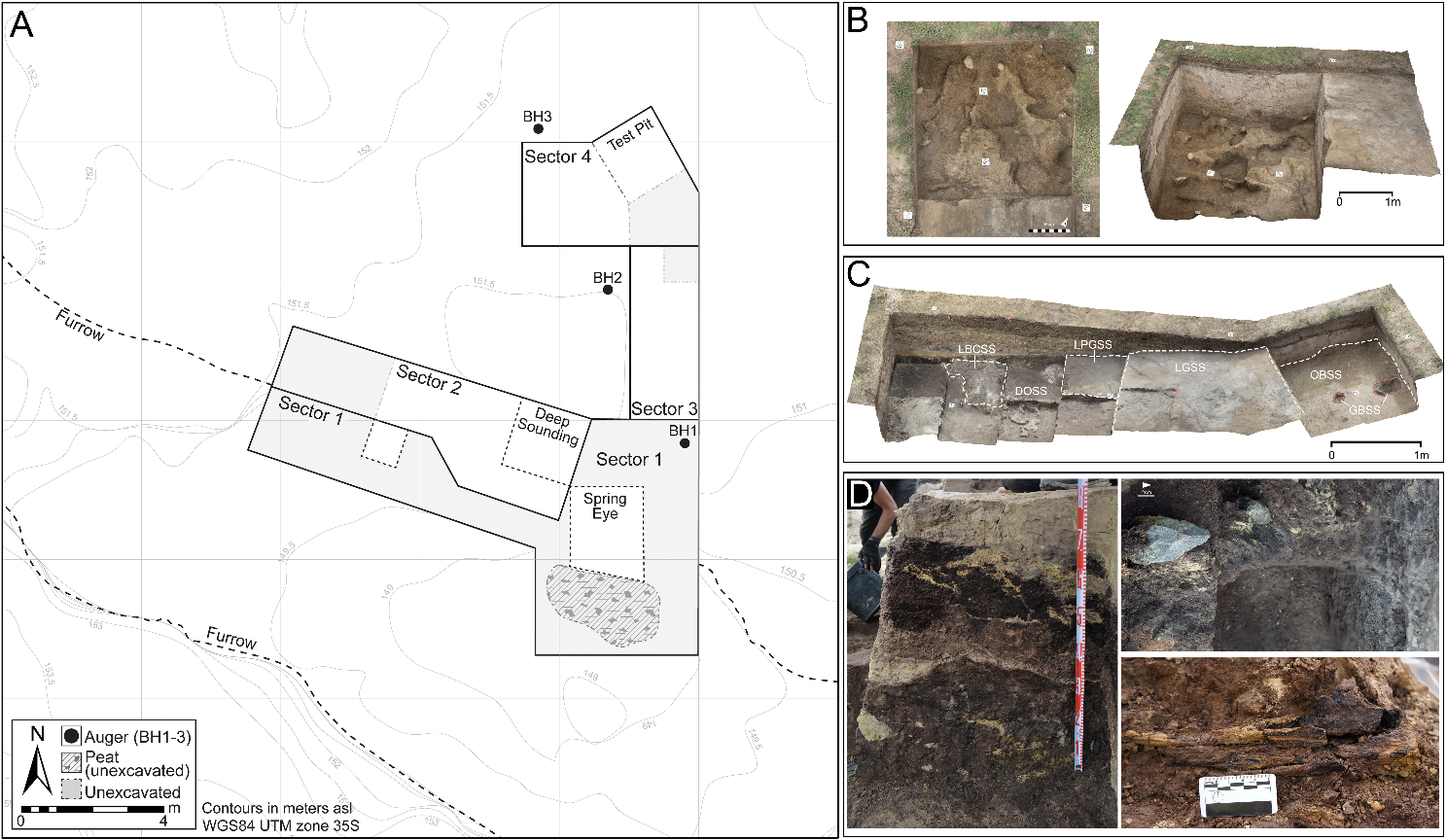


Fig. S9. Excavations at the Area 7 spring**.** (A) plan of excavations, (B) sector 4 test pit during initial excavation, (C) sector 3 during excavation, and (D) the sector 1 spring eye showing the west facing section (left) and *in situ* wood and artefacts (right).

**GH5**

The lowest and oldest unit currently excavated. This geological horizon includes the dark brown to black peaty sands (DBBPS), an aggregate of organic rich peat layers with preserved botanical remains including wood and plant macrofossils.

**DBBPS** The DBBPS includes a blackish layer at its base, overlain by a dark reddish-brown layer; both are composed of sandy loam to loamy sand with abundant organic matter. Yellowish-white lenses of sand, usually a few cm thick, are frequent. Light coloured sandy relics of pre-existing sediments are common. Subvertical or steep north-dipping cracks, filled with light coloured sand are present. Bioturbation is widespread. Abundant preserved organics, wood, and macrofossils. Lower contact not yet excavated, although small clay pedorelics towards the base of this horizon suggest that it embeds sediments reworked from older units still unobserved.

The micromorphology results indicate that DBBPS formed during a phase of still water in an environment that was close to the margins of the spring pool. This setting favoured the development of riparian vegetation and formation of turf, as indicated by the abundance of vegetal residues, partly mineralised, still in life position within the sediment. DBBPS is eroded to the south-east (towards the centre of the pool) forming a steep bank. Large clods of its sediment are reworked into the overlying unit near the erosion surface, suggesting that at least part of DBBPS collapsed towards the centre of the pool.

The lower horizon includes more abundant vegetal remains, consisting mainly of roots still in vertical position, and amorphous organic matter (AOM) suggesting that it formed in a low or null energy environment with low oxidation-reduction potential (Eh). Coarser inputs such as quartz sand resulted from regularly occurring higher energy episodes that deposited sediment among the vegetation stems. These episodes were probably more common during the deposition of the upper part of DBBPS, as suggested by the coarse grain size and the fragmented and chaotically dispersed vegetal remains. In fact, the top part of DBBPS is more oxidised and may have emerged for some time because of the lowering of the water table, so that soil started to develop on it. A thin reworked layer, also marked by secondary redoximorphic pedofeatures, can be observed at the boundary between peat layers within DBBPS, testifying to a hiatus separating the two subunits.

**GH4**

A sharp stratigraphic boundary separates GH5 from the overlying GH4, which consists of two units; the light brown compact sandy silt (LBCSS) at the spring centre, and the laterally equivalent green to brown silty sand (GBSS) towards the spring margins. The sedimentologic characteristics of GH4 suggest that water levels were raised above the top of GH5, forming a well-oxygenated basin. At the spring centre, the upper boundary of LBCSS is truncated by an abrupt erosional interface that separates it from the overlying GH3, forming a steep bank (Fig. 2d; Fig. S10). This major erosional event shaped the southern steep bank of LBCSS after a decrease in water level. In the north and west of the site, where GH3 overlies the GBSS layer, this erosional contact is less pronounced, varying from clear to diffuse with distance from the spring centre.

**LBCSS** The LBCSS is a grey-blue to light brown compact, hard sandy silt layer characterised by alternating subunits of fine, well sorted sand grading into a higher clay content near the top and base of this layer. Orange Fe-oxide staining in areas. It tapers towards the east and is heavily truncated by the overlying DOSS layer (GH3) at the edge of the spring centre (Fig. 2d; Fig. S10). The lower contact can be observed at the boundary with the underlying GH5 (DBBPS), where it is sharp with isolated load structures, and up to 1.2 m thick.

The texture of LBCSS is relatively fine, indicating a generally low depositional energy, even if some faint traces of crude layering/lamination may point to some rare but more energetic events. The organic matter is few and the relatively dark chroma of the sediment is largely due to the iron oxides that stain the clay textural component. This finer part is evenly dispersed within the sediment and accumulated within the basin together with the sand fraction. However, part of these minerals occurs in infillings and coatings that indicate post-depositional illuviation processes. The thickest of these infillings are microlaminated and include frequent fine amorphous organic particles that in some cases look charred. The texture of the LBCSS, the absence of vegetal remains, and the light colour indicate deposition in a centre basin environment, when the water level was raised above the top of DBBPS. The illuvial clay must have translocated into the sediment by soil forming processes when its surface was exposed. The occurrence of bits of amorphous and charred organic matter within the laminated infilling also suggests that wildfires affected the area. It is hypothesised that a northward shift of the basin depositional centre took place at or after this point, where an erosional phase removed the north-western and northern sides of LBCSS.

**GBSS** A green to brown fine to medium silty sand, with very well-developed medium prismatic aggregation. Laterally contiguous to the LBCSS layer, GBSS is fine and clayey, also suggesting deposition in a still basin environment, albeit lateral to LBCSS to the west and north. These two layers grade into one another in the western profile and towards the north of the site where they are exposed by excavations. They likely represent an energy-sorted progradation of sediments into the spring eye, with grain-size decreasing with distance from the point of sediment input into the basin, which would have been located to the south-east of the spring-eye. Frequent brownish red to yellowish Fe-oxide redoximorphic pedofeatures, mostly represented by coatings on ped sides and by mottles. Disturbances include small, speckled sandy pockets, roots and rootlets, and small burrows. GBSS is the lowermost excavated unit at the spring margins in the north and west of the site, and the lower contact has not yet been exposed.

**GH3**

GH3 consists of the orange to brown silty sand (OBSS) at the spring margin, and the dark organic silty sand (DOSS) layer towards the spring centre. At the spring centre, the upper boundary of DOSS has been truncated by farming activities and is overlain by modern surface deposits with a sharp contact. DOSS is characterised by very abundant organic matter which, unlike GH5, is mainly composed of highly fragmented and variably humified vegetal remains. This aspect indicates that environmental conditions were different from typical peat formation, with the transport into the pan of partly humified and partly charred material originated in areas subject to wildfires.

**OBSS** A light orange to brown silty sandy loam with medium to fine very well- (top) to well-developed (bottom) polyhedric to prismatic aggregation. Dominant orange to reddish Fe-oxide coatings on ped surfaces, as well as other redoximorphic pedofeatures, including small whitish mottles within the greyish sediment. Frequent modern roots and rootlets, soil mesofauna burrows and other biological disturbances. Lower contact in this part of the spring with GH4 (GBSS) is diffuse and irregular, and the thickness of the OBSS layer varies from 65-70 cm.

The grain-size of the OBSS layer is much finer than the GBSS, as indicated by its well-developed prismatic aggregation. It is likely that GBSS and the overlying OBSS can be considered as forming during a continuous, long-term period of sediment accumulation. The top of OBSS was later modified by soil-forming processes, as indicated by its better developed pedality, the patches of coarser sediment infiltrated into cracks, and the numerous traces of roots hypocoated by redoximorphic pedofeatures.

**DOSS** A dark brown to blackish compact moderately- to well-sorted very fine to medium silty sand. The DOSS is the lateral equivalent of the OBSS, where it overlies the LBCSS. The sediment is crossed by a network of light yellowish bands, probably crack infillings or depletion features within the sediment. Frequent Fe-oxide redoximorphic pedofeatures (mottles, coatings along voids); heavily bioturbated throughout. A sharp and irregular limit with frequent load and flame structures separates it from the underlying GH4 (LBCSS). It sharply plunges southwards towards the centre of the spring basin but becomes more horizontal in the west and north with distance from the spring centre. The top of this layer has been removed by modern alteration of the landscape, and the maximum thickness where it is exposed by excavations is 30 cm. Stratigraphically it grades into the top of the OBSS, its lateral expression in the west and north profiles, indicating that it was deposited towards the end of the formation of OBSS. This also suggests the sedimentary sequence towards the margins of the spring was not as heavily impacted by the erosional event that truncated LBCSS and is more intact.

**GH2**

GH3 consists of the light pink to grey sandy silt (LPGSS) layer. As with the top of the DOSS unit (GH3), the overlying GH2 and GH1 have been truncated at the centre of the spring by modern alteration of the landscape. They are preserved at the spring margins, however, where their sedimentary characteristics indicate limpid and oxygenated water had again filled the pan. Here, GH2 overlies DOSS (GH3) in the north of Sector 3, and OBSS in Sector 4, with an abrupt to clear contact (Fig. 2e; Fig. S10).

**LPGSS** A massive and very compact light pinkish brown to grey sandy loam with moderately sorted fine sand. Frequent Fe-oxide coatings on ped surfaces, cracks and root channels, particularly towards the base of the layer. Common biological disturbances (roots, insect burrows). Lower contact with GH3 (OBSS in Sector 4; DOSS in Sector 3) is sharp to abrupt, smooth and gently dipping northwards. The thickness of the LPGSS layer varies from 15-25 cm.

At the spring margin, the shape, texture and colour of the LPGSS and the overlying LGCC suggest moderate energy deposition, preceded by moderate erosive processes, at least in this part of the pan. This would represent a newer phase of deposition that shaped the top of DOSS more to the north and east, suggesting another shift of the erosional and depositional processes.

**GH1**

GH1 incorporates the light grey sandy silt (LGSS) layer. LPGSS and LGSS are not extremely different at the microscope scale, suggesting that the pool environment did not change remarkably through the time it took for their deposition. However, they differ under the microscope because LPGSS includes a large amount of phytoliths, which derive from the weathering of vegetal components and complete removal of their organic fraction in oxidising environment, whereas LGSS is characterised by some amorphous organic matter. The upper contact of GH1 is sharp and horizontal, sitting directly below the modern disturbed ground layers.

**LGSS** A light grey, compact and very fine to fine sandy loam to sandy silt loam. Clods of light brown/reddish sediment, rather loose, are sparsely included at the base of this unit, probably representing the remains of a thin layer of clay loam with well-developed fine to medium polyhedric aggregation. Few to common Fe-oxide staining within/around voids, disturbances include modern roots, insect burrows. Lower contact with GH2 (LPGSS) is abrupt to clear, wavy in east-west direction and sloping/concave upwards towards the south. At the boundary between these units, there is evidence of a short event that transported clods of reddish sediment eroded from elsewhere into the basin. The thickness of the LGSS layer varies from 25-34 cm.

**Modern Surface Deposit**

The uppermost layers of the Area 7 deposit are composed of modern fill deposited after the spring was modified in the early 20^th^ century. At the top of this package is a modern soil horizon approximately 10-20cm deep in areas, under which are at least two separate layers of ‘made ground’ composed of compacted and mixed sandy silts, with abundant inclusions consisting of large clasts of clay, ironstone, organic material, roots from modern surface vegetation, and occasional modern materials (fragments of brick, glass, and metal). These layers were formed by the dumping of material as the drainage furrow was dug through the deposits in the south of the site. The base of this package of surface layers forms a sharp contact that truncates the intact spring deposits with a moderate dip towards the southeast, visible in the northern and western sections (Fig. S10). The modification of the Area 7 spring resulted in the loss of a portion of the upper layers of the deposit, particularly at the spring centre, which we estimate to be between 1-5 m based on the height difference between the top of the southern section of the furrow and the present-day surface to the north. While we cannot be certain of the exact amount of deposit that has been removed, it is likely that considerable portions of the upper layers (GH1-2) have been lost, particularly towards the spring centre, with a remnant of these deposits remaining at the margins of the spring in the north of the site. There is a sharp contact with the intact spring deposits below the modern fill layers.


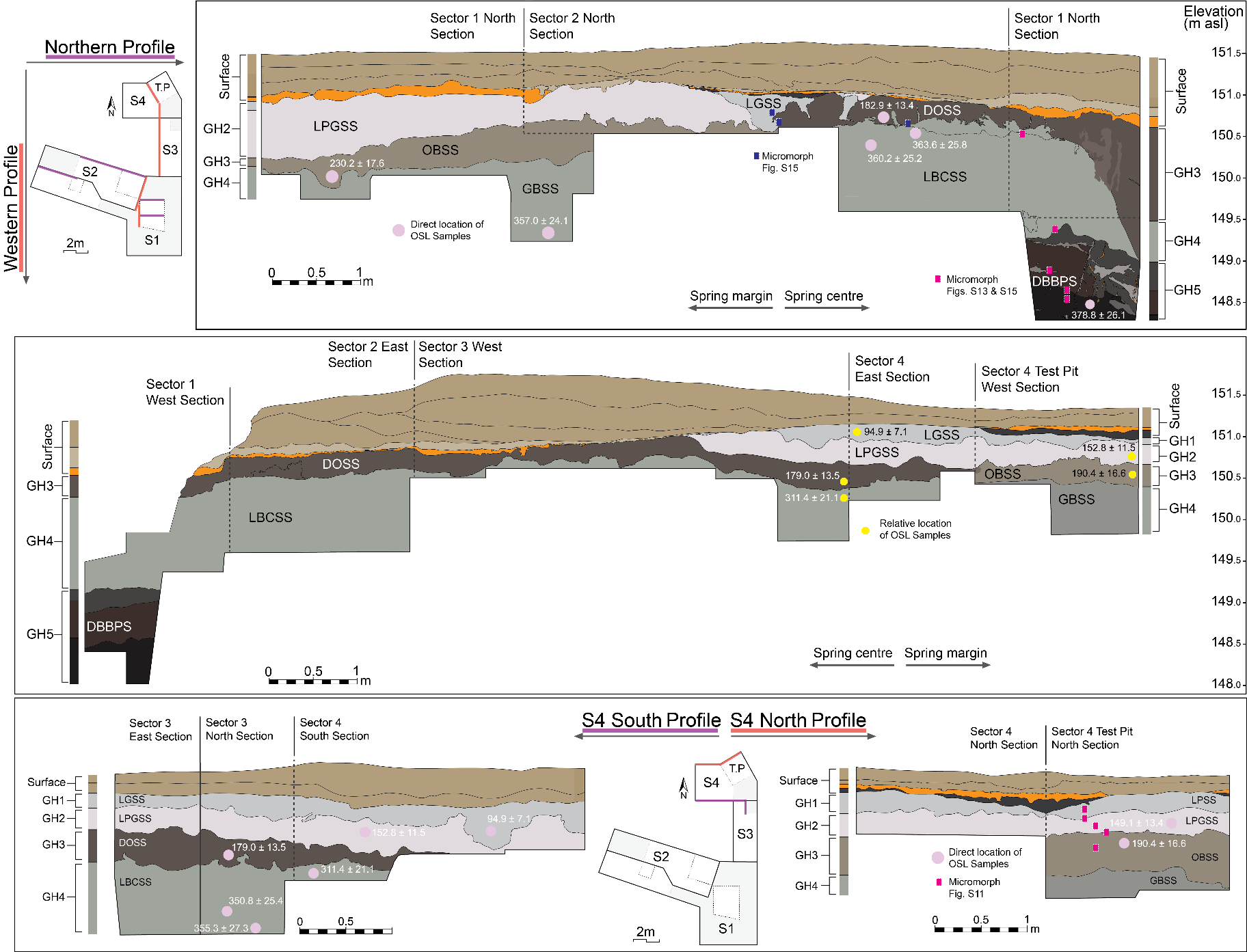


Fig. S10. Stratigraphy of the Area 7 spring**.** Composite stratigraphic profiles: north and west (top), and sector 4 north and south (bottom).

2.2 Micromorphology and Particle Size Analysis Summary

**Spring Margins (base to top)**

***GH4 - GBSS (green to brown silty sand)***

The micromorphological sample includes a homogeneous sediment mainly composed of angular to subangular quartz grains, poorly sorted and comprised between coarse silt and medium sand. The fine component is abundant, about 60%, and composed of clay with very well developed striated and granostriated b-fabric. The microstructure is massive and compact within the peds, which are separated by common cracks that are marked by slickensides and/or common Fe-oxide coatings/hypocoatings (Fig. S11a). Some channels are also present.

The mineral particle size demonstrates there is variability throughout the GBSS and overlying OBSS (GH3) units. Silt decreases from 48% at the base to 33% at the transition to the OBSS layer (Fig. S12). Conversely sand increases from 51 to 66%. The clay fraction is <1% throughout. The deposit is generally poorly sorted (mean σ*_G_* 3.8).

***GH3 - OBSS (orange to brown silty sand)***

At the microscopic scale, the sediment is dominated by subangular to angular quartz grains, with some rounded among the largest ones. The grain-size is very poorly sorted (σ*_G_* 3.95-4.4), ranging from coarse silt to medium sand-size granules, with occasional mm-size polycrystalline elements. The grain-size is also spatially distributed very irregularly, with juxtaposed patches (possibly clods or aggregates) characterised by distinct grain-size distribution (Fig. S11b). The fine component is comprised between 20-40% and is composed of clay with stipple-speckled or -less frequently granostriated- b-fabric. The c/f-related distribution ranges from single-spaced to double spaced porphyric to open porphyric, depending partly on the grain-size of the coarse fraction. The silt-sized and sand fractions compose a roughly equal proportion of the particle size distribution (41-59%, mean 49% and 39-57%, mean 49% respectively) (Fig. S12). The microstructure is generally massive, crossed by irregular cracks and common channels. Clay coatings may occur within the elongated voids, whereas dark brown Fe-oxide hypocoatings are very common. A micromorphological sample was collected close to the top of the unit, where soil forming processes are put into evidence at macroscopic scale by frequent redoximorphic features situated around channel-like voids.

***GH2 - LPGSS (light pink to grey sandy silt)***

At the microscopic scale, the sediment is dominated by subangular to angular quartz grains. The grain-size is poorly sorted and crude lamination of fine and coarse layers can be commonly observed (Fig. S11c), with no significant variation in measured-size distributions from the underlying OBSS layer. The c/f-related distribution ranges from single-spaced to double spaced porphyric to open porphyric and the microstructure is massive, crossed by common irregular cracks and including some channel-like voids. Among the pedofeatures, clay coatings are common, mostly disrupted and dispersed within the groundmass, but also frequently located within the voids. Phytoliths are very common throughout the sediment, giving the micromass a light colour in the areas where they are particularly abundant (Fig. S11d). In some cases, they are still articulated within larger vegetal structures.

The interface with the overlying GH1 (LGSS) is marked by a thin (2-3 cm) layer of brownish-reddish clods that are strongly inhomogeneous, sometimes including aggregates of almost pure clay with striated b-fabric, and reddish clay aggregates (Fig. S11e), probably pedorelics. The grain-size of the coarse fraction ranges from medium silt to fine sand (occasionally medium sand), with frequent lateral variation. The fine fraction ranges from 40 to 25%, being more common where the grain-size of the sand fraction is finer. It usually occurs among sand/silt grains with stipple-speckled or granostriated b-fabric. Aggregates of clay with few skeletons are also common (Fig. S11c).

***GH1 -*** ***LGSS (light grey sandy silt)***

Under the microscope, LGSS is dominated by subangular to angular quartz, with few subrounded and rounded grains. The sediment is very poorly sorted, with particles ranging from coarse silt to 3-400 μm, with common larger grains, up to 1 mm. The fine fraction is about 30% or even more in some parts, represented by greyish clay whose b-fabric is stipple-speckled or less frequently striated or granostriated. Silt-size bits of structureless amorphous organic matter (AOM) are commonly included within the fine fraction. Silt dominates the particle size distribution (77-82%), with sand composing 16-21% of the sample. Clay averages 1.4%. The microstructure is massive, with very few voids shaped as channels or elongated horizontal cracks; the c/f-related distribution is close to single-spaced porphyric. Very few voids include poorly developed pure clay coatings (sometimes also infillings) with strong continuous orientation; these coatings/infillings are sometimes disrupted (Fig. S11f), probably due to sediment compaction and void volume reduction. Occasional Fe-oxide impregnations occur throughout the sediment mass. Hints of horizontal variation in grainsize suggest some sort of crude and discontinuous layering occurring sparsely throughout the sediment.


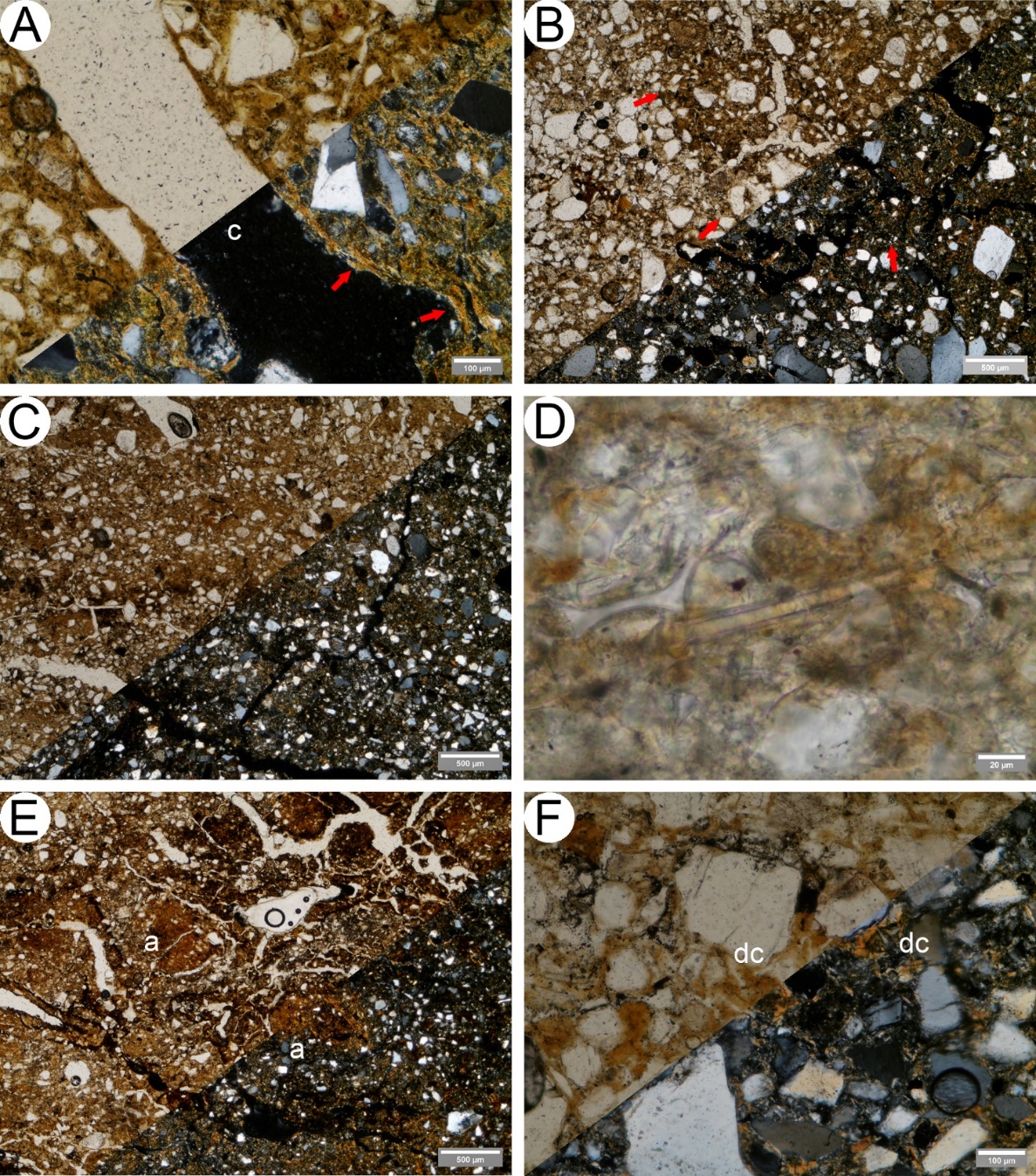


Fig. S11. Photomicrographs of Area 7 thin sections**.** (A) GBSS. Angular quartz sand embedded in abundant clay with stipple-speckled and striated b-fabric; the large void (c) is a crack separating peds with discontinuous slickensides on their faces (red arrows) (left: PPL; right: XPL). (B) OBSS, top part. Patches of sediment with distinct grain-size, separated by abrupt limit (red arrows), but without evident discontinuity, possibly representing sediment infiltrated into soil cracks; the right one is darker, with finer skeleton and more fine fraction (left: PPL; right: XPL). (C) LPGSS. Crude lamination of alternating fine and coarse sediment (left: PPL; right: XPL). (D) LPGSS. Elongate and geniculate phytoliths, located within patches/layers of light grey sediment dominated by finely fragmented phytoliths. (E) interface between LPGSS and LGSS. Clay-rich aggregates, probably pedorelics, embedded in an inhomogeneous sediment composed of clods and aggregates of sediments with distinct grain-size (left: PPL; right: XPL). (F) LGSS. Dusty/dirty clay infillings (ci) with stipple-speckled fabric (left: PPL; right: XPL).


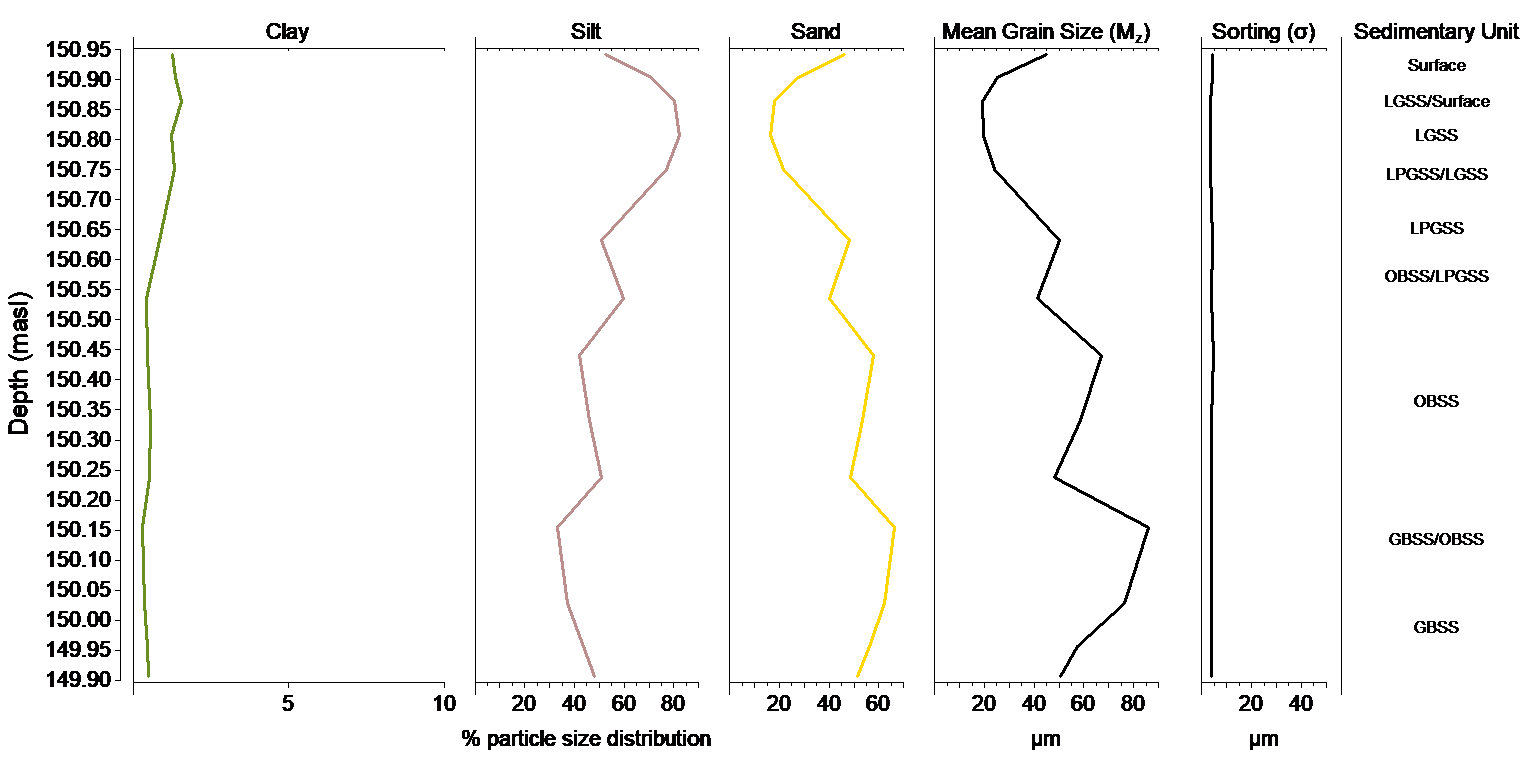


Fig. S12. Particle size distribution for layers GBSS, OBSS, LPGSS, and LGSS, presented as percentages of clay, silt, and sand. Mean grain size and sorting calculated based on geometric Folk and Ward ^24^ graphical measures.

**Spring Centre (base to top)**

***GH5 - DBBPS (Dark brown to black peaty sands)***

At the microscopic scale, the skeleton is dominated by angular to subangular quartz, poorly sorted within the classes of medium to very fine sand. It can be observed that the fine fraction is relatively few (around 15-20%) in the lower profile, but the grain-size distribution is strongly inhomogeneous, with areas where it may even become dominant. Reddish and altered shards of bubbly glass are present, though occasional. Light brownish clay aggregates with high-order interference colour under cross-polarising light (XPL), subrounded to rounded but with rough surface are common (Fig. S13a). This unit embeds well-preserved roots (up to several mm long) that are usually organised vertically and in more or less parallel arrays (Fig. S13b). In some areas, the root remains are disrupted into smaller fragments that are chaotically dispersed. Interestingly, the grain-size of the mineral sand-silt fraction is smaller in these areas. Smaller fragments of roots and of other vegetal remains are frequent. The amorphous organic matter is dominant within the fine fraction and includes opaque rounded-subrounded aggregates (up to 2-300 μm wide) that very rarely display residues of vegetal cellular structures. Fine sand-size aggregates of pure clay with stipple-speckled b-fabric are frequent (Fig. S13b-c).

The mineral fraction in the upper profile of DBBPS is slightly coarser, with common quartz grains in the medium sand fraction; the fine fraction is about 40-50% and is composed of Fe-stained brownish clay with amorphous to poorly developed stipple-speckled b-fabric. Vegetal remains are few to common, and usually represented by fibres at various degrees of mineralisation, not longer than 1.0-1.5 mm and randomly dispersed throughout the sediment. The contact between the lower and upper peats within DBBPS is marked by a 1-2 cm-thick layer whose texture and mineral composition do not differ significantly from the lower unit, but with slightly more frequent red glass grains. The organic component is less abundant and is composed of strongly fragmented vegetal remains. These are mainly dispersed chaotically within the sediment; in some cases, the elongated ones tend to lie parallel to the bedding planes. The top of this unit includes the same organic remains, which in this case are less sharply oriented downslope. The micromorphological observations are consistent with the mineral sediment size data (Fig. S14). This sedimentary unit is dominated by silt-sized clasts (11-77%; mean 57%), with a secondary sand component (17-89%, mean 49%). The highest sand relative abundance is in the uppermost sample. Clay is present in very low proportions (0-6%, mean 2%). The deposit is poorly to very poorly sorted (σ*_G_* 3.8-5) and the mean grain size ranges 18-150 µm (mean 49), with a in increasing trend from the base to top of deposit.

***GH4 - LBCSS (light brown compact sandy silt)***

At the microscopic scale, the texture is characterised by unsorted sand, with grain-size up to coarse sand; it is composed of dominant angular to subangular quartz, with occasional feldspars and chert. The coarse component makes up about 40%; in the bottom part of the observed sample, extremely faint traces of crude layering marked by changes in grain-size can be detected (Fig. S13d). The fine sediment is composed of dusty clay, amorphous or with moderate stipple-speckled or granostriated b-fabric. It fills a large part of the spaces among quartz grains, so that the microstructure is massive. Part of the fine component of the sediment is represented by clay coatings or infillings that are irregularly distributed within the sediment. The small ones fill the spaces among few (not more than 10-12) coarse grains and occur throughout the sample, though more frequently in the upper part. The majority of these pedofeatures are composed of microlaminated limpid clay that shows moderate to strong continuous orientation marked by characteristic b-fabric (Fig. S13e); however, in several cases the clay is dusty to impure, non-microlaminated, and with amorphous b-fabric (Fig. S13f). The large clay infillings fill large spaces among many grains, can be up to 1-2 cm wide, and correspond to the yellowish mottles observed at macroscopic scale (Fig. S15a). They are microlaminated, with alternating almost pure clay and silty layers. Frequent AOM is included within these infillings, where it usually lies parallel to the lamination. In most cases the larger AOM fragments look humified, but some may point towards charring. The contact between GH5 (DBBPS) and LBCSS is marked by a 2-3 cm thick layer, apparently discontinuous, composed of several wavy sandy to silty clay laminae, each with a different texture (Fig. S15b). Quartz grains removed by erosion from the underlying DBBPS are frequently embedded in the intermediate layer, mostly close to its bottom (Fig. S15c). The particle size of this unit is dominated by silt (mean 75%) with a secondary sand component mean (25%) (Fig. S16). At the upper boundary, between LBCSS and GH3 (DOSS), the sediment is composed of 58% sand and 40% silt. Deposit is very poorly sorted (σ*_G_* 3.62-3.69).

***GH3 - DOSS (dark organic silty sand layer)***

The texture is sandy, with poorly sorted grains seldom exceeding the fine sand class, and few fine mineral fraction. The minerals are angular quartz and very few feldspars and chert; altered glass clasts, up to 1.0-1.5 mm wide, are frequent. The c/f ratio is about 70-80% if organic matter is not counted as fine fraction, or 8% if it is. Tiny speckles of b-fabric suggest that some phyllosilicates are hidden within the amorphous mass. The microstructure is open porphyric and the voids occupy 40% of the sediment volume on average, so that the whole sediment mass is rather fluffy (Fig. S15d). Mineral particle size distributions are consistent with the c/f estimates excluding the organic fraction. Sand-sizes clasts range 53-66% (mean 61%), with silt ranging 32-45% (mean 36%) and the overall mean sizes ranging 49-74 µm. The deposit is very poorly sorted (mean σ*_G_* 4.23).

The abundant organic matter gives the sediment its characteristic grey to dark brown colour. It includes a wide spectrum of preservation states, from amorphous to moderately mineralised. The remains are unidentifiably small in the case of the AOM, whereas larger remains of vegetal anatomical parts -with identifiable cell structures- occur among the mildly mineralised fragments (Fig. S15e). The size of the AOM aggregates is always small (up to 2-300 μm), whereas the well-preserved vegetal tissue remains are up to millimetres long. Vertical cracks in the sediment mass, up to a few mm wide, are usually filled with AOM-rich fine sand and silt including tiny phyllosilicate laminae. The lower contact with GH4 (LBCSS) is frequently obscured by dense impregnations of Fe/Mn-oxides that give the whole sediment mass a dark brownish too greyish colour (Fig. S15f).


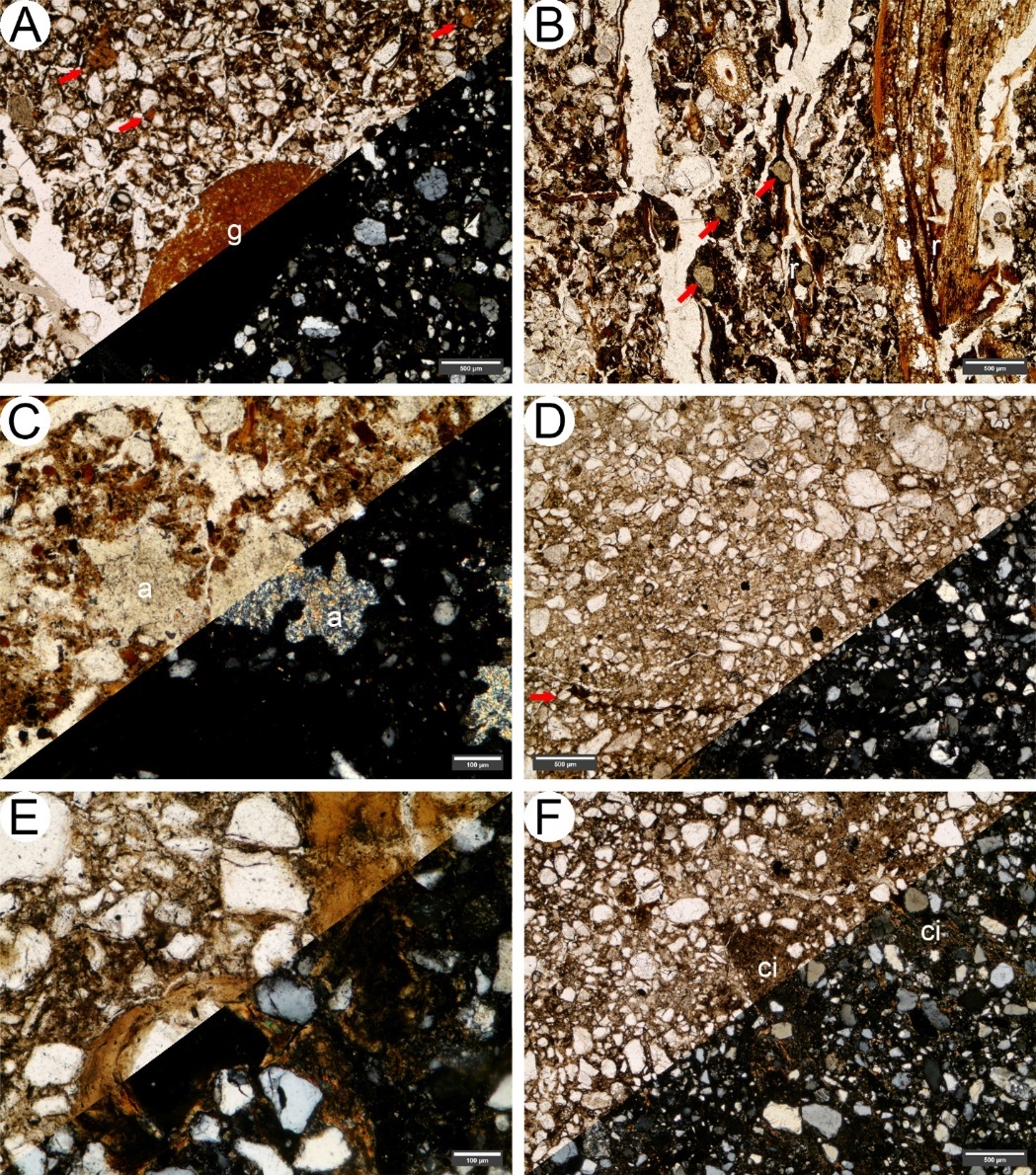


Fig. S13. Photomicrographs of Area 7 thin sections**.** (A) DBBPS (Peat A), top part. Angular to subangular quartz sand embedded in amorphous organic matter (AOM)-rich micro-mass¸ clay occurs as small reddish aggregates (red arrows) or is masked by the organic matter. Large, rounded grains of altered glass (g) occur sparsely (left: PPL; right: XPL). (B) DBBPS (Peat B), lower part. Well-preserved roots (r) vertically and parallel organised, embedded in AOM-rich micro-mass; minute pure clay aggregates (red arrows) are frequent (PPL). (C) DBBPS (Peat A). Detail of B: pure clay aggregates (a) with stipple-speckled b-fabric (left: PPL; right: XPL). (D) LBCSS. Very crude layering of finely (bottom) and coarsely (top) textured sediment; bottom of fine layer marked by a thin AOM and Fe/Mn-oxide rich lamina (red arrow) (left: PPL; right: XPL). (E) LBCSS. Irregularly laminated pure clay infillings with granostriated b-fabric (left: PPL; right: XPL). (F) LBCSS. Dusty/dirty clay infillings (ci) with stipple-speckled fabric (left: PPL; right: XPL).


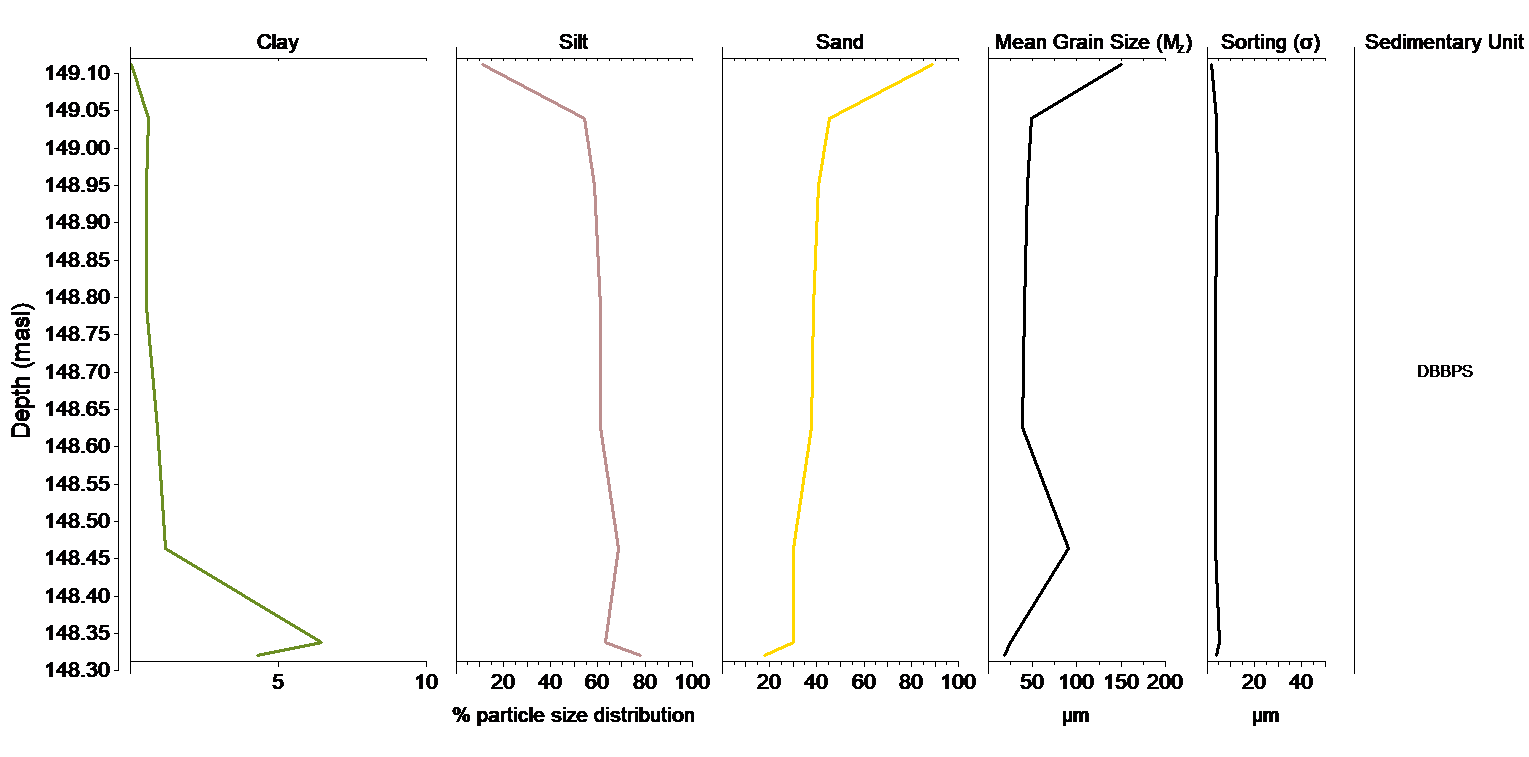


Fig. S14. Particle size analysis of samples from the DBBPS, presented as percentages of clay, silt, and sand. Mean grain size and sorting calculated based on geometric Folk and Ward ^24^ graphical measures.


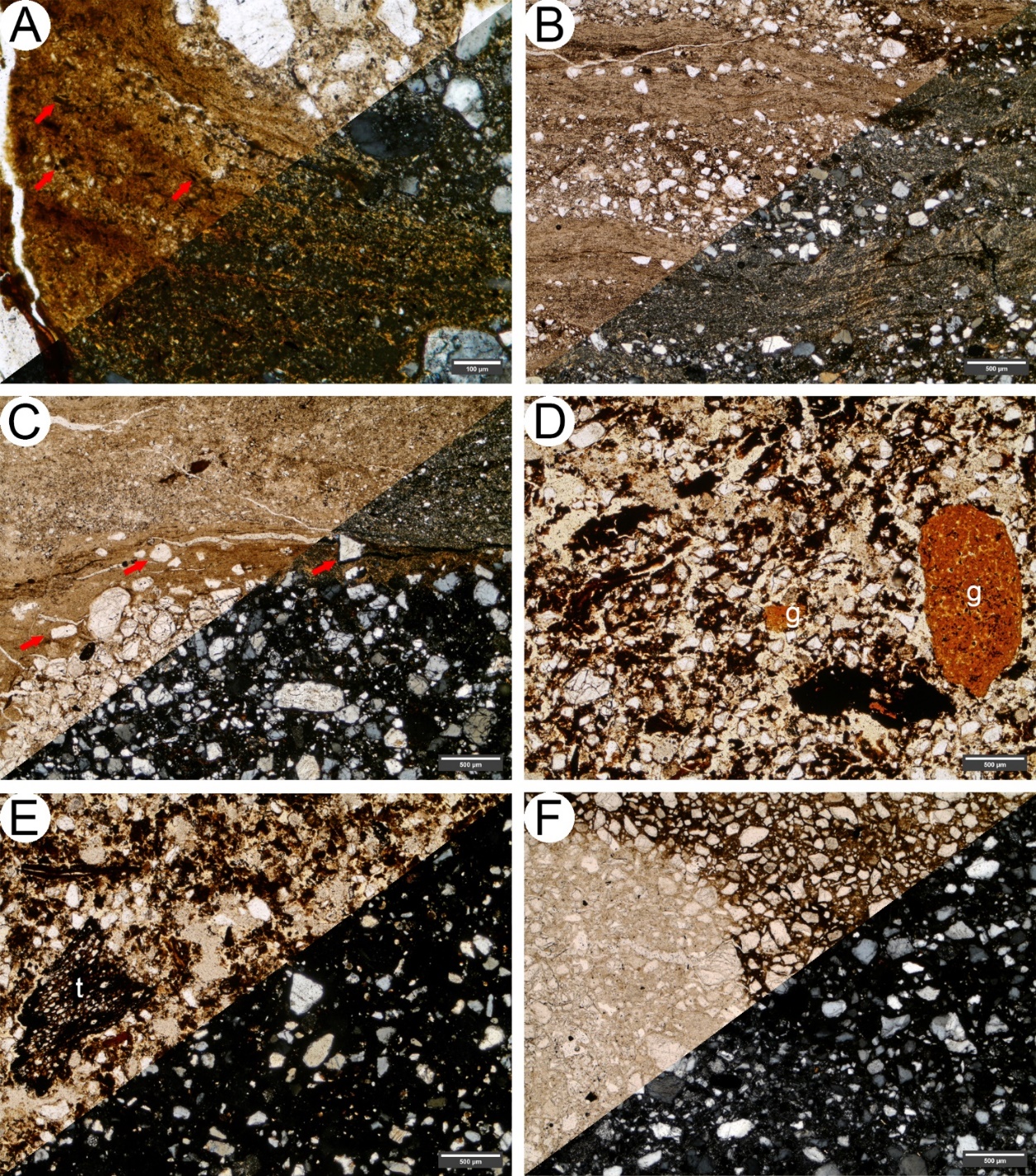


Fig. S15. Photomicrographs of Area 7 thin sections**.** (A) LBCSS. Large infilling of clay with poor continuous orientation, interlayered with fine silt. Minute fragments of charred AOM (red arrows) are common (left: PPL; right: XPL). (B) DBBPS/LBCSS boundary. Wavy lenses of loamy sand and clay (left: PPL; right: XPL). (C) DBBPS/LBCSS boundary. Quartz grains (red arrows) embedded in clay lamina after erosion from the underlying sandy layer (left: PPL; right: XPL). (D) DOSS. Fluffy sediment with many voids; poorly sorted and rather fine quartz skeleton and very abundant AOM. Common well-preserved fragments of vegetal tissue (t), variably humified (left: PPL; right: XPL). (E) DOSS. Crudely parallel oriented AOM aggregates (upper left), with few remains of vegetal cells. Reddish glass clasts (g) are common (PPL). (F) DOSS/LBCSS boundary, strongly impregnated dark brown Fe/Mn-oxides obscure the limit between units (left: PPL; right: XPL).


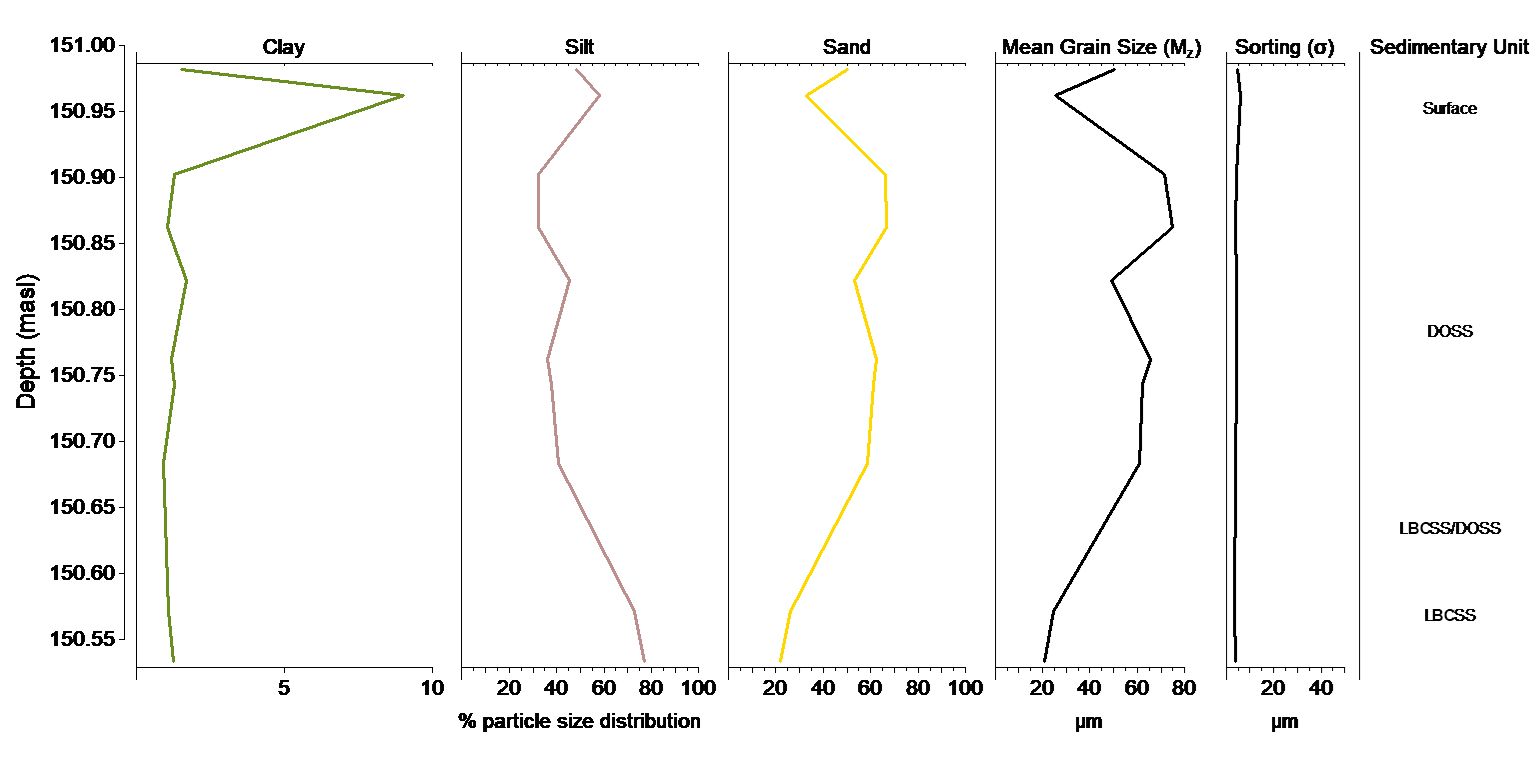


Fig. S16. Particle size analysis for samples from LBCSS and DOSS, as percentages of clay, silt, and sand. Mean grain size and sorting based on geometric Folk and Ward ^24^ graphical measures.

2.3 Site Formation Summary

The depositional history of the Area 7 spring can be hypothesised as follows:

1. GH5: Deposition of DBBPS (margins of spring centre, null/low energy, formation of turf).

2.1. GH4: Erosion of DBBPS (moderate energy, partial collapse of DBBPS).

2.2. GH4: Deposition of progradating LBCSS (margins of centre basin, low energy), and GBSS (towards centre of basin pool, forward of LBCSS, low/medium energy).

2.3 GH4: Drying up of the pan, wildfires (clay illuviation, charred AOM).

3.1 GH3: Northward shift of depositional centre and erosion of LBCSS.

3.2 GH3: Deposition of OBSC (centre basin, lower energy and shallower water) on top of GBSS.

3.3 GH3: Drying up of the pan, wildfires (clay illuviation, charred AOM).

3.4 GH3: Soil formation on top of OBSS.

3.5. GH3: Erosion of LBCSS (bank shaping at spring centre, same as in 2.1).

3.6. GH3: Shift southwards of depositional centre, shallow water, deposition of DOSS (centre basin facies, low-energy input of aged AOM from wildfires) and continued deposition of OBSS.

4.1. GH2: Minor erosional events.

4.2. GH2: Deposition of LPGSS (moderate energy, facies between basin centre and margin), deposition of vegetal residues (phytoliths).

5.1. GH1: Minor erosional events.

5.2. GH1: Deposition of LGSS (moderate energy, facies between basin centre and margin).

3. Luminescence Dating

The following sections provide addition sample information and luminescence methods, equivalent dose (D_e_) determination, single aliquot regenerative dose (SAR) D_e_ validation tests, pIR-IRSL anomalous fading tests, and D_e_ results and ages.

3.1 Sample information and Detailed Luminescence Methods

Sample ASP18-12 was collected from the lowermost exposed DBBPS layer in the Sector 1 Spring Eye Section, while six samples were taken from the overlying Acheulian-bearing GBSS and LBCSS layers in the Sector 1 North (ASP18-10), Sector 2 North (ASP18-5, ASP18-6), Sector 3 North (ASP19-12, ASP19-13) and Sector 4 South (ASP19-10) Sections. Two samples were collected from the MSA-bearing OBSS layer in the Sector 1 North Section (ASP18-11) and Sector 4 North Section (ASP18-9), with an additional two samples collected from the MSA-bearing DOSS layer in the Sector 2 and Sector 3 North Sections (ASP18-4, ASP19-11). The uppermost MSA-bearing layers (LPGSS and LGSS) were sampled for luminescence dating in the Sector 4 South Section (sample ASP19-6, ASP19-14) and Sector 4 North Section (sample ASP18-7).

K-feldspar (90–125 μm diameter) and quartz (212–250 μm diameter) grains were extracted from the unilluminated centres of the sample tubes under safe light conditions (630 nm LEDs, <0.15 μW/cm^2^ power density) and prepared for burial dose estimation using established procedures ^25^. The latter included etching by hydrofluoric (HF) acid to remove the alpha-irradiated external layers (10% HF digestion for 10 minutes to etch the K-feldspar fractions; 48% HF digestion for 40 min to etch the quartz fractions ^26,27^). The etched grains were subsequently washed in 30% hydrochloric acid to remove any precipitated fluorides, and re-sieved using a 63 µm sieve to eliminate any disaggregated grains. K-feldspar pIR-IRSL and quartz TT-OSL D_e_ measurements were made using the experimental apparatus and single aliquot regenerative dose (SAR) procedures described previously for the Area 1 and Area 2 samples by Herries et al. ^8^ and Caruana et al. ^9^. Multi-grain pIR-IRSL D_e_ measurements were made on 10–12 aliquots of K-feldspars per sample (each aliquot containing ~400 grains), while single-grain TT-OSL and OSL D_e_ measurements were made on 800–2000 individual quartz grains per sample. For TT-OSL and OSL D_e_ measurements, 212–250 μm quartz grains were measured in aluminium discs drilled with an array of 300 x 300 μm holes to ensure true single-grain resolution ^28^. Multi-grain K-feldspar dose-response curves were constructed from the first 10 s of each pIR-IRSL signal after subtracting a mean background count from the last 10 s of stimulation. Single-grain OSL and TT-OSL dose-response curves were determined using the first 0.08 s of each green laser stimulation after subtracting a mean background count obtained from the last 0.25 s of the signal.

Dose recovery tests were performed on samples ASP18-9, ASP18-12 and ASP19-6 to determine suitable preheating and measurement conditions for the OSL, TT-OSL and pIR-IRSL SAR protocols. A K-feldspar pIR-IRSL SAR protocol involving raised temperature pIR-IR stimulation at 250°C after a preheat of 280°C for 60s (herein denoted pIR-IRSL_250_) (Table S2) yielded an optimum dose recovery ratio in agreement with unity at 1σ (0.98 ± 0.02) for sample ASP18-9, and is therefore considered suitable for dating the Area 7 deposits. The optimum single-grain quartz OSL SAR protocol includes a preheat of 240°C for 10s prior to measurement of the natural (L_n_) and regenerative dose (L_x_) OSL signals, and a preheat of 200°C for 10s prior to measurement of the test dose (T_n_ and T_x_) OSL signals (Table S2). These preheating conditions yielded an accurate measured-to-given dose ratio of 0.98 ± 0.02, and an overdispersion value of 15 ± 2%, for a 160 Gy dose recovery test performed on individual grains of ASP19-6. The single-grain TT-OSL SAR protocol (Table S2) makes use of a TT-OSL test dose to correct for sensitivity change, four preheats of 260^o^C for 10s in each SAR cycle, and two high temperature OSL treatments to prevent TT-OSL signal carry over from previous L_x_ and T_x_ measurement steps. A weighted mean measured-to-given dose ratio of 1.00 ± 0.05 was obtained when applying this SAR procedure to sample ASP18-12, supporting the suitability of the TT-OSL D_e_ measurement conditions for dating purposes. The results of these dose recovery tests are consistent with those obtained previously for samples ASP18-13 and ASP18-16 from Area 1 ^8^ and sample ASP18-17 from Area 2 ^9^, confirming the broader applicability of the chosen OSL, TT-OSL and pIR-IRSL measurement conditions across different Amanzi Springs sites.

Individual and sample-averaged D_e_ estimates are presented with their 1σ uncertainties, which are derived from three sources of uncertainty: (i) a random uncertainty term arising from photon counting statistics for each OSL measurement, calculated using Eq. 3 of Galbraith ^29^; (ii) an empirically determined instrument reproducibility uncertainty of 0.6% for each multi-grain aliquot measurement and 1.5% for each single-grain measurement (calculated specifically for the Risø reader used for each sample, following the approach outlined in ^30^); and (iii) a dose-response curve fitting uncertainty determined using 1000 iterations of the Monte Carlo method described in Duller ^31^ and implemented in Analyst v4.

Anomalous fading tests were performed on K-feldspar aliquots that had been measured for D_e_ determination, following the procedures outlined by Auclair et al. ^32^ and Huntley and Lamothe ^33^. The combined weighted average anomalous fading rate (*g*-value; normalised to 2 days) for all measured aliquots (*n*=42) was 1.47 ± 0.06 %/decade. This empirical fading rate is consistent with published *g*-values for higher temperature pIR-IR_290_ signals (see summary in ^34^), as well as for athermally stable quartz OSL signals ^35^, and does not provide strong support for pIR-IRSL_250_ age correction. We therefore favour the uncorrected pIR-IRSL_250_ ages for chronological interpretations of the Area 7 sedimentary sequence; though fading corrected pIR-IRSL_250_ ages are also presented (Table S5) for comparative purposes. It is worth noting that the fading-corrected pIR-IRSL_250_ ages are statistically indistinguishable from the uncorrected pIR-IRSL_250_ age estimates at 2σ for all samples considered in this study. The consistency between the uncorrected pIR-IRSL_250_ ages and the replicate single-grain OSL ages for samples ASP19-6, ASP18-7 and ASP19-14 (Table 1) similarly supports our decision not to apply additional fading corrections in this study.

Dose rate evaluations have been undertaken using a combination of *in situ* gamma-ray spectrometry and low-level beta counting of dried and homogenised, bulk sediments collected directly from the luminescence sampling positions, as detailed in Table 1. Elemental concentrations of K, U, and Th were determined from the field gamma-ray spectra using the windows method described in Arnold et al. ^36^ and Duval and Arnold ^37^. These elemental concentrations were then used to calculate the gamma dose rates, enabling us to capture any spatial heterogeneity in the surrounding (~30 cm diameter) gamma radiation field of each sample. External beta dose rates were determined from measurements made using a Risø GM-25-5 beta counter ^38^. Background-subtracted count rates were measured for three aliquots of each sample and compared with net count rates obtained simultaneously for a loess sediment standard with known U, Th, and K concentrations ^39^. Cosmic-ray dose rates were calculated using the approach described in Prescott and Hutton^40^.

A small, assumed internal (alpha plus beta) dose rate of 0.03 ± 0.01 Gy / ka has been included in the final dose rate calculations for the quartz fractions of these samples, based on published ^238^U and ^232^Th measurements for etched quartz grains from a range of locations ^41–45^ and an alpha efficiency factor (a-value) of 0.04 ± 0.01 ^46,47^. Internal alpha and beta dose rate contributions for K-feldspar grains have also been calculated using previously published values for intrinsic ^238^U, ^232^Th, ^40^K, and ^87^Rb contents. Internal ^40^K and ^87^Rb concentrations were assumed to be 12.5 ± 0.5% ^48^ and 400 ± 100 ppm ^49^, respectively. Internal ^238^U and ^232^Th concentrations were assumed to be 0.15 ± 0.03 ppm and 0.35 ± 0.07 ppm, respectively, based on modal values obtained by Mejdahl ^41^ and similar values obtained by Huntley and Clague ^50^, Huntley and Lian ^51^, and Alappat et al. ^52^. An a-value of 0.09 ± 0.03 was used to estimate the internal alpha dose rate contributions from these ^238^U and ^232^Th concentrations, based on published estimates obtained for a range of K-feldspar samples ^46,53–57^.

Radionuclide concentrations and specific activities have been converted to dose rates using the conversion factors given in Readhead ^58^ and Guérin et al.^59^ (see footnotes of Table 1 for details), making allowance for beta-dose attenuation ^60,61^ and long-term sediment water contents ^62,63^. The present-day sediment water contents measured for the Area 7 samples, which ranged between 6 and 30% of dry sediment weight (mean = 14.6% dry weight), are not considered to be entirely representative of those prevailing throughout the long-term burial periods because: (i) the present-day water table level at Amanzi Springs Area 7 is artificially lower than the long-term average level owing to recent agricultural drainage and groundwater exploitation activities, (ii) some of the excavation pits and sediment exposures had partially dried out prior to sampling, and (iii) the luminescence dating samples were collected during the dry-season.

To determine more suitable long-term sediment moisture contents (and to ensure consistency in dose rate calculations across the various Amanzi Springs sites), we have followed the approach outlined by Caruana et al. ^9^ for samples collected within or directly adjacent to the spring eye, and the recommendations of Herries et al. ^8^ for samples collected further away from the spring eye or from the margins of the spring basin. Specifically, the long-term water content of sample ASP18-12 collected from the deepest layer of GH5 (DBBPS) within the spring eye section of Sector 1 is taken as 100% of the saturated water content estimate as these deposits would have effectively remained beneath the water table for the duration of their burial period. The long-term water content of all other samples collected from spring margins deposits or located further away from the spring eye are taken as 70% of the saturated water content as they would have been located above (albeit close to) the long-term permanent water table. For the latter samples, a suitable empirical long-term water content estimate has been derived from proportional saturated water contents assessments undertaken by Herries et al. ^8^ on freshly exposed deposits in comparative spring margin contexts at Area 1. By establishing suitable estimates of present-day water content (as a proportion of sediment saturation capacity) in the absence of significant desiccation effects prior to sampling, and factoring in the potential for higher long-term groundwater levels, as well as intermittent surface flooding and reactivation of the spring eye during past periods of higher sea level (as indicated by micromorphology analysis), Herries et al. ^8^ suggested that conservative long-term sediment moisture contents equivalent to 70% of present-day saturated water contents should be adopted for luminescence dating samples collected adjacent to the Amanzi Hill spring eyes. Additional sensitivity tests performed by Herries et al. ^8^ and Caruana et al. ^9^ revealed that the luminescence ages calculated using this approach are relatively insensitive to the specific choice of long-term water content when considering alternative hydrological scenarios between reasonable end-member limits. For example, use of lower long-term sediment moisture contents equivalent to 60% of present-day saturated values or adopting higher long-term sediment moisture contents equivalent to 80% of present-day saturated values only changed the final TT-OSL and pIR-IRSL ages of the Area 1 and 2 samples by 2–16 ka. Applying an extreme (unrealistic) long-term water content equivalent to 100% of the saturated water value for spring margin deposits at Area 2 only increased the final TT-OSL and pIR-IRSL ages by 29–49 ka. For each of the alternative long-term water content scenarios tested, the resultant ages were not statistically different at 2σ from those originally calculated using 70% present-day saturated values.

Using the approaches of ^8,9^, the long-term sediment moisture contents for the Area 7 luminescence dating samples range between 15 and 47% of dry sediment weight (mean = 28.5% dry weight) (Table 1). This mean value is consistent at 2σ with the long-term average water content adopted for Area 2 luminescence dating samples (27.8%) collected from a similar mixture of spring eye and spring margin deposits. The long-term moisture content values of the Area 7 samples have been assigned a fixed 1σ uncertainty of ±5% (±10% at 2σ) to accommodate any variations in hydrologic conditions during burial.

3.2 Equivalent dose (D_e_) determination

Quartz TT-OSL and OSL measurements and K-feldspar pIR-IRSL measurements were made on a Risø TL/OSL-DA-20 reader equipped with a ^90^Sr/^90^Y β radiation source and an Electron Tubes PDM 9107B photomultiplier tube. K-feldspar signals were stimulated using IR diodes (850 nm, maximum power of 340 mW/cm^2^) at 60% power and blue emissions were detected with a 2 mm-thick Schott BG39 and 3 mm-thick Schott BG3 filter pack. Single-grain quartz TT-OSL and OSL signals were stimulated with a 10 mW Nd:YVO4 single-grain laser attachment emitting at 532 nm (maximum power of ~50 W cm^2^). Additional multiple-grain quartz OSL measurements (i.e., the OSL wash steps in Table S2 were made using blue LEDs (470 nm, maximum power 102 mW/cm^2^) at 90% power. Quartz TT-OSL and OSL emissions were detected in the ultraviolet region using a 7.5 mm-thick U340 filter. The mounted ^90^Sr/^90^Y beta source on the Risø TL/OSL-DA-20 reader has been calibrated to administer known doses to multiple-grain aliquots and single-grain discs. For single-grain measurements, spatial variations in beta dose rates across the disc plane were taken into account by undertaking hole-specific calibrations using gamma-irradiated quartz ^64^.

The single-grain TT-OSL, single-grain OSL, and multi-grain pIR-IRSL SAR protocols adopted in this study are summarised in Table S2 and follow those reliably employed at Amanzi Springs Area 1 and Area 2 ^8,9^. Individual D_e_ values were only included in the final age calculation if they satisfied a series of quality-assurance criteria, as detailed in Demuro et al.^65^, Arnold et al. ^66^, and Méndez-Quintas et al. ^67^ (SOM Table S2). Single-grain TT-OSL and OSL D_e_ estimates were rejected from further consideration if they exhibited one or more of the following properties: (i) weak OSL signals (i.e., the net intensity of the natural test-dose signal, T_n_, was less than three times the standard deviation of the late-light background signal); (ii) poor recycling ratios (i.e., the ratios of sensitivity-corrected luminescence response (L_x_/T_x_) for two identical regenerative doses were not consistent with unity at 2σ); (iii) high levels of signal recuperation (i.e., the sensitivity-corrected luminescence response of the 0 Gy regenerative-dose point amounted to more than 5% of the sensitivity-corrected natural signal response (L_n_/T_n_) at 2σ); (iv) poorly defined or non-monotonic dose-response curves (i.e., those displaying no discernible dose-response curve, or a zero [flat] or negative response with increasing dose) and dose-response curves displaying very scattered L_x_/T_x_ values (i.e., those that could not be successfully fitted with the Monte Carlo procedure and, hence, did not yield finite D_e_ values and uncertainty ranges). Grains failing this quality assurance criterion primarily displayed dim signals and hence their L_x_/T_x_ uncertainties were too large for meaningful dose-response curve construction or finite D_e_ evaluation. This criterion is therefore functionally equivalent to criterion (v) of Demuro et al. (2019; i.e., net T_n_ signal has a relative error of >30%) but it additionally captures grains with ill-suited dose-response properties; (v) saturated or non-intersecting natural signals (i.e., L_n_/T_n_ values equal to, or greater than, the I_max_ saturation limit of the dose-response curve at 2σ); (vi) contamination by feldspar grains or inclusions (i.e., the ratio of the L_x_/T_x_ values obtained from two identical regenerative doses measured with and without prior IR stimulation (OSL IR depletion ratio^68^) was less than unity at 2σ).

Additionally, single-grain TT-OSL D_e_ estimates were rejected from further consideration if they exhibited: (vii) slowly decaying signals that correspond to slow OSL component carry-over from previous OSL stimulation rather than genuine thermal transfer of charge into the fast OSL trap (i.e., the TT-OSL Fast Ratio [FR^69^] was less than a minimum acceptance threshold of 20^8^). For this purpose, the FR has been calculated as per Herries et al. ^8^, i.e., by comparing the counts in the initial channel (0.017 seconds) of the TT-OSL decay curve (L_1_) with those in the middle part of the decay (average counts over 1.0–1.2 seconds; L_2_) after subtracting a late light background count from the last 0.15 s (L_3_), according to the equation (L_1_-L_3_)/(L_2_-L_3_). The FR has also been calculated using the highest regenerative dose TT-OSL signal for each grain in order to maximise signal-to-noise ratios and capture any progressive sensitisation or build-up of interfering, slowly bleaching OSL signals through the SAR procedure.

TT-OSL SAR quality assurance (vii) was included in this study because Herries et al. ^8^ and Caruana et al. ^9^ found that up to 4% of the initially accepted grain populations from the Area 1 and Area 2 luminescence samples exhibited non-negligible, slow-decaying TT-OSL signals (i.e., their T_x_ signals did not reach background after 2 seconds of laser stimulation). Further examination showed that the signals of these grains did not originate from genuine thermal transfer of charge into the fast OSL trap, but rather they corresponded to interfering (non-transferred) slow OSL components from the previous OSL stimulation, which had not reached background levels prior to commencing the TT-OSL measurements. Grains displaying such slow-dominated OSL signals may not fulfill basic SAR suitability requirements ^70^, and they have been shown to be associated with thermally unstable signals, experimentally sensitised components, or unreliable TT-OSL D_e_ estimates in several samples ^65,71–74^. Sensitivity tests involving the application of increasingly stringent FR thresholds to the accepted TT-OSL D_e_ datasets revealed a noticeable influence on weighted mean D_e_ and overdispersion for the Area 1 and Area 2 samples. In general, the samples showed a ~12–20% increase in weighted mean D_e_ and a 9–40% decrease in overdispersion when applying incrementally higher FR acceptance thresholds between 0 and 20. Use of more stringent FR acceptance ratios >20 had no further discernible effect on D_e_ or overdispersion, other than causing a significant reduction in the number of accepted grains. Application of a FR acceptance threshold of ≥20 resulted in the elimination of all slowly decaying grain populations with low and outlying D_e_ values, with dose-recovery tests confirming that these subsets of slowly decaying grains are poorly suited to the TT-OSL SAR protocol ^8,9^.

The TT-OSL and OSL grain classification statistics obtained for each sample after applying these quality assurance criteria are summarised in Table S3. The suitability of including these TT-OSL and OSL SAR quality assurance criteria is supported by the results of dose recovery tests performed on samples ASP18-12 and ASP19-6 (see next section). Multi-grain aliquot pIR-IRSL measurements were subject to the same quality assurance criteria as the TT-OSL and OSL measurements, with the exception of criteria (vi) and (vii). All but 3 of the 159 measured K-feldspar aliquots passed these quality assurance criteria and were included in the final D_e_ analysis (Table 1).

Fig. S17 shows representative pIR-IRSL_250_, single-grain TT-OSL, and single-grain OSL decay and dose response curves for aliquots / grains that passed the SAR quality assurance criteria and were used for dating. The pIR-IRSL_250_ decay curves typically decrease by ~90% within the first 30 s of stimulation and are optimally fitted with a single saturating exponential plus linear function. The majority of accepted quartz grains display rapidly decaying TT-OSL and OSL curves (reaching background levels within 0.5 s), which are characteristic of quartz signals dominated by the most readily bleachable (so-called ‘fast’) OSL component (Fig. S17 – compare OSL decay curve shape for a fast-dominated Risø calibration quartz grain^64^). The single-grain OSL and TT-OSL dose-response curves are all well-represented by a single saturating exponential function.

3.3 Single aliquot regenerative dose (SAR) D_e_ validation tests

A series of dose-recovery tests were undertaken to assess the suitability of the SAR procedures for OSL, TT-OSL and pIR-IRSL D_e_ determination. To determine the most suitable pIR-IRSL measurement and preheat conditions for the Area 7 samples, we undertook dose recovery tests using the pIR-IRSL_250_ SAR protocol shown in Table S2, as well as alternative procedures involving a preheat of 250 ^o^C for 60 s and stimulation temperature of 225 ^o^C (pIR-IRSL_225_), a preheat of 300 ^o^C for 60 s and stimulation temperature of 270 ^o^C (pIR-IRSL_270_), and a preheat of 320 ^o^C for 60 s and stimulation temperature of 290 ^o^C (pIR-IRSL_290_), as detailed in Méndez-Quintas et al.^67^. Twenty four aliquots of sample ASP18-9 (each containing ~400 grains) were prepared and placed under direct sunlight for 8 h to bleach their naturally accumulated pIR-IRSL signals. These bleached aliquots were then split into four batches (*n* = 6 each) for the pIR-IRSL_225_, pIR-IRSL_250_, pIR-IRSL_270_ and pIR-IRSL_290_ dose recovery tests. For each batch of aliquots, three were left un-dosed to determine the residual (unbleached) D_e_ remaining after daylight bleaching, while the remaining three were given a laboratory dose of 594 Gy. The four batches of six aliquots were then separately measured with the pIR-IRSL_225_, pIR-IRSL_250_, pIR-IRSL_270_ and pIR-IRSL_290_ SAR protocols to determine their D_e_ values. Dose recovery (recovered-to-given dose) ratios were calculated after subtracting the residual (unbleached) D_e_ of the un-dosed aliquots from the mean D_e_ obtained from the dosed aliquots.

The pIR-IRSL_250_ dose recovery ratio for sample ASP18-9 overlaps with unity at 1σ (0.98 ± 0.02) and supports the suitability of this SAR protocol for D_e_ determination (Table S4). In contrast, the pIR-IRSL_270_ and pIR-IRSL_290_ signals systematically overestimate the administered dose and yield inaccurate dose recovery ratios at 2σ (1.07 ± 0.03 and 1.19 ± 0.05, respectively), while the pIR-IRSL_225_ signal slightly underestimates the administered dose at 2σ (0.95 ± 0.02) (Table S4). On the basis of these dose recovery assessments, we have chosen to use the pIR-IRSL_250_ protocol for dating purposes. The weighted mean pIR-IRSL_250_ residual dose recorded for the three un-dosed aliquots of ASP18-9 after 8 h of daylight bleaching was 19.9 ± 1.1 Gy. Taking into consideration the limited bleaching time used in the dose recovery test, this relatively low residual D_e_ value provides reasonable support for the bleachability of the pIR-IRSL_250_ signals, and suggest that any thermal transfer of charge during preheating ^75,76^ or photo-transfer of electrons to low temperature TL traps during prior IR stimulation ^77^ is not likely to contribute significantly to the natural D_e_ values of these samples.

The suitability of the OSL D_e_ determination procedure (Table S2) was initially evaluated using multi-grain aliquot dose-recovery tests to ascertain optimal preheating conditions for bulk grain populations. These tests were performed on ~180-grain aliquots of sample ASP19-6 using a series of different regenerative dose preheat (PH_1_) conditions (ranging between 200 ^o^C for 10 s and 260 ^o^C for 10 s) and different test dose preheat (PH_2_) combinations (160 ^o^C for 10 s, 200 ^o^C for 10 s or 220 ^o^C for 10 s). A known laboratory dose of 60 Gy was applied to groups of 3-4 aliquots after optically bleaching their natural OSL signals using two 1,000 s blue LED stimulations separated by a 10,000 s pause (to ensure complete decay of any phototransferred charge in the 110 ^o^C TL trap). The administered dose was treated as a surrogate natural dose and subsequently measured using a multiple-grain version of the SAR sequence shown in Table S2, which involved replacing 125°C green laser stimulations with 125°C blue LED stimulations for 60 s, and inserting a 50°C IR bleach for 40 s prior to each OSL measurement to remove any feldspar signal contamination. Fig. S18a summarises the results of the multi-grain aliquot dose-recovery tests performed on sample ASP19-6. The most suitable dose-recovery results were obtained using a PH_1_ of 240 ^o^C for 10 s and a PH_2_ of 200 ^o^C for 10 s. This preheat combination yielded a weighted mean measured-to-given dose ratio of 1.01 ± 0.02, low inter-aliquot D_e_ scatter, low-dose and high-dose mean recycling ratios in closest agreement with unity (1.00 ± 0.01 and 1.01 ± 0.01, respectively) and a mean recuperation ratio of less than 1%.

To confirm the suitability of the OSL SAR procedure at the single-grain scale and over a higher dose range, we repeated the dose-recovery test on 600 individual quartz grains from sample ASP19-6 using the optimum multiple-grain preheat conditions (PH_1_ = 240 ^o^C for 10 s; PH_2_ of 200 ^o^C for 10 s). A dose of 160 Gy was administered to these quartz grains after bleaching their natural signals using the same procedure described above. 17% of measured grains (*n* = 100) satisfied the SAR quality assurance criteria and were included in the final D_e_ analysis (Table S3). The single-grain OSL dose recovery tests yielded a mean recovered-to-given dose ratio of 0.98 ± 0.02 and an overdispersion value of 15 ± 2% (Fig. S18b). These dose-recovery results support the general suitability of the SAR procedure and quality-assurance criteria for single-grain D_e_ estimation at Area 7. They also provide a minimum estimate of the intrinsic single-grain D_e_ scatter and overdispersion that is expected to originate from the laboratory procedures themselves, and from grain-to-grain variations in luminescence responses to the fixed SAR conditions.

A single-grain TT-OSL dose-recovery test was also undertaken on sample ASP18-12 to assess the suitability of the chosen SAR protocol shown in Table S2. The dose-recovery test was performed by adding a dose of 459 Gy on top of the naturally accumulated dose for a subset of 700 grains. This approach was adopted owing to the long durations of light exposure needed to bleach natural TT-OSL signals down to low residual levels ^65,78^. The dose recovery ratio was calculated by first adding the weighted mean natural TT-OSL D_e_ of sample ASP18-12 (i.e., 486 ± 23 Gy, as shown in Table 1 and determined from 1000 grains) to the weighted mean administered dose of the accepted grain population from the dose recovery test (459 ± 9 Gy). This was used to constrain the combined administered (natural + laboratory) dose being assessed in the dose recovery test. The measured (recovered) weighted mean D_e_ of the unbleached and dosed grains in the dose recovery test (947 ± 36 Gy; Fig. S18c) was then divided by the combined administered (natural + laboratory) dose (945 ± 25 Gy) to derive the measured-to-given dose ratio. A weighted mean TT-OSL measured-to-given dose ratio of 1.00 ± 0.05 was obtained using this approach, which is within 1σ of unity and supports the suitability of the chosen measurement conditions and SAR quality assurance criteria (including use of a FR acceptance threshold of ≥20) for final age determination.

3.4 pIR-IRSL anomalous fading tests

Anomalous fading assessments were performed on subsets of aliquots from each sample to investigate the potential for long-term athermal loss of K-feldspar pIR-IRSL_250_ signals over burial timescales. Anomalous fading measurements were made on aliquots that had previously been used to derive D_e_ values following the procedures of Auclair et al. ^32^, which involved undertaking repeated SAR L_x_/T_x_ measurements after different storage times of 0.2–30 hours. Anomalous fading rates (g-value) normalised to two days were calculated as described in Huntley and Lamothe ^33^ and used to quantify the expected percentage of signal loss per decade of storage time.

The long-term (athermal) stability assessments undertaken on the Area 7 K-feldspar fractions yielded weighted-mean g-values (normalized to 2 days) ranging between 0.9 ± 0.2 %/decade and 1.8 ± 0.2 %/decade per sample (Table S5), with a combined weighted average g-value of 1.5 ± 0.1 %/decade for all measured aliquots (*n* = 42). These empirical fading rates are in agreement with those obtained for the Area 1 samples ^8^ (weighted average g-value = 1.4 ± 0.1 %/decade) and Area 2 samples ^9^ (weighted average g-value = 1.5 ± 0.1 %/decade). They are also consistent with published *g*-values for higher temperature pIR-IRSL signals (e.g., pIR-IRSL_290_ signals involving pIR-IRSL measurements at 290 °C following a preheat of 320 °C for 60 s; see summary in ^34^). Such low g-values (on the order of <1–2%/decade) have been interpreted to be potentially unreliable indicators of long-term fading rates and / or artifacts of laboratory procedures on the basis of comparisons made with independent age control, observations of natural signal saturation, and measurements of similarly sized g-values for quartz ^35,79,80^ (see discussions in ^34^). Consequently, we do not consider the low g-values recorded in the present study to be indicative of the need for additional pIR-IRSL_250_ age corrections, though fading corrected pIR-IRSL ages are also presented in Table S5 for comparative purposes. The fading corrected pIR-IRSL ages of all fourteen samples are consistent with the original uncorrected pIR-IRSL age estimates at 1σ. The consistency between the uncorrected pIR-IRSL_250_ ages and the replicate single-grain OSL ages for samples ASP19-6, ASP18-7 and ASP19-14 (Table 1) similarly supports our decision not to apply additional fading corrections in this study.

3.5 D_e_ results and ages

Table 1 summarises the environmental dose rates, D_e_ values and final ages obtained for the fourteen luminescence dating samples from Area 7. The single-grain TT-OSL, single-grain OSL and multi-grain pIR-IRSL D_e_ distributions of each sample are also shown as radial plots in Fig. S19 and S20. In general, the D_e_ distributions of the Area 7 samples are consistent with those of well-bleached, unmixed sediments, with the exception of two single-grain TT-OSL D_e_ datasets (samples ASP18-7 and ASP18-9) that exhibit more pronounced scatter and enhanced tails of high D_e_ values.

The pIR-IRSL D_e_ datasets have low overdispersion values ranging between 7 ± 1% and 21 ± 4% (Table 1) and are not considered to be significantly skewed according to the multi-grain version of the weighted skewness test outlined by Arnold et al. ^81^ and Arnold and Roberts ^82,83^. The three OSL D_e_ datasets from the LGSS and LPGSS samples are characterised by low dose dispersion that is reasonably well-represented by the weighted mean value (as indicated by the large proportions of grains lying within the 2σ grey bands in Fig. S20), and overdispersion of 29 ± 2% to 31 ± 4% (Table 1). The overdispersion values for these samples are broadly similar to those reported for well-bleached and unmixed single-grain OSL D_e_ datasets ^66,82,84–87^. None of the single-grain OSL D_e_ datasets are considered to be significantly positively skewed according to the weighted skewness test outlined by Bailey and Arnold ^88^ and Arnold and Roberts ^83^. Application of the maximum log likelihood (*L_max_*) test ^89^ indicates that the central age model (CAM) is statistically favoured over the three- or four-parameter minimum age models (MAM-3 or MAM-4) of Galbraith et al. ^90^ for all three D_e_ datasets.

Twelve of the fourteen samples (ASP18-4 to -6, ASP-10 to -12; ASP19-6, ASP19-10 to -14) exhibit relatively homogeneous single-grain TT-OSL D_e_ distributions characterised by low to moderate overdispersion values of 20 ± 5% to 36 ± 4% (Table 1), and D_e_ scatter that is generally well-represented by the weighted mean value (Fig. S19). The TT-OSL overdispersion values for these samples are consistent with those reported elsewhere for ideal (well-bleached and unmixed) single-grain TT-OSL D_e_ datasets at 2σ (e.g., the average overdispersion values of 21 ± 2% presented by ^78^, 2019 and 33 ± 2% presented by ^84^), including those from similar well-bleached deposits across Area 1 (overdispersion values of 25–39% ^8^) and Area 2 (overdispersion values of 19–39% ^9^). These single-grain TT-OSL D_e_ datasets also do not display prominent leading-edges of low D_e_ values or elongated tails of higher D_e_ values, and they are not considered to be significantly positively skewed according to the weighted skewness test. Additionally, they are optimally represented by the CAM over the MAM-3 or MAM-4 when applying the *L_max_* test of Arnold et al. ^89^. Collectively, these single-grain TT-OSL D_e_ characteristics, mirror the pIR-IRSL and OSL datasets, and suggest that the samples do not suffer from major extrinsic D_e_ scatter related to insufficient bleaching prior to burial ^83,91^ or widespread post-depositional sediment mixing between units ^36,92^. The single-grain TT-OSL ages for these twelve samples, together with the single-grain OSL and multi-grain pIR-IRSL ages for all measured samples, have therefore been obtained using the weighted mean (CAM) D_e_ estimate, in accordance with their *L_max_* test results ^89^ (Table 1).

The remaining two samples (ASP18-7 and ASP18-9 from the LPGSS and OBSS deposits) exhibit more heterogeneous single-grain TT-OSL D_e_ distributions characterised by larger proportions of individual D_e_ values lying outside of the weighted mean burial dose 2σ ranges, overdispersion ranges of 35 ± 4% to 48 ± 5%, and more distinct leading-edges of low D_e_ values or tails of higher D_e_ values (Fig. S19, Table 1). The single-grain D_e_ datasets are additionally considered to be significantly positively skewed, and the MAM-3 is statistically favoured over the CAM for both samples when applying the *L_max_* test. These D_e_ characteristics are consistent with those commonly reported for heterogeneously bleached single-grain OSL and TT-OSL samples ^78,81,88,93,94^, which seems reasonable in this sedimentary context given the host deposits formed via groundwater discharge that could have involved limited transportation distances or indirect / filtered daylight exposures in spring-margin and subaqueous pond settings.

Additional insights into the bleaching adequacy of the ASP18-7 and ASP18-9 TT-OSL datasets can be gained by comparing the weighted mean D_e_ values of the various luminescence dating signals measured for these samples. Given that OSL, pIR-IRSL and TT-OSL signals bleach at different rates in natural daylight (e.g., ^65,78,95–97^), parity in quartz and K-feldspar luminescence ages would suggest that these sample were exposed to sufficient daylight to reset their residual doses prior to burial. If this was not the case, the comparative luminescence dating signals would yield markedly different weighted mean ages on account of their distinctly different bleaching sensitivities. The CAM ages for the single-grain OSL and pIR-IRSL datasets of ASP18-7 are consistent with each other at 1σ (143.8 ± 11.3 ka and 148.8 ± 12.2 ka, respectively; Table 1) according to the *X*^2^ homogeneity test outlined by Galbraith ^98^, providing support for adequate resetting of these two luminescence signals. In contrast, the TT-OSL CAM age of ASP18-7 is ~50 ka older than its OSL and pIR-IRSL counterparts (194.8 ± 15.5 ka) and is not consistent with either corresponding age at 2σ according to the *X*^2^ homogeneity test, suggesting that the more slowly bleaching TT-OSL signals were not adequately reset prior to burial for at least some of the measured grain populations. A statistically significant age offset is also apparent for sample ASP18-9 according to the *X*^2^ homogeneity test, with the slower bleaching TT-OSL signal yielding a weighted mean age that is ~89 ka older than the corresponding age obtained with the more rapidly bleaching pIR-IRSL signal (278.7 ± 24.6 ka versus 189.5 ± 10.5).

The systematically older weighted mean TT-OSL ages obtained for ASP18-7 and ASP18-9 relative to their pIR-IRSL and OSL counterparts confirms that the enhanced scatter observed across these two TT-OSL D_e_ datasets likely arises from the presence of heterogeneously bleached grain populations related to the relatively slow bleaching characteristics of TT-OSL signals (e.g., ^65,97^). For these two samples, the final TT-OSL ages have therefore been derived using the MAM-3 (the statistically favoured age model according to the *L_max_* test of ^89^) rather than the CAM in order to isolate burial dose estimates from the well-bleached portion of grains in each D_e_ dataset (e.g., ^78,88,89^).

The bleaching adequacy of the pIR-IRSL signals for the Area 7 samples is further supported by the low residual dose recorded in the dose recovery test. A weighted mean pIR-IRSL residual D_e_ of 19.9 ± 1.1 Gy was recorded for sample ASP18-9 after 8 hours of daylight bleaching, which would yield a corresponding residual age of 9.5 ± 0.7 ka. This residual D_e_ is consistent with the low values recorded for sample ASP18-17 from Area 2 after 10 hours of daylight bleaching 19.5 ± 4.1 Gy ^9^) and for sample ASP18-13 from Area 1 following 8 h of daylight bleaching (22.6 ± 2.3 Gy ^8^). The corresponding residual age of 9.5 ± 0.7 ka calculated for ASP18-9 also lies well within the existing 1σ uncertainty of the final pIR-IRSL age estimate for this sample (189.5 ± 10.5 ka; Table 1). Taking into consideration the relatively small size of the empirical residual D_e_ value, the insensitivity of the final age of ASP18-9 to this residual dose estimate, and the potential that natural bleaching durations may have significantly exceeded the 8 hours of experimental bleaching durations employed in the dose recovery experiment, we have not applied an additional residual dose subtraction to the final pIR-IRSL age estimates.

The final ages calculated for the Area 7 luminescence dating samples using the statistically favoured burial dose models (i.e., those selected in accordance with the *Lmax* test results) are summarised in Table 1. The agreement between the replicate luminescence ages for each sample obtained using different signals (OSL, pIR-IRSL, TT-OSL), different mineralogies (quartz and K-feldspar), and different scales of D_e_ analysis (single-grain and multiple-grain aliquots), provides good support for the reliability of the dating procedures employed at this site. All fourteen samples exhibit stratigraphically consistent and internally consistent replicate TT-OSL and pIR-IRSL ages, confirming the suitability of our age model selections and D_e_ dataset interpretations. Similarly, the consistency of the replicate single-grain OSL ages for samples ASP19-6, ASP18-7 and ASP19-14 confirms that the OSL D_e_ estimates from the uppermost MSA layers (LGSS, LPGSS) have not been negatively affected by dose saturation effects, all things being equal. The TT-OSL and pIR-IRSL signals of these samples exhibit significantly higher characteristic saturation doses than their OSL signal counterparts (e.g. Fig. S17), and thus provide useful inter-comparisons of OSL dating reliability in the absence of any potential dose saturation effects.


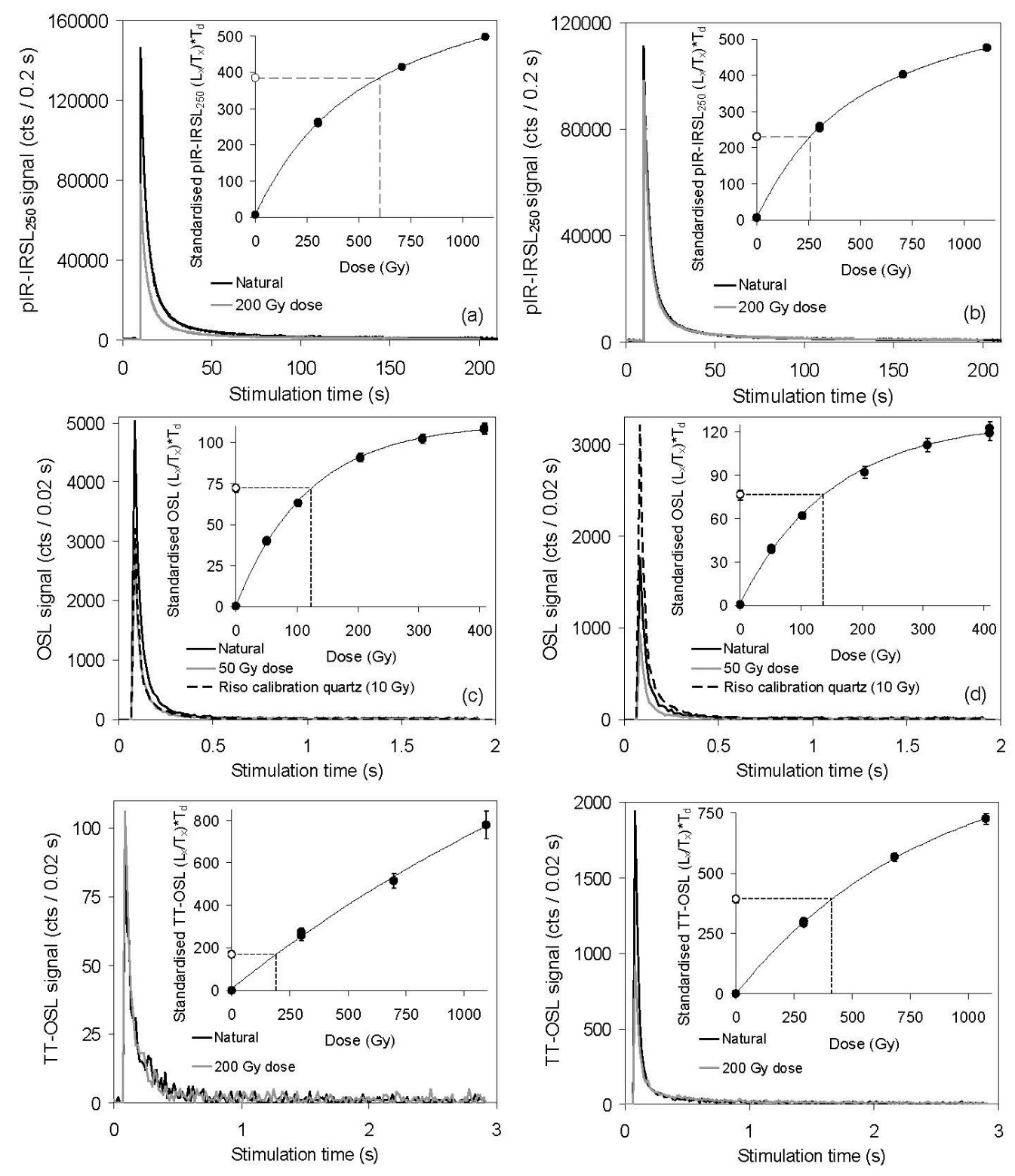


Fig. S17. Representative K-feldspar pIR-IRSL250, single-grain quartz OSL and single-grain quartz TT-OSL decay / dose-response curves**.** (a)-(b) ~400-grain K-feldspar aliquots of sample ASP19-10 and ASP19-10, respectively. (c)-(d) Individual quartz grains from ASP19-6 with bright and moderately bright OSL signals, typical decay shapes and saturating exponential dose-response curve functions. The OSL decay curve of a fast-component dominated calibration quartz grain is shown in this plot for comparison (Risø calibration quartz from Rømø, batch #98 ^64^). (e)-(f) Individual quartz grains from sample ASP19-14 and ASP19-13, respectively, with typical TT-OSL signals, decay shapes and saturating exponential dose-response curves.


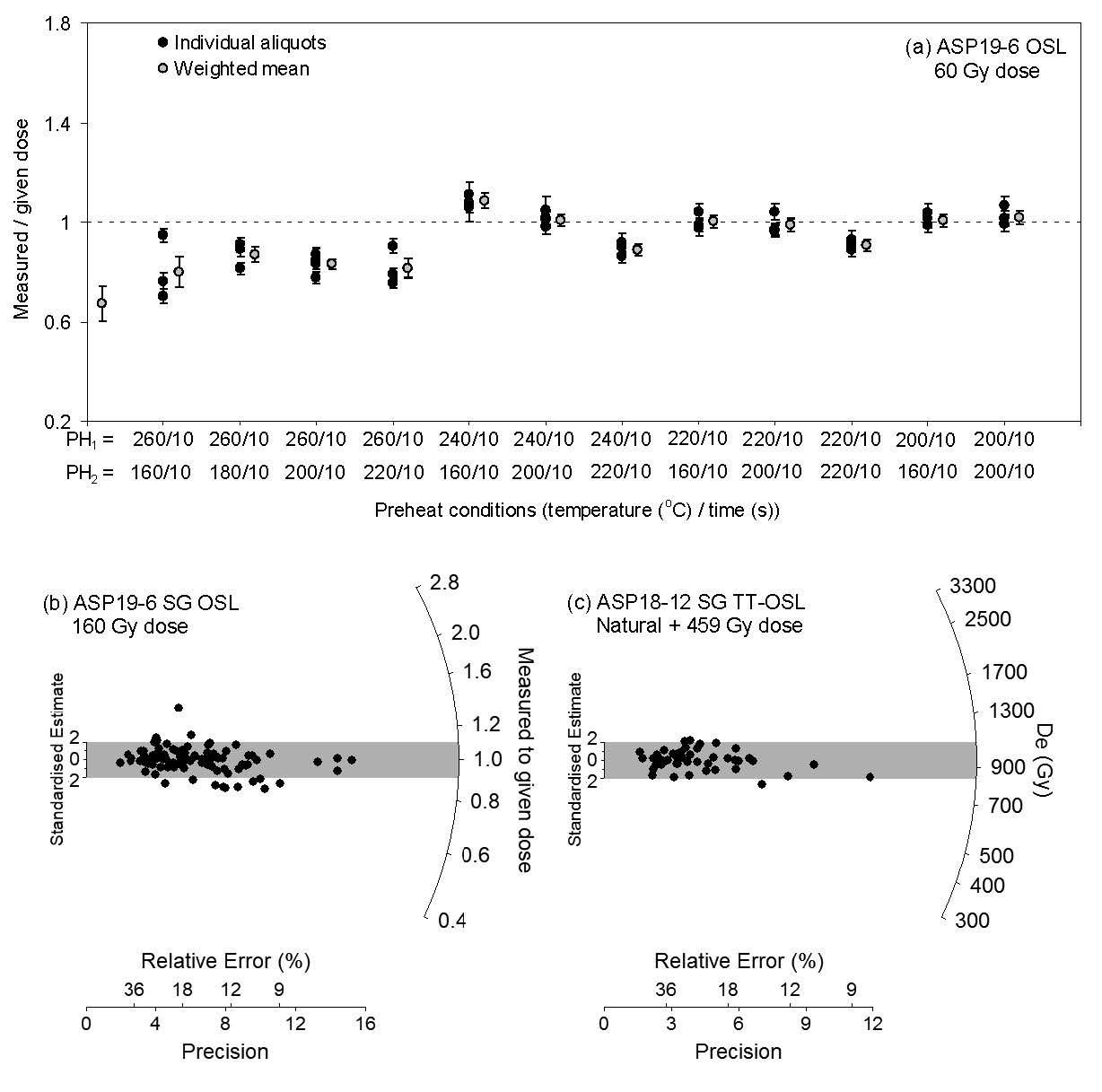


Fig. S18. Dose-recovery test results for samples ASP18-9, ASP18-12 and ASP19-6. (a) Measured-to-given dose OSL ratios versus regenerative dose preheat (PH_1_) and test dose preheat (PH_2_) temperature (held for 10 s) for ~180-grain aliquots of sample ASP19-6. (c) Radial plot showing the measured-to-given dose OSL ratios obtained for individual quartz grains of sample ASP19-6 in the single-grain SAR dose-recovery test. The grey shaded region is centred on the administered dose for each grain (sample average = 160 Gy). (d) Radial plot showing the dose-recovery test (natural + dosed) TT-OSL D_e_ values obtained for sample ASP19-12. The grey band is centred on the CAM D_e_ value obtained using accepted grains.

**
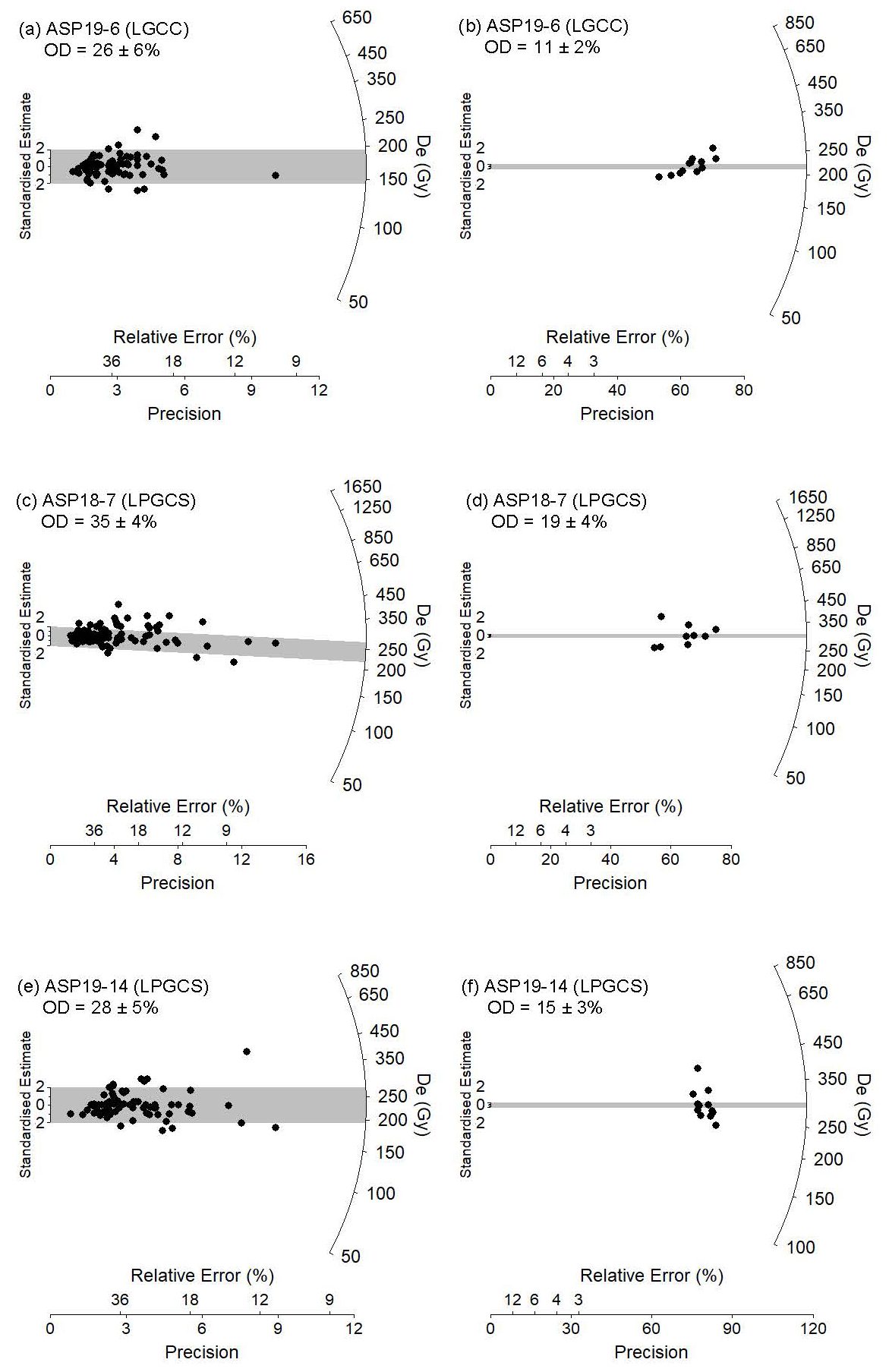

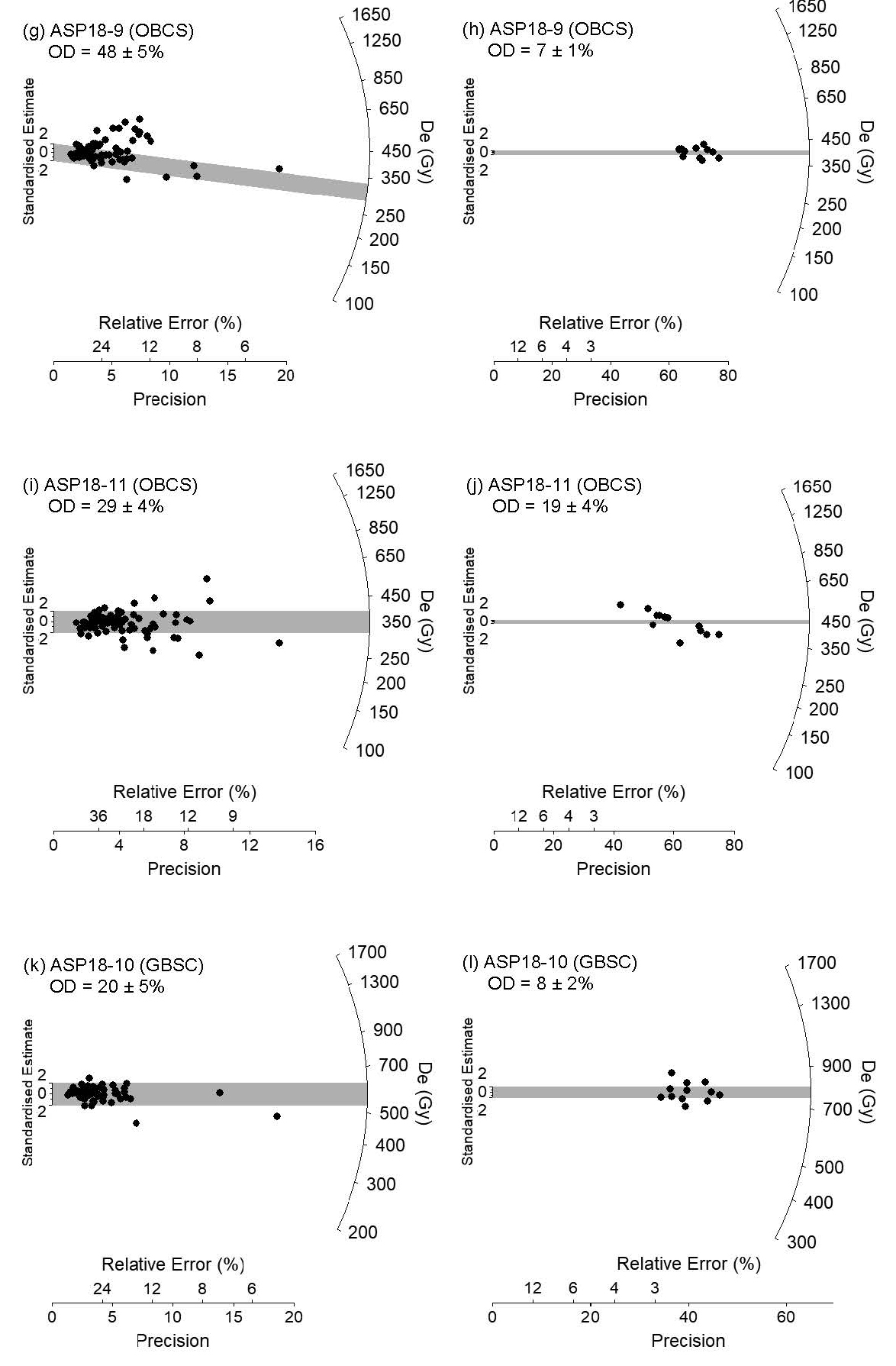

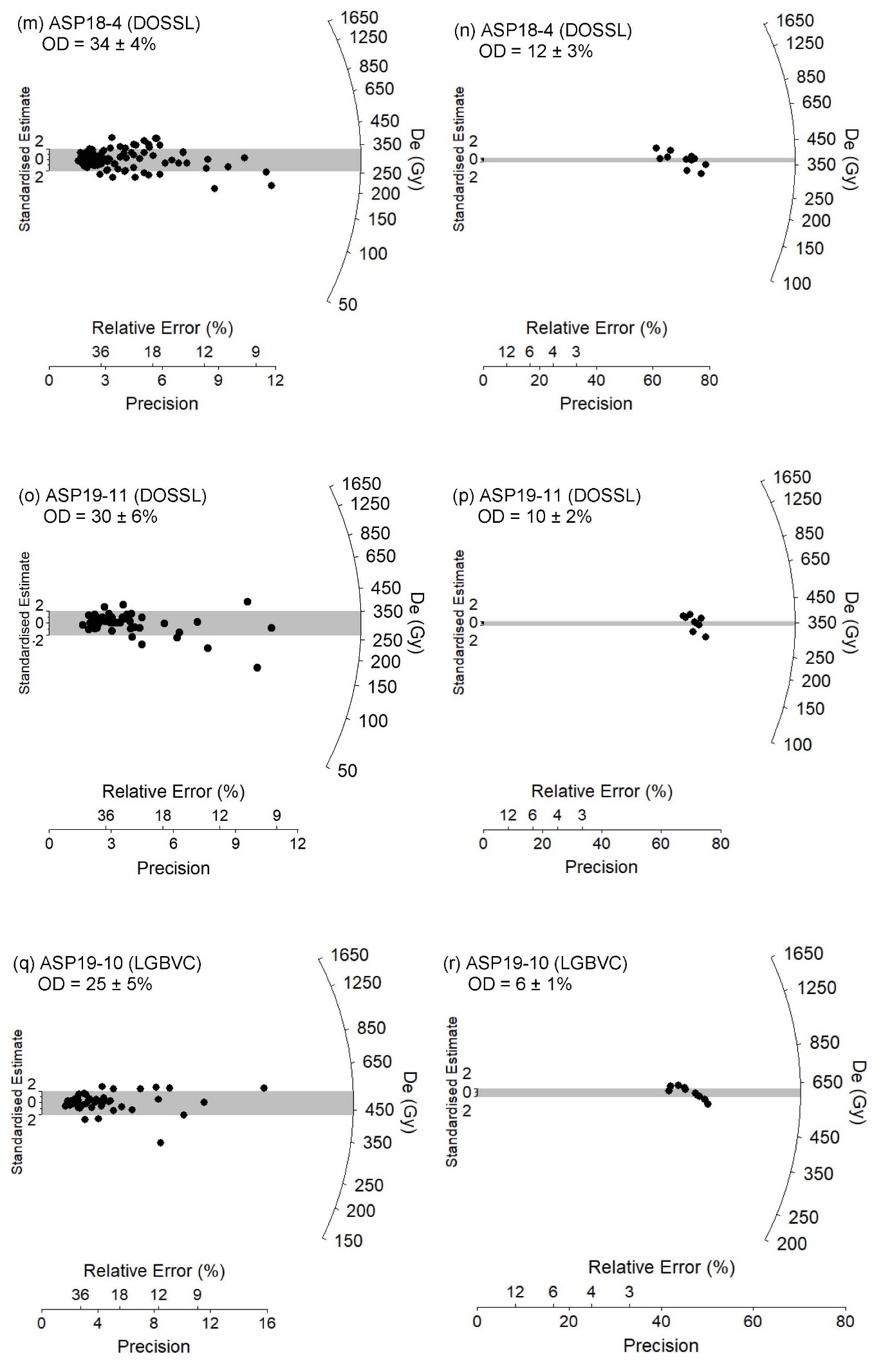

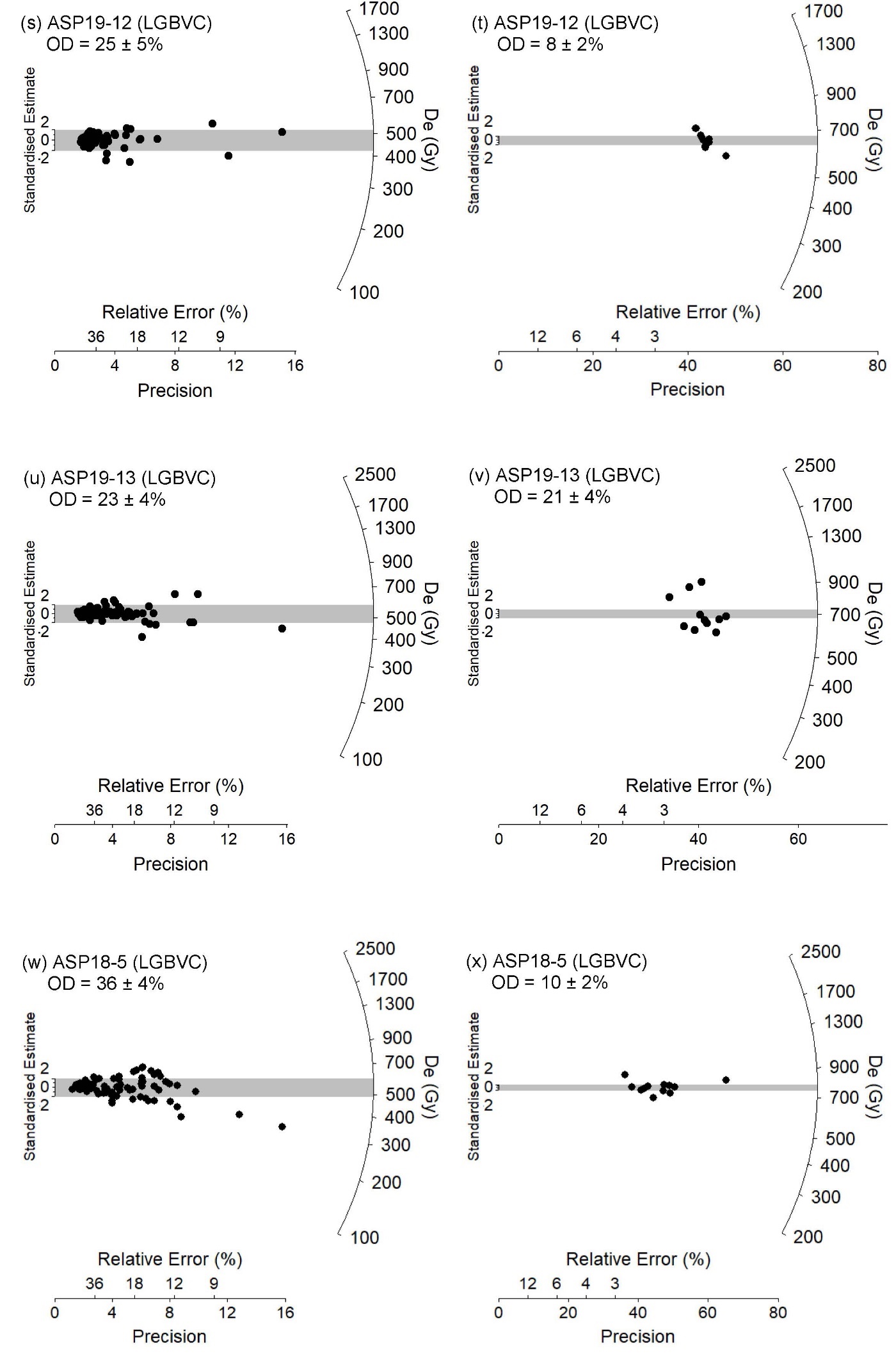

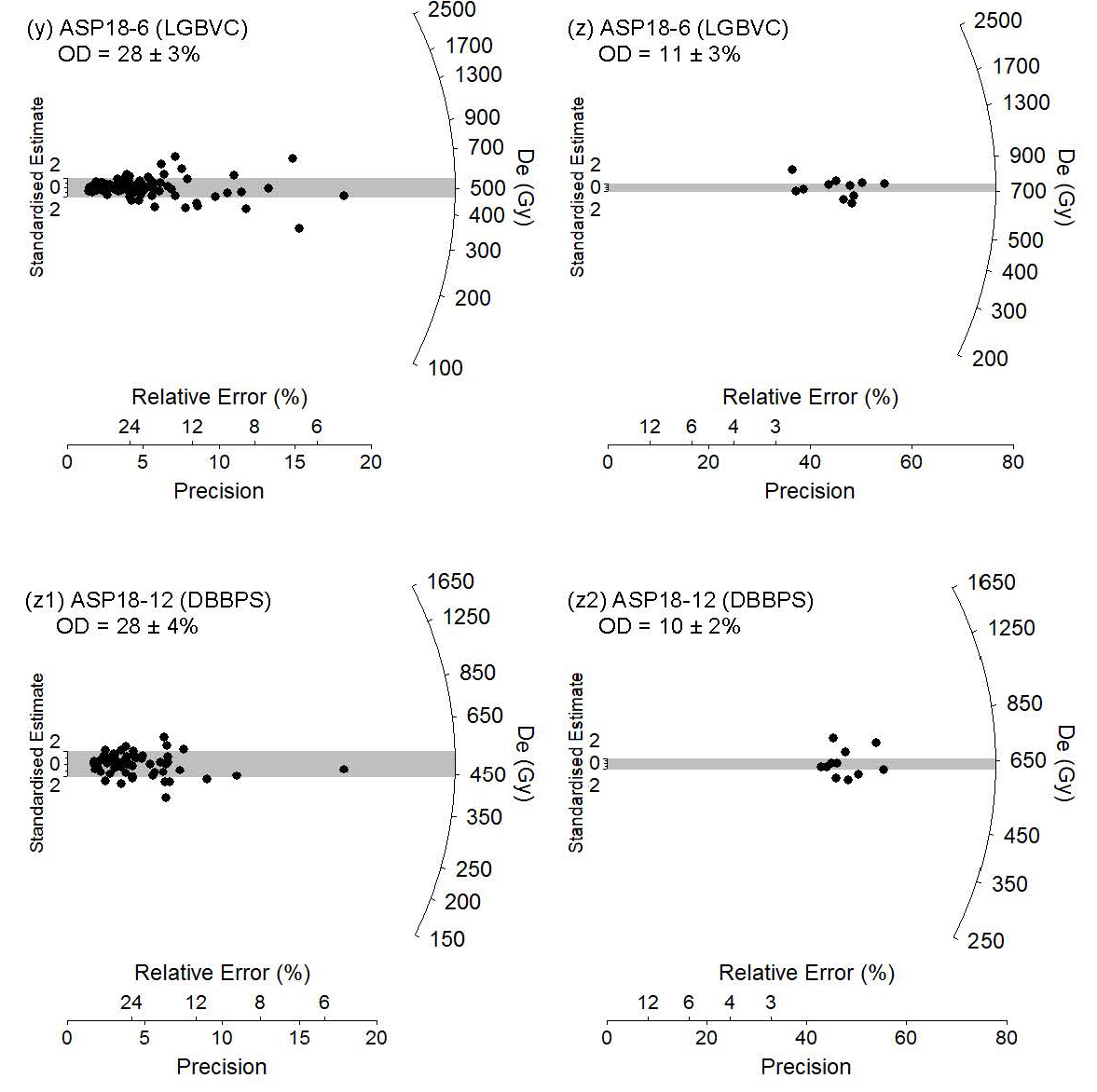
**

Fig. S19. Single-grain TT-OSL De distributions (left-hand plots) and multi-grain pIR-IRSL De distributions (right-hand plots) **for samples collected from Area 7**, shown as radial plots. The shaded bands are centred on the D_e_ values used for the age calculations, which have been derived using the 3-parameter minimum age model ^90^ for the single-grain TT-OSL D_e_ datasets of samples ASP18-7 and ASP18-9, and the central age model ^90^ for all other D_e_ datasets.


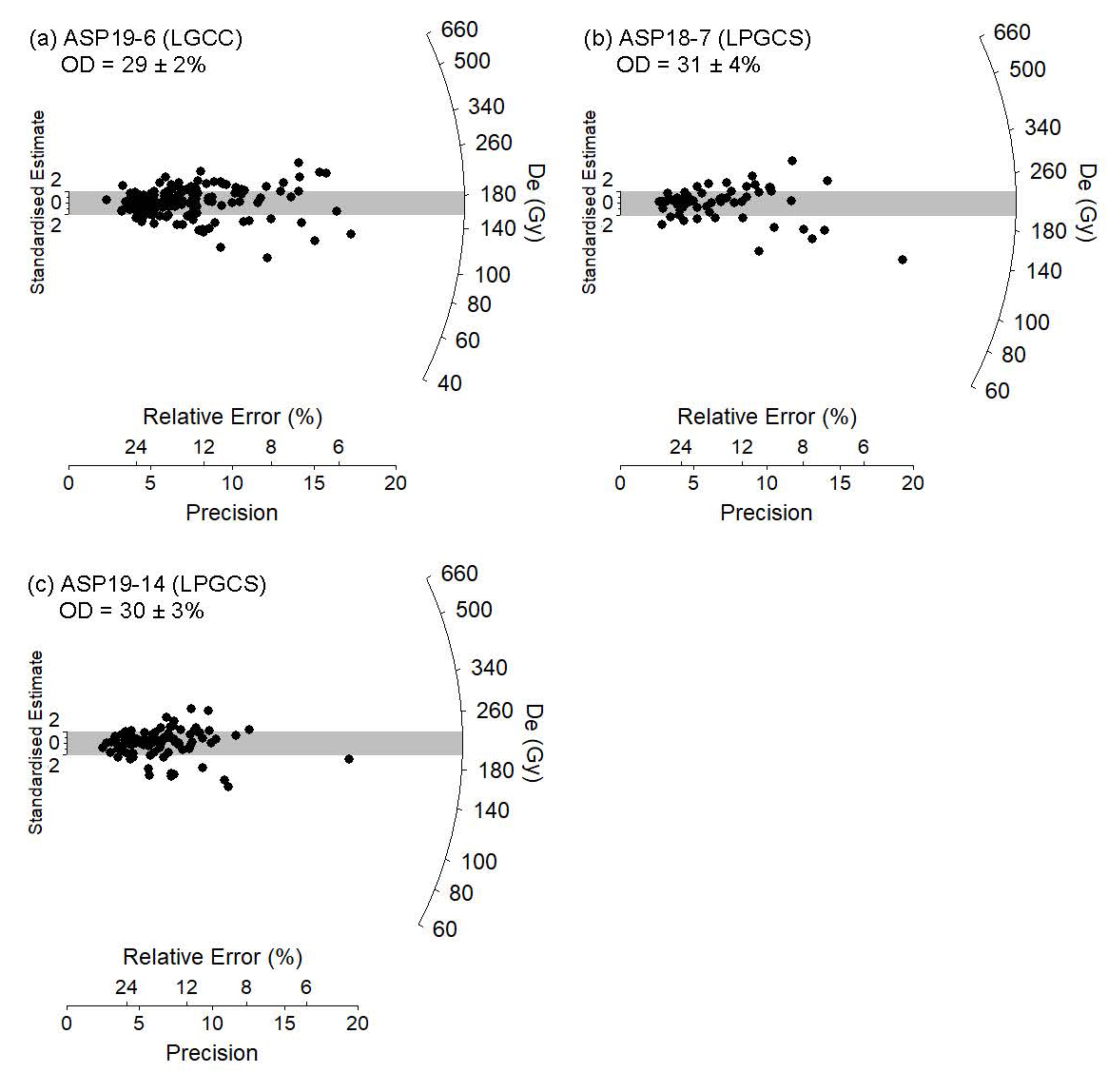


Fig. S20. Single-grain OSL De distributions for samples collected from the uppermost MSA layers, shown as radial plots. The shaded bands are centred on the D_e_ values used for the age calculations, which have been derived using the central age model ^90^.

Table S2. Single-aliquot regenerative-dose (SAR) protocols used to measure multiple-grain K-feldspar pIR-IRSL_250_, single-grain TT-OSL, and single-grain OSL D_e_ values. Each SAR measurement cycle was repeated for the natural dose, three to five different sized regenerative doses, a 0 Gy regenerative-dose (to measure signal recuperation) and a replicate of the first regenerative-dose cycle (to assess the suitability of the test-dose sensitivity correction). In the case of the single-grain OSL SAR procedure, both the smallest and largest non-zero Gy regenerative dose cycles were repeated at the end of the SAR procedure to assess the suitability of the test dose sensitivity correction over different dose ranges. The smallest regenerative-dose cycle was then repeated a second time with the inclusion of step 2 to check for the presence of feldspar contaminants using the OSL IR depletion ratio of Duller ^68^. L_x_ = regenerative dose signal response; L_n_ = natural dose signal response; T_x_ = test dose signal response for a laboratory dose cycle; T_n_ = test dose signal response for the natural dose cycle.

| **Step** | **pIR-IRSL_250_ SAR** | **Signal** | **Step** | **Single-grain TT-OSL SAR** | **Signal** | **Step** | **Single-grain OSL SAR** | **Signal** |  |
| --- | --- | --- | --- | --- | --- | --- | --- | --- | --- |
| 1 | Dose (natural or laboratory) |  | 1 | Dose (natural or laboratory) |  | 1 | Dose (natural or laboratory) |  |  |
| 2 | Preheat 1 (280ºC for 60 s) |  | 2 | Preheat 1 (260ºC for 10 s) |  | 2^a^ | IR stimulation (50ºC for 60 s) |  |  |
| 3 | IR stimulation (50ºC for 200 s) |  | 3 | SG OSL stimulation (green laser; 125ºC for 2 s) |  | 3 | Preheat 1 (240ºC for 10 s) |  |  |
| 4 | pIR-IR stimulation (250ºC for 200 s) | L_x_ or L_n_ | 4 | Preheat 2 (260ºC for 10 s) |  | 4 | SG OSL stimulation (green laser; 125ºC for 2 s) | L_x_ or L_n_ |  |
| 5 | Test dose (200 Gy) |  | 5 | SG OSL stimulation (green laser; 125ºC for 3 s) | L_x_ or L_n_ | 5 | Test dose (15 Gy) |  |  |
| 6 | Preheat 2 (280ºC for 60 s) |  | 6 | OSL wash (blue LEDS; 280ºC for 400 s) |  | 6 | Preheat 2 (200ºC for 10 s) |  |  |
| 7 | IR stimulation (50ºC for 200 s) |  | 7 | Test dose (200 Gy) |  | 7 | SG OSL stimulation (green laser; 125ºC for 2 s) | T_n_ or T_x_ |  |
| 8 | pIR-IR stimulation (250ºC for 200 s) | T_n_ or T_x_ | 8 | Preheat 3 (260ºC for 10 s) |  | 8 | Repeat measurement cycle for different sized |  |  |
| 9 | IR wash (at 290ºC for 100 s) |  | 9 | SG OSL stimulation (green laser; 125ºC for 2 s) |  |  | regenerative doses |  |  |
| 10 | Repeat measurement cycle for |  | 10 | Preheat 4 (260ºC for 10 s) |  |  |  |  |  |
|  | different sized regenerative doses |  | 11 | SG OSL stimulation (green laser; 125ºC for 3 s) | T_n_ or T_x_ |  |  |  |  |
|  |  |  | 12 | OSL wash (blue LEDS; 290ºC for 400 s) |  |  |  |  |  |
|  |  |  | 13 | Repeat measurement cycle for different sized |  |  |  |  |  |
|  |  |  |  | regenerative doses |  |  |  |  |  |

^a^ Step 2 is only included in the single-grain OSL SAR procedure when measuring the OSL IR depletion ratio ^68^.

Table S3. Single-grain TT-OSL and OSL classification statistics for the dose recovery and natural D_e_ measurements of the Area 7 luminescence samples. The proportion of grains that were rejected from final D_e_ estimation after applying the various SAR quality assurance criteria are shown in rows 5-13. These criteria were applied to each single-grain measurement in the order listed. T_n_ = natural test dose signal response; L_n_/T_n_ = sensitivity-corrected natural signal response; L_x_/T_x_ = sensitivity-corrected regenerative-dose signal response; *I_max_* = saturation OSL intensity of the fitted dose response curve.

| **Sample name** | **ASP19-6** | **ASP19-6** | **ASP19-6** | **ASP18-7** | **ASP18-7** | **ASP19-14** | **ASP19-14** | **ASP18-9** | **ASP18-11** |
| --- | --- | --- | --- | --- | --- | --- | --- | --- | --- |
| **SAR measurement type** | TT-OSL D_e_ | OSL D_e_ | OSL  Dose recovery | TT-OSL D_e_ | OSL D_e_ | TT-OSL D_e_ | OSL D_e_ | TT-OSL D_e_ | TT-OSL D_e_ |
| **Total measured grains (*n*)** | 1000 | 800 | 600 | 1000 | 800 | 1000 | 800 | 1000 | 1000 |
| **Grains rejected for failing SAR quality assurance criteria (%)** |  |  |  |  |  |  |  |  |  |
| T_n_ <3*σ* background | 59 | 16 | 26 | 61 | 37 | 64 | 16 | 68 | 58 |
| Low-dose recycling ratio ≠ 1 at ±2*σ* | 9 | 12 | 10 | 6 | 8 | 7 | 11 | 5 | 5 |
| High-dose recycling ratio ≠ 1 at ±2*σ* | - | 6 | 6 | - | 6 | - | 8 | - | - |
| OSL-IR depletion ratio <1 at ±2σ | 0 | 3 | 4 | <1 | 3 | 0 | 3 | 0 | 0 |
| 0 Gy L_x_/T_x_ >5% L_n_/T_n_ | 0 | 1 | 1 | 0 | 1 | 0 | 1 | 0 | 0 |
| Non-intersecting grains (L_n_/T_n_ > dose response curve saturation) | 0 | 5 | 2 | 0 | 4 | <1 | 7 | 0 | <1 |
| Saturated grains (L_n_/T_n_ ≥ dose response curve *I_max_* at ±2σ) | 0 | 5 | 4 | 0 | 4 | 0 | 3 | 0 | 0 |
| Anomalous dose response / unable to perform Monte Carlo fit | 22 | 32 | 30 | 19 | 29 | 21 | 39 | 17 | 27 |
| Slowly decaying signals ( L_x_ Fast Ratio <20) | 2 | - | - | 4 | - | 1 | - | 2 | 2 |
| **Sum of rejected grains (%)** | 92 | 80 | 83 | 90 | 92 | 93 | 88 | 92 | 92 |
| **Sum of accepted grains (%)** | 8 | 20 | 17 | 10 | 8 | 7 | 12 | 8 | 8 |

Table S3. Continued.

| **Sample name** | **ASP18-10** | **ASP18-4** | **ASP19-11** | **ASP19-10** | **ASP19-12** | **ASP19-13** | **ASP18-5** | **ASP18-6** | **ASP18-12** | **ASP18-12** |
| --- | --- | --- | --- | --- | --- | --- | --- | --- | --- | --- |
| **SAR measurement type** | TT-OSL D_e_ | TT-OSL D_e_ | TT-OSL D_e_ | TT-OSL D_e_ | OSL D_e_ | TT-OSL D_e_ | TT-OSL D_e_ | TT-OSL D_e_ | TT-OSL D_e_ | TT-OSL  dose recovery |
| **Total measured grains (*n*)** | 1000 | 1000 | 2000 | 1000 | 1000 | 1000 | 1000 | 1000 | 1000 | 700 |
| **Grains rejected for failing SAR quality assurance criteria (%)** |  |  |  |  |  |  |  |  |  |  |
| T_n_ <3*σ* background | 67 | 64 | 64 | 66 | 68 | 57 | 70 | 57 | 70 | 68 |
| Low-dose recycling ratio ≠ 1 at ±2*σ* | 4 | 5 | 5 | 4 | 5 | 13 | 3 | 5 | 4 | 4 |
| High-dose recycling ratio ≠ 1 at ±2*σ* | - | - | - | - | - | - | - | - | - | - |
| OSL-IR depletion ratio <1 at ±2σ | 0 | 0 | 0 | 0 | 0 | 0 | 0 | 0 | <1 | 0 |
| 0 Gy L_x_/T_x_ >5% L_n_/T_n_ | 0 | <1 | 0 | 0 | 0 | 1 | 0 | 0 | 0 | 0 |
| Non-intersecting grains (L_n_/T_n_ > dose response curve saturation) | <1 | <1 | <1 | <1 | <1 | <1 | <1 | <1 | <1 | <1 |
| Saturated grains (L_n_/T_n_ ≥ dose response curve *I_max_* at ±2σ) | 0 | 0 | <1 | 0 | 0 | 0 | 0 | 0 | 0 | <1 |
| Anomalous dose response / unable to perform Monte Carlo fit | 19 | 17 | 27 | 21 | 20 | 19 | 16 | 22 | 16 | 16 |
| Slowly decaying signals ( L_x_ Fast Ratio <20) | 2 | 4 | 1 | 2 | 1 | 2 | 2 | 5 | 4 | 4 |
| **Sum of rejected grains (%)** | 93 | 90 | 97 | 95 | 94 | 92 | 91 | 89 | 94 | 93 |
| **Sum of accepted grains (%)** | 7 | 10 | 3 | 5 | 6 | 8 | 9 | 11 | 7 | 7 |

Table S4. Summary of the K-feldspar pIR-IRSL dose recovery results obtained for sample ASP18-9 using different SAR D_e_ estimation protocols.

|  | **SAR D_e_ measurement protocol** | | | |
| --- | --- | --- | --- | --- |
| **Dose recovery results and statistics** | **pIR-IRSL_225_** | **pIR-IRSL_250_** | **pIR-IRSL_270_** | **pIR-IRSL_290_** |
| (i) Residual dose (Gy) (*n* = 3) ^a^ | 15.6 ± 1.4 | 19.9 ± 1.1 | 26.3 ± 1.8 | 30.0 ± 2.0 |
| (ii) Given dose (Gy) (*n* = 3) | 594 ± 12 | 594 ± 12 | 594 ± 12 | 594 ± 12 |
| (iii) Recovered dose (Gy) (*n* = 3) | 582 ± 6 | 603 ± 6 | 661 ± 27 | 737 ± 24 |
| (iv) Residual-subtracted recovered dose (iii-i) | 567 ± 7 | 583 ± 6 | 634 ± 8 | 707 ± 24 |
| (v) Residual-subtracted recovered-to-given dose ratio (iv / ii) | 0.95 ± 0.02 | 0.98 ± 0.02 | 1.07 ± 0.03 | 1.19 ± 0.05 |
| (vi) Mean recycling ratio (*n* = 6) ^b^ | 1.00 ± 0.01 | 1.00 ± 0.01 | 0.99 ± 0.01 | 1.00 ± 0.01 |

^a^ Mean residual dose remaining after 8 hrs of bleaching in direct sunlight.

^b^ Ratio of L_x_ / T_x_ values obtained for two identical regenerative doses measured in the second and final cycles of the SAR protocol.

Table S5. K-feldspar pIR-IRSL_250_ empirical fading rates (g-values), uncorrected ages and fading-corrected ages for the Area 7 luminescence samples.

| **Sample** | **Fading rate (*g_2days_*)**  **(% / decade) ^a^** | **Uncorrected age**  **(ka) ^b, c^** | **Fading-corrected**  **age (ka) ^b, c^** |
| --- | --- | --- | --- |
| ASP19-6 | 1.33 ± 0.25 | 94.5 ± 6.3 | 106.7 ± 7.6 |
| ASP18-7 | 1.17 ± 0.20 | 148.8 ± 12.2 | 165.9 ± 14.0 |
| ASP19-14 | 1.41 ± 0.05 | 151.2 ± 10.5 | 172.3 ± 12.0 |
| ASP18-9 | 1.46 ± 0.11 | 189.5 ± 10.9 | 217.5 ± 12.7 |
| ASP18-11 | 1.38 ± 0.27 | 224.1 ± 17.2 | 255.0 ± 20.8 |
| ASP18-10 | 0.94 ± 0.19 | 360.0 ± 21.4 | 393.0 ± 24.5 |
| ASP18-4 | 1.61 ± 0.17 | 178.9 ± 11.8 | 208.5 ± 14.2 |
| ASP19-11 | 1.78 ± 0.15 | 171.5 ± 10.9 | 202.8 ± 13.3 |
| ASP19-10 | 1.62 ± 0.15 | 306.2 ± 17.1 | 357 ± 20.8 |
| ASP19-12 | 1.61 ± 0.10 | 352.2 ± 21.3 | 410.5 ± 25.2 |
| ASP19-13 | 1.44 ± 0.33 | 354.9 ± 28.8 | 406.3 ± 35.7 |
| ASP18-5 | 1.34 ± 0.12 | 371.4 ± 23.0 | 421.7 ± 26.7 |
| ASP18-6 | 1.56 ± 0.36 | 368.9 ± 23.9 | 428.2 ± 32.2 |
| ASP18-12 | 1.64 ± 0.10 | 369.5 ± 22.2 | 433.3 ± 26.4 |

^a^ Laboratory fading rates were measured following the procedure suggested by Auclair et al. ^32^. The g-values were determined from repeated L_x_/T_x_ measurements made after different storage times (ranging from 0.2–30 hours) using Eq. 4 of Huntley and Lamothe ^33^ and have been normalized to a measurement delay time of two days (g_2days_) to enable direct comparisons with published values.

^b^ The final pIR-IRSL_250_ ages have been calculated without applying an additional empirical fading correction owing to the low g-values recorded for these samples (see main text). Fading corrected pIR-IRSL_250_ ages are presented in the final column for comparative purposes only.

^c^ Mean ± total uncertainty (68% confidence interval), calculated as the quadratic sum of the random and systematic uncertainties. Total uncertainty includes a systematic component of ±2% associated with laboratory beta-source calibration.

4. Palaeoecology

4.1 Previous Palaeoenvironmental Research at Amanzi Springs

Macroscopic plant remains and pollens were recovered during excavations at Amanzi Springs by Inskeep ^1^ and later Deacon ^2,3^. While these studies were focussed on Areas 1 and 2, given the proximity of these springs to Area 7 and the overall similarities in hydrological, sedimentological, and depositional environments, the available data is of relevance to the present study and is summarised below.

The geochemical conditions of the saturated spring deposits were favourable to the preservation of plant material, and seeds, thorns, wood fragments, fruits/fruit segments were identified. Despite the potential for recovering plant material, conditions were not uniform, and preservation was found to be better in the lower deposits. Pollen samples were collected from Area 1 (cutting 1, western section wall) by Inskeep ^1^, who reported that pollens of the Cyperaceae family of sedges, dicotyledonous flowering plants of the family Compositae, the Leguminosae (Fabaceae) family of herbaceous plants, and the Gramineae family of grasses were identified. Pollen counts were low in the sandy facies, and absent from the upper deposits where oxidisation had occurred ^2^. High pollen counts were present in samples from the BHS and GBS layers ^8,9^, reportedly dominated by “waters-edge plants”, with sedges and fern spores present in high frequencies, along with low counts of arboreal pollens ^2^.

Preserved wood fragments and other plant macrofossils were recovered during excavations in both Areas 1 and 2, with most of the wood found in the Rietheuval Member in Area 1. Inskeep ^1^ noted that the lower units in his Area 1 test excavation (cutting 1, units 1/1a, corresponding to Deacon’s Rietheuval Member) were rich in organic material, with a “large quantity” of wood, varying from <2.5cm to ~20cm in diameter, collected. Several pieces of wood were found to have dried out and shrunk *in situ*, leaving an impression in the sediment that preserved the original shape of the fragments, although no photos of these were included in the published report ^1^. Some wood samples appeared to have been burnt and looked charred, however again no further information was provided. The majority of the wood specimens recovered by Deacon ^2^ were reported to be in poor condition, with only a small proportion of the original cell structure remaining in most samples, limiting the potential for identification. Deacon ^2^ interpreted these to be the remains of woody plants growing around edges of the springs, either washed or blown into the spring deposits and accumulated amongst herbaceous plant communities around the spring vents. One wood fragment was reported as potentially worked; however no further information was provided. This specimen is housed in the Albany Museum, Makhanda (Grahamstown), Eastern Cape, South Africa, however due to its poor state of preservation it is not possible to ascertain whether it has been anthropogenically modified. Deacon ^2^ concluded that there was no evidence of a “direct” association between the wood and cultural materials.

Analysis of the macroscopic plant remains from Areas 1 and 2 recovered *in situ* and from bulk sediment samples was conducted by Wells ^23^, and is summarised in Table S6. There is no specific information provided by Wells ^23^ on the methods employed in this analysis, however Deacon ^2^ recounted that 18 wood samples were sectioned for comparative analysis with woody plants collected from the vicinity of the springs. Although some of these samples were noted to be potentially identifiable, none were actually identified during this study ^2^. It was noted however that this may indicate the specimens collected from Amanzi Springs were not represented in the present-day forest biomes found in this area. Wells ^23^ concluded that despite these limitations there was evidence the springs periodically supported communities of hygrophilous vegetation, indicating that the springs held pooled water at different times in the past. Spring conditions during the deposition of the Enqhura Member layers supported the growth of an aquatic community of herbaceous plants, followed by a period of very rapid sedimentation that covered and preserved the stems of these plants. Conditions were also likely more mesic than present day during the deposition of the Rietheuval Member, as evidenced by the presence of *Erythrina* species of trees. Finally, during the deposition of the Balmoral Member, conditions appear to have been drier, with woody species characteristic of the savanna or bushveld biomes growing in the vicinity of the springs ^23^.

In sum, macroscopic plant remains were recovered from each of the main stratigraphic aggregates in Areas 1 and 2, although preservation varied both between layers and laterally across the springs. This was most pronounced in the upper layers where recent reduction of the water-table had led to these layers drying out ^2^. With the exception of the more durable wood specimens and plant macrofossils, many of the organics were carbonised and could not be identified ^2,23^. Despite these limitations, there is great potential for further investigation of the palaeoenvironmental record at Amanzi Springs, and research into the palynology and paleoecology of the springs is ongoing.

Table S6. Summary of the macroscopic plant remains analysed by Wells ^23^ from Areas 1 and 2.

| **Layer** | **Specimen Type** | **Description** |
| --- | --- | --- |
| Enqhura Member | Fruits/Fruit Segments  (n=6) | Not identified to species nor considered morphologically indicative of any specific type of vegetation. Specimens 5-7mm in diameter, flattened on one side with an aril-like crown near the point of attachment. Fruits are similar in size and shape to *Grewia occidentaslis* L. |
|  | Dichotomously Branching Herbaceous Plants | Present in the White Sands and Brown Sands of Area 1 in large numbers. Stems ranging in thickness from ~5mm at their base to ~1-2mm at the branched top section, reaching heights up to 1.5m, likely representing an aquatic community. In the White Sands layer, the stems and roots were carbonised and very fragile, with preservation marginally better in the Brown Sands unit. Specimens preserved by deposition around the individual stems, indicating that sedimentation was rapid, within the span of a single growth season. |
| Rietheuvel Member | Woody Stems and Root Fragments | A wide range of woody stems, fragile twigs, and root fragments. Morphology resembles dicotyledonous trees and shrubs growing near the springs. Presence of these specimens in large numbers suggests vegetation had a strong woody element at the time of deposition. |
|  | Prickle Bases (n=3) | Prickle bases with raised rims, one containing its prickle, indistinguishable from prickles on arborescent *Erythrina* spp. (e.g., *E. caffra* Thunb. and *E. lysistemon* Hutch). Found in the Brown Humic Sands layer, specimens are 8-12mm in length and 5-7mm wide. *Erythrina* spp. do not occur in the local area, however trees of this species found in the dry coastal forest and in the bushveld around water sources. Presence suggests that climate at Amanzi at the time of deposition was similar/wetter than present day. |
| Balmoral Member | Leaf/Stem Epidermis  (n=13) | Sections of epidermis made up of octagonal cells with thickened lateral walls, ~2cm^2^ in area, found in Pothole Fill layer. No stomata present, nor features such as specialised tissues beneath the epidermis. |
|  | Leaflets (n=7) | Morphologically similar to *Schotia afra* (L) Bodin. ~5-9mm in length and ~3-4mm wide, found in the Pothole Fill layer and overlying Marginal Clays layer. Leaflets of this type are characteristic of bushveld and savanna woody species such as *Schotia* spp. and *Acacia* spp., indicating that vegetation in vicinity of springs at time of deposition included xerophytic woody species commonly found in bushveld, savanna, or dry forest. |
|  | Leaf portions (n=3) | Leaf portions of an unidentified large-leaved dicotyledonous species, 1cm^2^ in area, found in Pothole Fill. Usually found on woody trees and shrubs from hygrophilous communities, suggesting springs likely supported vegetation more hygrophilous than the wider area. |

4.2 Area 7 Pollen Analysis

14 samples were analysed for pollen and microcharcoal at Area 7. Of these, 7 samples from the LBCSS, GBSS, OBSS, LPGSS, and LGSS yielded negligible amounts of pollen and microcharcoal (maximum of 1 grain at *Lycopodium* counts >100). Processing of these clay-enriched layers using HF instead of SPT for mineral separation is currently underway to compare the effects of these methods in the collection of pollen from the samples. A remaining 3 samples from the DOSS (GH3) and 4 from the DBBPS (GH5) layers are presented here.

Of the 7 samples from DOSS and DBBPS, 18 pollen and spore taxa were identified to family and, where possible, genus. Overall, wetland taxa are well-represented within the DOSS and DBBPS layers at Area 7, especially Cyperaceae (26-48%; mean 43%), and mosses and ferns as represented by monolete (5-60%; mean 31%) and trilete spores (1-17%, mean 4%) (Figs. S21-S22). This is unsurprising given that micromorphological data indicates these two units formed during periods when the spring basin was filled with still water in a low-energy depositional environment during interglacials MIS 7 and 11. Monoletes (ferns) increase through the section with greater amounts during MIS 7 in the DOSS layers. Typhaceae is also seen to increase in DOSS, coincident with the appearance of *Gunnera,* a taxon that occurs in marshy conditions*.* *Gunnera* is a large-leafed herb, modern versions of which are edible and are used for medicinal purposes in southern Africa. It also known to have a symbiotic relationship with cyanobacteria that grow in still to slow moving water. Poaceae are seen in the uppermost layers of DBBPS and throughout the DOSS layers, which potentially represents Phragmite reed grasses that grow in wetland settings. *Podocarpus* is also seen in the DOSS layers. This conifer is found today throughout the coastal Afrotemperate forests and montane areas of Kwa-Zulu Natal and the Eastern Cape. Ebenaceae also occurs only in DOSS, likely representing a shift toward a more forested environment.

In contrast to the DOSS, the DBBPS is dominated (5-79%; mean 42%) by the Cape Rose, *Cliffortia,* most species of which are found within the Fynbos of the Cape Floral Region (CFR). While the Amanzi Springs area is not within the extent of the Fynbos Biome today, which mainly occurs closer to the coast to the west of Gqeberha (Port Elizabeth), it is possible that the high amount of *Cliffortia* in the DBBPS may correlate with higher sea levels during MIS 11. Bathymetric and sea-level modelling suggests that the MIS 11 sea level high-stand would have resulted in the formation of a palaeo-ria within 7 km of the site, although the minimum distance to the coastline likely did not change considerably ^8^. It has been suggested at Pinnacle Point during later time periods that as sea levels fluctuated the Fynbos moved with the coast ^99^ and so it may be that the same is occurring at Amanzi Springs during MIS 11. Ericaceae (heath) is another potential indicator of fynbos vegetation, but it is only found within the DOSS deposits in MIS 7 (<1% relative abundance), where *Cliffortia* still occurs but in much lower relative abundance. Aizoaceae (stone plants) are also only found in DOSS. While often an indicator of arid to semi-arid conditions in the Succulent Karoo there is also a high diversity of this within the CFR. *Passerina* is found in the lowest layers of DBBPS, which generally occur as low trees or shrubs in South Africa today with the greatest diversity within regions in the winter rainfall zone, perhaps also linked to the expansion of the CFR to the inland and eastwards of the coast.

There is also a clear distinction between the overall pollen and microcharcoal concentrations of the DBBPS and the DOSS. The DBBPS has high pollen concentrations (maximum 172 x 10^4^ grains g^--1^; minimum 74 x 10^4^ grains g^--1^) concurring with high microcharcoal content (Fig. S23). In contrast, the DOSS has comparatively lower pollen concentrations (maximum 63 x 10^4^ grains g^--1^; minimum 26 x 10^4^ grains g^--1^) with significant amounts of monolete spores, coinciding with low microcharcoal counts.

Thus, while both the DOSS and DBBPS generally represent an overall wetter environment, the development of vegetation during the formation of DBBPS and DOSS at Area 7 appears to reflect fluctuating moisture availability at the site, coinciding with sea level change. The DBBPS, in addition to its high relative abundance of *Cliffortia* and microcharcoal, contains very few, if any, forest taxa (<1%). Combined, this evidence suggests that the Area 7 spring experienced generally cool and arid conditions during the earlier phase of its formation at MIS 11. The absence of forest taxa within the DBBPS layer may also indicate that the landscape was relatively open during its formation. In contrast, the DOSS layers show a shift towards less open landscapes with more humid, and potentially warmer conditions at the site during MIS 7/6, as denoted by the increase in monolete spores and marked decrease of *Cliffortia* grains, substantial decrease in the amount of microcharcoal, and the presence of woodland taxa such as *Podocarpus* and *Ebenaceae*.


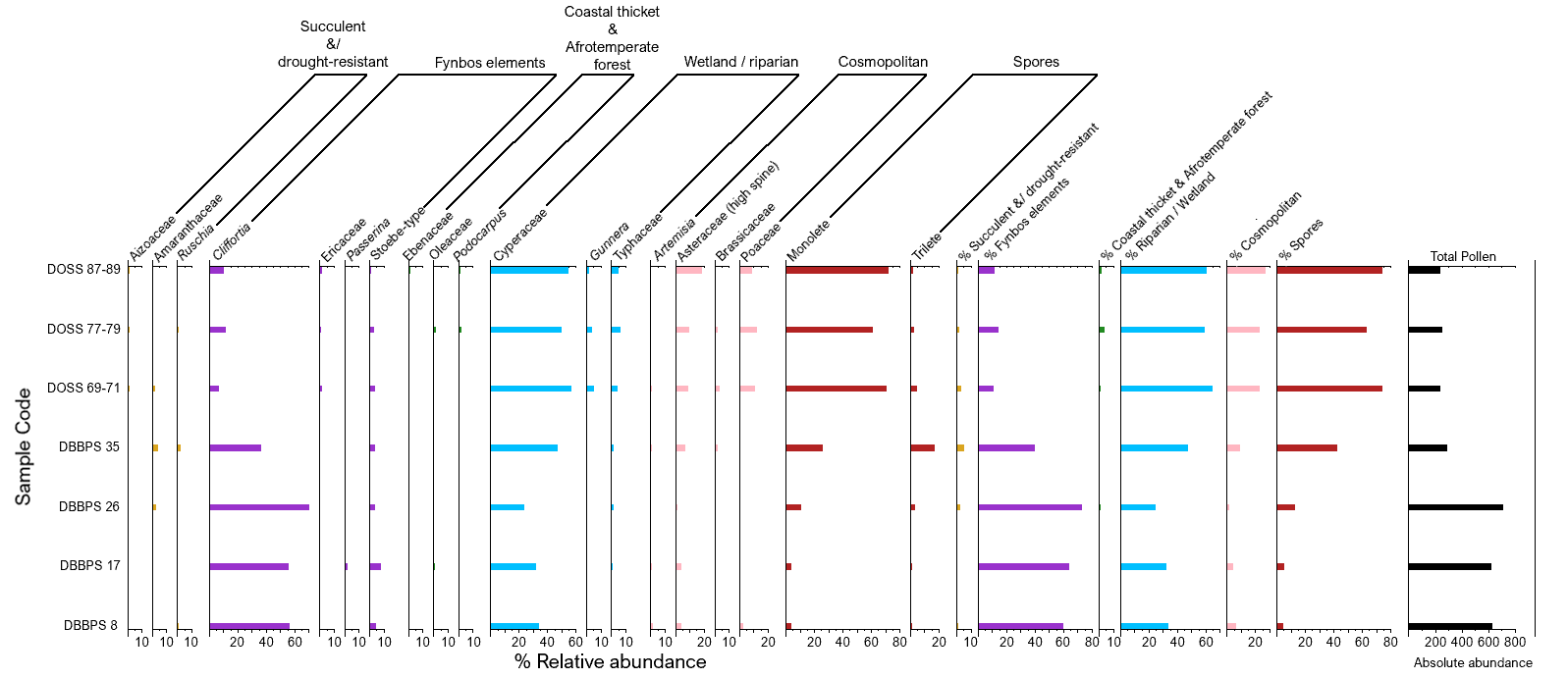


Fig. S21. Relative percentage pollen and non-pollen palynomorphs diagram **for the DOSS and DBBPS sedimentary units in Area 7.** Taxa are grouped according to general ecological affinities and are plotted according to sample code, based on relative height within sedimentary units. Spores (monoletes and triletes) were counted, but not included in the total pollen sum/absolute abundance.


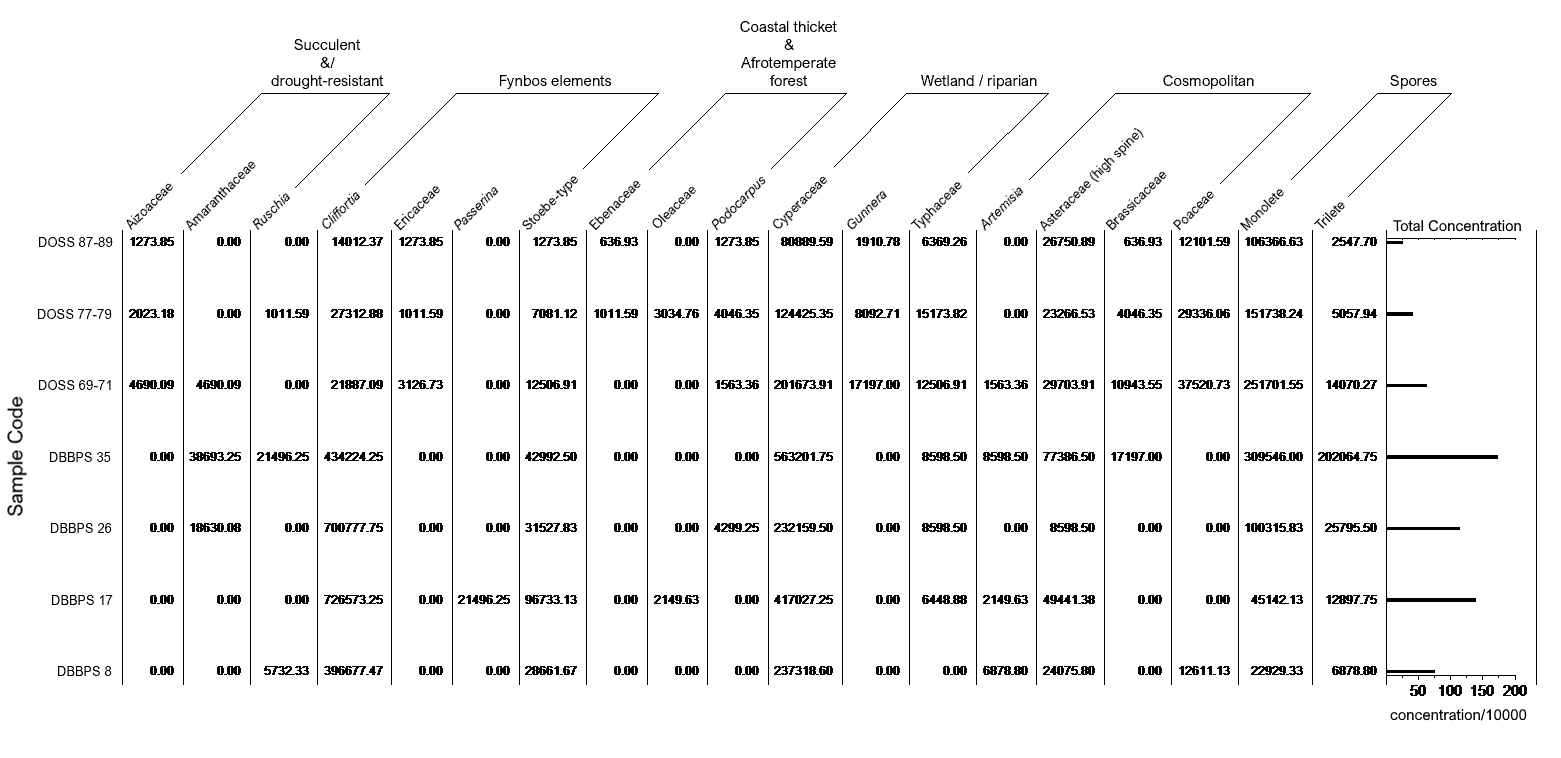


Fig. S22. Pollen and non-pollen palynomorph grains **presented as concentration (grains per gram sample) in the DOSS and DBBPS.** Concentration was calculated using *Lycopodium* spore counts ^100^.


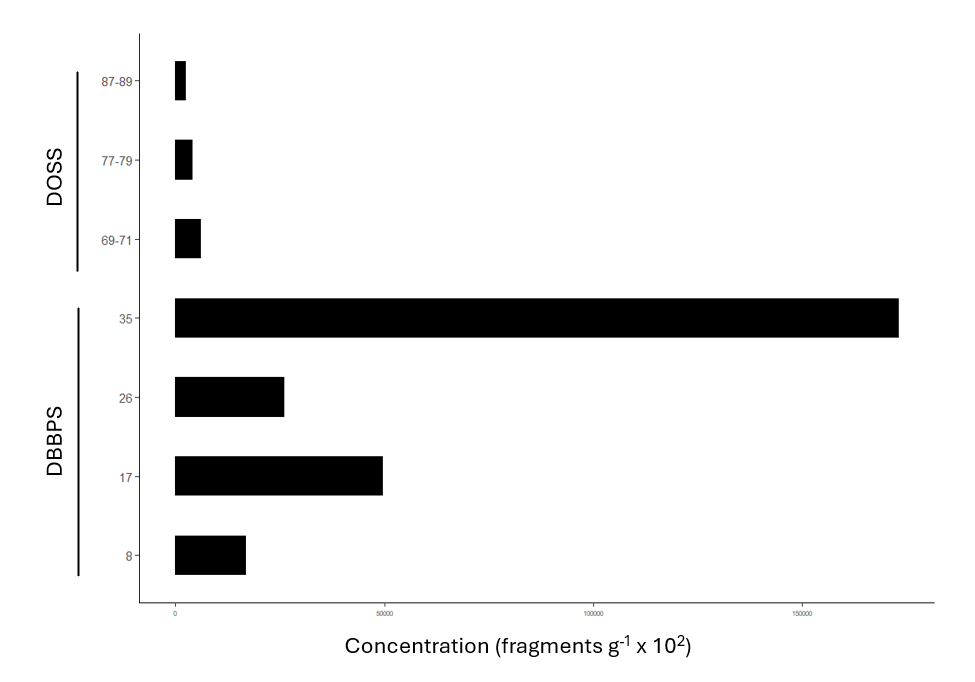


Fig. S23. Microscopic charcoal fragments **(fragments per gram) in the DOSS and DBBPS.** Concentration was calculated in the same manner as pollen and non-pollen palynomorphs.

5. Archaeological Data

5.1 Introduction

The following sections provide an overview of the lithic assemblages from Area 7, and includes a summary of the raw materials, typological descriptions of the lithic samples, flake platforms, retouch, cores, the evidence for on-site reduction, EL/M ratios, artefact weathering, and artefact size profiles.

In total, 3523 lithics were recovered during the 2017-2019 field seasons. This includes a small sample collected from the surface (*n*=21), as well as the overlying modern fill layers and disturbed contexts (*n*=19), and 3483 artefacts plotted using total stations from secure stratigraphic contexts. The artefacts from the surface, modern fill layers, and disturbed contexts have been excluded from this study. Of this sample, 3018 of were recovered from the upper deposits (GH1-3) and are assigned to the MSA, and 465 were found in the lower deposits (GH4-5) and are assigned to the Acheulian. A summary of excavation volumes and artefact density is provided in Table S7, and the lithic samples from each stratigraphic layer are presented in Table S8.

Table S7. Summary of excavations at Area 7 showing volume of excavated sediment, density of recovered lithics, charcoal, and organic material.

| **Spring Phase** | **Geological Horizon** | **Layer** | **Excavated** | **Lithics** | | **Charcoal** | | **Wood/organics** | |
| --- | --- | --- | --- | --- | --- | --- | --- | --- | --- |
|  |  |  | **Volume (m^3^)** | ***n*** | **Density (n/m^3^)** | ***n*** | **Density (n/m^3^)** | ***n*** | **Density (n/m^3^)** |
| Active | GH1 | LGSS | 2.02 | 179 | 89 | 2 | 0.99 | 0 | - |
| Active | GH2 | LPGSS | 3.02 | 967 | 320 | 3 | 0.99 | 1 | 0.33 |
| Marsh | GH3 | DOSS | 5.88 | 1180 | 201 | 65 | 11.05 | 11 | 1.87 |
|  |  | OBSS | 3.17 | 692 | 218 | 3 | 0.94 | 0 | - |
| Active | GH4 | LBCSS | 4.24 | 401 | 94 | 2 | 0.47 | 4 | 0.94 |
|  |  | GBSS | 0.33 | 29 | 88 | 0 | - | 0 | - |
| Marsh | GH5 | DBBPS | 0.67 | 35 | 52 | 0 | - | 88 | 131.34 |

Table S8. Summary of Area 7 artefacts arranged by layer.

| **Artefact Type** | | **GH1** | | **GH2** | | **GH3** | | | | **GH4** | | | | **GH5** | | **Total** | |
| --- | --- | --- | --- | --- | --- | --- | --- | --- | --- | --- | --- | --- | --- | --- | --- | --- | --- |
|  |  | ***LGSS*** | | ***LPGSS*** | | ***DOSS*** | | ***OBSS*** | | ***GBSS*** | | ***LBCSS*** | | ***DBBPS*** | |  |  |
|  |  | ***n*** | **%** | ***n*** | **%** | ***n*** | **%** | ***n*** | **%** | ***n*** | **%** | ***n*** | **%** | ***n*** | **%** |  | **%** |
| **Flakes** | *Complete* | 75 | 41.9% | 398 | 41.2% | 425 | 36.0% | 265 | 38.3% | 8 | 27.6% | 118 | 29.3% | 12 | 34.3% | *1301* | 37.3% |
|  | *Split* | 13 | 7.3% | 98 | 10.1% | 80 | 6.8% | 53 | 7.7% | - | - | 24 | 6.0% | 3 | 8.6% | *271* | 7.8% |
|  | *Proximal* | 11 | 6.1% | 33 | 3.4% | 37 | 3.1% | 17 | 2.5% | - | - | 6 | 1.5% | - | - | *104* | 3% |
|  | *Medial* | 8 | 4.5% | 25 | 2.6% | 29 | 2.5% | 16 | 2.3% | - | - | 7 | 1.7% | - | - | *85* | 2.4% |
|  | *Distal* | 6 | 3.4% | 30 | 3.1% | 28 | 2.4% | 18 | 2.6% | - | - | 2 | 0.5% | - | - | *84* | 2.4% |
|  | *Fragment* | 23 | 12.8% | 118 | 12.2% | 210 | 17.8% | 86 | 12.4% | 3 | 10.3% | 19 | 4.7% | 3 | 8.6% | *462* | 13.3% |
|  | ***Sub-Total*** | ***136*** | ***76%*** | ***702*** | ***72.6%*** | ***809*** | ***68.6%*** | ***455*** | ***65.8%*** | ***11*** | ***37.9%*** | ***176*** | ***43.7%*** | ***18*** | ***51.4%*** | ***2307*** | ***66.2%*** |
| **Blades** | *Complete* | 7 | 3.9% | 10 | 1.0% | 6 | 0.5% | 3 | 0.4% | - | - | 1 | 0.2% | - | - | *27* | 0.8% |
|  | *Split* | - | - | - | - | 1 | 0.1% | - | - | - | - | - | - | - | - | *1* | 0% |
|  | *Proximal* | 1 | 0.6% | - | - | 2 | 0.2% | - | - | - | - | - | - | - | - | *3* | 0.1% |
|  | *Medial* | 1 | 0.6% | 1 | 0.1% | 2 | 0.2% | - | - | - | - | - | - | - | - | *4* | 0.1% |
|  | *Distal* | 1 | 0.6% | - | - | - | - | - | - | - | - | - | - | - | - | *1* | 0% |
|  | ***Sub-Total*** | ***10*** | ***5.6%*** | ***11*** | ***1.1%*** | ***11*** | ***0.9%*** | ***3*** | ***0.4%*** | ***0*** | ***0.0%*** | ***1*** | ***0.2%*** | ***0*** | ***0.0%*** | ***36*** | ***1%*** |
| **Retouch** | *Complete* | 3 | 1.7% | 9 | 0.9% | 6 | 0.5% | 13 | 1.9% | - | - | 8 | 1.7% | 1 | 2.9% | *39* | 1.1% |
|  | *Split* | - | - | 1 | 0.1% | 3 | 0.3% | 3 | 0.4% | 1 | 3.4% | 2 | 0.5% | - | - | *10* | 0.3% |
|  | *Proximal* | 4 | 2.2% | 10 | 1.0% | 13 | 1.1% | 10 | 1.4% | - | - | 5 | 1.2% | 3 | 8.6% | *45* | 1.3% |
|  | *Medial* | 1 | 0.6% | 1 | 0.1% | 2 | 0.2% | 1 | 0.1% | - | - | - | - | - | - | *5* | 0.1% |
|  | *Distal* | - | - | 3 | 0.3% | 3 | 0.3% | - | - | - | - | - | - | - | - | *6* | 0.2% |
|  | *Fragment* | 1 | 0.6% | 3 | 0.3% | 7 | 0.6% | 2 | 0.3% | 1 | 3.4% | 5 | 1.5% | 1 | 2.9% | *21* | 0.6% |
|  | ***Sub-Total*** | ***9*** | ***5.0%*** | ***27*** | ***2.8%*** | ***34*** | ***2.9%*** | ***29*** | ***4.2%*** | ***2*** | ***6.9%*** | ***20*** | ***5.0%*** | ***5*** | ***14.3%*** | ***126*** | ***3.6%*** |
| **Cores** | *Complete* | 7 | 3.9% | 27 | 2.8% | 36 | 3.1% | 49 | 7.1% | 6 | 20.7% | 88 | 21.8% | 3 | 8.6% | *216* | 6.2% |
|  | *Fragment* | 1 | 0.6% | 2 | 0.2% | 8 | 0.7% | 9 | 1.3% | - | - | 12 | 3.0% | 1 | 2.9% | *33* | 0.9% |
|  | ***Sub-Total*** | ***8*** | ***4.5%*** | ***29*** | ***3.0%*** | ***44*** | ***3.7%*** | ***58*** | ***8.4%*** | ***6*** | ***20.7%*** | ***100*** | ***24.8%*** | ***4*** | ***11.4%*** | ***249*** | ***7.1%*** |
| **LCTs** | *Complete* | - | - | - | - | 3 | 0.3% | 4 | 0.6% | 2 | 6.9% | 21 | 5.2% | 1 | 2.9% | *31* | 0.9% |
|  | *Rough-out* | - | - | - | - | - | - | - | - | 1 | 3.4% | 10 | 2.5% | - | - | *11* | 0.3% |
|  | *Fragment* | - | - | - | - | 1 | 0.1% | 1 | 0.1% | 2 | 6.9% | 7 | 1.7% | 1 | 2.9% | *12* | 0.3% |
|  | ***Sub-Total*** | ***0*** | ***0.0%*** | ***0*** | ***0.0%*** | ***4*** | ***0.3%*** | ***5*** | ***0.7%*** | ***5*** | ***17.2%*** | ***38*** | ***9.4%*** | ***2*** | ***5.7%*** | ***54*** | ***1.5%*** |
| **Other** | *Shatter* | 16 | 8.9% | 187 | 19.3% | 259 | 21.9% | 126 | 18.2% | 2 | 6.9% | 28 | 6.9% | 4 | 11.4% | *622* | 17.8% |
|  | *Hammerstone* | - | - | 6 | 0.6% | 7 | 0.5% | 7 | 1.0% | 3 | 10.3% | 12 | 3.0% | 1 | 2.9% | *35* | 1% |
|  | *Cobble* | - | - | 4 | 0.4% | 11 | 0.9% | 9 | 1.3% | - | - | 24 | 6.0% | 1 | 2.9% | *49* | 1.4% |
|  | *Cobble Frag* | - | - | 1 | 0.1% | 2 | 0.2% | - | - | - | - | 2 | 0.5% | - | - | *5* | 0.1% |
|  | ***Sub-Total*** | ***16*** | ***8.9%*** | ***198*** | ***20.5%*** | ***278*** | ***23.6%*** | ***142*** | ***20.5%*** | ***5*** | ***17.2%*** | ***66*** | ***16.9%*** | ***6*** | ***17.1%*** | ***713*** | ***20.5%*** |
| **Total** | | **179** | **5.1%** | **967** | **27.7%** | **1180** | **33.9%** | **692** | **19.9%** | **29** | **0.8%** | **401** | **11.6%** | **35** | **1%** | **3483** | **100%** |

5.2 Raw Materials

Raw materials were identified by visual inspection under 10x magnification using a hand lens, and under 20-40x magnification using a stereo microscope if required. Attributes recorded for each artefact consisted of lithology type, texture, grain-size, sorting, inclusions, and colour. Some of these properties relate to the homogeneity and fracture properties of each raw material, and are features that correlate with the visual and textural attributes that would have been available to the stone tool makers ^101^. No geochemical or petrological analysis was undertaken at this stage of the study.

Cortex was recorded as a percentage of the dorsal surface of flakes and retouched pieces, the entire surface of cores and LCTs, and separately for each individual core and LCT face. Cortex represents the remnant external surface of a raw material and provides an indication of the original geological context of the raw material as well as information on the shape and size of the original clasts that were selected for use. In addition to the characteristics of the raw materials themselves, several behavioural factors can influence the amount of cortex present on flakes in an assemblage. These include, but are not limited to, the distance that raw material clasts were transported, the stage in the reduction sequence that artefacts were discarded, and the technological decisions made during core reduction that influenced the manner in which flakes were removed from a core ^102–104^. When assessed on an assemblage level, the presence, location, and relative amount of cortex on dorsal surfaces provide information on core reduction strategies and the length of core reduction sequences ^102–106^. To estimate the amount of cortex on Area 7 artefacts, a 7-stage scale was used (0%, 1-20%, 21-40%, 41-60%, 61-80%, 81-99%, 100%). Although this approach is not quantitative, it is useful for reducing the likelihood of estimation errors and improving reproducibility ^104,107^. The presence or absence of cortex on flake platforms was recorded as a separate attribute.

Several different varieties of fine-grained sedimentary raw materials were observed and combined into the category ‘fine grained siliceous’ (FGS). This was due to low overall numbers and difficulty in distinguishing them based on visual characteristics alone, especially where chemical weathering had altered artefact surfaces. FGS lithologies included silicified mudstone/claystone, siliceous shale, and otherwise non-diagnostic examples of fine-grained silica-rich raw materials. In addition, several different varieties of chert, chalcedony, and cryptocrystalline siliceous lithologies were combined into the category ‘chert/CCS’, following the approach of Wilkins et al. ^107^, to minimise errors in distinguishing these raw materials in hand specimen ^108^.

In total, there are at least 11 different raw materials represented at Area 7, including isolated examples of hornfels and igneous lithologies. A summary of the Area 7 artefact types by raw material is presented in Table S9, and the frequency of lithics with dorsal cortex by layer is presented in Table S10. Quartzite is the dominant raw material, accounting for 86% of the entire stone tool assemblage and greater than 80% of all raw materials in each layer. Sandstone is also present in all layers except the GBSS (GH5), albeit in very low frequencies, and accounts for 3.4% of the entire assemblage. Both are ultimately derived from the TMG quartzitic sandstone formations and are the most abundant lithologies found in primary and secondary sources within a 20 km radius of Amanzi Springs. Due to the variable nature of low-grade metamorphism in the TMG, both sandstones and quartzites can be found in the same primary and secondary deposits and often co-occur within the same outcrops. They can possess broadly similar mechanical properties, although quartzites that consist of higher proportions of cemented secondary silica generally have better fracture propagation characteristics and a finer texture. At secondary sources where they occur as cobbles, they can be indistinguishable from each other until a test flake has been removed. Quartzite is differentiated here by the presence of interlocking quartz grains, while sandstone is characterised by the presence of clear grain-boundaries between quartz grains.

There is a substantial increase in raw material diversity (χ*^2^* = 45.5, *df* = 3, *p*= <.0001) in the upper deposit, with elevated proportions of silcrete and other fine-grained siliceous lithologies in the MSA layers (Fig. 3). Silcrete forms the next most abundant raw material after quartzite, constituting 5.4% of the entire assemblage, followed by quartz (2.7%), FGS (1.3%), chert/CCS (0.7%), igneous lithologies (0.3%), and hornfels (0.1%). In the lower deposits, quartzite makes up more than 95% of all raw materials, with low numbers of sandstone (n=8), quartz (n=6), and silcrete (n=2) artefacts, and a single flake made on chert in GH4 (LBCSS). Although quartzite remains the dominant raw material in the upper deposit, the majority of the silcrete, quartz, and chert/CCS artefacts are found in these layers, and FGS, hornfels, and igneous lithologies appear for the first time at the site.

Table S9. Summary of Area 7 artefact types arranged by raw material type, including artefacts recovered from the surface and disturbed contexts.

| **Artefact Type** | **Raw Material** | | | | | | | | **Total** | |
| --- | --- | --- | --- | --- | --- | --- | --- | --- | --- | --- |
|  | **Quartzite** | **Silcrete** | **Sandstone** | **Quartz** | **FGS** | **CCS** | **Igneous** | **Hornfels** |  | **%** |
| **Flakes** |  |  |  |  |  |  |  |  |  |  |
| *Complete* | 1150 | 85 | 32 | 17 | 12 | 11 | 4 | 1 | *1312* | 37% |
| *Split* | 251 | 11 | 7 | - | 2 | 2 | 1 | 1 | *275* | 7.8% |
| *Proximal* | 88 | 11 | 2 | 1 | 3 | 1 | - | - | *106* | 3% |
| *Medial* | 74 | 7 | 3 | 1 | - | - | - | - | *85* | 2.4% |
| *Distal* | 64 | 13 | 1 | 1 | 1 | 4 | - | - | *84* | 2.4% |
| *Fragment* | 394 | 17 | 20 | 10 | 11 | 5 | 3 | 2 | *462* | 13% |
| ***Sub-Total*** | ***2021*** | ***144*** | ***65*** | ***30*** | ***29*** | ***23*** | ***8*** | ***4*** | ***2324*** | ***66*%** |
| **Blades** |  |  |  |  |  |  |  |  |  |  |
| *Complete* | 20 | 4 | 1 | - | 2 | - | - | - | *27* | 0.8% |
| *Split* | - | 1 | - | - | - | - | - | - | *1* | 0% |
| *Proximal* | 2 | 2 | - | - | - | - | - | - | *4* | 0.1% |
| *Medial* | 1 | 3 | - | - | - | - | - | - | *4* | 0.1% |
| *Distal* | - | 1 | - | - | - | - | - | - | *1* | 0% |
| ***Sub-Total*** | ***23*** | ***11*** | ***1*** | ***0*** | ***2*** | ***0*** | ***0*** | ***0*** | ***37*** | ***1.1%*** |
| **Retouched Tools** |  |  |  |  |  |  |  |  |  |  |
| *Complete* | 31 | 2 | 3 | - | 1 | 2 | - | - | *39* | 1.1% |
| *Split* | 10 | - | - | - | 1 | - | - | - | *11* | 0.3% |
| *Proximal* | 39 | 4 | 3 | - | - | - | - | - | *46* | 1.3% |
| *Medial* | 4 | 1 | - | - | - | - | - | - | *5* | 0.1% |
| *Distal* | 5 | 1 | - | - | - | - | - | - | *6* | 0.2% |
| *Fragment* | 20 | - | - | 1 | - | - | - | - | *21* | 0.6% |
| ***Sub-Total*** | ***109*** | ***8*** | ***6*** | ***1*** | ***2*** | ***2*** | ***0*** | ***0*** | ***128*** | ***3.6%*** |
| **Cores** |  |  |  |  |  |  |  |  |  |  |
| *Complete* | 205 | 8 | 7 | - | 1 | 1 | 1 | - | *223* | 6.3% |
| *Fragment* | 29 | 1 | - | 2 | - | 1 | - | - | *33* | 0.9% |
| ***Sub-Total*** | ***234*** | ***9*** | ***7*** | ***2*** | ***1*** | ***2*** | ***1*** | ***0*** | ***256*** | ***7.3%*** |
| **LCTs** |  |  |  |  |  |  |  |  |  |  |
| *Complete* | 38 | - | - | - | - | - | - | - | *38* | 1.1% |
| *Rough-out* | 11 | - | - | - | - | - | - | - | *11* | 0.3% |
| *Fragment* | 13 | - | - | - | - | - | - | - | *13* | 0.4% |
| ***Sub-Total*** | ***62*** | ***0*** | ***0*** | ***0*** | ***0*** | ***0*** | ***0*** | ***0*** | ***62*** | ***1.8%*** |
| **Other** |  |  |  |  |  |  |  |  |  |  |
| *Shatter* | 503 | 19 | 28 | 61 | 11 | 2 | 1 | 1 | *626* | 18% |
| *Hammer-stone* | 30 | - | 4 | 1 | - | - | - | - | *35* | 1% |
| *Unmodified-Cobble* | 40 | 1 | 7 | - | - | - | - | - | *48* | 1.4% |
| *Cobble- Fragment* | 5 | - | - | - | - | - | - | - | *5* | 0.1% |
| ***Sub-Total*** | ***578*** | ***20*** | ***39*** | ***62*** | ***11*** | ***2*** | ***1*** | ***1*** | ***714*** | ***20%*** |
| **Total** | **3027** | **192** | **118** | **95** | **45** | **29** | **10** | **5** | ***3522*** | ***100%*** |
| ***% Total*** | ***86%*** | ***5.4%*** | ***3.3%*** | ***2.7%*** | ***1.3%*** | ***0.8%*** | ***0.3%*** | ***0.1%*** | ***100%*** | |

Examples of both outcrop and cobble cortex types are present in all layers, indicating that raw materials were procured from both primary and secondary sources, although there is strong evidence for an increase in the proportion of outcrop to cobble cortex in the upper deposit (χ*^2^* = 27.8, *df* = 3, *p*= <.0001) (Fig. 3b). Despite this shift in the proportion of cortex types between layers, cobble cortex is always the most abundant type, indicating that cobbles were the main source of raw materials at the site. The high frequency of cores made on quartzite (91.4%) indicate it was always central to reduction sequences at Area 7. The clear preference for quartzite is not surprising given the abundance of this raw material at both primary and secondary sources on the surrounding landscape. However, despite the emphasis on quartzite there is also a clear shift towards the use of finer-grained lithologies in the younger GH1-3 layers.

Table S10. Cortex amounts for all complete flakes, blades, and retouched pieces at Area 7.

|  | **Dorsal Cortex Cover** | | | | | | | | | | | | | |  | **Total %**  **with**  **Cortex** |
| --- | --- | --- | --- | --- | --- | --- | --- | --- | --- | --- | --- | --- | --- | --- | --- | --- |
| **Layer** | **0%** | | **1-20%** | | **21-40%** | | **41-60%** | | **61-80%** | | **81-99%** | | **100%** | | **Total** |  |
|  | ***n*** | **%** | ***n*** | **%** | ***n*** | **%** | ***n*** | **%** | ***n*** | **%** | ***n*** | **%** | ***n*** | **%** |  |  |
| LGSS | 57 | 69% | 5 | 6% | 5 | 6% | 4 | 5% | 5 | 6% | 4 | 5% | 3 | 4% | **83** | 31% |
| LPGSS | 321 | 77% | 19 | 5% | 8 | 2% | 13 | 3% | 5 | 1% | 36 | 9% | 14 | 17% | **416** | 23% |
| DOSS | 340 | 78% | 21 | 5% | 16 | 4% | 14 | 3% | 11 | 3% | 24 | 6% | 10 | 12% | **436** | 22% |
| OBSS | 187 | 67% | 19 | 7% | 17 | 6% | 9 | 3% | 13 | 5% | 22 | 8% | 13 | 16% | **280** | 33% |
| GBSS | 3 | 38% | 1 | 13% | 1 | 13% | - | - | 1 | 13% | 1 | 13% | 1 | 1% | **8** | 63% |
| LBCSS | 82 | 66% | 8 | 6% | 3 | 2% | 8 | 6% | 6 | 5% | 13 | 10% | 5 | 6% | **125** | 34% |
| DBBPS | 11 | 85% | 2 | 15% | - | - | - | - | - | - | - | - | - | - | **13** | 15% |
| **Total** | **1001** | **74%** | **75** | **6%** | **50** | **4%** | **48** | **4%** | **41** | **3%** | **100** | **7%** | **46** | **3%** | **1361** |  |

5.3 The Area 7 Lithic Assemblages

Fig. S24 shows the Area 7 assemblages organised by type and layer. Table S11 summarises the LCTs collected during excavation. Figs. S25-27 show the spatial distribution of artefacts piece-plotted during excavation.

**GH5**

The DBBPS assemblage is small (n=35), and the available data indicates that lithic density (52/m^3^) in this part of the spring is the lowest at the site. The sample includes complete flakes, retouched pieces, core maintenance flakes, and fragments. Four cores were also found, along with nondiagnostic pieces of shatter, a hammerstone, and an unmodified cobble. The DBBPS assemblage includes one complete LCT and an LCT fragment (Table S11). A high quantity of organic material, including carbonised plant remains, charcoal, and preserved fragments of wood were found in this layer, and several artefacts, including a handaxe and unmodified flakes, were found in close spatial association with the preserved wood. At present the nature of this association remains unclear and investigation of residues and microwear traces on both the artefacts and preserved wood is required.

**GH4**

The LBCSS layer contains a relatively dense artefact accumulation immediately below the interface with the overlying DOSS, with a lower density of artefacts (75/m^3^) found deeper in the layer. The material from the LBCSS/DOSS interface likely represents a palimpsest of artefacts that were accumulated by erosion and deflation of the upper portion of this layer before being buried as the DOSS was formed. The LBCSS lithic sample (n=401) contains the bulk of the artefacts excavated from the lower deposit. The unretouched debitage consists of complete flakes, fragments, and core maintenance flakes. Three flakes showed evidence of being detached via bipolar reduction, and one flake was classed as a blade based on a length to width ratio of ≥2:1. Retouched pieces make up 5% of the sample, with simple notched pieces, scrapers, and laterally retouched flakes. The LBCSS assemblage includes 100 cores, the highest number found at the site. Although no Levallois debitage or cores were found in the lower deposit, the LBCSS sample includes five bifacial hierarchical cores, the only prepared cores in the lower deposit. A high number of hammerstones, unmodified cobbles, and nondiagnostic shatter were also found in this layer. The collection of LCTs from the LBCSS is the largest from the site, with 21 complete LCTs, 10 preforms, and 7 fragments.

The GBSS is the lowermost artefact bearing unit at the spring margins exposed by excavations and underlies the OBSS. Although the GBSS artefact sample is small (n=29), owing to the limited depth of excavations in this part of the spring, the artefact density appears to be comparable to the LBCSS (78/m^3^). Of the 29 artefacts in the GBSS, three were identified immediately below the interface with the overlying OBSS. The GBSS assemblage includes complete flakes, fragments, and two retouched pieces. Six cores were found in this layer, as well as two possible hammerstones and two pieces of shatter. The GBSS sample includes two complete LCTs, a preform, and two LCT fragments.

**GH3**

The OBSS lithic sample (n=692) is smaller than the DOSS (n=1180), although artefact density is slightly higher (OBSS: 218/m^3^; DOSS: 201/m^3^). Unretouched debitage in the DOSS consists of complete flakes, fragments, unretouched Levallois flakes, core maintenance flakes, blades, and five bipolar flakes. Retouched pieces make up 2.9% of the sample, including notched pieces and a denticulate, scrapers, and laterally retouched flakes. The assemblage includes 44 cores, including 8 prepared cores, four of which are Levallois cores, along with a high frequency of shatter, six hammerstones, two cobble fragments, and ten unmodified cobbles. Although the DOSS assemblage is consistent with an MSA designation, of notable inclusion in this layer are three complete LCTs and an LCT fragment (Table S11).

The OBSS assemblage consists of complete flakes, fragments, unretouched Levallois flakes, core maintenance flakes, blades, and two bipolar flakes. This sample also contains 58 cores, including five bifacial hierarchical cores and four Levallois cores. A large sample of shatter, seven possible hammerstones, and nine unmodified cobbles were also found. As with the DOSS layer LCTs are present in OBSS, with four complete LCTs and one fragment (Table S11).

**GH2**

Artefact density in the LPGSS (n=967; 320/m^3^) is the highest of all layers at the site. Unretouched debitage includes complete flakes, fragments, unretouched Levallois flakes, core maintenance flakes, blades, and bipolar flakes. Retouched pieces make up 2.8% of the sample, comprising notched pieces and denticulates (including a serrated denticulate), scrapers, and an awl. The assemblage also contains a greater number of cores (n=26), include two Levallois cores and a bifacial hierarchical core, shatter, hammerstones, a cobble fragment, and three unmodified cobbles.

**GH1**

The LGSS lithic sample is small (n=179), and artefact density is considerably lower than the underlying GH2-3 layers (89/m^3^). The unretouched debitage consists of complete flakes, fragments, unretouched Levallois flakes, core maintenance flakes, and one bipolar flake. Retouched pieces make up 5% of the artefacts from LPGSS, however except for a denticulate, two notched pieces, and an awl, most only exhibit non-invasive retouch along the lateral margins. The assemblage includes eight cores, including two Levallois cores and a bifacial hierarchical core, and no hammerstones or unmodified cobbles.

**Summary**

A summary of the typological categories of debitage (flakes, retouched pieces, and fragments) is provided in Table S12. Examples of the Area 7 lithics are provided in Figures S28-S31.

Most of the complete flakes fall into three categories, in order of abundance: unretouched non-cortical flakes, initial cortical flakes (61-100% dorsal cortex), and residual cortical flakes (1-60% dorsal cortex). The unretouched non-cortical flakes exhibit no cortex on their dorsal surfaces and lack any of the technological attributes that define other categories of flakes. Initial and residual cortical flakes represent ‘core initiation flakes’ ^109^ that were struck from cobbles during the early phases of core reduction. The proportion of initial cortical flakes is higher than residual cortical flakes in all layers.

Retouched and unretouched Levallois flakes (also referred to as ‘bifacial hierarchical core exploitation flakes’ ^109^) first appear in the GH3 layers (DOSS-OBSS), increasing in relative abundance through GH2-GH1. The relative abundance of blades also increases in the upper deposit, some of which were produced using the Levallois method, although the overall frequency of blades remains low throughout the sequence. Crested blades, a type of core maintenance flake, form 19% of all complete blades, and are found in the OBSS, LPGSS, and LGSS layers. They are often, but not exclusively, associated with blade production from single platform cores, and have been grouped into a separate category due to their high elongation ratios. Core maintenance flakes (‘core-repair flakes’ ^109^), which include debordants (‘lateral repair flakes’ ^109^), ridge straightening flakes, and core trimming flakes (‘other concavity/convexity removal flakes’ ^109^), occur in all layers except GBSS. A small number of retouched core maintenance flakes (n=5) attest to their occasional use as tools. Finally, bipolar flakes occur in low numbers in all layers except the GBSS, indicating that although rare, the bipolar core-on-anvil technique was occasionally deployed to detach flakes at Area 7.


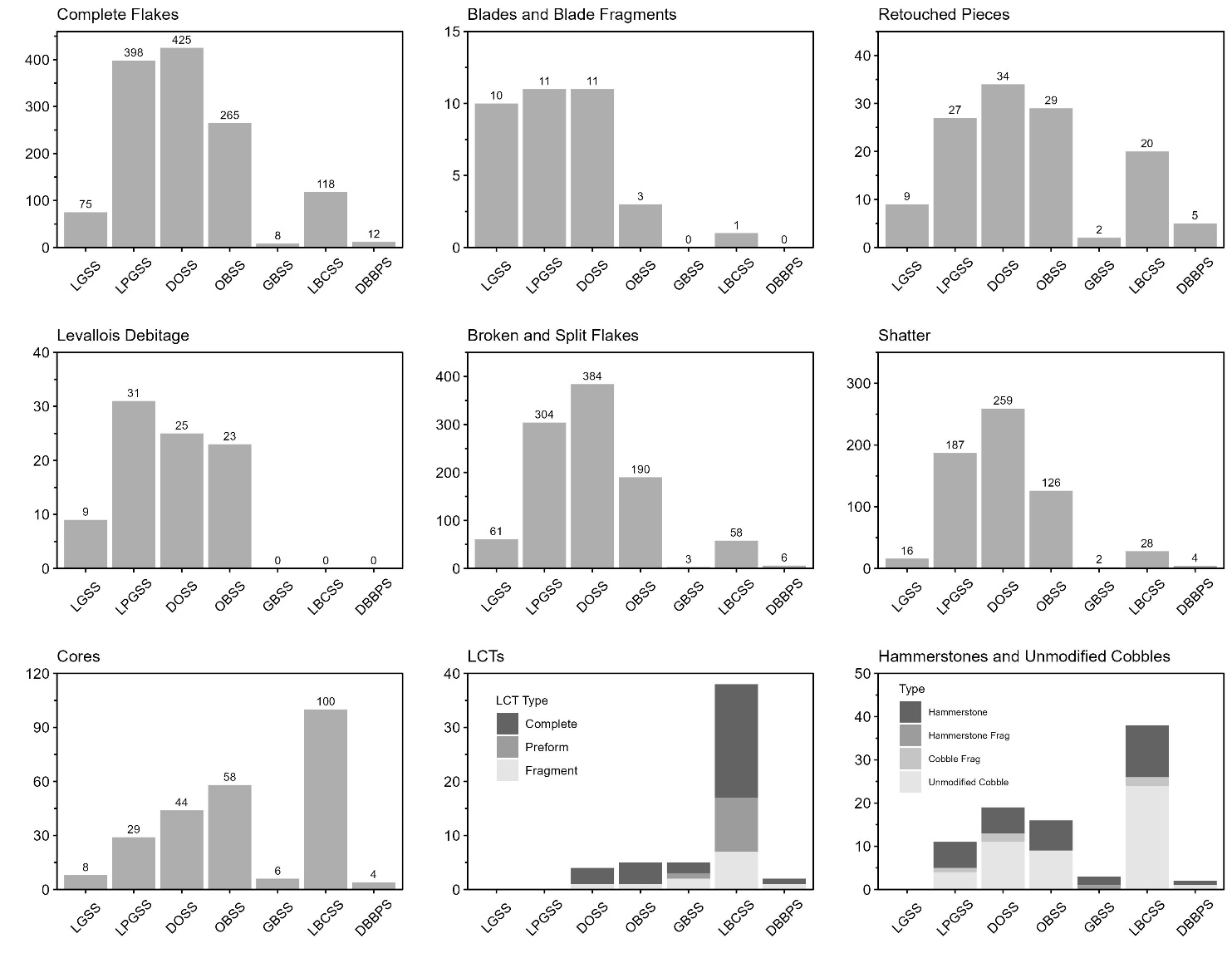


Fig. S24. Summary of the Area 7 assemblages organised by lithic type and layer.

Table S11. Summary of LCT classes and types.

| **LCTs** | **GH3** | | | | **GH4** | | | | **GH5** | | **Total** |
| --- | --- | --- | --- | --- | --- | --- | --- | --- | --- | --- | --- |
|  | **DOSS** | | **OBSS** | | **GBSS** | | **LBCSS** | | **DBBPS** | |  |
|  | ***n*** | **%** | ***n*** | **%** | ***n*** | **%** | ***n*** | **%** | ***n*** | **%** |  |
| **Artefact Class** |  |  |  |  |  |  |  |  |  |  |  |
| Complete LCT | 3 | *75%* | 4 | *80%* | 2 | *40%* | 21 | *55%* | 1 | *50%* | *38* |
| Preform | - | *-* | - | *-* | 1 | *20%* | 10 | *26%* | - | *-* | *11* |
| Fragment | 1 | *25%* | 1 | *20%* | 2 | *40%* | 7 | *18%* | 1 | *50%* | *13* |
| **Total** | **4** |  | **5** |  | **5** |  | **38** |  | **2** |  | **62** |
| **LCT Type** |  |  |  |  |  |  |  |  |  |  |  |
| Handaxe | 4 | *100%* | 4 | *80%* | 5 | *100%* | 34 | *89%* | 1 | *50%* | *54* |
| Cleaver |  |  | 1 | *20%* |  |  | 4 | *11%* | 1 | *50%* | *8* |
| **Total** | **4** |  | **5** |  | **5** |  | **38** |  | **2** |  | **62** |
| **Preforms** |  |  |  |  |  |  |  |  |  |  |  |
| Base & Tip | - |  | - |  | - |  | 7 | 41% | - |  | *7* |
| Tip | - |  | - |  | 1 | 33% | 3 | 18% | - |  | *4* |
| **Fragments** |  |  |  |  |  |  |  |  |  |  |  |
| Base | 1 | 100% | 1 | 100% | 2 | 67% | 4 | 24% | 1 | 100% | *10* |
| Tip | - |  | - |  | - |  | 3 | 18% | - |  | *3* |

Table S12. Area 7 debitage organised by typological category.

| **Flake Category** | **GH1** | | **GH2** | | **GH3** | | | | **GH4** | | | | **GH5** | | **Total by Type** | **%**  **Total** |
| --- | --- | --- | --- | --- | --- | --- | --- | --- | --- | --- | --- | --- | --- | --- | --- | --- |
|  | **LGSS** | | **LPGSS** | | **DOSS** | | **OBSS** | | **GBSS** | | **LBCSS** | | **DBBPS** | |  |  |
|  | ***n*** | **%** | ***n*** | **%** | ***n*** | **%** | ***n*** | **%** | ***n*** | **%** | ***n*** | **%** | ***n*** | **%** |  |  |
| Initial Cortical Flake | 12 | 7.7% | 55 | 7.4% | 44 | 5.2% | 44 | 9.0% | 3 | 23.1% | 21 | 10.7% | - | - | 179 | 7.2% |
| Residual Cortical Flake | 11 | 7.1% | 32 | 4.3% | 43 | 5.0% | 37 | 7.6% | 2 | 15.4% | 14 | 7.1% | 1 | 4.3% | 140 | 5.7% |
| Non Cortical Flake | 48 | *31%* | 293 | *39.6%* | 313 | *36.7%* | 165 | *33.9%* | 3 | *23.1%* | 67 | *34%* | 8 | *34.8%* | *897* | *36.3%* |
| Blade | 4 | *2.6%* | 7 | *0.9%* | 6 | *0.7%* | 1 | *0.2%* | - | *-* | 1 | *0.5%* | - | *-* | *19* | *0.8%* |
| Crested Blade | 1 | *0.6%* | 2 | *0.3%* | - | *-* | 2 | *0.4%* | - | *-* | - | *-* | - | *-* | *5* | *0.2%* |
| Levallois Blade | 2 | *1.3%* | 1 | *0.1%* | - | *-* | - | *-* | - | *-* | - | *-* | - | *-* | *3* | *0.1%* |
| Levallois Flake | 8 | *5.2%* | 24 | *3.2%* | 18 | *2.1%* | 17 | *3.5%* | - | *-* | - | *-* | - | *-* | *67* | *2.7%* |
| Retouched Levallois | 1 | *0.6%* | 6 | *0.8%* | 7 | *0.8%* | 6 | *1.2%* | - | *-* | - | *-* | - | *-* | *20* | *0.8%* |
| Retouched Flake | 7 | *4.5%* | 21 | *2.8%* | 24 | *2.9%* | 22 | *4.5%* | 2 | *15.4%* | 20 | *9.6%* | 5 | *21.7%* | *101* | *4.1%* |
| Bipolar Flake | 1 | *0.6%* | 3 | *0.4%* | 5 | *0.6%* | 2 | *0.4%* | - | *-* | 3 | *1.5%* | - | *-* | *14* | *0.6%* |
| Core Maintenance (CM) Flake | 2 | *1.3%* | 13 | *1.8%* | 18 | *2.1%* | 14 | *2.9%* | - | *-* | 14 | *7.1%* | 3 | *13%* | *64* | *2.6%* |
| Retouched CM Flake | 1 | *0.6%* | - | *-* | 2 | *0.2%* | 1 | *0.2%* | - | *-* | 1 | *0.5%* | - | *-* | *5* | *0.2%* |
| Split Flake | 10 | *6.5%* | 87 | *11.8%* | 71 | *8.3%* | 47 | *9.7%* | - | *-* | 23 | *11.7%* | 3 | *13%* | *241* | *9.8%* |
| Proximal Flake | 9 | *5.8%* | 27 | *3.6%* | 33 | *3.9%* | 11 | *2.3%* | - | *-* | 7 | *3.6%* | - | *-* | *87* | *3.5%* |
| Medial Flake | 8 | *5.2%* | 25 | *3.4%* | 29 | *3.4%* | 15 | *3.1%* | - | *-* | 7 | *3.6%* | - | *-* | *84* | *3.4%* |
| Distal Flake | 7 | *4.5%* | 26 | *3.5%* | 29 | *3.4%* | 17 | *3.5%* | - | *-* | 2 | *1%* | - | *-* | *81* | *3.3%* |
| Flake Fragment | 23 | *14.8%* | 118 | *15.9%* | 211 | *24.7%* | 86 | *17.7%* | 3 | *23.1%* | 18 | *9.1%* | 3 | *13%* | *463* | *18.8%* |
| **Total** | **155** |  | **740** |  | **853** |  | **487** |  | **13** |  | **198** |  | **23** |  | **2469** | *100%* |
| **% Total** | ***6.3%*** |  | ***30.0%*** |  | ***34.6%*** |  | ***19.7%*** |  | ***0.5%*** |  | ***8.0%*** |  | ***0.9%*** |  | ***100%*** |  |

**
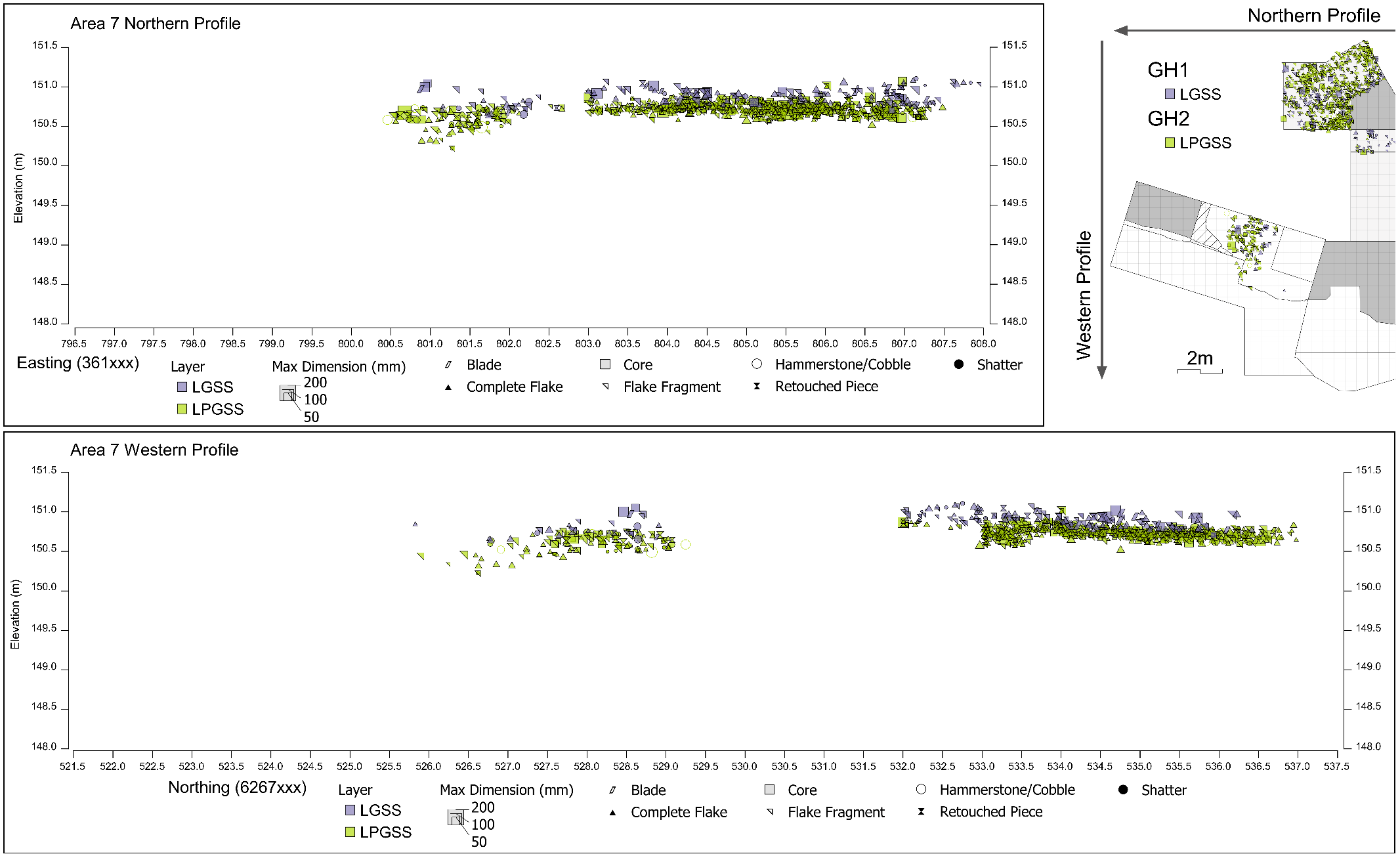
**

Fig. S25. Area 7 northern and western profile views**:** Distribution of artefact types in geological horizon 1 (LGSS) and 2 (LPGSS).

**
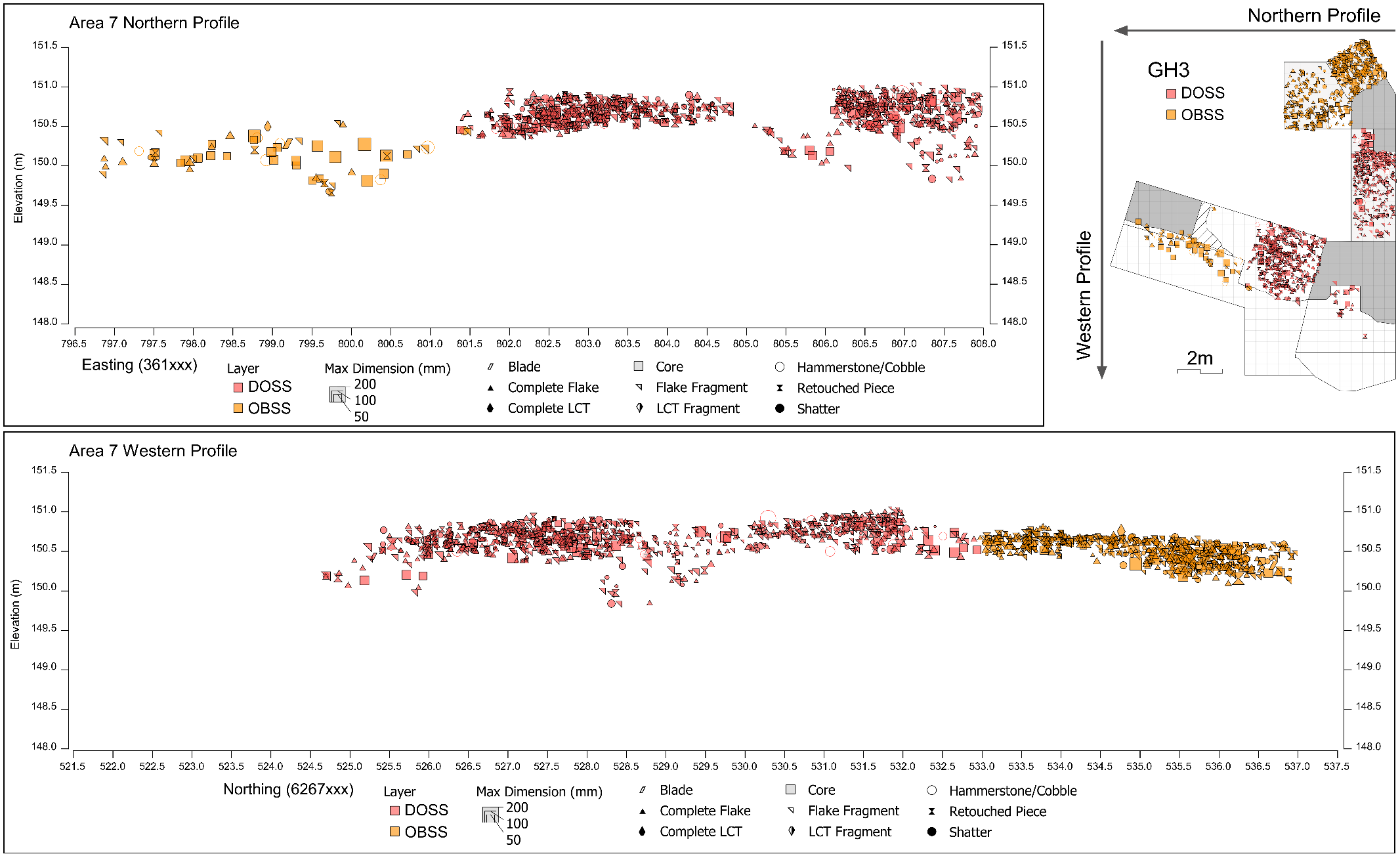
**

Fig. S26. Area 7 northern and western profile views**:** Distribution of artefact types in geological horizon 2 (DOSS and OBSS).

**
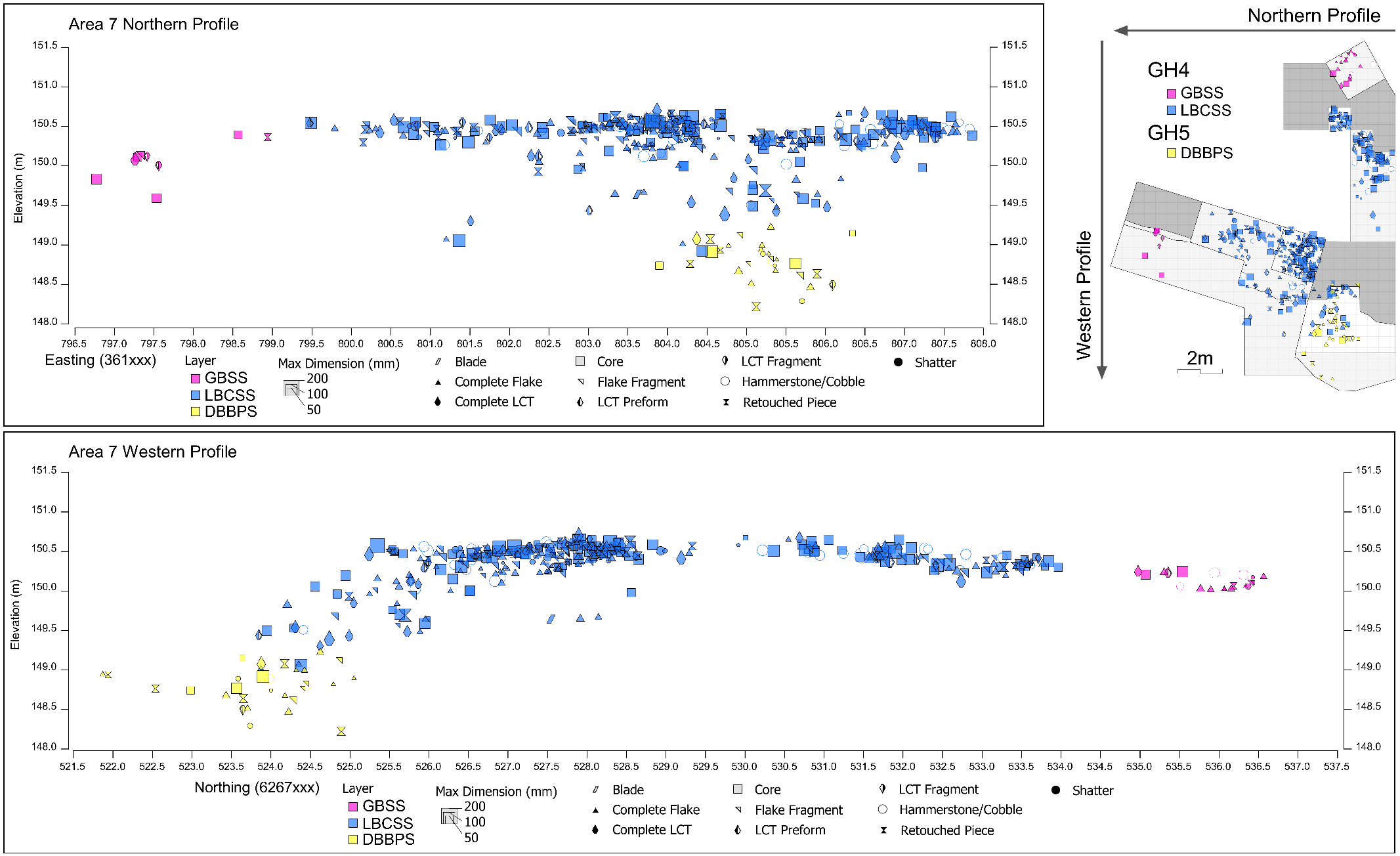
**

Fig. S27. Area 7 northern and western profile views**:** Distribution of artefact types in geological horizon 4 (GBSS and LBCSS) and 5 (DBBPS).


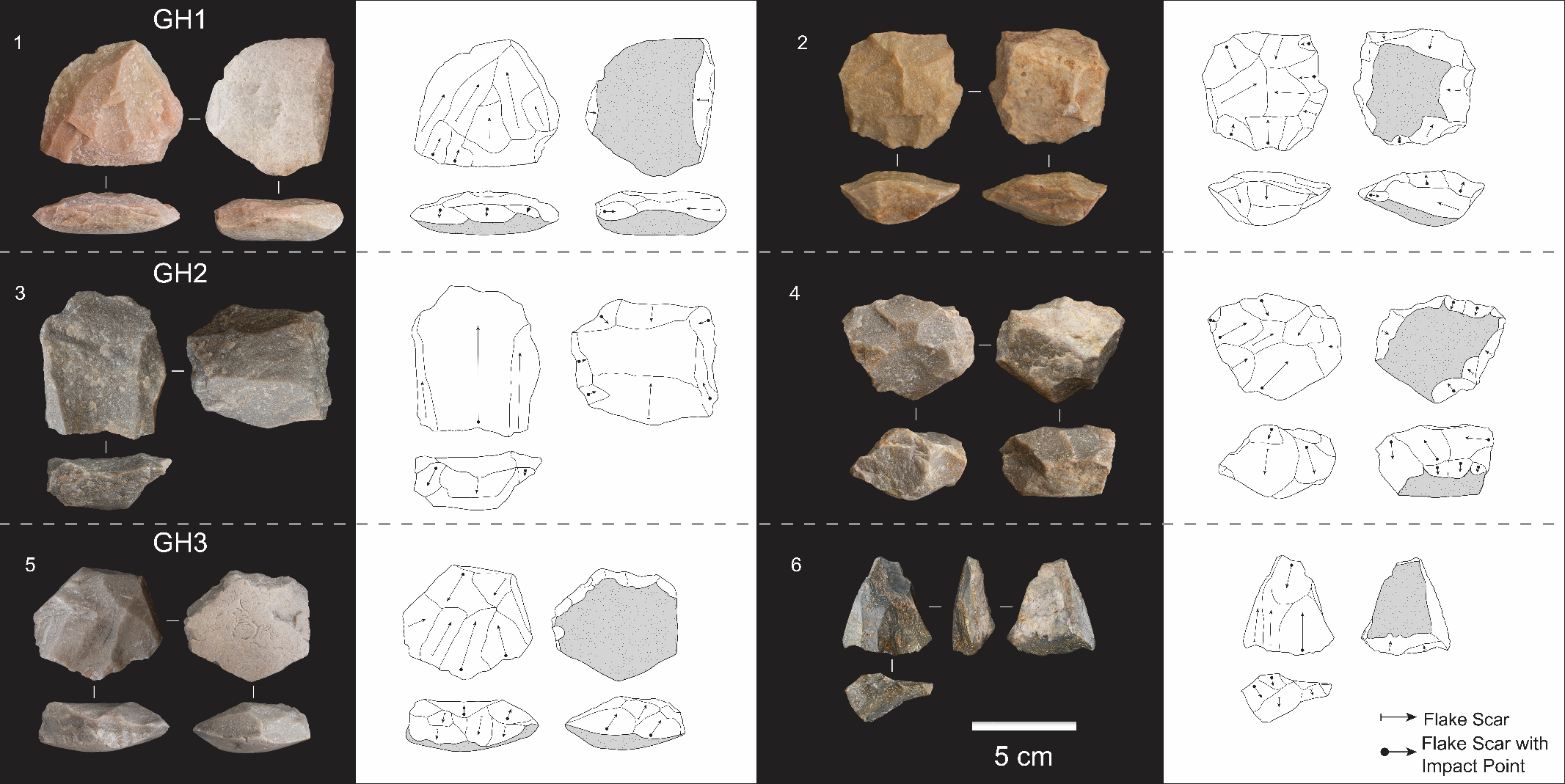


Fig. S28. Prepared cores from the upper deposit at Area 7. 1) Recurrent unidirectional core, and 2) recurrent centripetal core from GH1 (LGSS). 3) Preferential unidirectional core, and 4) recurrent centripetal core from GH2 (LGPSS). 5-6) Recurrent bidirectional cores from GH3 (DOSS and OBSS respectively).


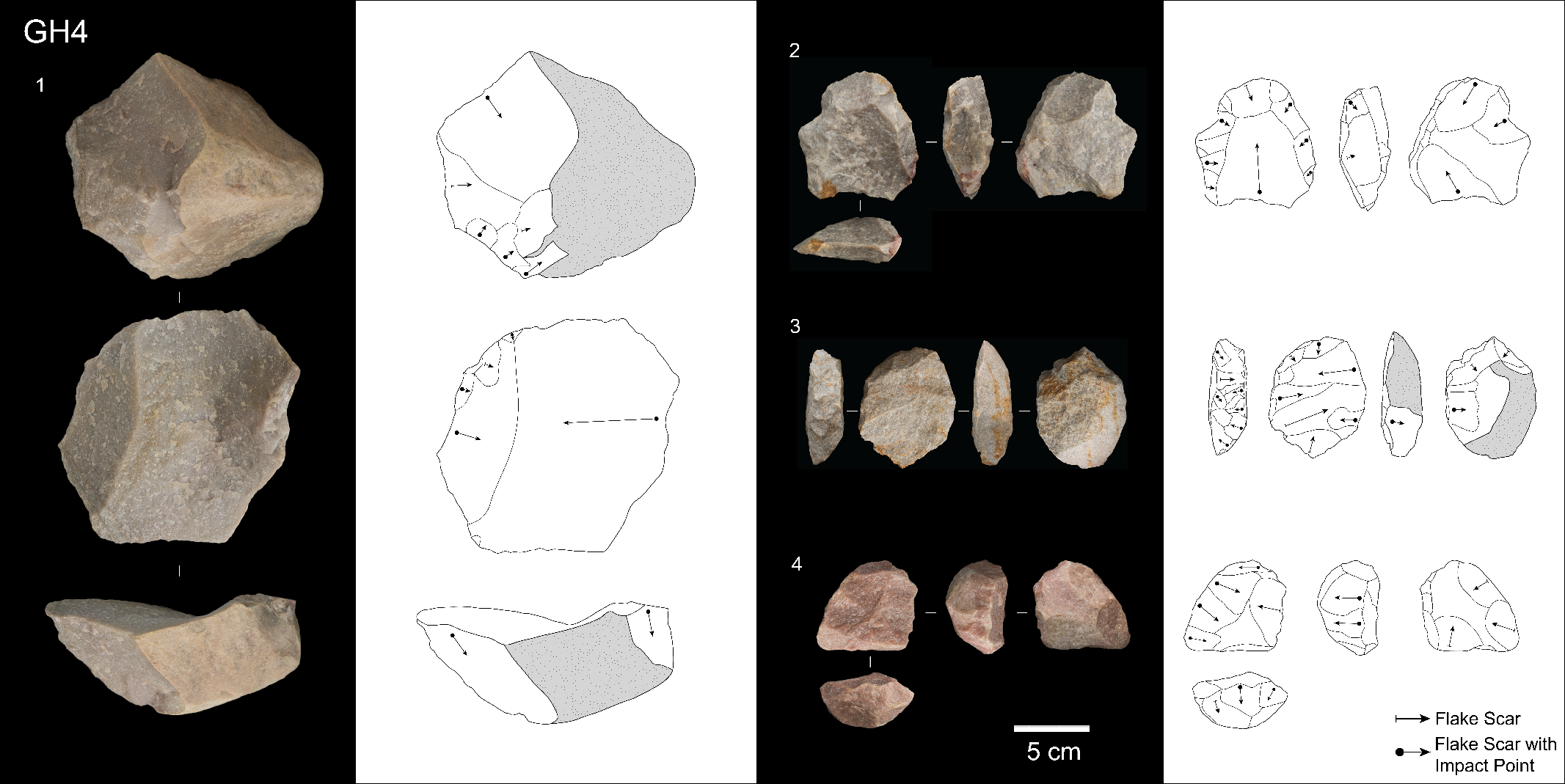


Fig. S29. Bifacial hierarchical cores from the lower deposit at Area 7**.** 1) A large preferential bifacial hierarchical core with bidirectional preparation, 2) preferential centripetal core, 3) recurrent centripetal core, and 4) recurrent bidirectional core. All cores from LBCSS.


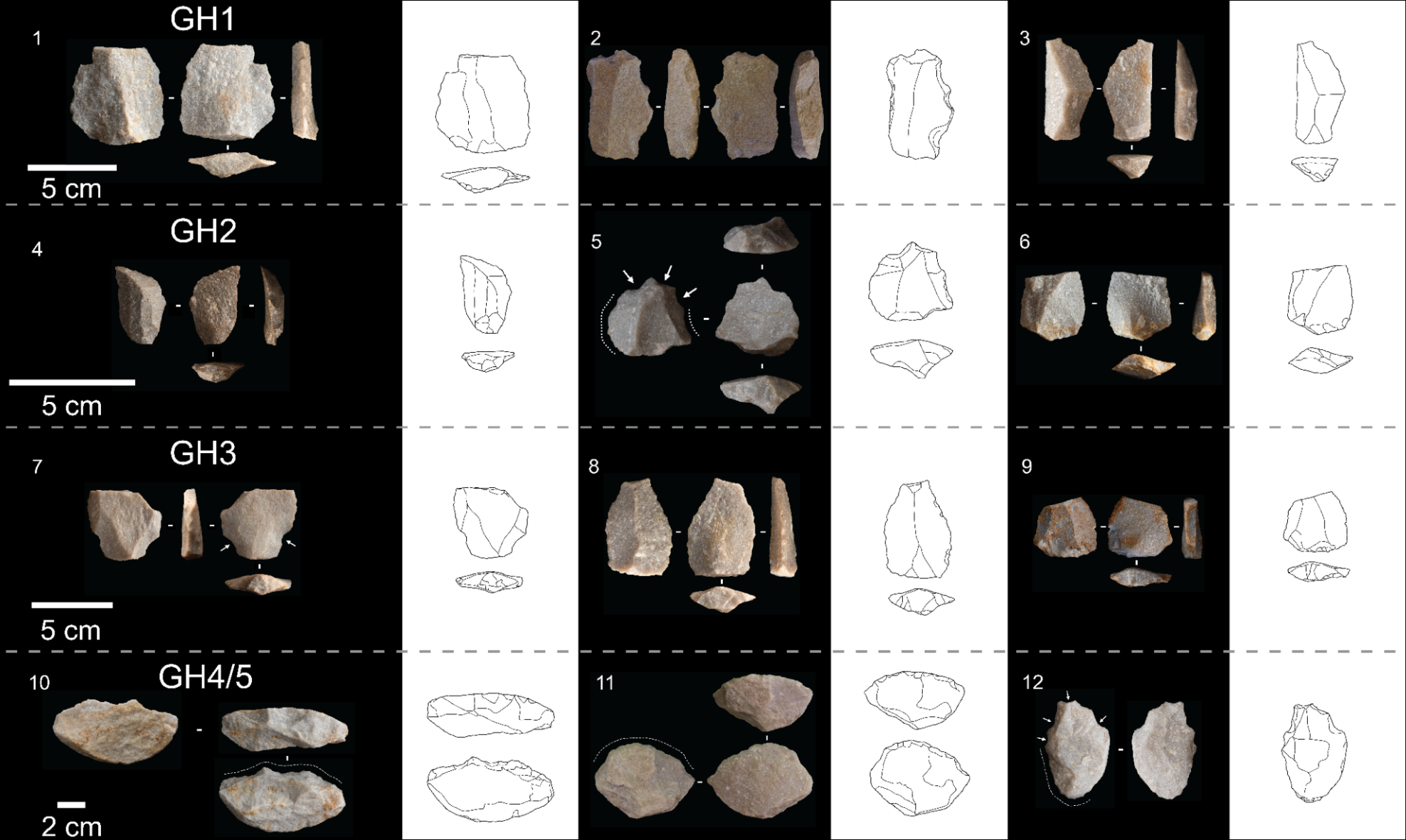


Fig. S30. Debitage and retouch from the Area 7 sequence**.** 1-3) Proximal fragment of a triangular flake, a denticulate made on silcrete, and a blade from GH1 (LGSS). 4-6) Silcrete flake, a notched denticulate, and a proximal fragment of a triangular flake from GH2 (LPGSS). 7-9) Proximal fragment of a triangular flake with evidence of basal notching, triangular flake with tip damage, and proximal fragment of a triangular flake from GH3 (DOSS). 10-11) Naturally backed scraper and end scraper from GH4 (LBCSS), and 12) notched piece from GH5 (DBBPS).


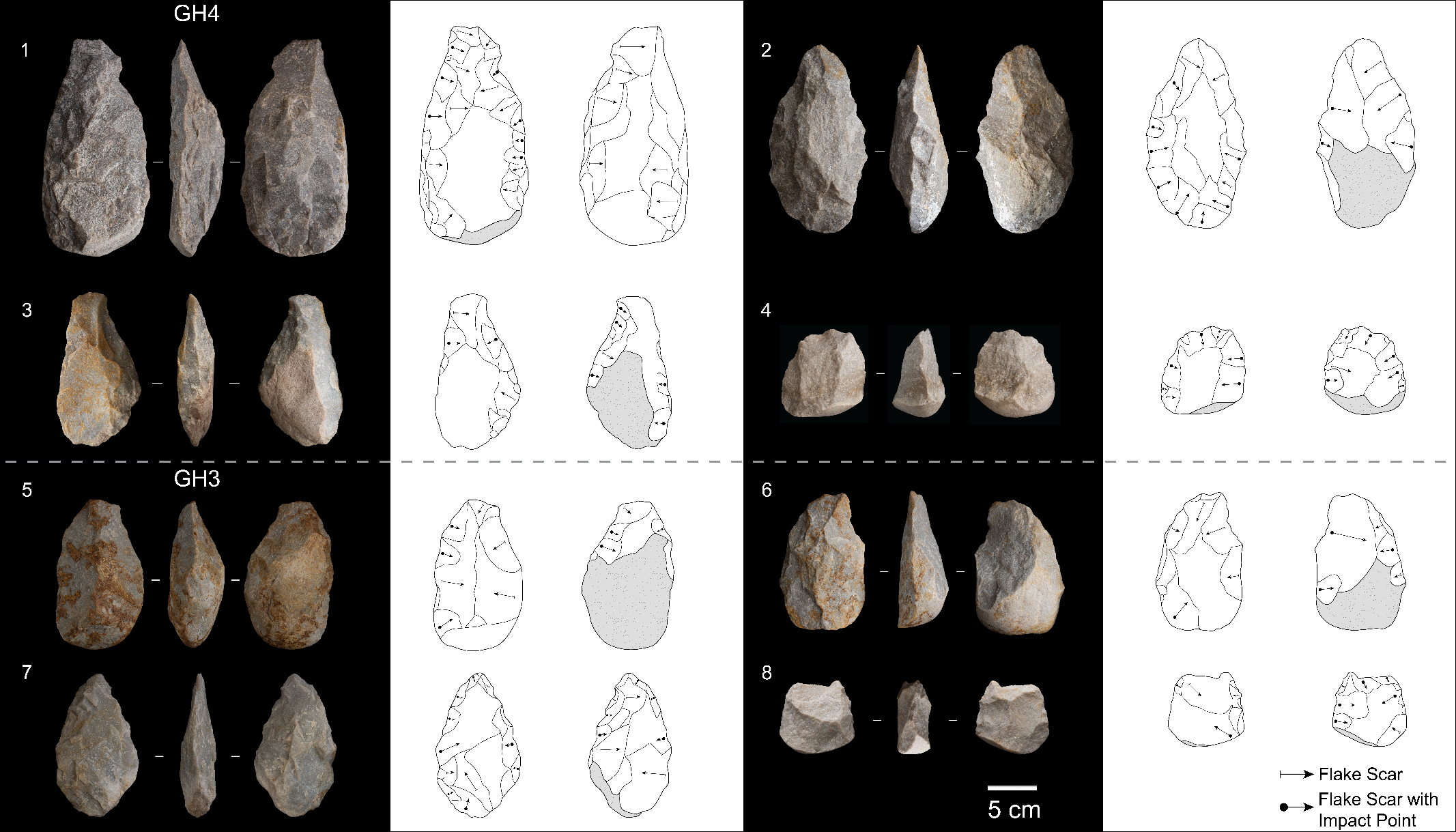


Fig. S31. Large cutting tools from Area 7**.** 1-2) Bifacial handaxes, 3) bifacial cleaver, and 4) the base of a cleaver fragment from GH4 (LBCSS). 5-6) Bifacial handaxes from GH3 (OBSS), and 7-8) bifacial handaxe and cleaver fragment from GH3 (DOSS).

5.4 Platforms

Table S13 provides a summary of platform types at Area 7. Platforms are mostly unprepared, represented by plain (71.6%), cortical (9.1%), crushed (4.2%), and focal (2.9%) types. Platform preparation, in the form of faceted and dihedral platforms, is rare in the lower deposit but increases in the upper deposit. In the lower deposit (GH4-5), unprepared platforms are mostly plain or cortical, with the proportion of cortical platforms highest in GH4. Platform maintenance is less common in these layers, and mostly consists of dihedral platform types, with only two examples of platform faceting in GH4. Platform preparation and maintenance is more common in the upper deposit (GH1-3), with chi-square tests confirming a large increase in the frequency of faceted and dihedral platforms between the GH1-3 and GH4-5 layers (faceted: *X^2^* = 111.02, *df* = 3, *p*= <.0001; dihedral: *X^2^* =72.277, *df* = 4, *p*= <.0001). There is also a decrease in the proportion of cortical platforms, while the percentage of crushed, focal, and plain platforms all increase.

5.5 Retouch

Table S14 provides a summary of the retouched debitage, organised into typological categories based on the form, location, and extent of retouch. Retouched debitage was most common in GH3 (DOSS-OBSS), followed by GH2 (LPGSS), and GH4 (LBCSS), with lower numbers found in GH1 (LGSS) and GH5 (DBBPS). The retouched implements classified here consist of notched pieces (39%), denticulates (6%), scrapers (23%), and other retouch types (33%). The ‘other retouched piece’ type incorporates variation in technological characteristics that do not fit into the other categories. Denticulates are a specific type of notched piece and are typically defined as artefacts with two or more adjacent notches ^110–112^. However, it has been noted that there is no clear definition of what constitutes ‘adjacent’ within this description ^110^, and there is the additional issue that some notches may be the result of edge damage and taphonomy, rather than intentional retouch ^113,114^. To address these issues, the definition of denticulate used here was narrowed to only include artefacts with three or more adjacent notches along the same retouched edge, that are no further apart than the diameter of the largest notch. This strict definition has the effect of excluding those notched pieces with only two adjacent notches, which are instead classified under the notched piece sub-type ‘multiple notched piece’, and narrows the typology to artefacts where denticulated retouch was the focus of reduction (*sensu* ^115^), reducing the likelihood that the edge modification was incidental.

To date, no complete unifacially or bifacially retouched points have been found at Area 7. Except for a bifacial ‘knife’ (Fig. 4) from GH4 in the lower deposit, no other examples of invasive bifacial retouch (other than the LCTs) were found. Retouched implements were mostly made on quartzite (85%), followed by silcrete (6%), and sandstone (5%) flakes, with equal numbers of FGS (2%) and chert (2%), and one retouched quartz artefact (1%). The majority did not have any cortex (71%), with dorsal scar patterns consisting mostly of bi- or unidirectional ridges (73%), compared to sub-radial or radial patterns (27%). A large proportion of the retouched debitage consists of proximal flakes (36%), followed by complete flakes (30%), fragments (17%), split flakes (8%), and distal (5%) or medial (4%) pieces. The ‘other retouch’ type showed the most variation in the types of retouch scars observed on tool edges, reflecting the variable and irregular nature of these artefacts. Notched pieces and denticulates also displayed a broad range of other retouch types, including feathered, stepped, and parallel scars, and one example with serrations. The retouch types recorded on scrapers were the most limited, consisting of feathered, parallel, and scalar retouch scars. No artefacts exhibited backing retouch. Across all implement types, retouch was mostly applied to the dorsal surface of flakes from ventral initiations, while ventrally invasive retouch scars were less common.

Table S13**.** Platform types for all proximally preserved debitage.

| **Layer** | **Platform Type** | | | | | | | | | | | | **Total** | **% Total** |
| --- | --- | --- | --- | --- | --- | --- | --- | --- | --- | --- | --- | --- | --- | --- |
|  | **Plain** | | **Dihedral** | | **Faceted** | | **Focal** | | **Crushed** | | **Cortical** | |  |  |
|  | ***n*** | ***%*** | ***n*** | ***%*** | ***n*** | ***%*** | ***n*** | ***%*** | ***n*** | ***%*** | ***n*** | ***%*** |  |  |
| LGSS | 83 | *73%* | 7 | *6%* | 10 | *9%* | 7 | *6%* | 2 | *2%* | 4 | *4%* | *113* | 6.3% |
| LPGSS | 415 | *75%* | 24 | *4%* | 32 | *6%* | 19 | *3%* | 28 | *5%* | 38 | *7%* | *556* | 31% |
| DOSS | 422 | *74%* | 32 | *6%* | 39 | *7%* | 20 | *4%* | 22 | *4%* | 35 | *6%* | *570* | 31.8% |
| OBSS | 238 | *66%* | 17 | *5%* | 37 | *10%* | 4 | *1%* | 13 | *4%* | 52 | *14%* | *361* | 20.1% |
| GBSC | 4 | *44%* | 1 | *11%* | - | - | - |  | - | - | 4 | *44%* | *9* | 0.5% |
| LBCSS | 109 | *67%* | 13 | *8%* | 2 | *1%* | 2 | *1%* | 9 | *6%* | 27 | *17%* | *162* | 9.0% |
| DBBPS | 12 | *57%* | 5 | *24%* | - | - | - | - | 1 | *5%* | 3 | *14%* | *21* | 1.2% |
| **Total** | **1283** | | **99** | | **120** | | **52** | | **75** | | **163** | | **1792** | 100% |
| **% Total** | ***71.6%*** | | ***5.5%*** | | ***6.7%*** | | ***2.9%*** | | ***4.2%*** | | ***9.1%*** | | ***100%*** |  |

Table S14**.** Retouched implements identified at Area 7.

| **Artefact Type** | **GH1** | | **GH2** | | **GH3** | | | | **GH4** | | | | **GH5** | | **Total** |
| --- | --- | --- | --- | --- | --- | --- | --- | --- | --- | --- | --- | --- | --- | --- | --- |
|  | **LGSS** | | **LPGSS** | | **DOSS** | | **OBSS** | | **GBSS** | | **LBCSS** | | **DBBPS** | |  |
|  | ***n*** | **%** | ***n*** | **%** | ***n*** | **%** | ***n*** | **%** | ***n*** | **%** | ***n*** | **%** | ***n*** | **%** |  |
| **Notched Pieces** |  |  |  |  |  |  |  |  |  |  |  |  |  |  |  |
| Simple Notch | 1 | *33%* | 8 | *50%* | 8 | *50%* | 10 | *67%* | - | - | 4 | *100%* | 2 | 100*%* | *33* |
| Complex Notch | 1 | *33%* | 3 | *19%* | 7 | *44%* | 5 | *33%* | - | - | - | *-* | - | *-* | *16* |
| ***Subtotal*** | ***2*** | ***22%*** | ***11*** | ***41%*** | ***15*** | ***44%*** | ***15*** | ***52%*** |  |  | ***4*** | ***20%*** | ***2*** | ***40%*** | ***49*** |
| **Denticulates** |  |  |  |  |  |  |  |  |  |  |  |  |  |  |  |
| Notched Denticulate | 1 | *33%* | 4 | *25%* | 1 | *6%* | - | *-* | - | - | - | *-* | - | *-* | *6* |
| Serrated Denticulate | - | *-* | 1 | *6%* | - | *-* | - | *-* | - | - | - | *-* | - | *-* | *1* |
| ***Subtotal*** | ***1*** | ***11%*** | ***5*** | ***19%*** | ***1*** | ***3%*** | ***0*** | ***0%*** |  |  | ***0*** | ***0%*** | ***0*** | ***0%*** | ***7*** |
| **Scrapers** |  |  |  |  |  |  |  |  |  |  |  |  |  |  |  |
| End Scraper | - | - | 6 | 75% | 3 | 33% | 2 | 40% | 1 | 50% | 2 | 40% | - | - | *14* |
| Side Scraper | - | - | 2 | 25% | 1 | 11% | 1 | 20% | - | - | 2 | 40% | - | - | *6* |
| Other Scraper | - | - | - | - | 5 | 56% | 2 | 40% | 1 | 50% | 1 | 20% | - | - | *9* |
| ***Subtotal*** | ***0*** | ***0%*** | ***8*** | ***30%*** | ***9*** | ***26%*** | ***5*** | ***17%*** | ***2*** | ***100%*** | ***5*** | ***25%*** | ***0*** | ***0%*** | ***29*** |
| **Other Types** |  |  |  |  |  |  |  |  |  |  |  |  |  |  |  |
| Laterally Retouched | 3 | *50%* | 1 | *33%* | 7 | *78%* | 6 | *67%* | - | - | 9 | *82%* | 3 | *100%* | *29* |
| Minimally Retouched | 2 | *33%* | 1 | *33%* | 2 | *22%* | 3 | *33%* | - | - | 2 | *18%* | - | - | *10* |
| Single Awl | 1 | *17%* | 1 | *33%* | - | *-* | - | *-* | - | *-* | - | *-* | - | *-* | *2* |
| ***Subtotal*** | ***6*** | ***67%*** | ***3*** | ***11%*** | ***9*** | ***26%*** | ***9*** | ***31%*** |  |  | ***11*** | ***55%*** | ***3*** | ***60%*** | ***41*** |
| **Total** | **9** |  | **27** |  | **34** |  | **29** |  | **2** |  | **20** |  | **5** |  | **126** |

5.6 Cores

The Area 7 lithic samples include a relatively high proportion of cores, accounting for 7.1% (n=249) of all artefacts and between 3-22.9% of the total lithic sample from each layer (Table S15). The highest number of cores were found in the LBCSS (40.2%), followed by the OBSS (23.3%), DOSS (17.7%), and LPGSS (11.6%) layers. The LGSS (3.2%), GBSS (2.4%), and DBBPS (1.6%) layers contained the fewest cores. The proportion of cores to the total lithic sample is greater in the lower deposit (GH4-5) than in the upper deposit (GH1-3).

Five main core reduction strategies were identified, including unifacial (32.9%), bifacial (16.5%), discoidal (22.1%), bifacial hierarchical (6.4%), and Levallois (4.8%) (Table S15). Blade cores were absent from all layers except for a single example in the DOSS layer and a core fragment from LPGSS (0.8% of all cores). Several cores made on flakes were identified (n=8, 3.2%), and core fragments were present in most layers (13.3%). Bifacial cores (including discoids) are more common than unifacial cores in all layers except for the LBCSS, where unifacial cores occur in higher numbers. All the cores present in the Area 7 assemblage were knapped using free-hand hard-hammer percussion.

Table S15. Summary of core types by layer for the Area 7 sequence (B. = Bifacial). % TLS is the percentage of the total lithic sample recovered from each stratigraphic layer.

| **Core Type** | **GH1** | **GH2** | **GH3** | | **GH4** | | **GH5** | **Total** | **%** |
| --- | --- | --- | --- | --- | --- | --- | --- | --- | --- |
|  | **LGSS** | **LPGSS** | **DOSS** | **OBSS** | **GBSS** | **LBCSS** | **DBBPS** |  |  |
| Unifacial | 1 | 9 | 13 | 16 | 1 | 41 | 1 | *82* | *32.9%* |
| Bifacial | 3 | 6 | 3 | 7 | 1 | 20 | 1 | *41* | *16.5%* |
| Discoid | - | 6 | 9 | 14 | 4 | 22 | - | *55* | *22.1%* |
| B. Hierarchical | 1 | 1 | 4 | 5 | - | 5 | - | *16* | *6.4%* |
| Levallois | 2 | 2 | 4 | 4 | - | - | - | *12* | *4.8%* |
| Blade | - | 1 | 1 | - | - | - | - | *2* | *0.8%* |
| Core On Flake | - | 2 | 2 | 3 | - | - | 1 | *8* | *3.2%* |
| Core Fragment | 1 | 2 | 8 | 9 | - | 12 | 1 | *33* | *13.3%* |
| **Total** | **8** | **29** | **44** | **58** | **6** | **100** | **4** | ***249*** | ***100%*** |
| **% Total Cores** | ***3.2%*** | ***11.6%*** | ***17.7%*** | ***23.3%*** | ***2.4%*** | ***40.2%*** | ***1.6%*** | ***100%*** |  |
| **% TLS** | ***4.5%*** | ***3.0%*** | ***3.7%*** | ***8.3%*** | ***19.4%*** | ***22.9%*** | ***11.1%*** | ***7.1%*** |  |

A total of 16 bifacial hierarchical cores were found in the LBCSS, OBSS, DOSS, LPGSS, and LGSS layers (Table S15; Figs. S28-29). These cores are characterised by two asymmetrical and hierarchically organised core surfaces, with a primary flake detachment surface from which the largest flakes were detached and a subordinate surface that functioned as the striking platform. Core volumes and striking platforms were managed via hierarchically sequenced core rotations, with flakes detached from the primary core surface in either a recurrent (69%), or preferential (31%) manner and mostly parallel or subparallel to the plane of intersection (87.5%), but occasionally at secant angles (12.5%). The subordinate striking platform surface was often the larger of the two volumes and was maintained via flake removals that were detached roughly perpendicular to the plane of intersection. Flakes were struck from the primary core surface on the recurrent hierarchical cores in a continuous sequence, using the ridges produced by previous removals to control the shape and size of each subsequent flake. The flake detachment surface was thus prepared, and convexities maintained, by the previous series of removals. These cores had on average more flake scars at the time of discard than preferential hierarchical cores, and featured longer reduction sequences than the unifacial, bifacial, and discoidal reduction strategies at Area 7. On the preferential hierarchical cores, the primary detachment surface was prepared by a series of flakes in a centripetal or orthogonal pattern, prior to the detachment of one large final flake. These cores generally preserved fewer flake scars, due to the presence of the large negative scar from the preferential flake, although as with the recurrent reduction strategies the average flake scar count remained higher than most other core types at Area 7. In both methods, striking platforms were on the subordinate core face, although only occasionally showed evidence of faceting.

A total of 12 Levallois cores, including two fragments, were found in the GH3 (OBSS-DOSS), GH2 (LPGSS), and GH1 (LGSS) layers (Table S15). Levallois reduction at Area 7 was highly flexible, with the final morphology of the discarded cores characterised by recurrent exploitation using unidirectional (33%, including one unidirectional-convergent), bidirectional (8%), and centripetal (42%) methods. Only one preferential core was found, in GH2. The Levallois cores were mostly made on quartzite (66.7%), however there was considerably more use of non-quartzite raw materials than any of the other core reduction methods, including a silcrete core from the DOSS, and cores made on silcrete, FGS, and an igneous lithology from the OBSS layer. The Levallois cores were made on cobbles (41.7%), or slabs/tabular pieces (16.7%), although many did not preserve diagnostic features of the original core-blank (41.7%).

The Area 7 cores also provide evidence for technological continuity through time, particularly in the unifacial, bifacial, and discoid methods. The shape of the cores within each of these types did not change significantly throughout the deposit, although there was an increase in the frequency of the smaller, more intensively exploited core types. The main elements of technological change observed were in the relative abundance of the different core reduction strategies, in particular the introduction of the Levallois method in the GH3 layers, reflecting an increased emphasis on prepared cores in the upper deposit. Core reduction strategies in the upper deposit (GH2-3) continued to be dominated by the unifacial, bifacial, and discoid methods that characterised the Acheulian layers, although the relative frequency of these core type does decrease and by MIS 5 in the LGSS layer (GH1) discoid cores are absent. No diachronic changes were detected in the morphology of the cores *within* each of these reduction strategies, as expressed by their elongation (length/width), width/thickness, and length/thickness ratios, and confirmed by a MANOVA test on these variables by layer (GH4/5-GH3: *p*=.3407; GH4/5-GH2: *p*=.6273; GH3-GH2: *p*=.2996). However, the cores produced by each of the reduction strategies differ substantially in morphology to *each other*, as attested to by a MANOVA test of elongation, width/thickness, and length/thickness ratios by reduction strategy (*p*=<.001). The observed continuity through time in the shape and size of cores *within* each reduction strategy is thus likely to be connected to the sequence of planned actions involved in knapping these cores, as well as raw material constraints imposed by the dominant use of quartzite cobbles.

5.7 Evidence for On-site Reduction

Unretouched flake blanks are the most abundant type of complete debitage at Area 7 and are divided into three main categories based on the proportion of cortex located on their dorsal surfaces: initial cortical (>61% dorsal cortex), residual cortical (<60% dorsal cortex), and non-cortical flakes (0% dorsal cortex). Given that quartzite, the main raw material used at Area 7, mostly occurs locally as cobbles that are entirely covered in cortex, it is expected that the initial stages of core reduction will produce flakes with cortical dorsal surfaces that are relatively large in relation to the overall size of the clast, while flakes produced later in the reduction sequence will be smaller and mostly non-cortical ^116,117^. In addition, because flakes with cortical platforms are generally removed earlier in the reduction sequence, they are also expected to be larger than those with non-cortical platforms.


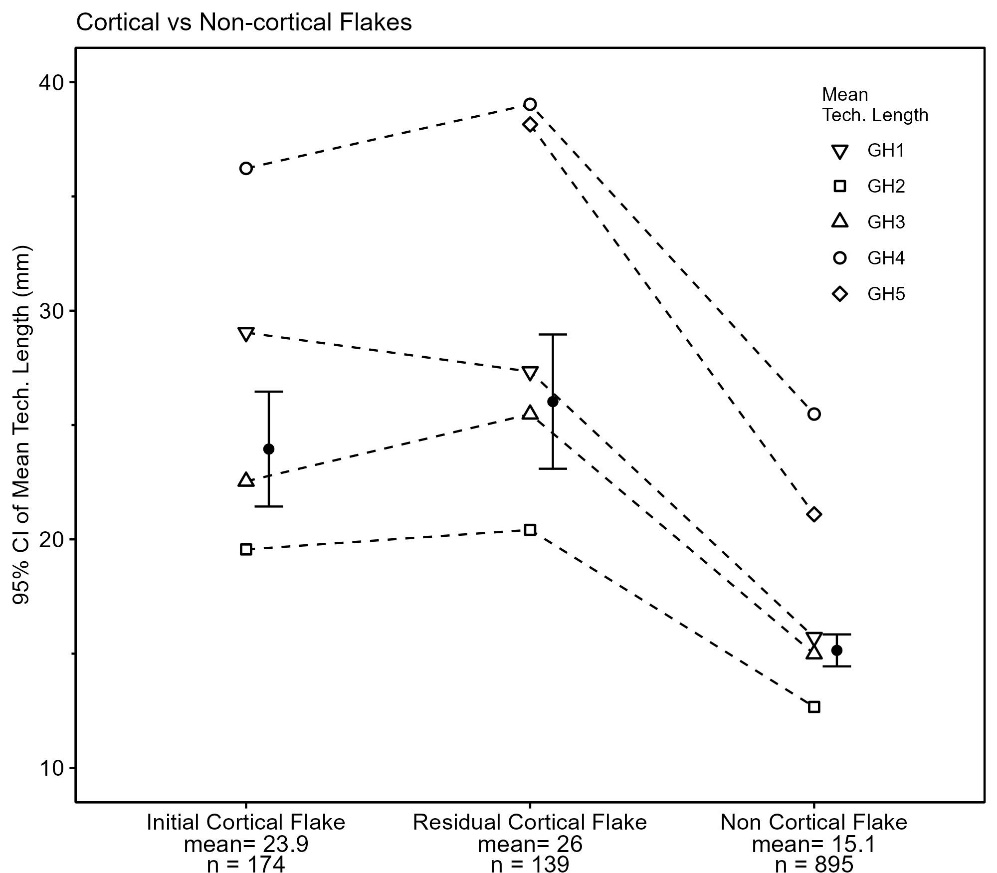


Fig. S32. Evidence for on-site reduction**.** Error bars showing the 95% confidence interval of mean technological length of all initial, residual, and non-cortical flakes, with the mean technological length of all complete debitage in each category indicated for each geological horizon.

Of the total sample of unretouched flake blanks (*n*=1216), 14% were detached during the early stages of core reduction (initial cortical flakes), 12% were detached after the initial sequence of flake removals and retain some degree of cortex (residual cortical flakes), and 74% were produced during the later stages of core reduction and have no cortex. The proportion of initial to residual cortical flakes varies between layers, although initial cortical flakes are always more numerous than residual cortical flakes. The length of initial cortical flakes is smaller than residual cortical flakes across all samples except GH1, although they overlap at the 95% confidence intervals centred on the mean values (Fig. S32). There is strong evidence that non-cortical flakes are shorter in length than both initial (*t*(201)=6.66, *p*=<.0001), and residual (*t*(154)=-7.12, *p*=<.0001) cortical flakes. The frequency and size difference of these three categories of unretouched flakes supports the interpretation that primary core reduction was undertaken on site, with the largest flake blanks being produced after initial core shaping, once ridges and convexities were established on the core detachment surfaces.

On an assemblage level, when all complete flakes, blades, and retouched pieces are compared, flakes with dorsal cortex are larger in maximum dimension than non-cortical flakes in every layer (Fig. S33). Independent samples t-tests and Mann-Whitney U tests confirm this for all samples except the GBSS and DBBPS, which is likely due to the very small sample sizes as the boxplots show the same pattern (Fig. S33). When compared by raw material type, the technological length of cortical flakes is larger than non-cortical flakes made on quartzite (*t*(439)=8.86, *p*=<.0001), while all other lithologies show non-significant differences (Fig. S33b). Small sample size is a potential limitation for FGS and chert/CCS flakes, however this is not the case for silcrete which exhibits a non-significant difference between the length of cortical and non-cortical flakes (*U*(1128), *p*=.0981). Finally, quartzite flakes with cortical platforms are substantially longer than those with non-cortical platforms (*t*(165)=2.86, *p*=.0142), while both sandstone and silcrete flakes are not. Cortical platforms are rarely present on other raw materials (Fig. S33c). The available evidence thus indicates that quartzite cobbles were flaked at the site, with the on-site reduction of quartzite cobbles being the focus of core reduction activities throughout the occupation history of the Area 7 spring. In contrast, the non-quartzite raw materials introduced into the site in the GH1-3 layers appear to have been initially reduced elsewhere and transported to the site, suggesting that operational sequences during the early MSA incorporated greater landscape mobility and different provisioning behaviours.


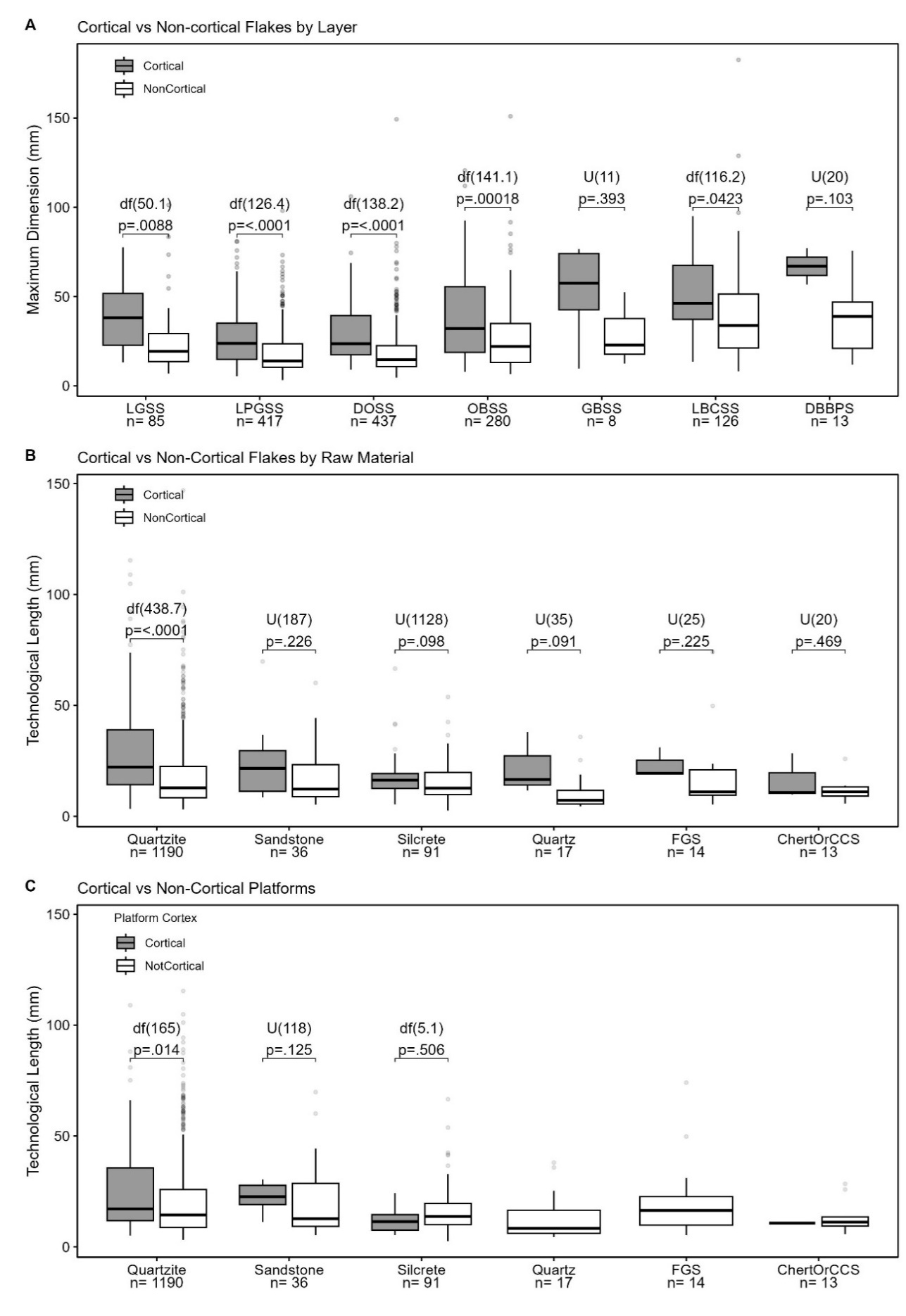


Fig. S33. Cortical vs non cortical complete flakes, blades, and retouched pieces with t-tests (df) and Mann-Whitney U tests (U) indicated, showing (a) maximum dimension of cortical vs non-cortical flakes by layer, (b) technological length by raw material, and (c) cortical vs non cortical platforms by raw material.

5.8 Edge length / mass (EL/M) Ratios

Given the overall reduction in the size of complete flakes through time (Fig. 3a and Fig. S32), it is worth examining if there is a corresponding change in the efficiency of flake production, or if the changes in flake dimensions are simply a matter of scale. The ratio of edge length to mass (EL/M) is used as a proxy for flaking efficiency by estimating the amount of usable cutting edge on each flake in relation to the amount of mass that was removed from a given core ^118–120^. Higher EL/M ratios indicate greater potentially useable cutting edge per unit of mass, while lower ratios indicate greater mass and less cutting edge. At Area 7, EL/M values are highest in the DOSS and LPGSS samples, followed by the OBSS and LGSS. The EL/M values of these four layers are higher than the DBBPS, LBCSS, and GBSS layers (*t*(176.6)=8.62, *p*=<.0001), indicating an overall increase in the amount of flake edge produced per unit of mass in the upper deposits. This also appears to correlate with raw material type, as the highest EL/M values observed were on silcrete and FGS/chert flakes, which are more abundant in the upper deposit (Fig. S34). The latter two raw materials are combined in Fig. S34 due to the small individual sample sizes and similar distribution of means and confidence intervals, in addition to general similarities in conchoidal fracture properties. Quartz flakes also exhibit higher EL/M values than quartzite and sandstone, although the variation is greater due to the small size of the sample. When EL/M ratios are compared by platform type, flakes with faceted platforms display the lowest values, with dihedral and cortical platforms overlapping with the upper range of faceted, while focal, crushed, and plain platforms exhibit the highest EL/M values (Fig. S34c). This appears to track well with mean platform thickness, which is highest for faceted, dihedral, and cortical platforms. EL/M ratios also appear to be correlated with artefact shape, as indicated by higher values for flakes with convergent lateral margins (*t*(651.3)=-4.51, *p*=<.0001), with all other edge shapes exhibiting overlapping mean values at the 95% confidence interval.

Finally, to examine whether the differences in flake size and EL/M ratios between layers is related to the greater proportion of small flaking debris in the upper deposit (GH1-3), EL/M ratios are compared for flakes >20 mm in technological length (Fig. S35). Although the sample size in the upper layers is considerably reduced and the mean values now plot closer together, mean flake length remains lower, and EL/M values higher, in the GH1-3 layers.


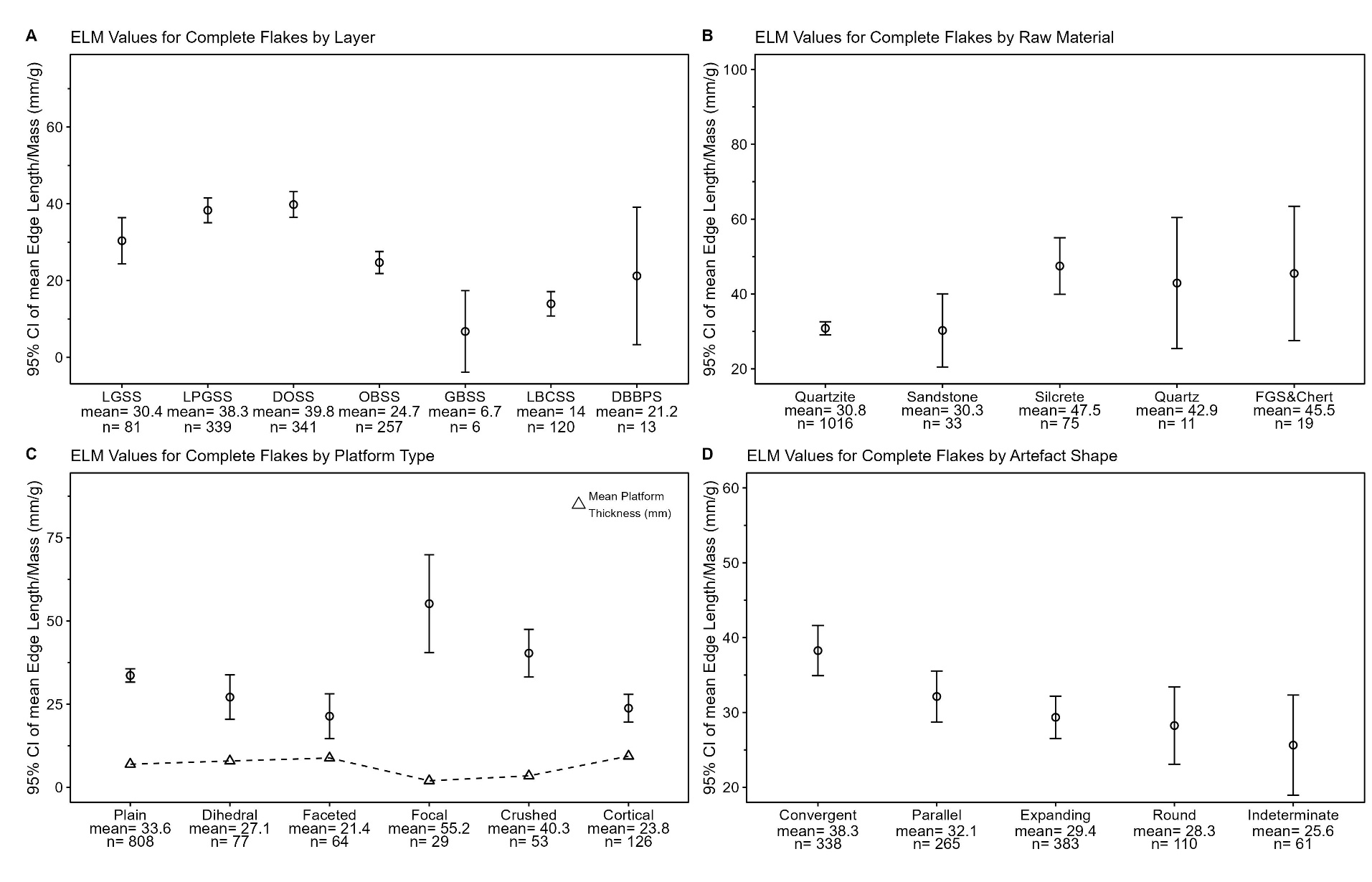


Fig. S34. Edge length to mass (EL/M) ratio of complete unretouched flakes, showing confidence intervals around mean EL/M by (a) layer, (b) raw material, (c) by platform type, and (d) by shape of lateral edges.


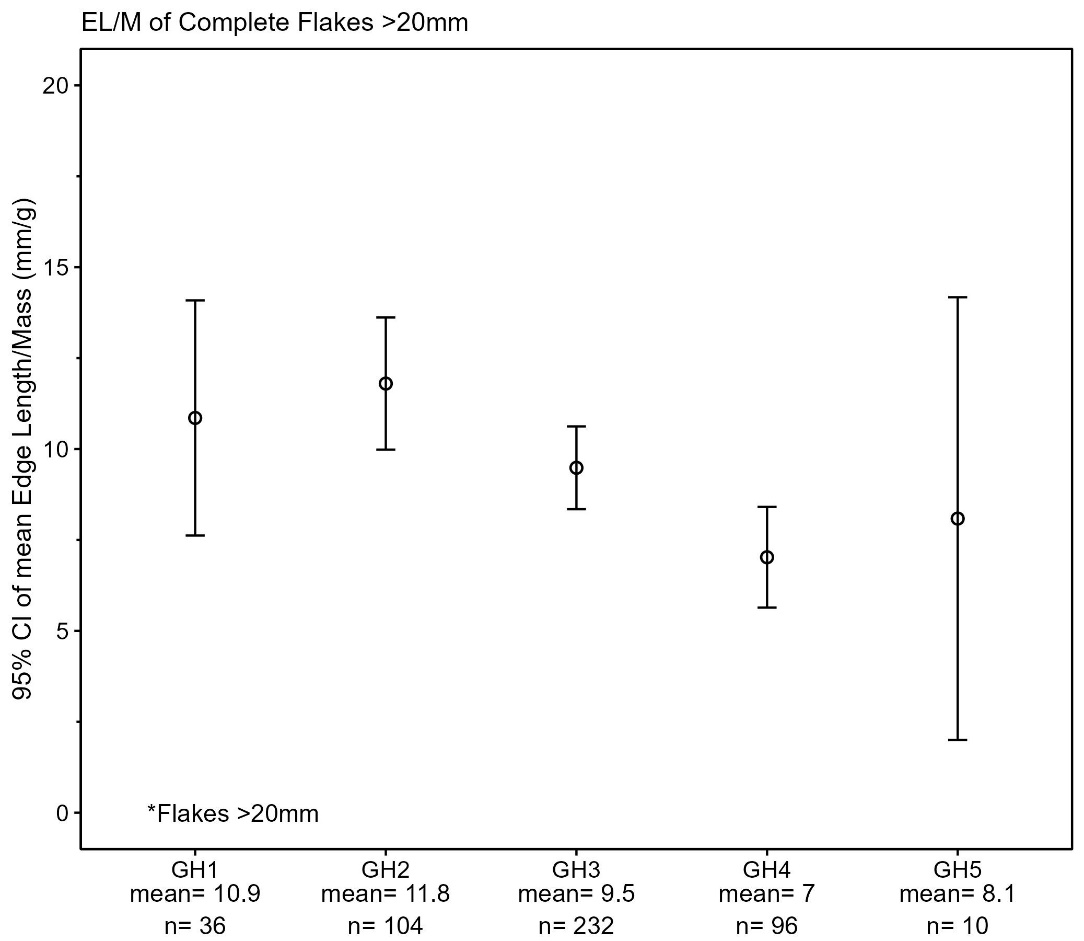


Fig. S35. Confidence intervals for EL/M ratios of complete flakes, blades, and retouched pieces >20 mm in max dimension.

5.9 Artefact Weathering Patterns

Artefact weathering was recorded using descriptions of weathering patterns that included smoothed arises and rounded edges, chemically weathered or decayed, patination, and damaged. Smoothed arises and rounded edges refers to the reduction in relief of flake scar ridges and sharpness of edges on artefacts. This type of weathering, also sometimes referred to as abrasion ^121–123^, can be caused by many different processes depending on the context in which the artefacts were found. It is often associated with mechanical weathering (i.e., erosion) caused by high velocity windblown sediment, referred to as wind blasting or scouring, or fluvial processes such as abrasion by gravels or sedimentary particles in flowing water ^123–125^. Chemical weathering and decay refer to the deterioration or leaching of raw materials caused by exposure to UV light or acidic/alkaline groundwater or sediments ^126^. Chemical weathering in particular affects lithologies such as hornfels and chert and will normally appear as a pale coloured ‘rind’ covering either a surface or the entire artefact and penetrating partially or fully into the internal structure of the rock. The development of a rind is less likely to occur on sandstone and quartzite lithologies, and degradation of these materials at Area 7 appears to result in the internal structure becoming friable. For this type of chemical weathering, the term decay was used. Patination refers to changes in colour or staining of artefacts and can usually be identified by the presence of different patinas on adjacent artefact surfaces. Even though this is also caused by chemical weathering, it is more commonly the result of accretion, rather than degradation, and at Area 7 appears to be mostly caused by the precipitation of iron oxides and hydroxides held in groundwater ^124–127^. Damaged refers to chipped or broken artefacts that have obvious post-depositional damage, often with a different patina to the rest of the artefact.

The amount of weathering was estimated using a four-stage ordinal scale for artefact surfaces (unweathered, slightly weathered, weathered, highly weathered), and artefact edges (unweathered, slightly dulled, dulled, very dulled). Where evidence of microwear was observed it was checked under magnification and the location recorded for future study. The results presented here represent a qualitative assessment of artefact condition, with the aim of identifying broad weathering patterns that can be further investigated in a dedicated study of artefact condition.

The bulk of artefacts at AMZ7 are unweathered, with 64.5% showing no signs of weathering. The most common weathering patterns observed were smoothing or dulling of arises and edges (30%), various forms of chemical weathering (3.1%), and patinas caused by iron staining (1.9%). Most of the weathered artefacts are only slightly weathered (22.5%) with slightly dulled edges (24.6%), followed by moderately weathered (9.7%) with dulled edges (1.8%). Heavily weathered artefacts are rare (3.3%), as are artefacts with very dulled edges (6.3%), many of which are either chemically weathered or decayed, rather than abraded. Raw materials that most frequently exhibited smoothed arises and rounded edges were sandstone (36%) and quartzite (32%), followed by quartz (21%), and silcrete (12%).

In the upper deposit, some raw materials showed signs of chemical degradation, particularly at the spring margins in the OBSS, LPGSS, and LGSS layers. FGS (62%), hornfels (60%), chert/CCS (52%), and the igneous lithologies (40%) appear to have been particularly susceptible to chemical degradation. Two silcrete artefacts appeared chemically weathered and one decayed, while several artefacts made on sandstone, and to a lesser extent quartzite, also appeared to be leached/decayed. This is most likely caused by the acidity of the groundwater at the spring. Patination appears to have affected quartzite artefacts disproportionately, with patinas relatively rare on other raw materials. This may be due to the quartzites being more resistant to the acidic groundwater, with iron oxides/hydroxides accreting onto artefact surfaces rather than degrading the raw material.

No discrete spatial patterns or specific trends related to depth within the deposit were observed, and weathering patterns are broadly similar between layers. There is a slight increase in the frequency of weathered artefacts in the layers at the spring margins, although this is also where lithic density is the highest, which may be contributing to this pattern. Given the available evidence, the most likely explanation for artefact weathering at Area 7 is spring activity and the acidic and iron-rich nature of the groundwater.

5.10 Artefact Size Distribution

The frequency and size distribution of lithic material produced during core reduction provide information on assemblage composition and post-depositional processes that may have altered the accumulation ^122,126,128–130^. For example, geomorphic processes such as subtractive distortion (winnowing) can remove the small fraction (<20 mm) of lithics produced as a by-product of knapping, potentially skewing assemblage composition towards larger and heavier artefacts. Additionally, artefact size distributions may be influenced by behavioural factors, such as provisioning systems that emphasised transport and conservation of raw material in maintainable toolkits, or on-site reduction and accumulation of manufacturing debris at fixed locations (places) on the landscape ^131,132^. Artefact transport and discard patterns are expected to differ substantially between both provisioning systems, and in the absence of significant post-depositional alteration the archaeological manifestations of these different behaviours should be observable on an assemblage level. However, disentangling the equifinality of the effects of post-depositional sorting and discard behaviours is challenging, and an approach that incorporates analysis of artefact size distribution along with three-dimensional analysis orientation and dip of the artefacts can help to better understand the formation of archaeological sites. This section focuses on assessing the size profiles of lithics within each layer to examine the composition of the samples and investigate post-depositional processes by comparison with expected size distributions developed through lithic manufacturing experiments ^129,133^.

In an experimental study of 107 separate knapping episodes, Schick ^128,129^ collected and recorded all debitage (flakes detached during core reduction) and debris (knapping by-products that include fragments and shatter) that were produced. The experimental artefacts were sieved through a 5 mm screen to simulate archaeological collection procedures, and the maximum dimension of each specimen measured. The results were presented as a density curve with the dataset organised into 1 cm bins, showing that 60-75% of the material produced during the knapping experiments measured less than 2 cm ^128^. The modal distribution is in the 10–20 mm range, resulting in a density plot strongly skewed toward small flaking debris. This density curve predicts an idealised assemblage that contains the full range of flaking debris (≥5 mm) produced during many separate knapping events and can be used to investigate evidence of on-site core reduction or subtractive distortion caused by post-depositional disturbance. It is worth noting that as a result of the sieving procedure used by Schick ^128^, the smallest size interval (0-1 cm) is truncated at the lower bound and is actually 0.5-1 cm.

For analysis of the Area 7 assemblages, size distributions were calculated for each of the main layers using complete flakes, fragments, blades, retouched pieces, and shatter, following the approach of Schick^128^. Density plots were produced to examine artefact size distribution and compare with Schick’s ^128^ dataset of 5931 experimental flakes. Independent samples t-tests were used to test for statistically significant differences in population means between the Area 7 samples and Schick’s ^128^ dataset, and where data were not normally distributed nonparametric Mann-Whitney *U* tests were used instead.

The upper layers at Area 7 (GH1-3) show artefact size density curves similar to Schick’s ^128^ experimental knapping dataset, with the highest percentage of debitage falling within the 10-20 mm size range, and mean artefact sizes ranging from 26.27 to 19.5 mm. The contribution of lithics <40 mm in maximum dimension in the LGSS (83%), LPGSS (91%), DOSS (92%), and OBSS (84%) is similar to the experimental dataset (91%). In contrast, the GH4-5 layers show signs of possible subtractive distortion, with fewer artefacts in the 10-20 mm size class and distribution curves that are less skewed towards small debitage than the overlying layers. The proportion of lithics <40 mm in the GBSS (47%), LBCSS (62%), and DBBPS (57%) is considerably lower than both the experimental dataset and the GH1-3 layers.

The layers in the upper deposit all show a good fit with the distribution of the experimental dataset. The LPGSS sample displays a similar density curve to the LGSS sample, however there is a difference in mean values between these two layers (*t*(4.745)=210.7, *p*=<.0001), likely due to the difference in sample sizes. Despite the small sample, the LGSS density curve closely matches that of Schick’s experimental dataset, with a distribution skewed towards flakes in the 10-20 mm category, although there is also a small peak in the 70-80 mm range (Fig. S36). The DOSS sample shows a unimodal peak in the 10-20 mm size class. The OBSS density curve is broadly similar, although the 10-20 mm peak is lower than the DOSS, and there is a very small peak in the 50-60 mm size class, indicating that larger flakes are contributing more to the structure of this sample. Thus, the artefact size profiles are marginally different in the DOSS and OBSS, however they both exhibit a high frequency of small flaking debris in the 10-20 mm size class, with no signs of subtractive distortion in the samples. In both samples the full range of knapping debris appears to be present, with debitage <30 mm forming most of the recovered material. These results indicate that the small debitage component of the lithic samples from the upper deposit are present, with no evidence of post-depositional subtractive distortion or size sorting having affected these layers.

In the lower deposit, the GH4 layers display similar density curves to GH5, although both the GBSS and LBCSS curves differ substantially from Schick’s ^128^ dataset, with higher frequencies of larger flakes and the smallest size classes being underrepresented. Mann-Whitney U tests and independent samples t-tests indicate that the mean values for the GH4 layers are higher than the experimental dataset (GBSS: *U*= 56350, *p*=.061; LBCSS: *t*(8.396) = 230, *p*=<.0001). The GBSS sample contains the fewest artefacts of all layers, and the large difference in sample sizes precludes meaningful statistical comparisons with the other layers. The LBCSS, however, does show a difference in mean values to the overlying layers (OBSS: *t*(6.735)=323, *p*=<.0001; DOSS: *t*(-10.969)=250.5, *p*=<.0001; LPGSS: *t*(10.694)=256.7, *p*=<.0001; LGSS: *t*(*5*.595)=390.2, *p*=<.0001). Comparison of the density curves in Fig. S36 indicates that larger flakes contribute more to the structure of this assemblage than the GH2-3 layers, with a bimodal distribution and peaks at the 10-20 mm and 30-40 mm size intervals. Despite the presence of some small flaking debris, it is likely that some degree of winnowing is responsible for the underrepresentation of the smaller size classes within the GH4 sample.

The GH5 (DBBPS) sample differs from the experimental dataset and the GH1-3 layers. Mann-Whitney U tests indicate that mean artefact size in this sample is larger than the experimental dataset (*U*=107677, *p*=<.0001), but not larger than either the LBCSS (*U*=3077, *p*=.913) or GBSS (*U*=212 p=.815) samples. There is also a difference in mean values between the DBBPS sample and the upper layers (OBSS: *U*=11125.5, *p*=.006; DOSS: *U*=23304, *p*=<.0001; LPGSS: *U*=19250, *p*=<.0001; LGSS: *U*=3029, *p*=.009). Comparison with the experimental dataset shows that artefacts within the 10-20 mm size interval are also underrepresented within this layer, and it is likely that some degree of winnowing has resulted in the loss of artefacts in the smallest size classes, especially given that this sample was located close to the centre of the spring.


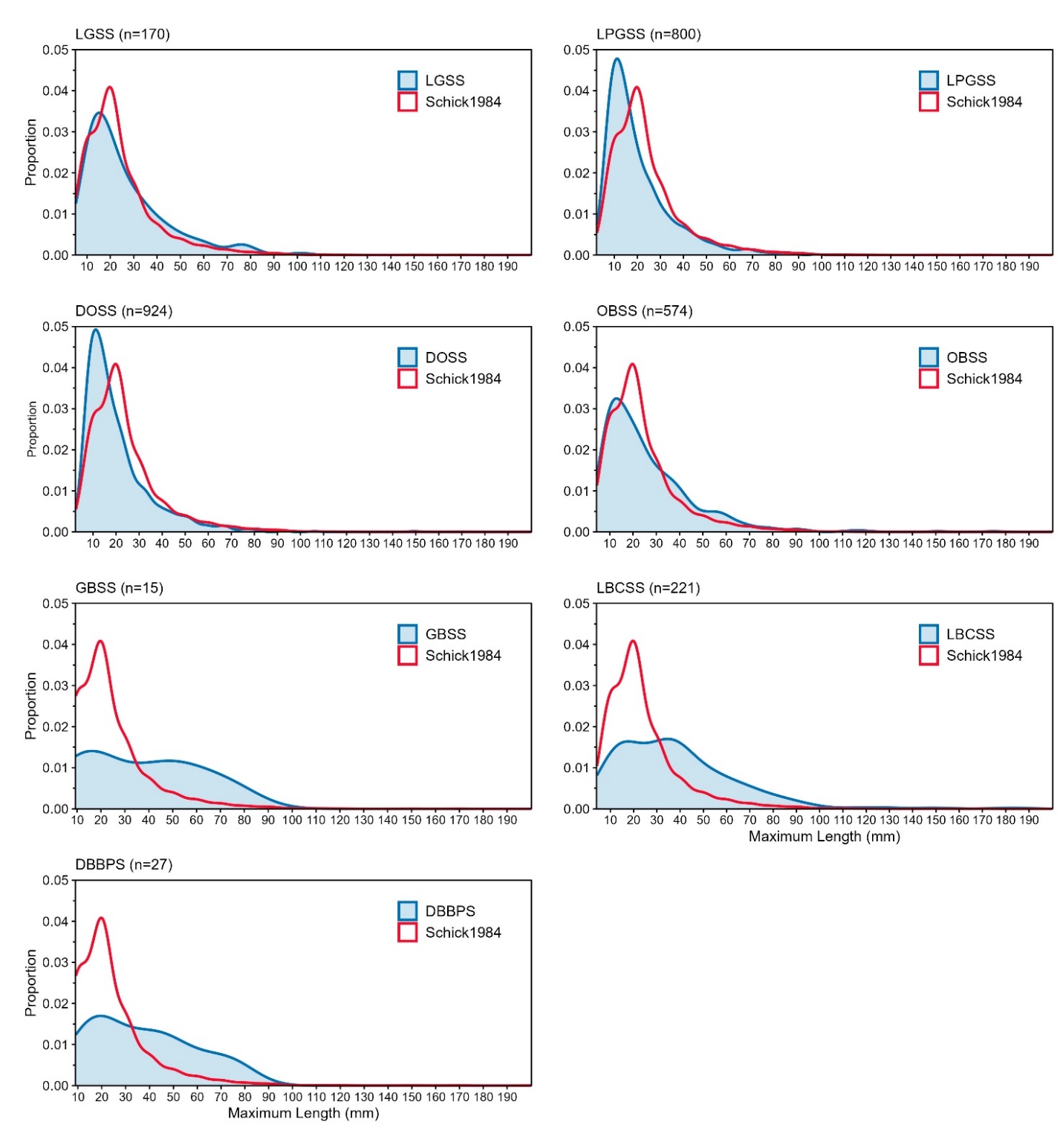


Fig. S36. Density plots showing artefact size distributions for all Area 7 layers.

5.11 Site Function

Site-specific activities during the Acheulian at AMZ7 were characterised by the manufacture of LCTs and on-site reduction of cores, exclusively made on quartzite, to produce debitage, some of which was retouched. The presence of complete and preform LCTs, as well as fragments, suggests that the manufacture and replacement of these tools was an important site function. There was a reasonable degree of flexibility in the operational sequences involved in LCT production, with large flakes struck from cobble (64-256mm) to boulder (>256mm) sized quartzite clasts (48%) and the direct reduction of quartzite cobbles (31%) used to produce bifacial handaxes and less commonly cleavers. Core reduction strategies were focussed on the exploitation of large flakes (>10mm in length or width) from quartzite cobbles, while many of the LCTs and cores appear to have been discarded during the early stages of reduction. This suggests that discard thresholds at the site were relatively low, supporting an interpretation of Amanzi Springs as an LCT workshop ^8,9^. However, there is also evidence for subsistence tasks being undertaken at the site, with scrapers and notched pieces being the most common retouch implements in the Acheulian layers ^8,9^.

Analysis of the debitage and cores suggest that the site was used in much the same way throughout the sequence. In the MSA layers, core reduction activities remained focussed on the use of local quartzites, although evidence for changes in landscape use and technological organisation appear in the form of exploitation of a wider range of fine-grained lithologies, and the use of greater quantities of quartzite sourced from outcrops rather than cobbles. There are also examples of non-quartzite cores made on lithologies with no associated debitage found within the assemblages, suggesting they were transported to the site after initial reduction elsewhere.

Supplementary References

1. Inskeep, R. R. Earlier stone age occupation at Amanzi: A preliminary investigation. *S. Afr. J. Sci.* **61**, 229–242 (1965).

2. Deacon, H. J. The Acheulian Occupation at Amanzi Springs Uitenhage District, Cape Province. *Ann. Cape Prov. Museums.* **8**, 89–189 (1970).

3. Deacon, H. J. The Early Stone Age Occupation at Amanzi Springs, Uitenhage District, Cape Province. (University of Cape Town, 1966).

4. Clark, J. D. *Kalambo Falls prehistoric site volume 3, the earlier cultures: Middle and Earlier Stone Age*. (University of California, 2001).

5. Barham, L. S., Tooth, S., Duller, G. A. T., Plater, A. J. & Turner, S. Excavations at Site C North, Kalambo Falls, Zambia: New insights into the mode 2/3 transition in South-Central Africa. *J. African Archaeol.* **13**, 187–214 (2015).

6. Duller, G. A. T., Tooth, S., Barham, L. S. & Tsukamoto, S. New investigations at Kalambo Falls, Zambia: Luminescence chronology, site formation, and archaeological significance. *J. Hum. Evol.* **85**, 111–125 (2015).

7. Barham, L. S. *et al.* Evidence for the earliest structural use of wood at least 476 , 000 years ago. (2023) doi:10.1038/s41586-023-06557-9.

8. Herries, A. I. R. *et al.* A marine isotope stage 11 coastal Acheulian workshop with associated wood at Amanzi Springs Area 1, South Africa. *PLoS One* **17**, 1–53 (2022).

9. Caruana, M. V. *et al.* A marine isotope stage 13 Acheulian sequence from the Amanzi Springs Area 2 Deep Sounding excavation, Eastern Cape, South Africa. *J. Hum. Evol.* **176**, 103324 (2023).

10. Rust, I. C. On the Sedimentation of the Table Mountain Group in the Western Cape Province. (University of Stellenbosch, 1967).

11. Issar, A. S. *On the regional hydrogeology of South Africa*. (1995).

12. Weaver, J. M. C. Potential of Table Mountain Group Aquifers and integration into catchment water management. in *A Synthesis of the Hydrogeology of the Table Mountain Group- Formation of a Research Strategy* (eds. Pertersen, K. & Parsons, R.) 249–255 (WRC Report, 2002).

13. Wu, Y. Groundwater recharge estimation in Table Mountain Group Aquifer system with a case study of Kammanassie area. (University of the Western Cape, 2005).

14. Maclear, L. G. A. The hydrogeology of the Uitenhage Artesian Basin with reference to the Table Mountain Group Aquifer. *Water SA* **27**, 499–505 (2001).

15. Muir, R. A., Bordy, E. M. M., Reddering, J. S. V. S. V & Viljoen, J. H. A. H. A. Lithostratigraphy of the enon formation (Uitenhage group) South Africa. *South African J. Geol.* **120**, 273–280 (2017).

16. Muir, R. A., Bordy, E. M. M., Reddering, J. S. V. S. V & Viljoen, J. H. A. H. A. Lithostratigraphy of the Kirkwood Formation (Uitenhage Group), including the Bethelsdorp, Colchester and Swartkops Members, South Africa. *South African J. Geol.* **120**, 281–293 (2017).

17. SACS. *Stratigraphy of South Africa, Part 1: Lithostratigraphy of the Republic of South Africa, South West Africa/Namibia, and the Republics of Bophuthatswana, Transkei and Venda*. (1980).

18. Roux, L. *Lithostratigraphy of the Alexandria Formation*. (1986).

19. Roux, L. *Lithostratigraphy of the Nanaga Formation (AlgoaGroup)*. (1992).

20. CGS. South African Council for Geoscience (2000).

21. Maclear, L. G. A. The Geohydrology of the Swartkops River Basin - Uitenhage Region, Eastern Cape. (University of Cape Town, 1996).

22. Summerfield, M. A. Silcrete as a palaeoclimatic indicator: evidence from southern Africa. *Palaeogeogr. Palaeoclimatol. Palaeoecol.* **41**, 65–79 (1983).

23. Wells, M. J. Plant remains from Amanzi Springs. *Ann. Cape Prov. Museums.* **8**, 191–194 (1970).

24. Folk, R. L. & Ward, W. C. Brazos River bar: a study in the significance of grain size parameters. *J. Sediment. Petrol.* **27**, 3–26 (1957).

25. Demuro, M. *et al.* New luminescence ages for the Galería Complex archaeological site: resolving chronological uncertainties on the acheulean record of the Sierra de Atapuerca, northern Spain. *PLoS One* **9**, e110169–e110169 (2014).

26. Aitken, M. J. *An Introduction to Optical Dating: The Dating of Quaternary Sediments by the Use of Photon-Stimulated Luminescence.* (Oxford University Press, 1998).

27. Duval, M. *et al.* Quantifying hydrofluoric acid etching of quartz and feldspar coarse grains based on weight loss estimates: implication for ESR and luminescence dating studies. *Anc. TL* **36**, 1–14 (2018).

28. Arnold, L. J., Demuro, M. & Ruiz, M. N. Empirical insights into multi-grain averaging effects from ‘pseudo’ single-grain OSL measurements. *Radiat. Meas.* **47**, 652–658 (2012).

29. Galbraith, R. F. A note on the variance of a background-corrected OSL count. *Anc. TL* **20**, 49–51 (2002).

30. Jacobs, Z., Duller, G. A. T. & Wintle, A. G. Interpretation of single grain distributions and calculation of. *Radiat. Meas.* **41**, 264–277 (2006).

31. Duller, G. A. T. Assessing the error on equivalent dose estimates derived from single aliquot regenerative dose measurements. *Anc. TL* **25**, (2007).

32. Auclair, M., Lamothe, M. & Huot, S. Measurement of anomalous fading for feldspar IRSL using SAR. *Radiat. Meas.* **37**, 487–492 (2003).

33. Huntley, D. J. & Lamothe, M. Ubiquity of anomalous fading in K-feldspars and the measurement and correction for it in optical dating. *Can. J. Earth Sci.* **38**, 1093–1106 (2001).

34. Arnold, L. J. *et al.* Evaluating the suitability of extended-range luminescence dating techniques over early and Middle Pleistocene timescales: Published datasets and case studies from Atapuerca, Spain. *Quat. Int.* **389**, 167–190 (2015).

35. Buylaert, J. P. *et al.* A robust feldspar luminescence dating method for Middle and Late<scp>P</scp>leistocene sediments. *Boreas* **41**, 435–451 (2012).

36. Arnold, L. J., Duval, M., Falguères, C., Bahain, J.-J. & Demuro, M. Portable gamma spectrometry with cerium-doped lanthanum bromide scintillators: Suitability assessments for luminescence and electron spin resonance dating applications. *Radiat. Meas.* **47**, 6–18 (2012).

37. Duval, M. & Arnold, L. J. Field gamma dose-rate assessment in natural sedimentary contexts using LaBr3(Ce) and NaI(Tl) probes: A comparison between the “threshold” and “windows” techniques. *Appl. Radiat. Isot.* **74**, 36–45 (2013).

38. Bøtter-Jensen, L. & Mejdahl, V. Assessment of beta dose-rate using a GM multicounter system. *Int. J. Radiat. Appl. Instrumentation. Part D. Nucl. Tracks Radiat. Meas.* **14**, 187–191 (1988).

39. Potts, P. J., Thompson, M., Chenery, S. R. N., Webb, P. C. & Kasper, H. U. Geopt13 - An International Proficiency Test for Analytical Geochemistry Laboratories - Report on Round 13 / July 2003 (Köln Loess). *Int. Assoc. Geoanalysts* (2003).

40. Prescott, J. R. & Hutton, J. T. Cosmic ray contributions to dose rates for luminescence and ESR dating: Large depths and long-term time variations. *Radiat. Meas.* **23**, 497–500 (1994).

41. Mejdahl, V. Internal radioactivity in quartz and feldspar grains. *Anc. TL* **5**, 10–17 (1987).

42. Bowler, J. M. *et al.* New ages for human occupation and climatic change at Lake Mungo, Australia. *Nature* **421**, 837–840 (2003).

43. Jacobs, Z., Duller, G. A. T., Wintle, A. G. & Henshilwood, C. S. Extending the chronology of deposits at Blombos Cave, South Africa, back to 140ka using optical dating of single and multiple grains of quartz. *J. Hum. Evol.* **51**, 255–273 (2006).

44. Pawley, S. M. *et al.* Age limits on Middle Pleistocene glacial sediments from OSL dating, north Norfolk, UK. *Quat. Sci. Rev.* **27**, 1363–1377 (2008).

45. Lewis, R. J. *et al.* Insights into subtropical Australian aridity from Welsby Lagoon, north Stradbroke Island, over the past 80,000 years. *Quat. Sci. Rev.* **234**, 106262 (2020).

46. Rees-Jones, J. Optical dating of young sediments using fine-grain quartz. *Anc. TL* **13**, 9–14 (1995).

47. Rees-Jones, J. & Tite, M. S. Optical dating results for British archaeological sediments. *Archaeometry* **39**, 177–187 (1997).

48. Huntley, D. J. & Baril, M. R. The K content of the K-feldspars being measured in optical dating or in thermoluminescence dating. *Anc. TL* **15**, 11–13 (1997).

49. Huntley, D. J. & Hancock, R. G. V. The Rb contents of the K-feldspar grains being measured in optical dating. *Anc. TL* **19**, 43–46 (2001).

50. Huntley, D. J. & Clague, J. J. Optical Dating of Tsunami-Laid Sands. *Quat. Res.* **46**, 127–140 (1996).

51. Huntley, D. J. & Lian, O. B. Using optical dating to determine when a sediment was last exposed to sunlight. in *Holocene Climate and Environmental Change in the Palliser Triangle: A Geoscientific Context for Evaluating the Impacts of Climate Change on the Southern Canadian Prairies* 211–222 (Geological Survey of Canada, 1999).

52. Alappat, L. *et al.* Chronology of Cauvery Delta Sediments from Shallow Subsurface Cores Using Elevated-Temperature Post-IR IRSL Dating of Feldspar. *Geochronometria* **37**, 37–47 (2010).

53. Lang, A. & Wagner, G. A. Infrared stimulated luminescence dating of holocene colluvial sediments using the 410 NM emission. *Quat. Sci. Rev.* **16**, 393–396 (1997).

54. Banerjee, D., Murray, A. S., Bøtter-Jensen, L. & Lang, A. Equivalent dose estimation using a single aliquot of polymineral fine grains. *Radiat. Meas.* **33**, 73–94 (2001).

55. Lang, A. *et al.* High-resolution chronologies for loess: comparing AMS 14C and optical dating results. *Quat. Sci. Rev.* **22**, 953–959 (2003).

56. Berger, G. W. *et al.* Luminescence chronology of cave sediments at the Atapuerca paleoanthropological site, Spain. *J. Hum. Evol.* **55**, 300–311 (2008).

57. Feathers, J. K., Casson, M. A., Schmidt, A. H. & Chithambo, M. L. Application of pulsed OSL to polymineral fine-grained samples. *Radiat. Meas.* **47**, 201–209 (2012).

58. Readhead, M. L. Absorbed dose fraction for 87Rb β particles. *Anc. TL* **20**, (2002).

59. Guérin, G., Mercier, M. & Adamiec, G. Dose-rate conversion factors: update. *Anc. TL* **29**, 5–8 (2011).

60. Mejdahl, V. THERMOLUMINESCENCE DATING: BETA‐DOSE ATTENUATION IN QUARTZ GRAINS. *Archaeometry* **21**, 61–72 (1979).

61. Brennan, B. J. Beta doses to spherical grains. *Radiat. Meas.* **37**, 299–303 (2003).

62. Aitken, M. J. *Thermoluminescence Dating*. (Academic Press, 1985).

63. Readhead, M. L. Thermoluminescence dose rate data and dating equations for the case of disequilibrium in the decay series. *Nucl. Tracks Radiat. Meas.* **13**, 197–207 (1987).

64. Hansen, V., Murray, A., Buylaert, J. P., Yeo, E.-Y. & Thomsen, K. A new irradiated quartz for beta source calibration. *Radiat. Meas.* **81**, 123–127 (2015).

65. Demuro, M., Arnold, L. J., Parés, J. M. & Sala, R. Extended-range luminescence chronologies suggest potentially complex bone accumulation histories at the Early-to-Middle Pleistocene palaeontological site of Huéscar-1 (Guadix-Baza basin, Spain). *Quat. Int.* **389**, 191–212 (2015).

66. Arnold, L. J. *et al.* OSL dating of individual quartz ‘supergrains’ from the Ancient Middle Palaeolithic site of Cuesta de la Bajada, Spain. *Quat. Geochronol.* **36**, 78–101 (2016).

67. Méndez-Quintas, E. *et al.* First evidence of an extensive Acheulean large cutting tool accumulation in Europe from Porto Maior (Galicia, Spain). *Sci. Rep.* **8**, 1–13 (2018).

68. Duller, G. A. T. Distinguishing quartz and feldspar in single grain luminescence measurements. *Radiat. Meas.* **37**, 161–165 (2003).

69. Durcan, J. A. & Duller, G. A. T. The fast ratio: A rapid measure for testing the dominance of the fast component in the initial OSL signal from quartz. *Radiat. Meas.* **46**, 1065–1072 (2011).

70. Wintle, A. G. & Murray, A. S. A review of quartz optically stimulated luminescence characteristics and their relevance in single-aliquot regeneration dating protocols. *Radiat. Meas.* **41**, 369–391 (2006).

71. Tsukamoto, S., Duller, G. A. T. & Wintle, A. G. Characteristics of thermally transferred optically stimulated luminescence (TT-OSL) in quartz and its potential for dating sediments. *Radiat. Meas.* **43**, 1204–1218 (2008).

72. Brown, N. D. & Forman, S. L. Evaluating a SAR TT-OSL protocol for dating fine-grained quartz within Late Pleistocene loess deposits in the Missouri and Mississippi river valleys, United States. *Quat. Geochronol.* **12**, 87–97 (2012).

73. Arnold, L. J. & Demuro, M. Insights into TT-OSL signal stability from single-grain analyses of known-age deposits at Atapuerca, Spain. *Quat. Geochronol.* **30**, 472–478 (2015).

74. Bartz, M. *et al.* Single-grain TT-OSL dating results confirm an Early Pleistocene age for the lower Moulouya River deposits (NE Morocco). *Quat. Geochronol.* **49**, 138–145 (2019).

75. Buylaert, J. P. *et al.* IRSL and post-IR IRSL residual doses recorded in modern dust samples from the Chinese Loess Plateau. *Geochronometria* **38**, 432–440 (2011).

76. Qin, J. T. & Zhou, L. P. Effects of thermally transferred signals in the post-IR IRSL SAR protocol. *Radiat. Meas.* **47**, 710–715 (2012).

77. Wang, X. L. & Wintle, A. G. Investigating the contribution of recuperated TL to post-IR IRSL signals in a perthitic feldspar. *Radiat. Meas.* **49**, 82–87 (2013).

78. Arnold, L. J. *et al.* Single-grain TT-OSL bleaching characteristics: Insights from modern analogues and OSL dating comparisons. *Quat. Geochronol.* **49**, 45–51 (2019).

79. Roberts, H. M. Testing Post-IR IRSL protocols for minimising fading in feldspars, using Alaskan loess with independent chronological control. *Radiat. Meas.* **47**, 716–724 (2012).

80. Vasiliniuc, Ş. *et al.* Testing the potential of elevated temperature post-IR IRSL signals for dating Romanian loess. *Quat. Geochronol.* **10**, 75–80 (2012).

81. Arnold, L. J., Bailey, R. M. & Tucker, G. E. Statistical treatment of fluvial dose distributions from southern Colorado arroyo deposits. *Quat. Geochronol.* **2**, 162–167 (2007).

82. Arnold, L. J. & Roberts, R. G. Stochastic modelling of multi-grain equivalent dose (De) distributions: Implications for OSL dating of sediment mixtures. *Quat. Geochronol.* **4**, 204–230 (2009).

83. Arnold, L. J. & Roberts, R. G. Paper I - Optically stimulated luminescence (OSL) dating of perennially frozen deposits in north-central Siberia: OSL characteristics of quartz grains and methodological considerations regarding their suitability for dating. *Boreas* **40**, 389–416 (2011).

84. Arnold, L. J. *et al.* Examining sediment infill dynamics at Naracoorte cave megafauna sites using multiple luminescence dating signals. *Quat. Geochronol.* **70**, 101301 (2022).

85. Demuro, M., Arnold, L. J., Aranburu, A., Gómez-Olivencia, A. & Arsuaga, J.-L. Single-grain OSL dating of the Middle Palaeolithic site of Galería de las Estatuas, Atapuerca (Burgos, Spain). *Quat. Geochronol.* **49**, 254–261 (2019).

86. Demuro, M., Arnold, L. J., González‐Urquijo, J., Lazuen, T. & Frochoso, M. Chronological constraint of Neanderthal cultural and environmental changes in southwestern Europe: MIS 5–MIS 3 dating of the Axlor site (Biscay, Spain). *J. Quat. Sci.* **38**, 891–920 (2023).

87. Hocknull, S. A. *et al.* Extinction of eastern Sahul megafauna coincides with sustained environmental deterioration. *Nat. Commun.* **11**, (2020).

88. Bailey, R. M. & Arnold, L. J. Statistical modelling of single grain quartz De distributions and an assessment of procedures for estimating burial dose. *Quat. Sci. Rev.* **25**, 2475–2502 (2006).

89. Arnold, L. J., Roberts, R. G., Galbraith, R. F. & DeLong, S. B. A revised burial dose estimation procedure for optical dating of youngand modern-age sediments. *Quat. Geochronol.* **4**, 306–325 (2009).

90. Galbraith, R. F., Roberts, R. G., Laslett, G. M., Yoshida, H. & Olley, J. M. Optical dating of single and multiple grains of quartz from Jinmium rock shelter, northern Australia: part I, experimental design and statistical models. *Archaeometry* **41**, 339–364 (1999).

91. Arnold, L. J. *et al.* Optical dating of perennially frozen deposits associated with preserved ancient plant and animal DNA in north-central Siberia. *Quat. Geochronol.* **3**, 114–136 (2008).

92. Arnold, L. J., Demuro, M., Navazo Ruiz, M., Benito-Calvo, A. & Pérez-González, A. OSL dating of the Middle Palaeolithic Hotel California site, Sierra de Atapuerca, north-central Spain. *Boreas* **42**, 285–305 (2013).

93. Olley, J. M., Caitcheon, G. G. & Roberts, R. G. The origin of dose distributions in fluvial sediments, and the prospect of dating single grains from fluvial deposits using optically stimulated luminescence. *Radiat. Meas.* **30**, 207–217 (1999).

94. Arnold, L. J. *et al.* Single-grain luminescence and combined U-series/ESR dating of the early Upper Palaeolithic Lagar Velho Rock Shelter, Leiria, Portugal. *Quat. Geochronol.* **83**, 101572 (2024).

95. Godfrey-Smith, D. I., Huntley, D. J. & Chen, W.-H. Optical dating studies of quartz and feldspar sediment extracts. *Quat. Sci. Rev.* **7**, 373–380 (1988).

96. Thomsen, K. J., Murray, A. S., Jain, M. & Bøtter-Jensen, L. Laboratory fading rates of various luminescence signals from feldspar-rich sediment extracts. *Radiat. Meas.* **43**, 1474–1486 (2008).

97. Duval, M. *et al.* Electron spin resonance dating of optically bleached quartz grains from the Middle Palaeolithic site of Cuesta de la Bajada (Spain) using the multiple centres approach. *Quat. Geochronol.* **37**, 82–96 (2017).

98. Galbraith, R. F. A simple homogeneity test for estimates of dose obtained using OSL. *Anc. TL* **21**, 75–77 (2003).

99. Bar-Matthews, M. *et al.* A high resolution and continuous isotopic speleothem record of paleoclimate and paleoenvironment from 90 to 53 ka from Pinnacle Point on the south coast of South Africa. *Quat. Sci. Rev.* **29**, 2131–2145 (2010).

100. Stockmarr, J. Tables with spores used in absolute pollen analysis. *Pollen et spores* **13**, 615–621 (1971).

101. Goldman-Neuman, T. & Hovers, E. Raw material selectivity in Late Pliocene Oldowan sites in the Makaamitalu Basin, Hadar, Ethiopia. *J. Hum. Evol.* **62**, 353–366 (2012).

102. Dibble, H. L., Schurmans, U. A., Iovita, R. P. & McLaughlin, M. V. The Measurement and Interpretation of Cortex in Lithic Assemblages. *Am. Antiq.* **70**, 545–560 (2005).

103. Marwick, B. What attributes are important for the measurement of assemblage reduction intensity? Results from an experimental stone artefact assemblage with relevance to the Hoabinhian of mainland Southeast Asia. *J. Archaeol. Sci.* **35**, 1189–1200 (2008).

104. Braun, D. R., Tactikos, J. C., Ferraro, J. V., Arnow, S. L. & Harris, J. W. K. Oldowan reduction sequences: methodological considerations. *J. Archaeol. Sci.* **35**, 2153–2163 (2008).

105. Dibble, H. L. Middle paleolithic scraper reduction: Background, clarification, and review of the evidence to date. *J. Archaeol. Method Theory* **2**, 299–368 (1995).

106. Neeley, M. & Lee, C. Assessing Cortex Ratios: An Example from the Beaucoup Site (24PH188/189) in the Northwestern Plains. *Lithic Technol.* **0**, 1–13 (2020).

107. Wilkins, J. *et al.* *Lithic technological responses to Late Pleistocene glacial cycling at Pinnacle Point Site 5-6, South Africa*. *PLoS ONE* vol. 12 (2017).

108. Luedtke, B. E. *An archaeologist’s guide to chert and flint*. *Chert and flint* (Los Angeles : Institute of Archaeology, University of California, 1992).

109. Shea, J. J. *Prehistoric Stone Tools of Eastern Africa: A Guide*. (Cambridge University Press, 2020). doi:DOI: 10.1017/9781108334969.

110. O’Driscoll, C. A. & Mackay, A. On the Operation of Retouch in Southern Africa’s Early Middle Stone Age. *J. Paleolit. Archaeol. 2020 34* **3**, 1149–1179 (2020).

111. Hiscock, P. & Clarkson, C. Retouched Notches at Combe Grenal (France) and the Reduction Hypothesis. *Am. Antiq.* **72**, 176–190 (2007).

112. Bordes, F. *Typologie du paléolithique ancien et moyen*. (CNRS Editions, 1961).

113. Close, A. E. On the Validity of Middle Paleolithic Tool Types: A Test Case from the Eastern. *J. F. Archaeol.* **18**, 256–264 (1991).

114. Holdaway, S. J., McPherron, S. P. & Roth, B. Notched Tool Reuse and Raw Material Availability in French Middle Paleolithic Sites. *Am. Antiq.* **61**, 377–387 (1996).

115. Eren, M. I. *et al.* Defining and measuring reduction in unifacial stone tools. *J. Archaeol. Sci.* **32**, 1190–1201 (2005).

116. Holdaway, S. J. & Stern, N. *A record in stone : the study of Australia’s flaked stone artefacts*. (Museum Victoria and Aboriginal Studies Press, 2004).

117. Holdaway, S. J. *Continuity and Change. An Investigation of the Flaked Stone Artefacts from the Pleistocene Deposits at Bone Cave South West Tasmania, Australia*. *Report of the Southern Forests Archaeological Project* vol. 2 (La Trobe University, 2004).

118. Braun, D. R. Examining Flake Production Strategies: Examples from the Middle Paleolithic of Southwest Asia. *Lithic Technol.* **30**, 107–125 (2005).

119. Braun, D. R. & Harris, J. W. K. Technological developments in the Oldowan of Koobi Fora: Innovative techniques of artifact analysis and new interpretations of Oldowan behavior. in *Oldowan: Rather More thanSmashing Stones* (eds. Mora, R. & de la Torre, I.) 132–144 (Centre d’Estudis del Patrimoni Arqueològic de la Prehistòria, 2003).

120. Mackay, A. A method for estimating edge length from flake dimensions: use and implications for technological change in the southern African MSA. *J. Archaeol. Sci.* **35**, 614–622 (2008).

121. Lotter, M. G. & Kuman, K. A. The Acheulean in South Africa, with announcement of a new site (Penhill Farm) in the lower Sundays River Valley, Eastern Cape Province, South Africa. *Quat. Int.* **480**, 43–65 (2018).

122. Kuman, K. A. & Field, A. S. The Oldowan Industry From Sterkfontein Caves, South Africa. in *The Cutting Edge: New Approaches to the Archaeology of Human Origins* (eds. Schick, K. D. & Toth, N.) 151–169 (Stone Age Institute Press, 2009).

123. Kuman, K. A., Lotter, M. G. & Leader, G. M. The Fauresmith of South Africa: A new assemblage from Canteen Kopje and significance of the technology in human and cultural evolution. *J. Hum. Evol.* **148**, 102884 (2020).

124. Thompson, E. Acheulean artifact accumulation and early hominin land use, Garden Route Casino Road, Pinnacle Point, South Africa. *Geoarchaeology* **24**, 402–428 (2009).

125. Paddayya, K. & Petraglia, M. D. Formation processes of Acheulian localities in the Hunsgi and Baichbal Valleys, peninsular India. in *Formation processes in archaeological context* (eds. Goldberg, P., Nash, D. T. & Petraglia, M. D.) 61–82 (Prehistory Press, 1993).

126. Schiffer, M. B. Toward the Identification of Formation Processes. *Am. Antiq.* **48**, 675–706 (1983).

127. Rottländer, R. The Formation Of Patina On Flint. *Archaeometry* **17**, 106–110 (1975).

128. Schick, K. D. Processes of Palaeolithic Site Formation: An Experimental Study. (University of California, Berkeley, 1984).

129. Schick, K. D. *Stone age sites in the making: experiments in the formation and transformation of archaeological occurrences*. (B.A.R., 1986).

130. Bertran, P., Lenoble, A., Todisco, D., Desrosiers, P. M. & Sørensen, M. Particle size distribution of lithic assemblages and taphonomy of Palaeolithic sites. *J. Archaeol. Sci.* **39**, 3148–3166 (2012).

131. Mackay, A., Hallinan, Emily & Steele, T. E. Provisioning Responses to Environmental Change in South Africa’s Winter Rainfall Zone: MIS5-2. in *Lithic Technological Organisation and Paleoenvironmental Change* (eds. Robinson, E. & Sellet, F.) 13–36 (Springer International Publishing, 2018).

132. Kuhn, S. L. *Mousterian lithic technology : an ecological perspective*. (1995).

133. Schick, K. D. Modeling the formation of Early Stone Age artifact concentrations. *J. Hum. Evol.* **16**, 789–807 (1988).

134. NASA/METI/AIST/Japan Spacesystems and U.S./Japan ASTER Science Team. *ASTER Global Digital Elevation Model V003*. NASA Land Processes Distributed Active Archive Center. (2019) https://doi.org/10.5067/ASTER/ASTGTM.003
